# An OMV Vaccine Derived from a Capsular Group B Meningococcus with Constitutive FetA Expression: Preclinical Evaluation of Immunogenicity and Toxicity

**DOI:** 10.1371/journal.pone.0134353

**Published:** 2015-09-21

**Authors:** Gunnstein Norheim, Holly Sanders, Jardar W. Mellesdal, Idunn Sundfør, Hannah Chan, Carina Brehony, Caroline Vipond, Chris Dold, Rory Care, Muhammad Saleem, Martin C. J. Maiden, Jeremy P. Derrick, Ian Feavers, Andrew J. Pollard

**Affiliations:** 1 Oxford Vaccine Group, Department of Paediatrics, University of Oxford, and the NIHR Oxford Biomedical Research Centre, Oxford, United Kingdom; 2 Norwegian Institute of Public Health, Oslo, Norway; 3 National Institute of Biological Standards and Control, Potters Bar, United Kingdom; 4 Department of Zoology, University of Oxford, Oxford, United Kingdom; 5 University of Manchester, Manchester, United Kingdom; Universidad Nacional de la Plata, ARGENTINA

## Abstract

Following the introduction of effective protein-polysaccharide conjugate vaccines against capsular group C meningococcal disease in Europe, meningococci of capsular group B remain a major cause of death and can result in debilitating sequelae. The outer membrane proteins PorA and FetA have previously been shown to induce bactericidal antibodies in humans. Despite considerable antigenic variation among PorA and FetA OMPs in meningococci, systematic molecular epidemiological studies revealed this variation is highly structured so that a limited repertoire of antigenic types is congruent with the hyperinvasive meningococcal lineages that have caused most of the meningococcal disease in Europe in recent decades. Here we describe the development of a prototype vaccine against capsular group B meningococcal infection based on a N. meningitidis isolate genetically engineered to have constitutive expression of the outer membrane protein FetA. Deoxycholate outer membrane vesicles (dOMVs) extracted from cells cultivated in modified Frantz medium contained 21.8% PorA protein, 7.7% FetA protein and 0.03 μg LPS per μg protein (3%). The antibody response to the vaccine was tested in three mouse strains and the toxicological profile of the vaccine was tested in New Zealand white rabbits. Administration of the vaccine, MenPF-1, when given by intramuscular injection on 4 occasions over a 9 week period, was well tolerated in rabbits up to 50 μg/dose, with no evidence of systemic toxicity. These data indicated that the MenPF-1 vaccine had a toxicological profile suitable for testing in a phase I clinical trial.

## Introduction

Meningococcal disease is a severe, potentially life-threatening infection, with highest incidence among children less than five years of age. The incidence in high income countries has decreased in recent decades, in part driven by the implementation of protein-conjugated polysaccharide vaccines providing protection against capsular group C meningococci [1], but perhaps also as a result of environmental changes including reductions in smoking rates [2]. Changes in the meningococci circulating in asymptomatic carriage and changes in population immunity may also have led to the decrease in disease incidence. Conjugate vaccines are available against meningococci expressing polysaccharide capsules of capsular groups A, C, Y and W, which further reduce disease incidence where they are deployed. The development of a similar capsular group B polysaccharide vaccine has been hampered by poor immunogenicity and a concern about the risk of such a vaccine generating cross-reactive antibodies [3] against neural tissue [4].

To date, the most successful alternative vaccine formulations have included outer membrane vesicles (OMVs) [5]: these vaccines are shown to be safe, moderately reactogenic, and provide protection against outbreaks of capsular group B meningococcal disease caused by the vaccine strain. However, their routine use against endemic disease is complicated by the high degree of variation in sequence and expression levels among meningococcal outer membrane proteins. The protection provided by OMV vaccines, made from the OMVs of wild type circulating invasive meningococci (wt OMV vaccines), is largely attributed to their ability to induce bactericidal antibodies to the immunodominant and antigenically variable outer membrane protein (OMP) porin A (PorA) [5]. The challenge has been to produce a vaccine based on OMP antigens that is broadly cross-protective against diverse meningococci. One approach has been to supplement an OMV vaccine formulation with additional antigens identified from genome sequence data such as the recently licensed Novartis vaccine, Bexsero [6]. In contrast, utilizing the extensive epidemiological data available, we have proposed a multivalent OMV approach based on two OMP antigens, PorA and FetA [7]. Molecular epidemiological studies have shown that meningococcal antigenic diversity is structured by immunoselection, effectively limiting the number of antigenic variants that would have to be included in a multivalent vaccine to offer broad protection [8].

The outer membrane protein FetA is present in nearly all invasive meningococcal isolates, and like PorA exhibits a high level of antigenic diversity [3]. Moreover, its expression is dependent on the concentration of available free iron [9], consistent with its function as an iron transporter [9]. FetA has, in previous studies, been shown to induce bactericidal antibodies in animals and in humans following infection or vaccination with OMV vaccines [10–14]. The bactericidal antibodies target one specific variable region (VR, ‘loop 5’) of the FetA protein [9] which is immunodominant and shields the second most immunogenic domain (loop 3) [15]. The FetA content of wild-type OMV vaccines is, however, batch-variable, with relative protein content 1–8% depending on the amount of available iron in the growth medium [16].

In the current study, we have generated a meningococcus that was constitutive for FetA expression by genetic modification of the promoter region of the fetA gene to constitutively express high levels of the protein. We demonstrate how a combined PorA and FetA vaccine can be formulated from this strain to produce a PorA/FetA vaccine. Secondly, we demonstrate the suitability of this vaccine concept for scale-up of manufacture, the toxicological profile of the OMV in rabbits and impact of genetic variation on immune responses as demonstrated by use of a well characterized panel of genetically engineered target N. meningitidis strains in the bactericidal assay.

## Materials and Methods

### Ethics statement

Animal studies were conducted according to the UK Home Office regulations under licence number 80/2157 and were approved by the National Institute for Biological Standards and Control ethics committee. Bleeds were taken under terminal anaesthesia. The GLP testing with New Zealand White rabbits was performed under contract to Charles River, Edinburgh, United Kingdom according to Good Laboratory Practice (GLP), relevant EMA guidelines (CPMP/ICH/286/95, CPMP/SWP/465/95 and CHMP/SWP/488313/07), in accordance with licences granted under the terms of the UK Animals (Scientific Procedures) Act 1986 and in compliance with the European Directive 2010/63/EU. Potency testing of the vaccine in Norway was undertaken according to the Norwegian Animal Welfare Act.

### Generation of the vaccine strain and verification of expression and genomic stability

**Fig 1:**
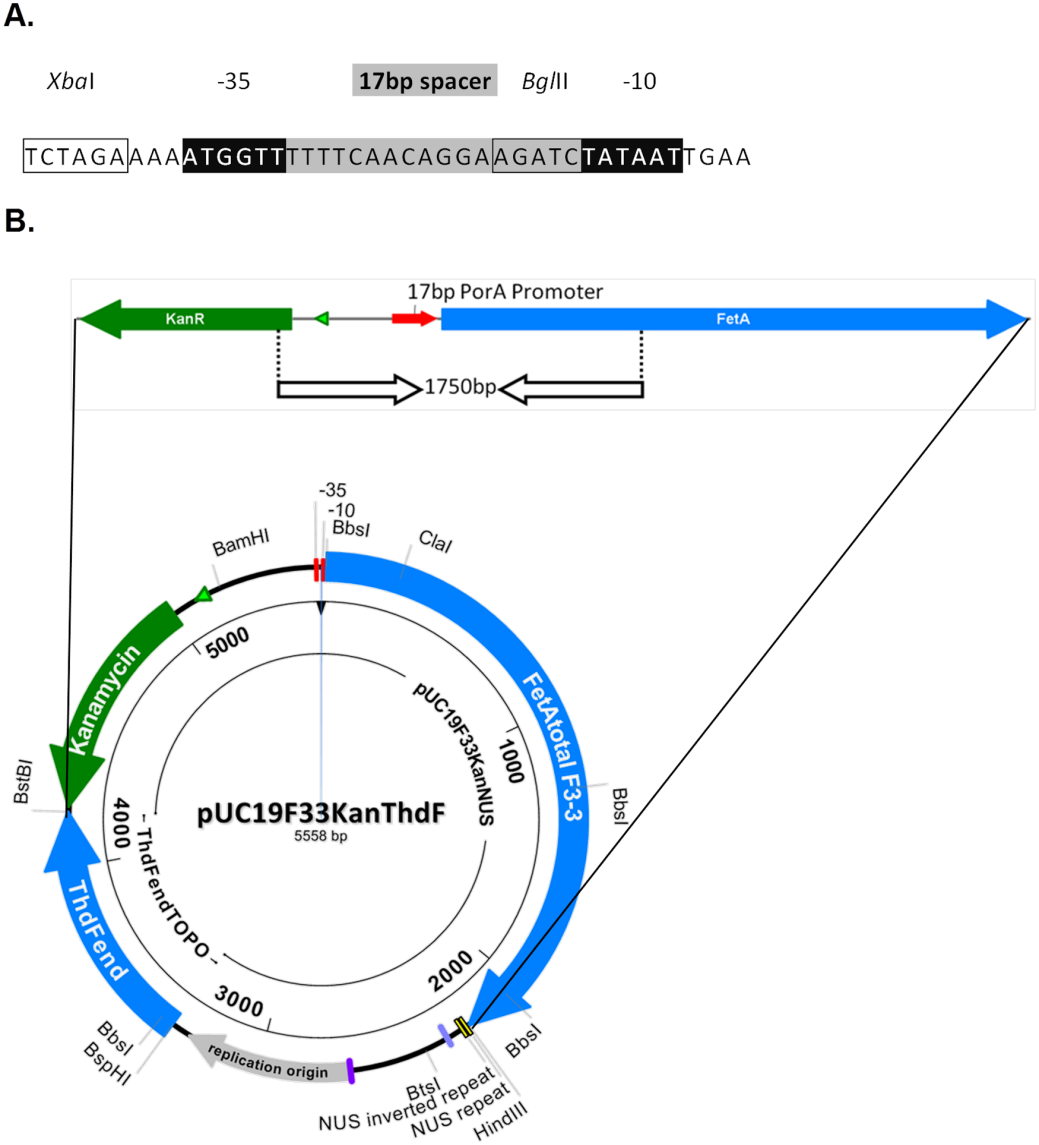
A. Genetic modification of N. meningitidis strains. Promoter region designed to alter expression levels of FetA. Spacer area (17 bp) indicated in grey shading. Restriction enzyme sites for XbaI and BglII indicated along with -35 and -10 sequences. B. Schematic drawing of the plasmid used for transformation of the N. meningitidis strain 2851 (H44/76), and the modified region in the transformed strains and the region amplified by the initial PCR screening.

The parent isolate for preparation of the vaccine strain was a capsular group B N. meningitidis isolate, H44/76, derived from a patient who developed MenB disease in Norway in 1976 and characterized as B:15:P1.7,16: F3-3: ST-32 (cc32) [16,17]. The H44/76 isolate used for this study was a sub-culture previously received from NIPH and retained in the NIBSC strain collection as strain 2851. Constitutive expression of fetA was achieved by replacing the native promoter sequence with a novel promoter, based on the nucleotide sequences of the PorA and PorB promoter regions. A 17bp spacer between the -10 and -35 sequences was found to result in consistently high expression levels of FetA (Fig 1A).

The novel promoter was introduced by transforming the 2851 strain with the plasmid shown in Fig 1B. Briefly, strain 2851 was resuspended from overnight growth on blood agar (Oxoid, Cambridge, UK) to an OD_650nm_ of 0.2–0.3 in 1ml Mueller Hinton broth (Oxoid) containing 8 mM MgCl_2_ (Sigma Aldrich, St Louis, Missouri, USA). Undigested plasmid DNA (1 μg) was added to and the suspension was incubated at 37°C with 150 rpm rotational shaking for 4 hours. Cells were collected by centrifugation and plated onto Mueller-Hinton agar (Oxoid) containing 50 μg/ml kanamycin (Sigma Aldrich) to select from plasmid integration. Kanamycin resistant colonies were screened for the presence of the new promoter region by colony PCR. PCR products were analyzed on a 0.8% agarose gel to check for band lengths, which were subsequently checked by nucleotide sequencing to confirm the presence of the promoter region.

The protein profiles of OMVs prepared by deoxycholate (DOC) detergent extraction as described previously [18,19] were assessed on 8% SDS-PAGE (BioRad) run at 10 mA overnight and stained with Silver Stain Protein kit (Amersham Biosciences). The effect of iron limitation on FetA expression was also assessed by comparing membrane extracts for protein profiles, as described above, derived from strains grown in Tryptone Soya Broth (TSB) with or without 50 μM deferoxamine mesylate (DFAM). Finally, the vaccine strain (3207) was assessed for genetic stability, i.e. whether the inserted construct into the parent strain (2851) was stable after consecutive passages. This was performed by growing strain 3207 on non-selective tryptic soy agar (TSA) plates with Vitox (Oxoid), and comparing the number of colonies recovered on selective (Kanamycin 50 μg/ml) and non-selective TSA plates through 10 consecutive passages. Outer membrane protein preparations and genomic DNA preparations were then prepared from before and after the 10 passages, with and without selective growth agar medium. Outer membrane preparations were run on a 12% SDS-PAGE (Biorad), run at 10 mA overnight and visualized by Coomassie staining. The nucleotide sequence of the promoter region of the fetA gene was determined to assess potential variability as previously described.

### Generation of isogenic strains to evaluate vaccine immunogenicity

Three genetically modified N. meningitidis strains, with combinations of high and constitutive expression of FetA and PorA or knockout mutants of these, were generated for use in serum bactericidal activity (SBA) assay to evaluate PorA and FetA-specific vaccine responses following vaccination of research animals and human volunteers (Table 1).

**Table 1:** Meningococcal strains used in this study.

Strain	Genotype	PorA expression ^a^	FetA expression ^a^	Source
SMenPF1.2 (3207) ^b^	H44/76 fetAp_17bp_	Wild-type and high	Constitutive	This study
3043	H44/76 fetA::kan	Wild-type and high	Absent	[28]
3311	H44/76 fetA::kan porA::ery	Absent	Absent	[Sanders et al., unpublished observations]
3312	H44/76 fetAp_17bp_ porA::ery	Absent	Constitutive	[Sanders et al., unpublished observations]

^a^ Expression levels of major outer membrane proteins PorA and FetA are indicated.

^b^ The vaccine strain SMenPF1.2 is the master seed lot that was prepared under GMP conditions from the strain 3207.

Strain 3043 had been transformed previously to interrupt expression of the FetA protein [3]. To generate the two new mutants with interrupted expression of the full-length PorA protein, strains SMenPF1.2 (3207) and 3043 were transformed with plasmid PorAEryF (Sanders et al., unpublished observations). Meningococci were transformed as described above and plated onto selective plates containing 50 μg/mL erythromycin (Sigma Aldrich). The expression of FetA and PorA in the three strains was verified as described above for the vaccine strain. In addition, whole cells of the strain panel were tested by immunoblotting for binding with a PorA-specific monoclonal antibody (Anti-P1.16, 01/538, NIBSC, Potters Bar, U.K.) or a FetA-specific mouse antiserum. The latter was generated by immunization of NIH/OlaHsd mice with recombinant FetA variant F3-3 protein (Sanders et al., unpublished observations).

### Serum bactericidal activity

The suitability of the strain panel was further tested in serum bactericidal activity (SBA) assay, using the tilted agar plate method and human complement as previously described [20]. Exogenous human complement without intrinsic bactericidal activity was sourced from consenting healthy adults and used at 25% (vol/vol) with each target strain having a single complement source. Bacterial strains were grown overnight to single colonies on blood agar plates at 37°C and 5% CO_2_. Approximately 50 colonies were then sub-cultured for 4 hours before being reconstituted in Hanks buffered salt solution (Gibco) with 0.5% bovine serum albumin (Sigma Aldrich). The bacteria were further diluted to give approximately 100 colony forming units per 10 μl used for the assay. The SBA titre was defined as the reciprocal of the highest dilution of serum that yielded ≥50% decrease in colony forming units relative to that of control wells within 60 min at 37°C without CO_2_. Pools of sera from mice immunized with OMVs derived from the vaccine strain SMenPF1.2 (batch FMOX1102) were tested in duplicate for their SBA against the same panel, along with a PorA P1.16 mAb (NIBSC Cat. No. 01/538). The pools were composed of all available sera from each test group: At release (0 months): for the saline group n = 20, for the 2.5 μg group n = 20 and for the 10 μg group n = 10. After 12 months at 5°C and 2.5 μg dose: n = 19. After 24 months at 5°C and 2.5 μg dose: n = 20.

### Impact of genetic modifications on vaccine and evaluation strains

Genomic DNA from the modified strains SMenPF1.2 (3207), 3043, 3311 3312 and parent strain 2851 was extracted and purified using the Wizard Genomic DNA purification kit (Promega). Standard Illumina multiplex genomic libraries were prepared and sequenced on the Illumina Genome Analyser II platform generating 100 bp paired end reads as previously described [21]. Genome sequences were assembled using Velvet version 1.2.08 [22] with optimal parameters determined by the Velvet-Optimiser script. Genomes were uploaded to the Neisseria PubMLST isolates database (http://pubmlst.org/neisseria/) which is implemented with the BIGSdb database platform [23]. When sequence data for each isolates record are deposited in PubMLST, they are scanned against the definitions databases using the BLAST algorithm. When a known allele is identified, it is recorded as a designation for that locus in the isolate record, and the position of the allele in the contig is marked (tagged) [24]. Using the GenomeComparator tool, the four isolates as well as the parent strain were compared for genes related to the major antigens in the vaccine (FetA, PorA, PorB, LPS) and all loci defined in the FAM18 genome annotation [25]. Repetitive sequences due to multiple copies of insertion element transposases and pseudogenes were removed when using the FAM18 annotation as the source of comparator coding sequences. Paralogous loci that matched multiple alleles were also removed from the analysis. Sequence conflicts were checked by remapping using the Bowtie version 1 short-read aligner [26]. For a specific locus of interest, the contig containing the locus was extracted from the isolate sequence bin and used as the reference segment. Short-reads for each isolate being investigated were converted to SAM files and mapped against the reference segment using a randomized alignment in order to avoid mapping bias. The aligned SAM files were visualized using the Tablet software package [27] and read depth and conflicting nucleotides of interest were identified and examined. CGView was used to visualize the locus positions in the circular H44/76 genome [28].

### GMP production of OMVs, final vaccine product and GLP study control product

**Fig 2:**
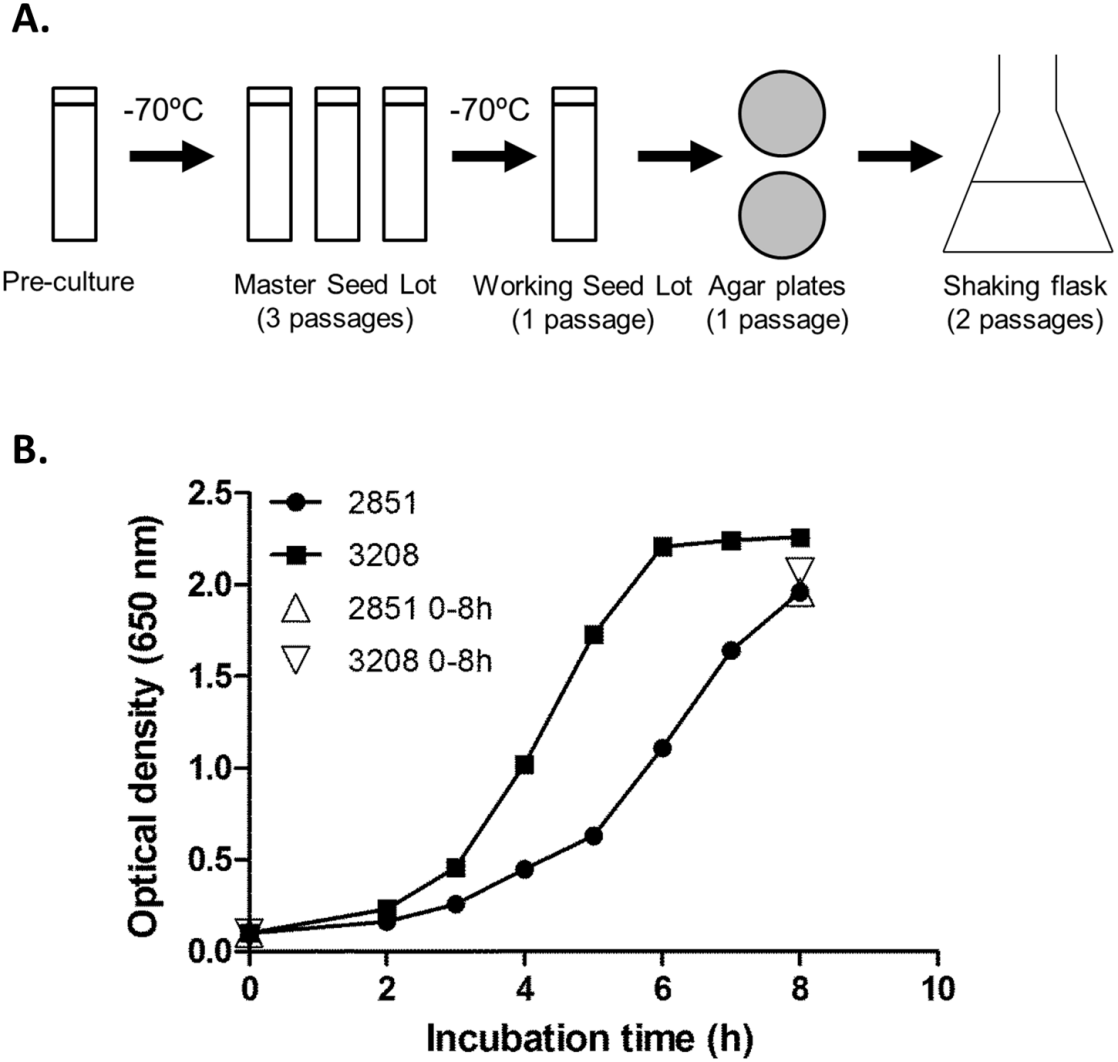
Meningococcal cultivation procedures. A. The ideal growth times on agar plates under production of Seed Lots were 10–12 hours for the 1st passage, 7–8 hours for the 2nd passage, and 6–8 hours for the 3rd passage at 35°C. When cultivated in 1.5L medium in 2.8 L shaking flasks, shaking speed 200 rpm and temperature 35°C and an initial OD_650_ = 0.1, the length of the lag phase was approximately 2 hours, whereas the log-phase seemed to end at 7–8 hours with an OD_650_ of ~2. B. Comparison of growth in 1.0 L modified Frantz liquid medium in a 2L baffled shaking flask for the strains 3208 (MenPF-1 vaccine strain) and the 2851 strain (wild type H44/76 strain).

A GMP production process for OMV extraction was established at the Biomanufacturing unit at the NIPH, Oslo, Norway based on experiments at OVG, Oxford, U.K. and on experience of the Norwegian MenBvac vaccine production process [18]. After passaging the vaccine strain SMenPF1.2 three times on TSA+Vitox agar plates certified to be TSE/BSE-free, a seed lot was produced and evaluated in Oxford for growth characteristics at the same scale as to be performed at NIPH, performed in shaking flasks with a growth medium volume ranging from 0.2 to 1.5 L in modified Frantz medium [17] and to finalise the process parameters. Meningococci from the working seed bank (WSB) were spread to confluent growth on TSA plates with Vitox growth supplement (Oxoid, U.K.), incubated at 35°C and 5% CO2 using anaerobic jars for 12–24 hours, harvested in 10 mL modified Frantz medium [17] and used to inoculate 1–1.5L Frantz medium in single-use 2800 mL baffled shaking flasks (Nalgene, U.S.) Fig 2).

Growth with shaking was performed at 35°C and 150–200 rpm for 5–8 hours, and thereafter the bacterial suspension was harvested into a sterile 10 L flask. The culture was sampled for yield (OD at 650 nm) and purity by testing with Gram stain and growth on TSA and plate count agar plates. Concentration of the cell mass was performed in a class II biosafety cabinet in a P3 GMP laboratory facility by cross-flow filtration (CFF) using a Millipore CFF filter until a final volume of 0.6–1.1 L was reached. pH adjustment was performed by adding 0.1 M Tris/HCl (pH 8.6) with 10 mM EDTA (buffer A), and a second buffer (10% sodium deoxycholate (DOC) in Tris/HCl, pH 8.9; buffer A1) was added to obtain a final concentration of 0.5% DOC; leading to the inactivation of the cell mass and extraction of OMVs. An equal volume of Buffer A was slowly added, homogenized by magnet stirring and pH measured to ensure a pH > 8.0. Ninety mL of Buffer A1 was slowly added and the suspension was kept homogenous by stirring for 30 minutes and then sampled to check the inactivation procedure. Cell debris was removed by centrifugation at 12000 rpm for 90 minutes at 2–8°C, supernatants were pooled and centrifuged at 19000 rpm for 10 hours. The pellet was suspended in a 1:2 mix of buffers B (0.05 M Tris/HCl, 2 mM EDTA, 2.5% DOC) and buffer B1 (0.05 M Tris/HCl, 2 mM EDTA, 0.5% DOC, 30% sucrose) and homogenized by magnetic stirring for 5 hours or overnight at room temperature and sonication for 1 minute. The suspension was then subjected to a new centrifugation at 19000 rpm for 5 hours, for thereafter to suspend the pellet in a 3% sucrose solution. Homogenisation was ensured by magnetic stirring for 5 hours or overnight at room temperature and sonication for 1 minute. The OMVs were diluted in 3% sucrose solution to obtain a final protein concentration of about 1 mg/mL, briefly sonicated and filtered using a MiniKleenPak capsule with 0.2 μm Supor EKV polyethersulfone low-protein-binding membrane filter (Pall corporation, U.S.). The OMVs were adsorbed to the adjuvant aluminium hydroxide (Brenntag, Denmark), suspended in a 3% sucrose solution, diluted to an end concentration of 50 μg protein/mL and ultimately filled in sealed, capped and labelled 2R injection vials for single dose-use with an injection volume of 0.5 ml (equals 25 μg of OMV protein, 12.5 mg sucrose and 0.333% w/v aluminum hydroxide in water for injection). Three GMP batches were produced; FMOX1002, FMOX1005 and FMOX1007, which were stored at 5°C (± 3°C) until use.

A control vaccine product was specifically manufactured for the GLP immunotoxicological study in rabbits, using the same reagents and equipment as for the vaccine product and according to GMP regulations. The product (termed MOX Control) contained a suspension of aluminium hydroxide (0.333% w/v) diluted in 3% sucrose in water for injection and was tested for extractable volume, sterility, aluminum content, pH, endotoxin level and appearance; with the same specifications for these parameters as for the final vaccine product (Table 2).

**Table 2:** Acceptance criteria and results from quality control testing of OMVs and final vaccine product MenPF-1.

**I. OMVs**			**Batch name**		
**Parameter**	**Specification**	**Method**	**MOX1101**	**MOX1104**	**MOX1106**
Inactivation control	Inactivated material		Pass	Pass	Pass
Total protein pre-formulation	To be reported	Folin Lowry	0.74 mg/mL	0.80 mg/mL	0.84 mg/mL
Bioburden, pre sterile filtration	Total aerobic microbial count (TAMC); Total yeasts and moulds count (TYMC) and total bioburden: all <10^1^/mL		Pass	Pass	Pass
Identity, antigen pattern	To be reported	SDS-PAGE Ph. Eur. 2.2.31	FetA: 8.0, PorA: 21.7 and PorB: 32.6	FetA: 7.3, PorA: 21.5 and PorB: 33.0	FetA: 7.7, PorA: 21.8 and PorB: 31.9
Identity, presence of antigens	Verify detection of 70 kDa (FetA F3-3), Class 1 (P1.16) and Class 3 (P3.15) with antigen specific monoclonal antibodies	Immunoblotting with specific antibodies	Detected	Detected	Detected
Deoxycholate	<0.4 μg/μg protein	Total Bile Acid Assay, Bio-Stat	0.26	0.20	0.20
LPS content	Total content: <0.12. LPS L3,7,9: to be reported	SDS-PAGE, silver staining and digital scanning	Total content: 0.044 and L3,7,9 content: 0.043	Total content: 0.024 and L3,7,9 content: 0.024	Total content: 0.030 and L3,7,9 content: 0.030
Total protein	0.45–1.24 mg/mL	Folin Lowry	0.60	0.65	0.86
Appearance	Turbid, white to yellow, even suspension, easily redispersed	Ph. Eur. 20920	Pass	Pass	Pass
pH	To be reported	Ph. Eur. 2.2.3	7.3	7.4	7.5
**II. Final product**					
**Parameter**	**Specification**	**Method**	**FMOX1102**	**FMOX1105**	**FMOX1107**
Aluminium	< 1.25 mg/ml	Ph. Eur 0153	1.0	1.1	1.0
Endotoxin	< 1 x 10^5^ IU/ml	Ph. Eur. 2.6.14	< 1 x 10^5^ IU/ml	<0.5 x 10^5^ IU/ml	< 1 x 10^5^ IU/ml
Identity	Verify detection of 70 kDa (FetA F3-3), Class 1 (P1.16) and Class 3 (P3.15) with antigen specific monoclonal antibodies	Ph. Eur. 2.2.31	Detected	Detected	Detected
pH	To be reported *	Ph. Eur. 2.2.3	6.1	5.9	6.1
Potency	To be reported **	In-house	2067 (1509–2839)	3027 (2239–4091)	2741 (1778–4224)
Pyrogenicity	Pass	Ph. Eur. 20608	Pass	Pass	Pass
Sterility	Pass	Ph. Eur. 20601	Pass	Pass	Pass
Appeareance	Opaque, even, milky	Ph. Eur. 20920	Pass	Pass	Pass
Extractable volume	≥0.50 mL	Ph. Eur. 2.9.17	Pass	Pass	Pass
Degree of adsorption	To be reported	Ph. Eur. 01503	>93%	>93%	>93%

*For comparison the pH of MenBvac has been in the range 5.5–7.0 (unpublished observations, NIPH).

** Data presented in Fig 6. The relative IgG antibody response against OMVs is here reported as Geometric mean titre (GMT) and 95% confidence intervals (Arbitrary Units of anti-OMV IgG/mL) in serum after 2 doses of 2.5 μg OMV protein 3 weeks apart, 2 weeks after last dose.

### Quality control testing of OMVs and adsorbed vaccine

The analytical methods used for quality control of the active pharmaceutical ingredient (OMV) and the vaccine final product (MenPF-1) are given in Table 1, along with acceptance criteria. The OMVs were characterised for their antigenic composition by 12.5% SDS-PAGE stained with Coomassie blue, as previously described [19], and compared with OMVs used as the active pharmaceutical ingredient in the Norwegian capsular group B vaccine MenBvac [17]. The relative content of proteins that were either present in high amount and/or regarded as relevant for immunological protection were quantified by densitometric digital scanning (BioRad), i.e. FetA, PorA and PorB. The LPS content of OMVs was quantified by densitometric scanning of silver stained tricine-SDS PAGE gels. The final vaccine product was tested for appearance, pyrogen content, protein content, potency (i.e. immunogenicity), endotoxin level, sterility, pH and degree of adsorption [18] (Table 2). In addition, the three first batches produced were tested for the abnormal toxicity test according to Ph. Eur 20609. A stability study evaluated if the OMV vaccine final product was able to meet the acceptance criteria at normal (5°C) and accelerated (25°C) conditions over 24 months (Table 3).

**Table 3:** Stability of three batches of the final product evaluated at normal (5°C) at 24 months and accelerated (25°C) conditions at 6 months.

Test	Specification	FMOX1102		FMOX1105		FMOX1107	
		5°C	25°C	5°C	25°C	5°C	25°C
Aluminium	< 1.25 mg/ml	1.0	1.1	1.0	1.0	1.1	1.0
Endotoxin	< 1 x 105 IU/ml	< 1 x 10^5^ IU/ml	<0.5 x 10^5^ IU/ml	< 1 x 10^5^ IU/ml	< 1 x 10^5^ IU/ml	<0.5 x 10^5^ IU/ml	< 1 x 10^5^ IU/ml
pH	To be reported	6.1	5.9	6.1	6.1	5.9	6.1

Potency data are shown in Fig 6. Acceptance criteria for tests specific for identify (detection of 70 kDa (FetA F3-3), Class 1 (P1.16) and Class 3 (P3.15) proteins), pyrogenicity, sterility, appearance (opaque, even, milky) and extractable volume (≥0.50 mL) passed for all 3 batches, and degree of adsorption was >93% for all.

The ability of the batches to induce bactericidal antibodies was assessed by SBA on pools of sera from mice immunized with batch FMOX1102 on release (mice receiving either zero, 2.5 μg or 10 μg OMV), after 12 and 24 months stored at 5°C (both tested at 2.5 μg doses) and after 6 months stored at 25°C. Batch FMOX1107 was similarly tested after 24 months stored at 5°C.

### Potency testing and comparative immunogenicity in three mouse strains

Potency testing of 3 batches of the final vaccine product (FMOX1002, FMOX1005 and FMOX1007) was performed to assess the dose-response curve in mice. Groups of 10 female, inbred NIH Ola/Hsd mice (12–14 g) (Harlan, U.K.) were immunised sub-cutaneously with one of four dose levels (1, 2.5, 5 or 10 μg OMV protein) of OMV vaccine final product. In each experiment (one per batch), a negative control group (five mice) was immunized with a control vaccine consisting of aluminum hydroxide in sucrose solution (MOX Control). Two doses (each in 0.5 mL volume) were administered 3 weeks apart, with blood samples drawn 3 weeks after the second dose (day 42 after 1^st^ dose, d42). The antibody responses were assessed by use of OMV ELISA as previously described [19] using OMVs from GMP batches as the coating antigen. Recombinant PorA and FetA antigens for ELISA were provided by Hema Patel (NIBSC) and refolded from inclusion bodies using protocols described elsewhere [29]. Pools of sera were further analysed in SBA against the capsular group B vaccine strain panel as described in previous sections.

To assess the potential impact of strain variation on the immunological response, the immunogenicity of the final vaccine product was assessed in three different mouse strains, using laboratory scale OMVs prepared from the working seed lot of the vaccine strain SMenPF-1.2. Groups of 10 mice (female, 18–22 g) of three different mouse strains (NIH Ola/Hsd, Balb/c and C57/Black) were immunised subcutaneously with two doses of 2.5 μg OMV protein adsorbed to Al(OH)_3_ (1/10th of human dose) in an injection volume of 0.5 mL. Doses were given 3 weeks apart, and blood sampled 3 weeks after the second dose. Sera were analysed for IgG against vaccine strain OMVs or recombinant FetA protein (VR 3–3) by ELISA, including IgG2 subclass responses.

### Toxicological evaluation of final OMV vaccine

The non-clinical safety assessment of the final vaccine product was performed to assess its suitability for testing in a future phase I clinical trial. The GLP testing was performed under contract to Charles River, Edinburgh, United Kingdom according to Good Laboratory Practice (GLP), relevant EMA guidelines (CPMP/ICH/286/95, CPMP/SWP/465/95 and CHMP/SWP/488313/07) and to the U.K. Animal Welfare Act (2006). The testing was performed in one general toxicology study for local tolerance and repeated dose in rabbits. The test scheme was designed to support the clinical administration of up to three intramuscular doses of the MenPF-1 vaccine, either in 25 μg or 50 μg total protein dose levels (Table 4).

**Table 4:** Experimental design of the GLP toxicity study of MenPF-1 in New Zealand white rabbits.

	Main Study		Recovery		Test Item	Dosage (μg/dose)	Conc. (μg/mL)	Dose Volume (mL/dose)
Group No.	M	F	M	F				
1	1–3	10–12	19–21	28–30	MOX Control	0	0	0.5 mL
2	4–6	13–15	22–24	31–33	MenPF-1	25	50	0.5 mL
3	7–9	16–18	25–27	34–36	MenPF-1	50	50	2 x 0.5 mL

The objective of the toxicology study was therefore to determine the potential toxicity of vaccine when given by intramuscular injection on 4 occasions over a 9 week period to New Zealand white rabbits to evaluate the potential reversibility of any findings. The vaccine product evaluated in the GLP toxicity test (batch FMOX1102) had passed the same relevant specifications as for the GMP batch to be used in the clinical phase I trial (FMOX1107; Table 2). A placebo product was used as comparator in the study, containing a suspension of aluminum hydroxide (0.333% w/v) diluted in 3% sucrose in water for injection (MOX Control, lot FMOX1102). In total 36 rabbits (18 male and 18 female) were injected into a hind limb muscle on Days 1, 22, 43 and 64 with necropsy on Days 66 and 92. Of the 36 animals, 12 were treated with a control preparation containing the same adjuvant and excipients as the test vaccine. The following parameters and end points were evaluated: viability, clinical signs, injection site reactions, body weights, body weight changes, food consumption, ophthalmology, body temperatures, clinical pathology parameters (haematology, coagulation and clinical chemistry), antibody analysis, gross necropsy findings, organ weights and histopathological examinations (S1 Text).

### Statistical analyses

Differences in antibody levels or SBA titers levels between groups were analysed using non-parametric statistical tests using GraphPad Prism Version 5 (GraphPad Software, CA, U.S.). Correlations were assessed by the Spearman rank order correlation test. The statistical analyses in the GLP toxicology study were performed two-sided and at the 5% significance level. Male and female animals were analysed separately. Pairwise comparisons were performed against the control group. Body weight, food consumption, haematology, coagulation and clinical chemistry were analysed for homogeneity of variance using the ‘F Max' test, with specified procedures according to the homogeneity of the group variances involving use of parametric ANOVA, Fisher’s F protected LSD method via Student's t test, Kruskal-Wallis non-parametric ANOVA or chi squared protection. Organ weights, and organ weights as a percentage of terminal body weight, were analysed using ANOVA and by analysis of covariance (ANCOVA) using terminal kill body weight as covariate.

## Results

### Construction of vaccine strain and verification of suitability for OMV production

**Fig 3:**
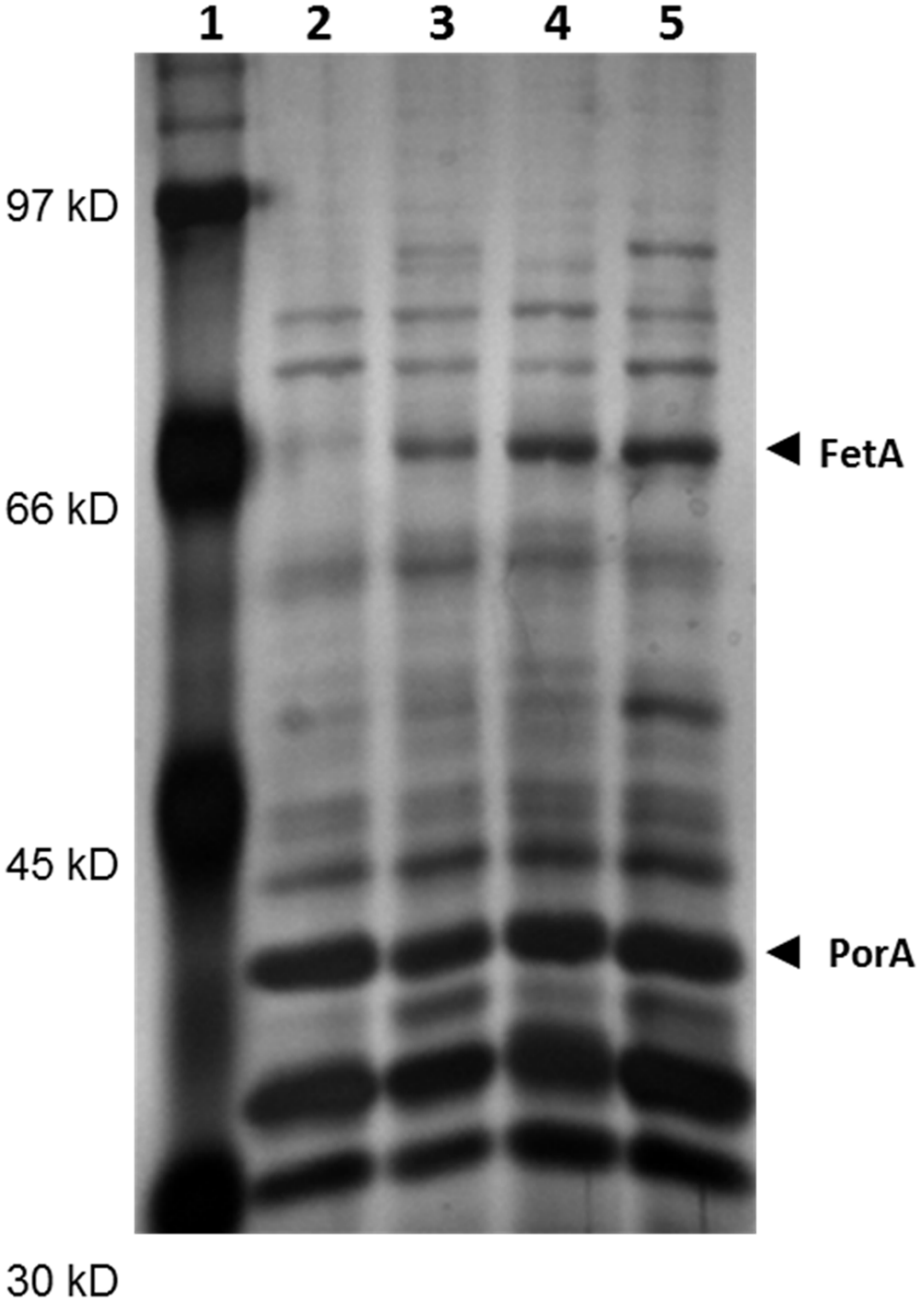
Effect of iron restriction and consecutive passages on FetA expression in OMVs from strain 3208, as analysed by gel electrophoresis of protein extracts. Silver stained resolving gel of OMVs prepared from the parent and transformants. Lanes: (adjusted to 10ug protein per well). Lane 1: BioRad pre-stained SDS broad-range standards. Lane 2: Wildtype (2851)–not iron restricted. Lane 3: Wildtype (2851)–iron restricted. Lane 4. Constitutive Strain (3207)–not iron restricted. Lane 5: Constitutive Strain (3207)–iron restricted.

A meningococcal strain was genetically modified to express the FetA protein, regardless of the amount of available iron, by replacing the wild-type promoter region of the fetA gene with a constitutive promoter containing a 17 bp spacer region between its -10 and -35 sequences. The strain used for this genetic modification expressed PorA at a high level that was retained after the modification of FetA expression (Fig 3).

When tested for stability of FetA expression in various growth media, it was found to appear independent of iron content in the growth medium as compared to the wild type strain 2851 (Fig 3). The vaccine construct was stable, as the mutant showed consistently upregulated FetA expression after 10 consecutive passages with or without kanamycin selection (data not shown). Furthermore, the strain showed good growth characteristics as measured by viability before and after processing into seed lots and growth in 1.5 L modified Frantz liquid medium (10^8^ to 10^9^ CFU/mL) (Fig 2), whilst retaining its expression of key antigens FetA, PorA, PorB and LPS.

Taken together these observations verified the suitability of SMenPF1.2 as a vaccine manufacturing strain. The cross flow filtration process showed that depending on the concentration of bacteria after incubation in shaking flasks, the bacterial suspension could be concentrated 8–15 times. Three GMP batches of OMVs were produced (FMOX1102, FMOX1105, FMOX1107) with mean protein concentration pre-formulation of 0.79 mg/mL (CV 5.2%) (Table 2).

### Characteristics and stability of the OMV vaccine

**Fig 4:**
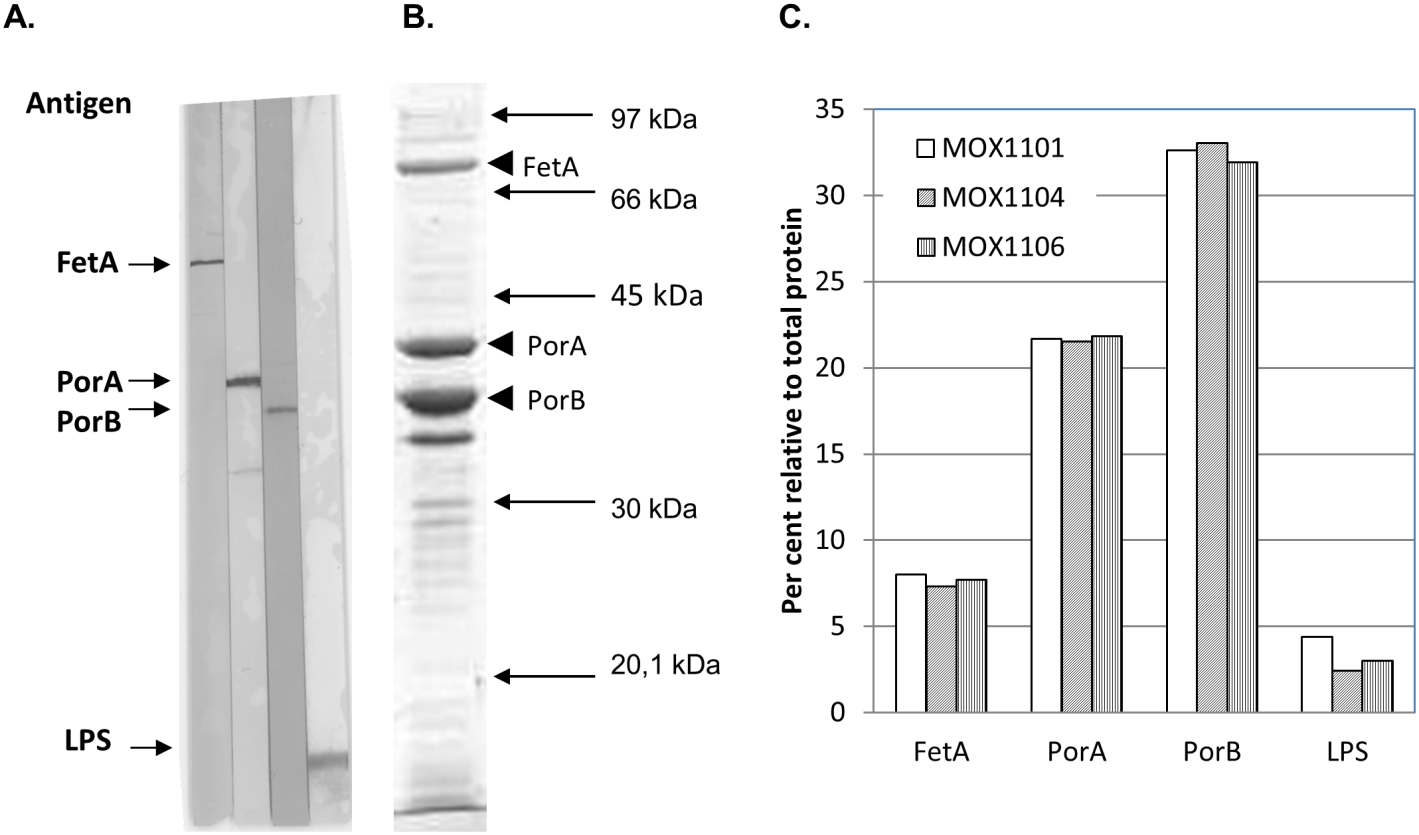
Antigen characterization. A. Binding of monoclonal antibodies (PorA P1.16 (01/538), PorB P3.15 (02/310) and LPS L3,7,9 (01/412)) and antisera (FetA F3-3 (12/03/2010)) to a whole cell preparation of a sample of SMenPF1.2 from a Frantz 7h liquid culture, separated by 12% SDS-PAGE and electrotransferred to nitrocellulose membrane. B. Antigen profile of the drug substance in the MenPF-1 vaccine (lot MOX1106), as determined by gel electrophoresis. Arrows indicate molecular weight standard (Amersham). C. Relative protein content of three batches of OMVs derived from vaccine strain SMenPF1.2.

Quality control data were generated for the three vaccine batches (Table 2). These demonstrated that FetA was elevated compared to iron replete conditions in the OMVs, and that this did not vary between batches (mean 7.9% FetA protein relative to total protein bands, CV 9.1%). The protein content of the vaccine was found to be consistent between the three batches of OMVs, with mean PorA content of 21.7% (CV 0.6%), and mean PorB content of 32.5% (CV 1.4%), whereas the LPS content relative to protein (acceptance criteria <12%) showed larger variation (range 2.4 to 4.4%, CV 26%) (Fig 4).

**Fig 5:**
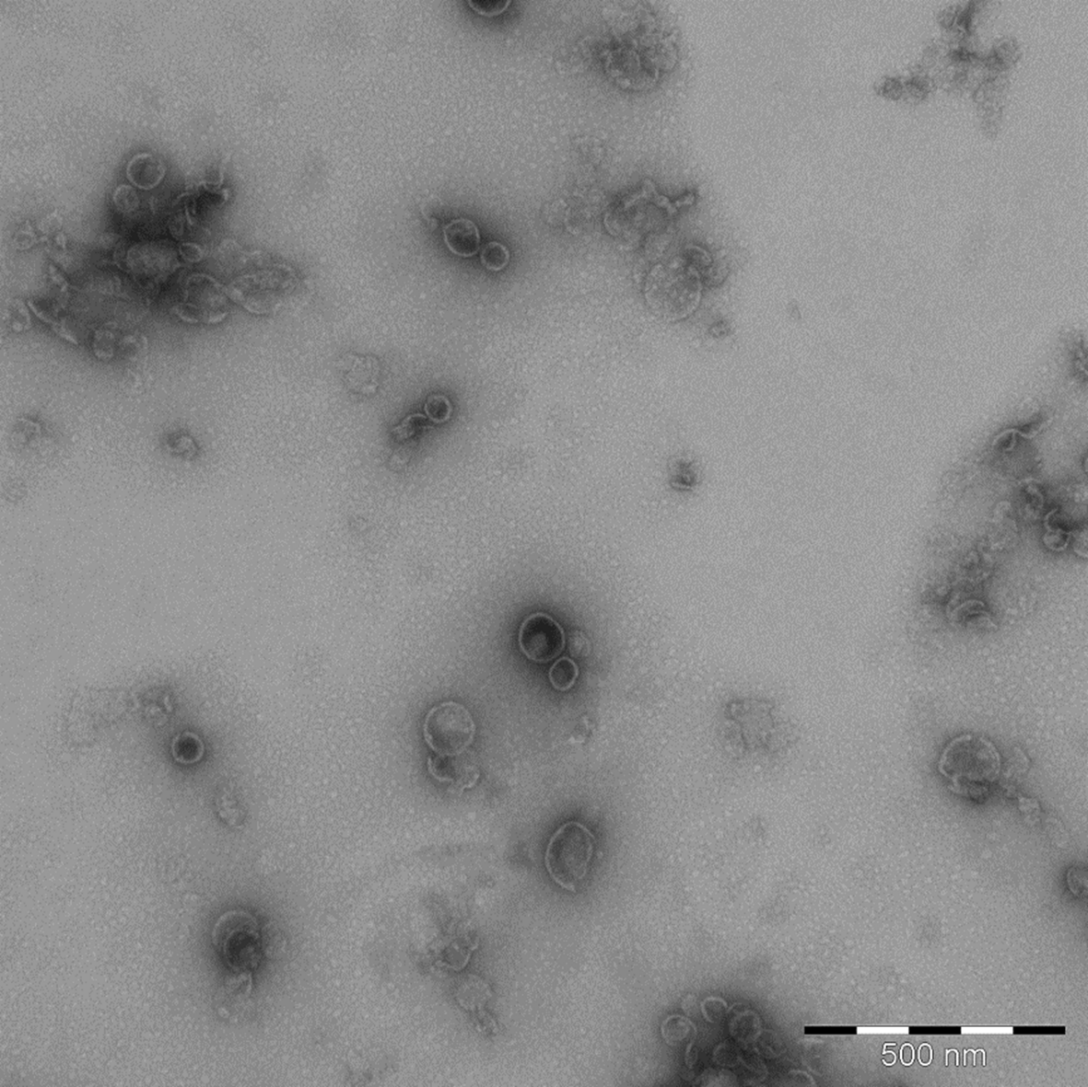
Morphological characterization. An example of bulk OMVs (batch MOX1106) derived from Neisseria meningitidis strain SMenPF1.2 visualized by negative staining and transmission electron microscopy (size bar is 500 nm). Courtesy of Sverre-Henning Brorson, Department of Pathology, University of Oslo, Oslo, Norway.

Electron microscopy (EM) analysis to provide morphological characterization was not part of the acceptance criteria, and was performed on one bulk OMV batch (MOX1106) as the 3 batches were otherwise comparable (Table 2). EM demonstrated the presence of both intact and fragmented vesicles, with intact vesicles in the size range of approximately 40–140 nm (Fig 5).

Stability studies verified that the acceptance criteria could be met after 24 months of storage at normal conditions (5°C), and at accelerated conditions (25°C) for up to 6 months (Table 3).

### Immunogenicity evaluation in three mouse strains and in potency studies

**Fig 6:**
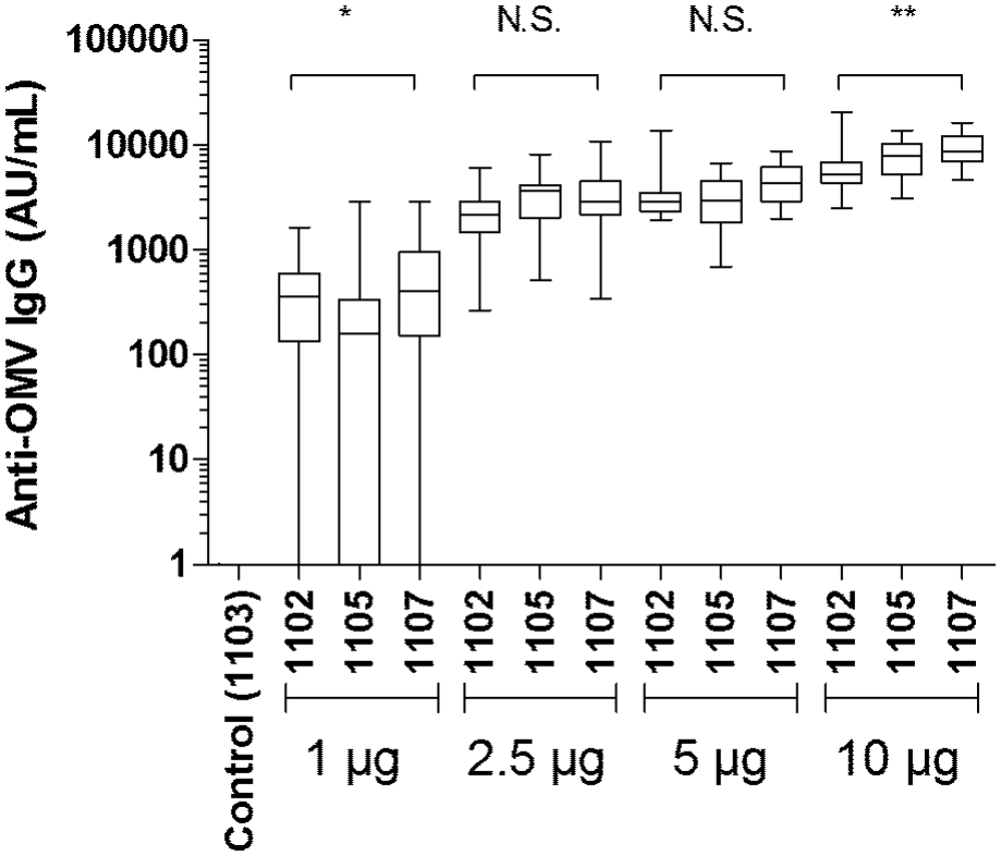
Dose response immunogenicity. Potency dose response curve for three different batches of final OMV vaccine product (FMOX1102, FMOX1105 and FMOX1107), as measured by OMV specific IgG ELISA. Statistically significant differences were detected between batches at 1 μg (*) and 10 μg (**) dose levels respectively but not for other dose levels.

The potency studies showed that the vaccine elicited dose-responsive OMV specific antibodies (Fig 6).

**Fig 7:**
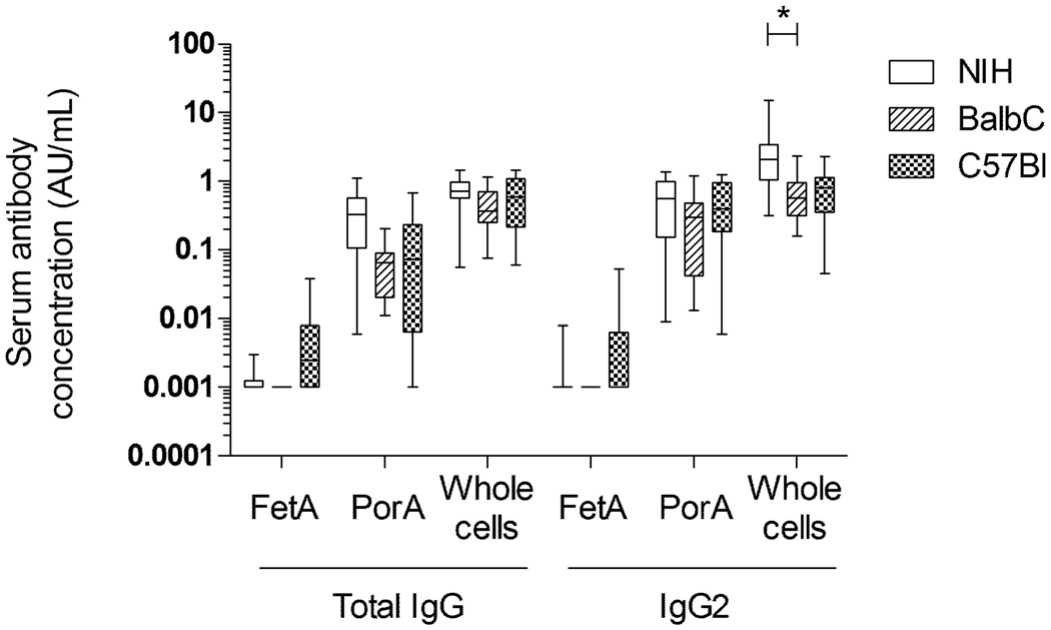
Comparative immunogenicity between mouse species. Immunogenicity in three mouse species (NIH, Balb/c, C57/Black), analyzed by ELISA measuring total IgG and IgG2 against rFetA, rPorA, or whole cells (WC) of the vaccine strain 3207 (constitutive FetA expression and wt PorA expression). Two doses of OMVs (2.5ug protein/dose with Al(OH)3 adjuvant) were given to mice s.c. 3 weeks apart, and these were bled 3 weeks after the final dose. N.S.; not significantly different. *; significant difference detected.

A comparison of the immunogenicity in three inbred mouse strains (NIH, Balb/c and C57/Black) with two doses of 2.5 μg OMV protein (1/10th of the human dose) showed that there was no significant difference between the three mouse strains in their IgG response. Antibody responses with this dose level induced IgG against whole cells of the vaccine strain, recombinant PorA and FetA (Fig 7). There were no statistically significant differences between mouse strains, apart from the IgG2 response against whole cells being higher in NIH mice than in Balb/c and C57/Black mice.

### Product characteristics upon release and following storage for 24 months

**Fig 8:**
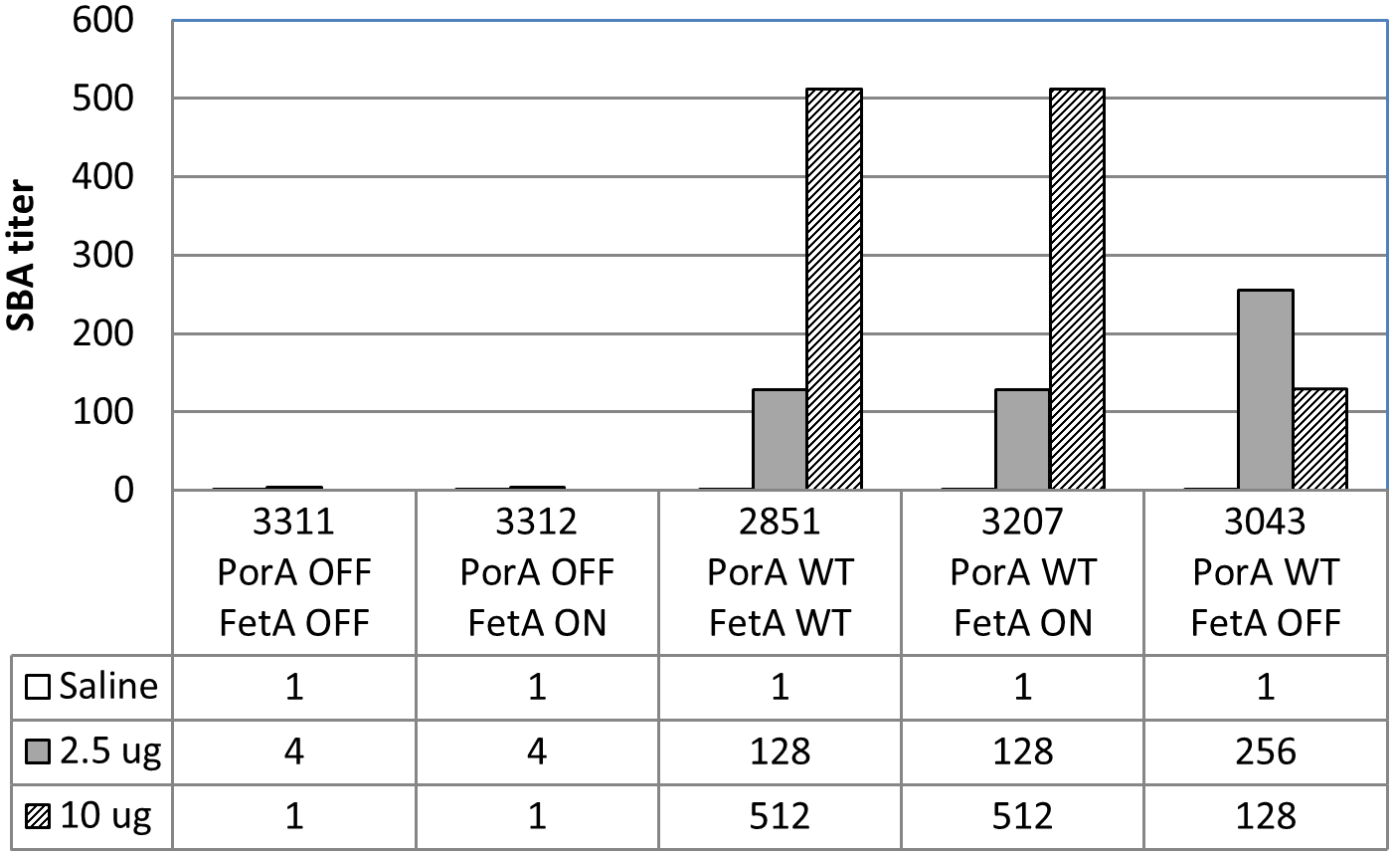
SBA against the target strain panel in serum pools from mice immunised with 0, 2.5 μg or 10 μg OMV protein, measured using 25% human complement.

**Fig 9:**
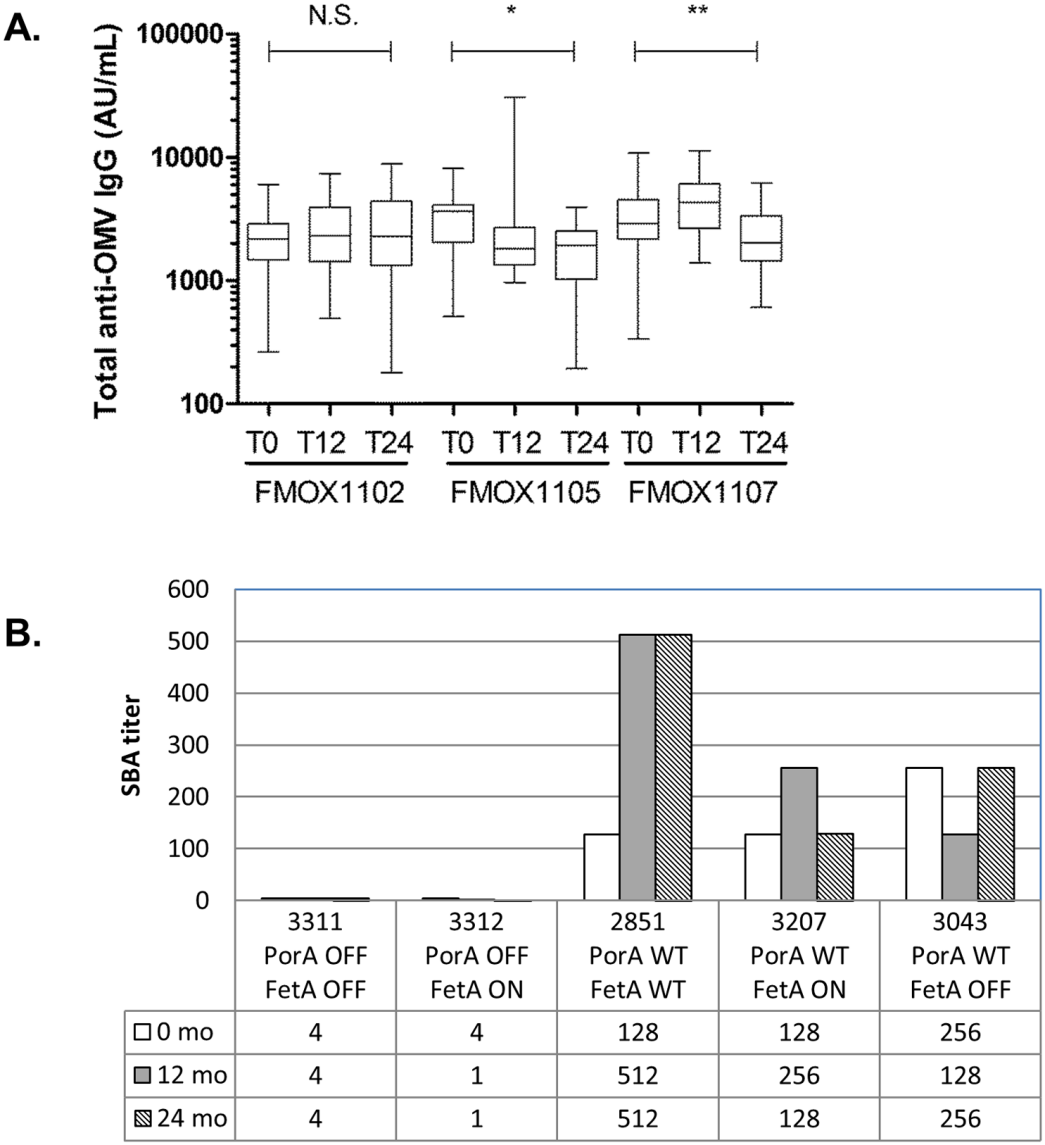
Impact of storage on vaccine immunogenicity. A. Stability of MenPF-1 final vaccine (lot FMOX1102, FMOX1105 and FMOX1107), measured as potency at release, 12 months and 24 months of storage at normal temperature (5°C). Potency, as measured by immunogenicity of a 2.5 μg OMV protein dose in mice female, inbred NIH Ola/Hsd mice, 12–14 g. Statistically significant differences between timepoints were observed for batches FMOX1105 (*) and FMOX1107(**). B. SBA against the target strain panel in serum pools from mice immunised with MenPF-1 vaccine (batch MOX1102) on release (0), after 12 months storage (12 mo) or after 24 months storage (24 mo) at 5°C.

Stability testing included two dose levels; 1 and 2.5 μg protein per dose. For all three batches (FMOX1102, FMOX 1105 and FMOX 1107), samples were stored for 24 months at 5°C, and for 6 months at 25°C. All tests yielded results that met the release specification requirements. No significant change was observed for pH in this time period (Table 3), and potency remained high for all 3 batches over 24 months (Figs 8 and 9). However, some reduction in potency was observed for batches FMOX1105 between release to 12 and 24 months (GMT fell from 3027 to 2141 to 1539, respectively) and FMOX1107 between 12 and 24 months (GMT fell from 4075 to 2215) (Fig 9).

The adsorption to adjuvant was consistent during the stability study, indicating that the predicted immunological activity was also stable for 24 months.

### Toxicological evaluation

Results from the evaluation of potential toxicity and reversibility of reactions to the vaccine MenPF-1 (lot FMOX1102) when given by intramuscular injection for 4 occasions over a 9-week period to New Zealand White rabbits showed that there were no unscheduled deaths during the observation period (S1 Text). There were no systemic signs and no local irritation noted in any animal during the observation period. Body weight and food consumption profiles were unaffected by treatment and there were no eye changes that were considered to be treatment related. There were no differences in body temperatures recorded up to 48 h after injection with MenPF-1. Other than higher neutrophil numbers, higher fibrinogen levels and minor disturbances in plasma proteins at Day 64 in animals that received MenPF-1, when compared with controls, there were no in-life findings that were considered to be related to treatment with the vaccine.

At Day 66 (2 days after the last injection), 50 μg/dose of MenPF-1 resulted in minor findings at the injection site with foreign material-laden macrophages and giant cells noted. Polymorphonuclear and mononuclear inflammation with myofibre necrosis and/or regeneration, interstitial fibrosis and/or mineralisation were also observed upon necropsy. Lumbar lymph node enlargement was observed at necropsy and this correlated with lymphoid hyperplasia. Accumulation of foreign material-laden macrophages and giant cells was also noted in the lumbar lymph nodes. After a 4 week recovery period, a number of findings persisted in treated injection sites; however, these were of a lesser severity and frequency. There were no differences in organ weight that were considered to be related to MenPF-1.

### Whole genome sequencing for assessing impact of genetic modifications on vaccine and evaluation strains

Comparative genomics of the draft genomes of the four strains used for vaccine production and serological evaluation of the MenPF-1 vaccine (Table 5, S1 Fig) and the parent strain showed that they were identical to each other at 1584 loci and differed at just six.

**Table 5:** Genome Comparator analysis of isogenic strains.

**Locus**	Gene product	Strain identity						Specification of differences
		H44/76 (Norwegian)	2851 (wild type)	3043 (porA+ fetA-)	3207 (SMenPF1.2)	3311 (porA- fetA-)	3312 (porA- fetA+)	
PorA ^a^	PorA variable regions	P1.7,16	P1.7,16	P1.7,16	P1.7,16	P1.7,16	P1.7,16	
FetA ^a^	FetA variable region	F3-3	F3-3	F1-5	F3-3	F1-5	F3-3	pEAT2 plasmid insert sequence contains F1-5VR
NEIS0276	putative rotamase	147	100	100	100	100	100	18bp tandem repeat ‘TGCCAAAGTTGCCAAAGT’ allele-147, 2x allele-100 3x
NEIS0308	hypothetical protein	14	16	16	16	16	16	1bp indel (allele-14 internal stop)
NEIS0568 (pglE)	glycosyl transferase	47	178	178	178	178	178	7bp tandem repeat ‘CAAACAA’ allele-178 12x, allele-47 23x (phase variable OFF)
NEIS0577	putative iron-uptake permease inner membrane protein	1	1	1	1	80	1	1bp indel (allele-80 internal stop)
NEIS0771	cytosine-specific DNA methyltransferase	1	1	1	1	1	17	1bp indel (allele-17 internal stop)
NEIS1310	type III restriction/modification system modification methylase	179	Off end of contig	254	179	254	179	4bp tandem repeat ‘AGCC’ allele-179 18x (internal stop-phase-variable OFF), allele-254 19x (ON), 2581(wt) 20x (OFF)
NEIS1418	putative membrane peptidase	4	4	4	87	87	4	240bp indel and bp diff
NEIS1646	glycyl aminopeptidase	1bp del	4	4	4	4	4	1bp indel (H44/76 internal stop)
NEIS1998	phosphopantetheine adenylyltransferase	1bp del	4	4	4	4	4	1bp indel (H44/76 out of frame)
NEIS0210 (pilE)	pilE protein	20	442	off end of contig	443	440	444	

^a^ Listed are the two variable regions of the two genetically modified genes porA and fetA and the loci where there were sequence differences.

The first three locus differences were: a putative iron-uptake permease inner membrane protein (locus NEIS0577); a cytosine-specific DNA methyltransferase (locus NEIS0771); a putative membrane peptidase (locus NEIS1418). There was a single base pair difference in each of the first two loci and a 240bp indel, and one base pair difference in the latter. The fourth locus difference was at the NEIS1310 locus also known as modA the methylase modification gene of the Neisseria type III restriction/modification system [30]. The presence of a certain number of the tandem repeat ‘AGCC’ can render the gene in frame (phase-variable ON) and out of frame (phase-variable OFF). The two FetA-negative strains 3043 and 3311 were phase variable ON. The highly variable pilE gene (locus NEIS0210) also differed amongst all strains tested including these four. This was mainly due to variation in the C-terminal D region of the gene; the class I type pilin in the case of these strains [31]. The pilS cassette region adjacent to the pilE locus in each genome was the same in 3043, 3311 and for the fragment that was present at the end of a contig for 3312 but differed by 21 bp and 7 bp in strains 2851 and 3207 respectively in the fragments of contig that were aligned to the pilS cassette region allele 29. The pilE alleles in each strain were possibly the result of recombination with different pilS cassettes: 2851 and 3207 with cassettes 19 bp and 5 bp different to the pilS allele 5 from Helm and Seifert retrieved from Genbank [32]. These pilS donor sequences were present unchanged in the fragment of the pilS cassette region in their respective genomes. The resulting pilE alleles in strains 2851 and 3207 were atypical with an extended C terminal which may result in a non-functional pilin subunit [31]. The remaining strains were putatively the result of recombination of pilS cassette alleles 2 and 5 and possibly others. The FetA VR also differed in two of the strains (F1-5 instead of F3-3) but this was due to the use of the plasmid pEAT2 [3] in the preparation of the FetA knockouts which had the F1-5 VR. Remapping sequence reads using Bowtie and visualization using Tablet showed lower read depth than surrounding regions of the genome in all of these loci apart from pilE. Therefore, caution must be exercised when interpreting changes in poly nucleotide tracts and tandem and complex repeat regions, features commonly associated with phase-variable and complex genes, from draft genomes assembled from short-read data.

## Discussion

### Development and evaluation of the vaccine strain SMenPF1.2

The development of a capsular group B meningococcal vaccine with constitutive FetA expression was made possible by genetic modification of strain H44/76. Phenotypic characterisation of the vaccine strain showed it to have a constitutive expression of FetA of ~7% of protein, comparable to the highest level seen in OMVs derived from the wild-type strain H44/76. The fetA gene is regulated by its native, iron-repressible promoter in wild-type H44/76 and consequently FetA represents anything from one to eight percent of the total protein in wtOMV vaccines depending on culture conditions [16]. It is not clear if 7% is the maximum expression level that can be stably attained for FetA in meningococcal outer membranes. Previous data indicate that FetA expression can be at similar levels to those seen for PorA [10] and other mechanisms for regulating FetA expression may achieve higher expression levels. Nevertheless, the stable constitutive of expression of FetA was compatible with the use of the SMenPF1.2 strain to manufacture a vaccine.

### Genomic effects of transformation of the vaccine strain and the evaluation panel strains

The suitability of the vaccine evaluation strains to assess FetA and PorA-specific immunity was verified in SBA against the 4 isogenic strains of the evaluation panel (Fig 9). Whole genome sequencing of the vaccine and evaluation strain panel demonstrated that the genetic modifications also included changes outside of the PorA and FetA genes; hence, it is possible that some bactericidal effects may not be attributable to the altered PorA and FetA expression levels alone. The majority of differences were single base indels or tandem repeat differences associated with phase variation; however, many of these differences could be the result of sequencing and/or assembly errors due to the nature of these complex and/or repetitive regions. The two fetA knock-outs were phase variable ON for the type III restriction/modification system modification methylase [30] which may indicate some knock-on response to the genetic manipulation of the fetA gene. The highly variable class I pilin gene pilE was different in each of the isogenic strains in this study, underlining its ability to rapidly undergo antigenic diversification. In a global collection of 219 isolates, a different class I sequence was found in each isolate [31]. This exceptional ability to diversify is mediated in individual strains through unidirectional homologous recombination with multiple pilS cassettes located close to the pilE locus and was demonstrated here [32] [33]. In summary, genome sequencing allowed for a high-resolution investigation of genome changes that may have occurred as a result of genetic manipulation and demonstrated that the isogenic strains showed remarkably little variation and were essentially identical. The panel of strains was therefore appropriate to elucidate the respective contributions to bactericidal activity of PorA and FetA specific antibodies.

### Manufacturing process of MenB OMV vaccines—lessons learnt

The small scale manufacturing process for OMV production developed in this study resulted in a consistent product, as indicated by quality control analyses of critical parameters. A stable production process was established, which satisfied both GMP standards and resulted in a sufficient production yield for testing OMV vaccines in phase I and II clinical trials. Advantages with the production methods employed were that they were simple, reproducible, and compatible with criteria for safe handling of genetically modified organisms (GMOs) [18]. The protein yield in in this small scale process (mean 0.79 mg protein per L growth medium) was lower than observed for other OMV vaccine processes. Factors affecting the yield probably included the critical micelle concentration (detergent concentration) affecting the ability of the OMVs to pass the sterility filter. The hydrophobicity and electric charge exerted by the CFF membrane, as well as the salt concentration of the buffer (zeta potential) were parameters likely to be connected with the dispersion and formation of vesicles [34]. Despite differences in the production processes in Norway, The Netherlands, and Cuba [17,18,35], the various OMV vaccines produced in these countries have similar safety profiles [36], whereas their ability to induce SBA may differ. The latter may be related to various factors, including the target strain used in the SBA assay and the relative amount of LPS in the vaccine; a potent immune stimulator. The current 9 litre-scale process was used to manufacture three batches of vaccine, each of sufficient yield and containing a consistent amount of the PorA and FetA antigens. Each batch contained a similar amount of LPS to other OMV vaccines and met Ph. Eur requirements for endotoxin content (<10^5^ IU/mL as measured by LAL gel clot assay).

### Immunogenicity and stability

The final product was highly immunogenic in potency tests in NIH mice, demonstrating a dose-response curve (Fig 8). Comparative immunogenicity across three mouse strains showed a strong response measured in ELISA against PorA and no significant response against FetA (Figs 8 and 9). This discrepancy between PorA and FetA responses was consistent with the fact that, within the administered dose of 2.5 μg total OMV protein, approximately 0.54 μg (21.7%) of this was PorA and 0.19 μg (7.7%) was FetA. Bactericidal antibody testing of serum pools from mice immunized as part of potency testing in batch release and stability testing showed that there was practically no contribution of FetA to the bactericidal activity observed (Figs 8 and 9). The immunogenicity of both PorA and FetA has been well documented in previous studies, and in particular the role of IgG against PorA in inducing protective antibodies in humans. This latter aspect is not as well characterised for FetA, and will be important to establish in a phase I clinical trial with the MenPF-1 vaccine. The vaccine evaluation panel described in this study will be instrumental to delineate the ability of anti-FetA IgG to mediate bactericidal activity.

The product was stable, meeting the same identity and purity specifications after 24 months as upon release. Although ELISA-based potency results varied during the observation period (Fig 9), results from SBA performed on pools of sera from mice immunized with MenPF-1 batches stored for up to 24 months did not suggest any decline in the functional antibody effect (Table 3 and Fig 9). Previous studies indicate that aluminium-adjuvanted deoxycholate extracted OMV vaccines are stable for at least 1 year [18,37], and the shelf-life of the MenPF-1 vaccine was consistent with this.

### Toxicological evaluation of the MenPF-1 OMV vaccine

The toxicological profiling of rabbits exposed to 4 intramuscular injections of the MenPF-1 vaccine (i.e. one more than typically administered with the MeNZB and MenBvac OMV vaccines in adults) showed inflammation at the injection site, as well as lymph node enlargement and other signs indicative of an inflammatory response (S1 Text). This was in line with treatment-related findings typically seen in repeat-dose studies with parenterally administered vaccines, as recently reviewed [38]. Changes observed in this type of studies are typically not severe and are transient. The strong anti-OMV IgG response mounted following administration of MenPF-1 indicates the relevancy of the rabbit model and the doses used for assessing toxicology. The findings are also consistent with previous experience using aluminium adjuvanted vaccines, with expected presence of local inflammation. In addition to boosting the immunological response, another role of the adjuvant for OMVs is to adsorb the free LPS and hence reduce the toxicity of the dOMVs. Ultimately, the vaccine was considered to be well tolerated, if moderately reactogenic, and satisfied the E.U. requirements for testing in a phase I clinical trial [39].

### Future role of OMV vaccines in prevention of meningococcal disease

Major strengths of using detergent-extracted OMVs from wild-type meningococci as vaccine formulations are that they: (i) are immunogenic and protective, inducing functional antibodies, and (ii) have an established track record of safe effective use in humans. Recent data on the safety and efficacy of the MeNZB OMV vaccines in clinical use in New Zealand supports our manufacturing process as a platform technology for vaccine development [36]. MenB vaccine development has in the last decade focused on recombinant OMPs and native OMV vaccines; however, recombinant proteins were found to be more immunogenic when combined with a dOMV [35]. The recently licensed four component vaccine (4CMenB), consists of similar OMVs to the MeNZB vaccine combined with three recombinant protein antigens identified from meningococcal genome sequence data. Although the performance of a novel MenB vaccine to a large extent can be estimated based on epidemiological mapping, a clinical trial of the MenPF-1 OMV vaccine should be pursued to enable assessment of FetA specific responses in humans.

### The MenPF vaccine concept

The rationale underpinning the MenPF vaccine is that epidemiological data indicate that the meningococcal PorA and FetA antigens exhibit repertoire structuring and diversity consistent with their generating a protective human immune response in vivo [8]. Although the extensive repertoire of these antigens presents an apparent problem for the design of a broadly cross-protective vaccine that include these antigens, the repertoire structuring apparent in natural population of meningococcal is such a that a multivalent vaccine consisting of relatively few variants has the potential to offer a high level of coverage of invasive meningococci [7]. This approach contrasts with the development of the currently licensed recombinant protein-based vaccines (4CMenB and the RP2086 fHbp), in which the antigenic components were chosen from a single untypical isolate and subsequently assessed for their ability to elicit cross-reactive antibodies.

The current study was a first step in the proof-of-concept for the MenPF approach to the prevention of meningococcal disease. We were not able to define a separate contribution of FetA-specific antibodies to the bactericidal response to the vaccine through the immunogenicity studies here described. This could be further studied by assessing other target strains in SBA with a heterologous PorA, controlling for the variable wt FetA expression if possible. Secondly adsorption of sera with rFetA could prove useful. Finally, the bactericidal effect of FetA antibodies could be part of a synergistic SBA effect and be masked by other antibodies in combination in the SBA. A separate study demonstrated that one to four immunizations of mice with 2.5 μg protein per dose induced a small but significantly higher SBA GMT against the vaccine strain SMenPF1.2 compared to against the strain 3043 (FetA off, PorA wt) when assessed using rabbit complement, but not with human complement [Sanders et al. Manuscript PONE-D-14-43371, in revision]. The same study further showed that human complement SBA GMTs were higher against the vaccine strain SMenPF1.2 than against the FetA wild type strain H44/76; the latter expressing a lower level of FetA. The current study has demonstrated that the MenPF-1 vaccine likely is suitably safe for testing in a clinical phase I trial, and the study by Sanders et al. that FetA specific antibodies induced following vaccination may have a small but significant role in conferring bactericidal antibodies following MenPF vaccination. On the basis of previous studies with the MenBvac OMV vaccine [35], a high seroconversion rate with anti-PorA IgG induced will likely be observed with the MenPF-1 vaccine in a clinical trial. A key focus will however be to study the relative contribution of FetA antibodies to the induction of protective immunity. We have recently proceeded to evaluate this vaccine in phase I human studies with the data supporting the safety and immunogenicity of this approach, and demonstrating the induction of FetA bactericidal antibodies in humans [40] Further studies are required to investigate the species-specific differences of the ability of FetA to induce bactericidal antibodies, and to which extent the FetA dose level and source of complement affects this evaluation.

## Supporting Information

S1 Fig Genomic differences. Graphical map of H44/76 circular genome, with labels indication genomic positions of loci differing between the vaccine strain SMenPF1.2 and the evaluation strains 3043, 3311 and 3312.
(DOCX)

S1 Text GLP study report. Toxicity evaluation of the vaccine MenPF-1 (lot FMOX1102) when given by intramuscular injection for 4 occasions over a 9-week period to New Zealand White rabbits.
(PDF)

S1 Table Supplemental data. Raw data from meningococcal culture, antigenic characterisation of vaccine preparations or sera from mice immunised with OMV vaccine.
(XLSX)
