# Supplementary material for: An OMV Vaccine Derived from a Capsular Group B Meningococcus with Constitutive FetA Expression: Preclinical Evaluation of Immunogenicity and Toxicity
Source: PLoS One. 2015 Sep 21;10(9):e0134353. doi: 10.1371/journal.pone.0134353 (PMC4577077; doi:10.1371/journal.pone.0134353)

**S1 Fig.** Graphical map of H44/76 circular genome, with labels indication genomic positions of loci differing between the vaccine strain SmenPF1.2 and the evaluation strains 3043, 3311 and 3312.
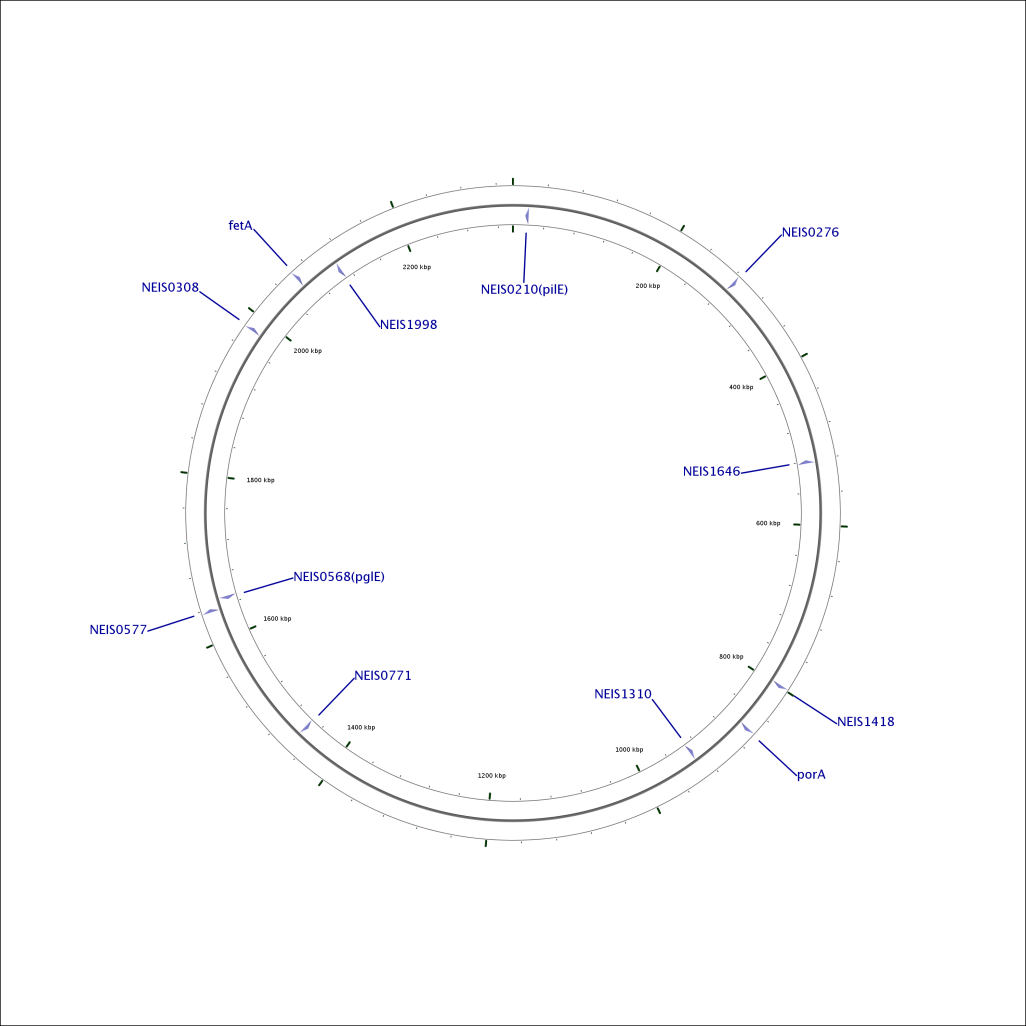

Supplement: S1 Fig — Graphical map of H44/76 circular genome, with labels indication genomic positions of loci differing between the vaccine strain SMenPF1.2 and the evaluation strains 3043, 3311 and 3312. (DOCX) [file pone.0134353.s001.docx]
