# Supplementary material for: An OMV Vaccine Derived from a Capsular Group B Meningococcus with Constitutive FetA Expression: Preclinical Evaluation of Immunogenicity and Toxicity
Source: PLoS One. 2015 Sep 21;10(9):e0134353. doi: 10.1371/journal.pone.0134353 (PMC4577077; doi:10.1371/journal.pone.0134353)
Supplement: S1 Text — Toxicity evaluation of the vaccine MenPF-1 (lot FMOX1102) when given by intramuscular injection for 4 occasions over a 9-week period to New Zealand White rabbits. (PDF) [file pone.0134353.s002.pdf]

FINAL

Test Facility Study No. 520419

A 9 Week Study of MenPF-1 Vaccine by Intramuscular Injection in Rabbits with a 4 Week Recovery Period

SPONSOR:
Oxford Vaccine Group
Department of Paediatrics
University of Oxford
Room 02-46-07
Children's Hosptial
Oxford, OX3 9DU
UK

TEST FACILITY:
Charles River Laboratories
Preclinical Services, Tranent (PCS-EDI)
Edinburgh
EH33 2NE
UK

22 February 2012

TABLE OF CONTENTS

1. LIST OF FIGURES 4
2. LIST OF TABLES 5
3. LIST OF APPENDICES 6
4. COMPLIANCE STATEMENT 7
5. QUALITY ASSURANCE STATEMENT 8
6. RESPONSIBLE PERSONNEL 9
6.1. Test Facility 9
6.2. Test Facility Individual Scientists (IS) 9
6.3. Sponsor-designated Responsible Scientists 9
6.4. Sponsor 9
7. SUMMARY 10
8. INTRODUCTION 12
9. MATERIALS AND METHODS 13
9.1. Test Item 13
9.2. Control Items 13
9.3. Test and Control Item Characterisation 13
9.4. Reserve Samples 14
9.5. Test and Control Item Inventory and Disposition 14
9.6. Dose Formulation and Analysis 14
9.6.1. Dispensing of Control Item 14
9.6.2. Dispensing of Test Item 15
9.6.3. Sample Collection and Analysis 15
9.7. Test System 15
9.7.1. Species and Receipt 15
9.7.2. Justification for Test System and Number of Animals 15
9.7.3. Animal Identification 15
9.7.4. Environmental Acclimation 15
9.7.5. Selection, Assignment, and Replacement of Animals 16
9.7.6. Disposition 16
9.7.7. Husbandry 16
9.8. Veterinary Care 17
9.9. Experimental Design 17
9.9.1. Administration of Test and Control Items 17
9.9.2. Justification of Route and Dosage Levels 18
9.10. Definition of Day 18
9.11. In-life Procedures, Observations, and Measurements 18
9.11.1. Mortality/Moribundity Checks 18
9.11.2. Clinical Observations 18
9.11.3. Dermal Scoring 19
9.11.4. Body Weights 19
9.11.5. Food Consumption 19
9.11.6. Water Consumption 19
9.11.7. Ophthalmic Examinations 19

9.11.8. Body Temperature 20
9.12. Laboratory Evaluations 20
9.12.1. Clinical Pathology 20
9.13. Terminal Procedures 22
9.13.1. Unscheduled Deaths 22
9.13.2. Scheduled Euthanasia 22
9.13.3. Necropsy 22
9.13.4. Organ Weights 22
9.13.5. Tissue Collection and Preservation 23
9.13.6. Histology 23
9.13.7. Histopathology 24
9.13.8. Peer Review 24

10. COMPUTERISED SYSTEMS 24

11. STATISTICAL ANALYSIS 24

12. RETENTION OF RECORDS, SAMPLES, AND SPECIMENS 25

13. RESULTS 26
13.1. Mortality 26
13.2. Clinical Observations 26
13.3. Dermal Scoring 26
13.4. Body Weight and Body Weight Changes 26
13.5. Food Consumption 26
13.6. Ophthalmic Examinations 26
13.7. Body Temperature 27
13.8. Haematology 27
13.9. Coagulation 27
13.10. Clinical Chemistry 28
13.11. Antibody Analysis 28
13.12. Gross Pathology 28
13.12.1. Scheduled Euthanasia (Day 66) 28
13.12.2. Scheduled Euthanasia (Day 92) 28
13.13. Organ Weights 29
13.14. Histopathology 29
13.14.1. Scheduled Euthanasia (Day 66) 29
13.14.2. Scheduled Euthanasia (Day 92) 29

14. DISCUSSION 30

15. CONCLUSION 31

16. REFERENCES 32

1. LIST OF FIGURES

Figure 1 : Cage Plan 34
Figure 2 : Body Weights (kg): Group Mean Values 36

2. LIST OF TABLES

Table 1 : Body Weights with Change (kg): Group Mean Values: Treatment Period 39
Table 2 : Body Weights with Change (kg): Group Mean Values: Recovery Period 43
Table 3 : Food Consumption (g/animal/day): Group Mean Values: Treatment Period 45
Table 4 : Food Consumption (g/animal/day): Group Mean Values: Recovery Period 49
Table 5 : Haematology and Coagulation : Group Mean Values: Pretrial 51
Table 6 : Haematology and Coagulation : Group Mean Values: Day 66 55
Table 7 : Haematology and Coagulation: Group Mean Values: Day 92 59
Table 8 : Clinical Chemistry : Group Mean Values: Pretrial 63
Table 9 : Clinical Chemistry : Group Mean Values: Day 66 67
Table 10 : Clinical Chemistry : Group Mean Values: Day 92 71
Table 11 : Summary of Necropsy Findings: Day 66 75
Table 12 : Summary of Necropsy Findings: Day 92 77
Table 13 : Absolute Organ Weights (g) : Group Mean Values: Day 66 79
Table 14 : Organ Weights (Covariance Analysis): Group Mean Values: Day 66 83
Table 15 : Relative Organ Weights (% Body Weight): Group Mean Values: Day 66 87
Table 16 : Absolute Organ Weights (g) : Group Mean Values: Day 92 91
Table 17 : Organ Weights (Covariance Analysis): Group Mean Values: Day 92 95
Table 18 : Relative Organ Weights (% Body Weight): Group Mean Values: Day 92 99
Table 19 : Summary of Histological Findings: Day 66 103
Table 20 : Summary of Histological Findings: Day 92 117

3. LIST OF APPENDICES

Appendix 1 : Protocol, Amendments and Deviations 121
Appendix 2 : Certificates of Analysis for Test and Control Items 173
Appendix 3 : Individual Clinical Observations: Treatment Period 179
Appendix 4 : Individual Clinical Observations: Recovery Period 185
Appendix 5 : Injection Site Reaction Scores: Individual Findings 188
Appendix 6 : Body Weights with Change (kg): Individual Values: Treatment Period 190
Appendix 7 : Body Weights with Change (kg): Individual Values: Recovery Period 194
Appendix 8 : Food Consumption (g/animal/day): Individual Values: Treatment Period 196
Appendix 9 : Food Consumption (g/animal/day): Individual Values: Recovery Period 200
Appendix 10 : Individual Ophthalmoscopy Findings 202
Appendix 11 : Body Temperatures (°C): Individual Recordings 204
Appendix 12 : Methods, Units and Abbreviations Used for Laboratory Investigations 208
Appendix 13 : Haematology and Coagulation : Individual Values: Pretrial 216
Appendix 14 : Haematology and Coagulation: Individual Values: Day 66 220
Appendix 15 : Haematology and Coagulation : Individual Values: Day 92 224
Appendix 16 : Clinical Chemistry : Individual Values: Pretrial 228
Appendix 17 : Clinical Chemistry : Individual Values: Day 66 232
Appendix 18 : Clinical Chemistry : Individual Values: Day 92 236
Appendix 19 : Antibody Analysis 240
Appendix 20 : Individual Necropsy and Histological Findings: Day 66 255
Appendix 21 : Individual Necropsy and Histological Findings: Day 92 275
Appendix 22 : Absolute Organ Weights (g) : Individual Values: Day 66 288
Appendix 23 : Absolute Organ Weights (g) : Individual Values: Day 92 292
Appendix 24 : Relative Organ Weights (% Body Weights) : Individual Values: Day 66 296
Appendix 25 : Relative Organ Weights (% Body Weights) : Individual Values: Day 92 300
Appendix 26 : Pathology Report 304

4. COMPLIANCE STATEMENT

This study was performed in compliance with the following Good Laboratory Practice (GLP) regulations:

- The Organisation for Economic Co-operation and Development (OECD) Principles on Good Laboratory Practice (ENV/MC/CHEM(98)17).

Exceptions from the above regulations are listed below.

- Stability data are currently being generated and no formal expiry date for the test or control items were provided (see Section 9.3).

This study was conducted in accordance with the procedures described herein. All deviations authorised/acknowledged by the Study Director are documented in the study records. The report represents an accurate and complete record of the results obtained.

There were no deviations from the above regulations that affected the overall integrity of the study or the interpretation of the study results and conclusions.

The Test Site, National Institute of Biological Standards and Controls, is not in the UK GLP compliance programme, however the work has been monitored by Charles River personnel and is considered to be in compliance with the principles of GLP.

[signature]	22 FEB 2012.
Bruce Robertson, BSc<br>Study Director	Date

The test and control items were used as supplied and their production and subsequent analysis are outside the scope of this compliance statement.

5. QUALITY ASSURANCE STATEMENT

The Charles River Quality Assurance Unit conducted a protocol review, protocol amendment reviews, study-based inspections and report audits on this study, as detailed below.

Dates of QA Activity	Activity	Date of Report to Management and Study Director
20 Jul 2011	Facility Inspection	01 Aug 2011
09 Aug 2011	Protocol Review	09 August 2011
11 Aug 2011	Protocol Amendment 1 Review	11 August 2011
12 Aug 2011	Protocol Amendment 2 Review	12 August 2011
16-17 Aug 2011	Dose Dispensing	22 August 2011
17 Aug 2011	Dosing and Protocol Compliance	22 August 2011
05 Sep 2011	Protocol Amendment 3 Review	05 Sep 2011
23 Sep 2011	Protocol Amendment 4 Review	23 Sep 2011
21 Oct 2011	Necropsy	21 Oct 2011
16 Nov 2011	Antibody Bleeds	17 Nov 2011
21 Nov 2011	Protocol Amendment 5 Review	21 Nov 2011
22 Nov 2011	Sample Analysis	24 Nov 2011
28 Nov 2011	Protocol Amendment 6 Review	28 Nov 2011
04-12 Jan 2012	Draft Report Audit	12 Jan 2012
09 Feb 2012	Final Report Audit	09 Feb 2012

Process-based inspections relevant to this study are scheduled once every quarter. The outcome of each inspection is reported to Management and, where relevant, the Study Director.

Facilities relevant to this study are included in Charles River's annual facility inspection programme. The outcome of each inspection is reported to Management.

This report is considered to describe accurately and completely the procedures used in the study and the results obtained.

Caroline Garth BSc
Quality Assurance

22 February 2012
Date

6. RESPONSIBLE PERSONNEL

6.1. Test Facility

Study Director	Bruce Robertson, BSc<br>(Study Initiation-01 Sep 2011, 23 Sep 2011-Study completion)
	Elizabeth Donald, BSc<br>(02 Sep-22 Sep 2011)
Quality Assurance	Caroline Garth BSc
	Stewart Fraser BSc
Report Peer Review	Adam Woolley, MSc, DABT, FRCPath, ERT, CBiol, MSB
	ForthTox Limited

6.2. Test Facility Individual Scientists (IS)

Pathology	Lise Bertrand, DVM, MSc, DESV, DiplECVP
Peer Review Pathologist	Petrina Rogerson BMVS, MRCVS

6.3. Sponsor-designated Responsible Scientists

Antibody analysis	Caroline Vipond, PhD
	National Institute of Biological Standards and Control, Hertfordshire, UK

6.4. Sponsor

Sponsor Representative	Andrew J Pollard, FCRPCH, PhD
	Professor of Paediatric Infection & Immunity, Oxford Vaccine Group, Department of Paediatrics, University of Oxford, Room 02-46-07, Level 2, Children’s Hospital, Oxford, OX3 9DU

7. SUMMARY

This study evaluated the potential toxicity and reversibility of reactions to MenPF-1, a prophylactic vaccine for the prevention of infection from bacterial meningitis, when given by intramuscular injection for 4 occasions over a 9 week period to New Zealand White rabbits. In addition, immunogenicity was characterised.

Animals were treated into a hind limb muscle on Days 1, 22, 43 and 64 with necropsy on Days 66 and 92.

The study design was as follows:

Text Table 1
Experimental Design

Group No.	Animal Numbers				Test Item	Dosage (µg/dose)	Conc. (µg/mL)	Dose Volume (mL/dose)
	Main Study		Recovery					
	M	F	M	F				
1	1-3	10-12	19-21	28-30	MOX Control	0	0	0.5 mL
2	4-6	13-15	22-24	31-33	MenPF-1	25	50	0.5 mL
3	7-9	16-18	25-27	34-36	MenPF-1	50	50	2 x 0.5 mL

The following parameters and end points were evaluated in this study: viability, clinical signs, injection site reactions, body weights, body weight changes, food consumption, ophthalmology, body temperatures, clinical pathology parameters (haematology, coagulation and clinical chemistry), antibody analysis, gross necropsy findings, organ weights, and histopathological examinations.

There were no unscheduled deaths during the observation period.

There were no systemic signs and no local irritation noted in any animal during the observation period. Body weight and food consumption profiles were unaffected by treatment and there were no eye changes that were considered to be treatment related. There were no differences in body temperatures recorded up to 48 h after injection with MenPF-1.

Other than higher neutrophil numbers, higher fibrinogen levels and minor disturbances in plasma proteins at Day 64 in animals that received MenPF-1, when compared with controls, there were no in-life findings that were considered to be related to treatment with the vaccine.

An increase in titre of specific IgG was observed with increasing dose and over time. Following completion of 4 week treatment-free period, titres on Day 92 were similar or higher in the majority of animals to those recorded on Day 64.

At Day 66 (2 days after the last injection), 50 µg/dose of MenPF-1 resulted in minor findings at the injection site with foreign material-laden macrophages and giant cells noted. Polymorphonuclear and mononuclear inflammation with myofibre necrosis and/or regeneration, interstitial fibrosis and/or mineralisation were also observed. Lumbar lymph node enlargement was observed at necropsy and this correlated with lymphoid hyperplasia. Accumulation of foreign material-laden macrophages and giant cells was also noted in the lumbar lymph nodes. After a 4 week recovery period, a number of findings persisted in treated injection sites, however were of a lesser severity and frequency.

There were no differences in organ weight that were considered to be related to MenPF-1.

In conclusion, administration of the vaccine, MenPF-1, when given by intramuscular injection for 4 occasions over a 9 week period, was well tolerated in rabbits up to 50 µg/dose.

There was only an expected, minor inflammatory response which was associated with vaccine administration, characterised by macrophages, giant cells and polymorphonuclear and mononuclear inflammation at the injection sites with on-going recovery noted. There was no evidence of systemic toxicity.

8. INTRODUCTION

The objective of this study was to determine the potential toxicity of MenPF-1, a prophylactic vaccine for the prevention of infection from bacterial meningitis, when given by intramuscular injection for 4 occasions over a 9 week period to rabbits, and to evaluate the potential reversibility of any findings. Data will support the use of MenPF-1 in humans. In addition, immunogenicity was characterised.

The design of this study was based on the study objectives, the overall product development strategy for the test item, including the following study design guidelines:

- CPMP Note for Guidance on Preclinical Pharmacological and Toxicological Testing of Vaccines (CPMP/ICH/302/95), December 1997
- WHO guidelines on nonclinical evaluation of vaccines (WHO Technical report series No. 927, 2005)
- CPMP Note of Guidance on Non0Clinical Local Tolerance Testing of Medicinal Products (CPMP/SWP/2145/00), March 2001

The Study Director signed the protocol on 03 Aug 2011, and dosing was initiated on 17 Aug 2011. The in-life phase of the study was completed on 16 Nov 2011. The experimental start date was 10 Aug 2011, and the experimental completion date was 16 Dec 2011. The study protocol, protocol amendments, and deviations are presented in Appendix 1.

9. MATERIALS AND METHODS

9.1. Test Item

Identification:	MenPF-1
Batch (Lot) No.:	FMOX1102
Receipt Date:	07 July 2011
Expiration Date:	Concomitant assessment, ongoing
Physical Description:	Opaque, even milky suspension; easily redispersed
Purity:	The active pharmaceutical ingredient (API), formulated as outer membrane vesicles, is a mixture of *Neisseria meningitidis* serogroup B outer membrane proteins that shows >93% adsorption degree to aluminium hydroxide adjuvant. The API contains 8.0% 70kD FetA F3-3 variant protein, 21.7% Class 1 P1.7, 16 variant protein and 32.6% Class 3 P3.15 protein. The test item batch (i.e., vaccine product) contains 1.0 mg/mL aluminium. Dose calculations were not corrected for purity.
Concentration:	25 µg protein/dose of 0.5 mL
Storage Conditions:	In a refrigerator set to maintain 4°C
Supplier:	Norwegian Institute of Public Health, Oslo, Norway

9.2. Control Items

Identification:	MOX Control
Batch (Lot) No.:	FMOX1103
Expiration Date:	Concomitant assessment, ongoing
Physical Description:	Opaque, even milky suspension; easily redispersed
Purity:	The product contains the adjuvant, Alhydrogel; specifically containing 1.1 mg/mL aluminium. Dose calculations were not corrected for purity.
Concentration:	Nominal 0.333% w/v Alhydrogel in 3% sucrose solution
Storage Conditions:	In a refrigerator set to maintain 4°C
Supplier:	Norwegian Institute of Public Health, Oslo, Norway

Test and control items were monitored during transit to Charles River, Pre-Clinical Services, Edinburgh. Items were despatched refrigerated (2-8°C); an average temperature of 6.3°C was observed, with a high of 9.3°C which was maintained for 40 minutes. This deviation was considered to be transient and minor and not to have impacted on the integrity of the test and control items.

9.3. Test and Control Item Characterisation

The Sponsor provided to the Test Facility documentation of the identity, strength, purity, composition, and stability for the test and control items. A Certificate of Analysis for the vaccine, control item and the bulk vaccine from which the batch for this study was prepared, was provided to the Test Facility and these documents are presented in Appendix 2.

The vaccine, control item and the bulk vaccine from which the batch for this study was prepared were tested in accordance with Good Manufacturing Practice (GMP).

The Sponsor has appropriate documentation on file concerning the method of synthesis, fabrication or derivation of the test and control items, and this information is available to the appropriate regulatory agencies should it be requested.

No formal expiration dates were provided for the batches of MOX Control or MenPF-1 used on this study. The Sponsor indicated that batches of the control and test items were currently subject to stability testing. Data for the MenPF-1 batch including the latest available timepoint of 3 months were supplied, with further data being generated. The data indicated that at 3 months there was little difference between the results at this timepoint and the results at the initiation of testing. To provide stability data for the period of this preclinical study stability data of 4 months would be required, however, the current data suggested that there would be no reason to expect any degradation in MenPF-1 between 3 and 4 months.

For the MOX control item, current testing is underway, but no data were provided to the Test Facility. Given that this product has been subject to various testing before release, and as this is a control item, the lack of a definitive expiration date was considered not to have had any impact on the data generated from this study.

9.4. Reserve Samples

For each batch (lot) of test and control item, a reserve sample of one vial was retained under the appropriate storage conditions by the Test Facility.

9.5. Test and Control Item Inventory and Disposition

Records of the receipt, distribution, and storage of test and control items were maintained. With the exception of reserve samples, it is currently the intention to return all unused test and control items to the Sponsor after finalisation of the study report:

Andrew J Pollard, FRCPCH, PhD

Professor of Paediatric Infection & Immunity
Oxford Vaccine Group
Department of Paediatrics
University of Oxford
Room 02-46-07
Level 2, Children’s Hospital
Oxford, OX3 9DU
UK

9.6. Dose Formulation and Analysis

9.6.1. Dispensing of Control Item

The control item, MOX Control, was provided in single dose vials for administration to Group 1 control animals. No aliquoting of the control item was required. The vials were stored in a refrigerator set to maintain 4°C until use and on each day of injection an appropriate quantity was despatched to the animal unit for dosing. Details of the dispensing of the control item have been retained in the study records.

9.6.2. Dispensing of Test Item

The test item, MenPF-1, was also provided in single dose vials and no aliquoting of the test item was required. The vials were stored in a refrigerator set to maintain 4°C until use and on each day of injection an appropriate quantity was despatched to the animal unit for dosing. Details of the dispense of the test item have been retained in the study records.

9.6.3. Sample Collection and Analysis

The test and control items were used as received from the Sponsor, therefore, dose formulation analysis was not conducted at the Test Facility.

9.7. Test System

9.7.1. Species and Receipt

Eighteen male and 18 female New Zealand White rabbits were received from Harlan UK Ltd, Bicester, Oxon, UK on 02 August 2011.

On despatch, the animals were approximately 11-12 weeks old and weighed approximately 2.5 kg. At the start of treatment all animals were approximately 13-14 weeks old and weighed in the range of 2.6-2.8 kg for males while females weighed in the range of 2.7-3.1 kg (see protocol deviations in Appendix 1).

9.7.2. Justification for Test System and Number of Animals

The intramuscular route of administration was selected for this study as this route has been defined by the Sponsor as the route of clinical application/human exposure. The rabbit was selected by the Study Director in consultation with the Sponsor as the test model:

i. to satisfy regulatory requirements for toxicity testing

ii. because of the availability of background data in this species and proven suitability in toxicology studies

iii. because at this time, studies in laboratory animals provide the best available basis for extrapolation to humans and acceptable models which do not use live animals currently do not exist.

iv. because immunogenicity can be investigated in this species.

The numbers of animals chosen for this study was the smallest number considered necessary to provide sufficient data.

9.7.3. Animal Identification

Each animal received a unique ear tag which identified it individually within the study and which corresponded to that animal’s on-study number.

9.7.4. Environmental Acclimation

The animals were allowed to acclimate to the Charles River, Edinburgh rabbit toxicology accommodation for a period of 15 days before the first administration (see protocol deviations in Appendix 1).

9.7.5. Selection, Assignment, and Replacement of Animals

Animals were removed in a random order from their transport boxes and allocated to dose groups on arrival by placing them in separate cages. Cages were housed on racks according to treatment and labelled with the study, animal and group number. Control animals were housed on a separate rack.

A cage plan is presented in Figure 1.

During the week before commencement of dosing, the animals were approved for entry into the experiment on the basis of satisfactory clinical observation records and body weight profiles. There was no replacement of animals.

9.7.6. Disposition

All animals remained on-study until completion of in-life phases at which point designated animals were humanely euthanised by an intravenous overdose of a barbiturate. Details are retained in the study records.

9.7.7. Husbandry

9.7.7.1. Housing

Animals were housed individually in stainless steel cages (approximate dimensions 77 x 70 x 48 cm) with a 'Noryl' dual level interior, perforated floor, a mesh top, and a metal food hopper. Beneath each cage was a suspended tray containing absorbent paper. Paper was changed once a week.

Cages, cage racks, hoppers and bottles were changed weekly throughout the course of the study.

Animal room floors and work surfaces were washed daily with disinfectant solution. The ceiling, walls and all other surfaces within the animal room were washed weekly. Cage racks remained in the room throughout washing procedures.

9.7.7.2. Environmental Conditions

The environmental conditions are continually monitored and recorded every 15 min. Target ranges for temperature and humidity were 16-20°C and 40-85%, respectively, with a room air flow intended to give a minimum of 15 air changes per hour. From animal arrival, until study completion, the average daily ranges for temperature was 15-21°C and for humidity was 33-64% (see protocol deviations in Appendix 1).

Lighting was controlled to provide a 12 h light/dark cycle, normally being 0700-1900 hours.

9.7.7.3. Food

Harlan Irradiated Certified Global Rabbit Diet, supplied by Harlan, UK, was available *ad libitum* throughout the study. Each animal was also offered a supplement of hay at least 3 times per week.

Each batch of diet is routinely analysed by the supplier for various nutritional components and chemical and microbiological contaminants.

The results of the diet analysis did not provide evidence of contamination, and so did not prejudice the outcome of the study. Certificates of analysis for each batch used are retained at the Test Facility. The hay is not analysed.

9.7.7.4. Water

Water taken from the public supply (Scottish Water, Edinburgh, Midlothian, UK) was available *ad libitum* throughout the study.

The quality of water supply is stipulated by Water Quality (Scotland) Regulations 2001 and certificates of analysis for dissolved materials, heavy metals, pesticide residues, pH, nitrates, nitrites and selected bacteria are periodically provided. These analyses are based on water samples taken from these laboratories.

Results of water analysis did not provide evidence of contamination, and so did not prejudice the outcome of the study. Certificates of analysis relevant to the study are retained at the Test Facility.

9.7.7.5. Animal Enrichment

For environmental enrichment wooden chewsticks, produced by Datesand, Manchester, UK were placed in each cage and treats 'Bunny blocks' as supplied by William Lillico & Son Ltd, UK were also provided.

Analyses of these were considered to indicate that there were no additional substances in sufficient concentration to have any influence on the outcome of the study. Certificates of analysis for these items are retained at Charles River, Edinburgh.

9.8. Veterinary Care

All animals were under the care of Charles River's clinical veterinary surgeons, who were available at all times to provide advice and assistance.

On veterinary advice, a lesion to the right hind limb of Animal 27 (Group 3, Recovery Male), which resulted from clipping the injection site, was bathed twice a day for 4 consecutive days from Days 22-25 with aqueous chlorhexidine (an anti-septic). No further advice was required.

9.9. Experimental Design

Animals were treated on Days 1, 22, 43 and 64 with necropsy on Days 66 and 92. The Study design was as follows:

Text Table 2
Experimental Design

Group No.	Animal Numbers				Test Item	Dosage (µg/dose)	Conc. (µg/mL)	Dose Volume (mL/dose)
	Main Study		Recovery					
	M	F	M	F				
1	1-3	10-12	19-21	28-30	MOX Control	0	0	0.5 mL
2	4-6	13-15	22-24	31-33	MenPF-1	25	50	0.5 mL
3	7-9	16-18	25-27	34-36	MenPF-1	50	50	2 x 0.5 mL

9.9.1. Administration of Test and Control Items

The test and control items were administered to the appropriate rabbits by intramuscular injection on Days 1, 22, 43 and 64.

The injection sites (left hind limb – Injection site 1) were clipped free from hair. The aliquots of test and control item were removed from the refrigerator and allowed to warm to room temperature for at least 30 minutes before dosing. To ensure homogeneity, the vials were inverted before dosing and the dose volume required to meet the dosage was administered;

0.5 mL for controls and animals receiving 25 µg/dose or 2 x 0.5 mL µg/dose. The injection sites were delineated.

Animal 27 (Group 3; 50 µg/dose) also received injection in the right hind limb (Injection Site 2) on Day 22. This was due to a small injury caused by clipping of the injection site.

The injection sites were clipped free from hair and delineated before necropsy.

9.9.2. Justification of Route and Dosage Levels

The intramuscular route of administration was selected for this study as this route has been defined by the Sponsor as the route of clinical application/human exposure.

The dose levels were agreed with the Sponsor and took into account the maximum tolerated dose in the test model and other factors such as anticipated therapeutic dose. The test item was produced with similar methodology as for the vaccine product MenBvac (Norwegian Institute of Public Health), based on deoxycholate extracted outer membrane vesicles from *Neisseria meningitidis*. MenBvac is known to be moderately reactogenic but safe in humans (Nøkleby *et al*. Vaccine 2007:25:3080-3084).

Clinical injections are planned every 6 to 8 weeks, with three doses intended. In this study, injections were given to rabbits over a shorter period and one more injection (n + 1) was also given. The intended clinical dose may include a dosage of up to 50 µg/dose. This amount was tested in this preclinical study and based on body weight ratio of rabbit 3 kg: human 60 kg and the administration of an additional injection, this was considered to provide adequate safety data.

9.10. Definition of Day

The first day of treatment (Day 1) ran from midnight before the first administration until 24 h later, subsequent day numbers (Day 2 etc) also followed this pattern. Body weights and food consumption recorded immediately before dosing on the day of treatment started (Day 1 of the study) were classified as Day 0, relating to the number of days of treatment completed. Subsequent recordings also followed this pattern (that is, body weights, food consumption and clinical signs recorded at the end of the 1st day of treatment, Day 2 of the study, were documented as Day 1). Any body weights or food consumption recorded before Day 0 were classified as Day -1, etc. Recording of laboratory investigation bleeds and terminal kills were carried out according to study days, that is, Day 1 being the day treatment started.

9.11. In-life Procedures, Observations, and Measurements

The in-life procedures, observations, and measurements listed below were performed for all animals.

9.11.1. Mortality/Moribundity Checks

Animals were checked early morning and as late as possible each day for viability.

9.11.2. Clinical Observations

9.11.2.1. Detailed Clinical Observations

Once each week, starting during the pretrial period, animals received a detailed clinical examination including appearance, movement and behaviour patterns, skin and hair condition, eyes and mucous membranes, respiration and excreta.

9.11.2.2. Postdose Observations

Animals were examined regularly throughout the day on each dosing day, and once on each non-dosing day. Particular attention was paid to the animals during and for the first hour after dosing. The onset, intensity and duration of any signs were recorded.

9.11.3. Dermal Scoring

Dermal scoring was conducted 0 h (immediately before dosing), 24 h and 48 h after each injection. On 2 separate occasions, scoring was recorded up to 120 h following injections due to erythema observed. Skin was assessed for erythema and eschar formation, oedema formation, skin thickening, desquamation and any other reaction to treatment. The scoring system below was used for assessing erythema, eschar and oedema formation.

Erythema and Eschar Formation	Grade
No erythema	0
Very slight erythema (barely perceptible)	1
Well defined erythema	2
Moderate to severe erythema	3
Severe erythema (beet redness) to slight eschar formation (injuries in depth)	4

Oedema Formation	Grade
No oedema	0
Very slight oedema (barely perceptible)	1
Slight oedema (edges of area well defined by definite raising)	2
Moderate oedema (edges raised approximately 1 mm)	3
Severe oedema (raised by more than 1 mm and extending beyond the area of exposure)	4

9.11.4. Body Weights

Body weights were recorded once during the pretrial period then twice weekly during the dosing and recovery periods.

9.11.5. Food Consumption

The quantity of food consumed by each animal was measured and recorded twice weekly from the beginning of the pretrial period until the end of the study.

9.11.6. Water Consumption

Water consumption was not monitored.

9.11.7. Ophthalmic Examinations

Ophthalmic examinations were carried out on all animals during pretrial and after the completion of dosing. Examinations were conducted by a veterinary surgeon.

The eyes were examined using an indirect ophthalmascope after the application of a mydriatic agent (1% Tropicamide, Mydriacyl®). The anterior, lenticular and fundic areas were examined.

9.11.8. Body Temperature

The body temperature of each animal was measured by digital thermometer inserted into the ear and recorded once during the pretrial period, then 0 h (immediately before dosing), 1 h, 3 h, 24 h and 48 h after each injection.

9.12. Laboratory Evaluations

9.12.1. Clinical Pathology

9.12.1.1. Sample Collection

Blood was collected from an auricular artery. Animals were not fasted prior to sampling. After collection, samples were transferred to the clinical pathology laboratory at Charles River, Edinburgh for processing.

Samples were collected from all animals according to Text Table 3.

Text Table 3
Samples for Clinical Pathology Evaluation

Group Nos.	Time Point	Haematology	Coagulation	Clinical Chemistry
1-3	Pretrial	X	X	X
1-3	Day 66	X	X	X
1-3	Day 92	X	X	X

X = Sample to be collected;

On occasion, repeat samples were collected (where possible) due to clotting of samples. Details are retained in the study records. Values obtained from repeat samples have been reported and used for statistical analysis.

9.12.1.2. Haematology

Blood samples (0.5 mL) were collected into tubes containing EDTA and analysed for the parameters specified in Text Table 4.

Text Table 4
Haematology Parameters

Red blood cell count<br>Haemoglobin<br>Haematocrit<br>Mean cell volume<br>Mean cell haemoglobin concentration<br>Mean cell haemoglobin<br>Reticulocytes (percentage)<br>Reticulocyte count (absolute)<br>Red blood cell distribution width<br>Platelet count<br>Blood Smear	White blood cell count<br>Neutrophils<br>Lymphocytes<br>Monocytes<br>Eosinophils<br>Basophils<br>Large unstained cells<br>Other cells (as appropriate)

A blood smear was prepared from each haematology specimen. Blood smears were labelled, stored and archived. Blood smears were not evaluated as there were no abnormal haematological findings and it was considered that examination would not yield any further information.

9.12.1.3. Coagulation

Blood samples (0.9 mL) were collected into tubes containing 3.8% (w/v) trisodium citrate, processed for plasma, and the plasma analysed for the parameters listed in Text Table 5.

Text Table 5
Coagulation Parameters

Activated partial thromboplastin time<br>Fibrinogen	Prothrombin time

9.12.1.4. Clinical Chemistry

Blood samples (1.5 mL) were collected into tubes containing lithium heparin, processed for serum, and the serum analysed for the parameters specified in Text Table 6.

Text Table 6
Clinical Chemistry Parameters

Urea<br>Glucose<br>Aspartate aminotransferase<br>Alanine aminotransferase<br>Alkaline phosphatase<br>Creatine phosphokinase<br>Lactate dehydrogenase<br>Sodium<br>Potassium<br>Chloride	Total protein<br>Albumin<br>Globulin<br>Albumin/globulin ratio<br>Cholesterol<br>Creatinine<br>Total bilirubin<br>Calcium<br>Inorganic Phosphate

9.12.1.5. Antibody Sample Collection, Processing and Analysis

Blood samples (2 mL) were collected from an auricular artery once during pretrial, and before dosing on Days 22, 64 and 92. Blood samples were allowed to stand at room temperature for a minimum period of 30 min and processed to serum by centrifugation (at least 1500g at 2-8°C for 10 min).

The serum samples were stored in a freezer set to maintain -80°C and then shipped to the Responsible Scientist on dry ice:

Caroline Vipond, Department of Bacteriology, National Institute of Biological Standards and Control (NIBSC), Blance Lane, Potters Bar, South Mimms, Hertfordshire, EN6 3QG, UK.

The samples were to remain frozen throughout transit. Although the temperature was not recorded during transportation, there is evidence that the samples were despatched frozen and were received frozen and in good condition at NIBSC. The immunology laboratory was notified before shipment of the samples, and upon receipt, were stored at ≤-20°C (see protocol deviations in Appendix 1).

The samples were analysed for antibodies against MenPF-1 using a validated ELISA analytical method. No validation was performed for the plate reader software, however, the calibration performed by the service engineer confirmed Operational Qualification (OQ) and standards, and QC samples run with each batch of samples confirmed Performance Qualification (PQ) of the reader.

Any residual anti-therapeutic antibody samples may be retained for research purposes. The results of any subsequent analysis of these samples are not covered in this study.

9.13. Terminal Procedures

Terminal procedures are summarised in Text Table 7.

Text Table 7
Terminal Procedures

Group No.	No. of Animals M	No. of Animals F	Scheduled Euthanasia Day	Necropsy Procedures: Necropsy	Necropsy Procedures: Tissue Collection	Necropsy Procedures: Organ Weights	Histology	Histopathology
1	3	3	66	X	X	X	Full Tissue	Full Tissue[a]
2	3	3					None	None
3	3	3					Full Tissue	Full Tissue[a]
1	3	3	92	X	X	X	Select Tissues	Select Tissues[b]
2	3	3					None	None
3	3	3					Select Tissues	Select Tissues[b]

X = Procedure to be conducted
[a] See Tissue Collection and Preservation table for listing of tissues.
[b] Injection site and lumbar and inguinal lymph node.

9.13.1. Unscheduled Deaths

There were no unscheduled deaths during the study.

9.13.2. Scheduled Euthanasia

Main and recovery study animals were euthanised by an intravenous overdose of a barbiturate, weighed and major blood vessels severed to exsanguinate. The animals were euthanised rotating across dose groups such that similar numbers of animals from each group, including controls were necropsied at similar times throughout the day. Animals were not fasted before their scheduled necropsy.

9.13.3. Necropsy

All main and recovery study animals were subjected to a complete necropsy examination, which included evaluation of the carcass and musculoskeletal system; all external surfaces and orifices; cranial cavity and external surfaces of the brain; and thoracic, abdominal, and pelvic cavities with their associated organs and tissues. Scheduled necropsy examinations were conducted by a trained technician and consisted of an external and internal examination and recording of observations for all animals. A veterinary pathologist was available for consultation during normal working hours.

9.13.4. Organ Weights

The organs identified in Text Table 8 were weighed at necropsy for all animals. Paired organs were weighed and are reported together. Organs were weighed before fixation unless otherwise noted. Terminal body weights were used for organ weight analysis.

Text Table 8
Organs Weighed at Necropsy

Brain	Liver
Epididymis[a]	Lung
Gland, adrenal[a]	Ovary[a]
Gland, pituitary	Spleen
Gland, prostate	Testis[a]
Gland, thyroid[a]	Thymus
Heart	Uterus
Kidney[a]	

[a] Paired organ weight.

9.13.5. Tissue Collection and Preservation

Representative samples of the tissues identified in Text Table 9 were collected from all animals and preserved in 10% neutral buffered formalin, unless otherwise indicated.

Text Table 9
Tissue Collection and Preservation

Administration sites	Liver
Animal identification	Lung
Artery, aorta	Lymph node, mandibular
Bone marrow smear	Lymph node, mesenteric
Bone marrow, femur	Lymph node, lumbar
Bone marrow, sternum	Lymph node, inguinal
Bone, femur	Muscle, skeletal
Bone, sternum	Nerve, optic[a]
Brain	Nerve, sciatic
Cervix	Oesophagus
Epididymis	Ovary
Eye[a]	Oviduct
Gall Bladder	Pancreas
Gland, adrenal	Skin
Gland, lacrimal	Small intestine, duodenum
Gland, mammary	Small intestine, ileum
Gland, parathyroid	Small intestine, jejunum
Gland, pituitary	Spinal cord
Gland, prostate	Spleen
Gland, salivary	Stomach
Gland, seminal vesicle	Testis[b]
Gland, thyroid	Thymus
Gross lesions/masses	Tongue
Gut-associated lymphoid tissue	Trachea
Heart	Ureter
Kidney	Urinary bladder
Large intestine, appendix	Uterus
Large intestine, caecum	Vagina
Large intestine, colon	
Large intestine, rectum	
Large intestine, saccalus rotundus	

[a] Preserved in Davidson’s fixative.
[b] Preserved in Modified Davidson’s fixative.

9.13.6. Histology

Tissues identified in Text Table 9 (except animal identification and bone marrow smears) were embedded in paraffin, sectioned, mounted on glass slides, and stained with haematoxylin and eosin.

Bone marrow smears were collected at necropsy. The smears were retained but not evaluated.

9.13.7. Histopathology

Histopathological evaluation was performed by a veterinary pathologist with training and experience in laboratory animal pathology.

9.13.8. Peer Review

A pathology peer review was conducted by a second pathologist at Charles River Laboratories, Preclinical Services, Tranent, Edinburgh, EH33 2NE, UK as per the appropriate SOP of the Pathology Department.

10. COMPUTERISED SYSTEMS

Critical computerised systems used in the study are listed below. All computerised systems used in the conduct of this study have been validated; when a particular system has not satisfied all requirements, appropriate administrative and procedural controls were implemented to assure the quality and integrity of data. The computer systems used by the Responsible Scientist are detailed in the phase report (Appendix 19).

Text Table 10
Critical Computerized Systems

System Name	Version No.	Description of Data Collected and/or Analysed
Dispense	7.0.3.7	Test item control
Provantis	Release 14	In-life data collection
Nautilus 2003	Release 2	Clinical Pathology Laboratory Information Management System (LIMS)
PLACES 2000	1.1	Histopathology/Organ Weights

11. STATISTICAL ANALYSIS

All statistical tests were two-sided and performed at the 5% significance level using in-house software. Males and females were analysed separately.

Pairwise comparisons were only performed against the control group (Group 1). The following pairwise comparisons were performed:

Control Group *vs* Group 2
Control Group *vs* Group 3

Body weight, food consumption, haematology, coagulation and clinical chemistry were analysed for homogeneity of variance using the 'F-Max' test. If the group variances appeared homogenous, a parametric ANOVA was used and pairwise comparisons were made using Fisher's F protected LSD method *via* Student's t test, i.e. pairwise comparisons were made only if the overall F-test was significant. If the variances were heterogeneous, log or square root transformations were used in an attempt to stabilise the variances. If the variances remained heterogeneous, then a Kruskal-Wallis non-parametric ANOVA was used and pairwise comparisons were made using chi squared protection (*via* z tests, the non-parametric equivalent of Student's t test).

In circumstances where it was not possible to perform the F-Max test due to zero standard deviation in at least one group, the non-parametric ANOVA results were reported.

Organ weights were analysed using ANOVA as above and by analysis of covariance (ANCOVA) using terminal kill body weight as covariate. In addition, organ weights as a percentage of terminal body weight were analysed using ANOVA.

In circumstances where the variances in the ANCOVA remained heterogeneous following log or square root transformations, the data was subjected to rank transformation prior to analysis. Where it was not possible to perform the F-Max test due to the small sample size (less than 3 animals in any group), the untransformed parametric ANCOVA results are reported.

In the ANOVA and ANCOVA summary tables, the results of the analysis are reported indicating the level of statistical significance ($p<0.05$, $p<0.01$ and $p<0.001$) of each pairwise comparison.

Actual p-values are not reported in the summary tables for these analyses.

12. RETENTION OF RECORDS, SAMPLES, AND SPECIMENS

All study-specific raw data, documentation, samples, specimens and final reports from this study are the property of the Sponsor. These materials will be available at the Test Facility during the progress of the study. When the Final Report is issued, all study-specific raw data, documentation, protocol, samples, specimens and final reports will be archived by the Test Facility for a period of 2 years. After this period, the Sponsor will be contacted to determine the disposition of these materials.

Electronic data generated by the Test Facility will be archived and the software and hardware required to produce it in a readable form will be maintained and available.

All records, and reports generated from phases or segments performed by the Test Site will be returned to the Test Facility for archiving. Residue samples, specimens will be retained at the Test Site for research purposes.

13. RESULTS

13.1. Mortality

There were no unscheduled deaths during the observation period.

13.2. Clinical Observations

(Appendices 3 and 4)

There was no signs indicative of systemic toxicity noted during the observation period.

There were local signs recorded at injection sites of 3 animals. Animal 32F (25µg/dose; Group 2) on Days 23-28 had a scab recorded at the injection site and discoloured skin from Days 29-49. Animal 6M (25 µg/dose; Group 2) had a lesion on the injection site on Day 50 and discoloured skin on Day 57 and 64. Animal 27M (50µg/dose; Group 3) had a lesion/scab at the injection site on left hind limb from Days 22-29 and discoloured skin on Day 36.

These local signs were considered minor, transient, had no relationship with dosage and for one animal (Animal 27M) were related to the small injury caused at clipping of injection sites. Overall it was difficult to relate these observations to MenPF-1.

13.3. Dermal Scoring

(Appendix 5)

There was no irritation noted at injection sites that were considered to be related to administration of MenPF-1.

The few instances of very slight erythema that were recorded were sporadic, transient and there was no evidence of a relationship with dosage.

13.4. Body Weight and Body Weight Changes

(Tables 1-2 and Appendices 6-7)

Bodyweight or body weight change was unaffected by treatment with MenPF-1.

13.5. Food Consumption

(Tables 3-4 and Appendices 8-9)

Food consumed was unaffected by treatment with MenPF-1.

There were occasions where a statistically significant difference in food consumption was recorded. These differences were noted in females receiving 25 µg/dose, where food consumed was lower, when compared with controls ($p>0.05$). This lower food consumed was recorded on day 45 of the treatment period and Days 73, 87 and 91 of the recovery period. Inspection of the individual animal data indicated that there was individual variation within the data and that 2 animals (Animals 23F and 24F) consumed less food than others within the group. These differences were isolated, did not result in any difference in body weight and there was no evidence of a relationship with dosage. These differences were considered not to be related to administration with MenPF-1.

13.6. Ophthalmic Examinations

(Appendix 10)

There were no changes in the eye that were related to administration with MenPF-1.

13.7. Body Temperature

(Appendix 11)

Body temperature was unaffected by treatment with MenPF-1.

13.8. Haematology

(Tables 5-7 and Appendices 13-15)

There was an effect on the number of white blood cells in males on Day 66, 48 hours after dosing, that was considered to be related to treatment with MenPF-1.

On Day 66, the number of circulating neutrophils was approximately 2x higher in males receiving 50 µg/dose, when compared with controls ($p<0.01$). The group mean for the controls was 1.29 x 10^9/L with an individual range of 0.88-2.34 x 10^9/L and for the males receiving 50 µg/dose the group mean was 2.43 x 10^9/L with an individual range of 1.58-3.41 x 10^9/L. All of the individual values for the males receiving the vaccine were higher than the group mean of the controls and values were also higher than those recorded pretrial. The number of monocytes was higher in males receiving 25 or 50 µg/dose, when compared with controls ($p<0.01$ or $p<0.05$, respectively).

On Day 66, the mean cell haemoglobin concentration was higher in females receiving 25µg/dose, when compared with controls ($p<0.05$). There was no effect on any other red blood cell index and this minor difference was considered not to be related to treatment with MenPF-1.

At Day 92, haematology was considered to be unaffected by treatment with MenPF-1.

13.9. Coagulation

(Table 5-7 and Appendices 13-15)

There was an effect on fibrinogen in males and females receiving MenPF-1.

On Day 66, fibrinogen was higher in males and females receiving MenPF-1, when compared with controls ($p<0.001$). The group mean values (mg/dL) are summarised.

Males			Females		
Treatment	Pretrial	Day 66	Treatment	Pretrial	Day 66
Control	226	199	Control	168	129
25 µg/dose	225	279	25 µg/dose	171	215
50 µg/dose	230	335	50 µg/dose	179	222

There was also a shorter activated partial thromboplastin time (-8%), which achieved statistical significance, noted in males receiving 50 µg/dose, when compared with controls ($p<0.05$). One of the control values (Animal 21) was longer than the others in the group and this may be in some part due to this value being from a repeat blood collection. If this value is excluded, inspection of the individual data indicated that although there was variation within the data, broadly between the groups the values were similar. Four of the 6 values recorded for males receiving the vaccine are within the control range. There was no evidence of a relationship with dosage with males receiving 25 µg/dose having a longer activated partial thromboplastin time recorded. This small difference was considered not to be related to treatment with MenPF-1. The shorter prothrombin times recorded in males receiving 25µg/dose and females receiving 50 µg/dose, when compared with controls, was considered to be unrelated to treatment with the vaccine given the small magnitude of change and lack of a relationship with dosage.

On Day 92, coagulation was unaffected by treatment with MenPF-1.

13.10. Clinical Chemistry

(Tables 8-10 and Appendices 16-18)

There was an effect on plasma proteins in males and females receiving MenPF-1.

On Day 66, globulin ($p<0.001$) and total protein ($p<0.01$) was higher in males receiving 25 µg/dose and males and females receiving 50 µg/dose, when compared with controls. There was a lower albumin:globulin ratio in these groups. The protein levels were also higher than those recorded pretrial.

There were other statistically significant differences recorded, for example, lower potassium in males receiving 50 µg/dose, when compared with controls, however these differences were considered to be unrelated to treatment with the vaccine given the small magnitude of change and lack of a relationship with dosage.

On Day 92, there were no plasma chemistry differences that were considered to be related to treatment with MenPF-1. There were statistically significant differences recorded, for example, higher aspartate aminotransferase activity in females receiving 25 µg/dose when compared with controls, however these differences were considered to be unrelated to treatment with the vaccine given the small magnitude of change, similar values recorded pretrial and lack of a relationship with dosage.

13.11. Antibody Analysis

(Appendix 19)

The data provided by the Sponsor-designated Responsible Scientist indicated the presence of specific IgG to dosages of 25 or 50 µg MenPF-1/dose. An increase in titre was generally observed with increasing dose and time in both males and females. Although the group mean for each of the groups receiving MenPF-1 was lower on Day 92, inspection of the individual data indicated that 7/12 animals had a similar or higher titre then those recorded on Day 64.

13.12. Gross Pathology

(Tables 11-12; Appendices 20-21 and 26)

13.12.1. Scheduled Euthanasia (Day 66)

There were enlarged lumbar lymph nodes (left) recorded in 2/3 males and 1/3 females that received 50 µg/dose.

Other gross findings observed were considered incidental, of the nature commonly observed in this strain and age of rabbit, and/or were of similar incidence in control and treated animals and, therefore, were considered unrelated to administration of MenPF-1.

13.12.2. Scheduled Euthanasia (Day 92)

Test article-related gross findings noted at the terminal euthanasia were not observed at the end of the recovery period. Other gross findings observed were considered incidental, of the nature commonly observed in this strain and age of rabbit, and/or were of similar incidence in control and treated animals and, therefore, were considered unrelated to administration of MenPF-1.

13.13. Organ Weights

(Tables 13-18 and Appendices 22-25)

Organ weights were considered to be unaffected by treatment with MenPF-1.

On Day 66, absolute adrenal gland weights were statistically higher in females that received 25 or 50 µg/dose, when compared with controls ($p<0.05$). No dose-response was evident. There was no difference noted after adjustment for terminal body weight and as analysis as a percentage of terminal body weight (relative). This difference was considered not to be related to treatment with MenPF-1.

On Day 92, there were statistically significant differences in males and females that received 25 or 50 µg/dose, when compared with controls; lower absolute and relative liver weight in females (25 µg/dose), lower covariant thymus weight in females (50 µg/dose) and a higher liver weight in females (50 µg/dose). There was non histological correlate and no relationship with dosage, consequently these differences were considered not to be related to treatment with MenPF-1.

13.14. Histopathology

(Tables 19-20; Appendices 20-21 and 26)

13.14.1. Scheduled Euthanasia (Day 66)

There was accumulation of macrophages, observed both in the injection sites and lumbar lymph nodes, which was characterised by aggregates of macrophages containing an abundant, pale basophilic, amorphous cytoplasmic material considered to be aluminium hydroxide. These macrophages were admixed with variable numbers of multinucleated giant cells.

Lymphoid hyperplasia was also recorded which correlated with the enlarged lumbar lymph nodes observed at necropsy.

Other microscopic findings at this dose level observed were considered incidental, of the nature commonly observed in this strain and age of rabbit, and/or were of similar incidence and severity in control and treated animals and, therefore, were considered unrelated to administration of MenPF-1.

A number of changes were observed in the clinical chemistry, haematology and coagulation group mean values, when compared to their respective controls: there were increased total proteins and globulins, and decreased albumin/globulin ratio in all treated males and in females given 50 µg/dose; increased neutrophil counts in males given 50 µg/dose; increased monocyte counts in all treated male groups; and increased fibrinogen in treated groups from both sexes. These differences correlated with the inflammatory reaction observed in the injection sites.

13.14.2. Scheduled Euthanasia (Day 92)

Some of the microscopic findings noted at the terminal euthanasia (Day 66) were observed at the end of the period off dose (Day 92), however were of a lesser severity and frequency. No treatment related findings were noted in Injection Site 2 (Animal 27).

Other microscopic findings observed were considered incidental, of the nature commonly observed in this strain and age of rabbit, and/or were of similar incidence and severity in control and treated animals and, therefore, were considered unrelated to administration of MenPF-1.

14. DISCUSSION

Intramuscular administration of up to 50 µg/dose of MenPF-1 was associated with an expected, minor physiological response and findings of macrophages, giant cells and polymorphonuclear inflammation at the injection sites and lymph node enlargement at the draining lymph node. These findings were noted 4 weeks after the last injection, however, they were of a lesser severity indicating recovery.

The presence of antibodies to MenPF-1 indicated immunogenicity in rabbits, and confirmed that this species was a suitable selection for this study.

The local inflammatory response noted histologically with the findings of myofibre necrosis and/or regeneration, interstitial fibrosis and/or mineralisation at the injection sites and lymphoid hyperplasia at the injection site draining lymph node correlated systemically with higher fibrinogen and higher levels of the acute phase protein globulin noted after four injections. These minor differences in protein levels are considered to be a physiological response and of little toxicological significance.

The accumulation of the macrophages noted at the injection sites and lymph nodes was considered to be related to the aluminium hydroxide. This response is not unusual where an aluminium based adjuvant has been administered.

15. CONCLUSION

In conclusion, administration of the vaccine, MenPF-1, when given by intramuscular injection for 4 occasions over a 9 week period, was well tolerated in rabbits up to 50 µg/dose. There was only an expected, minor inflammatory response which was associated with vaccine administration, characterised by macrophages, giant cells and polymorphonuclear and mononuclear inflammation at the injection sites with on-going recovery noted. There was no evidence of systemic toxicity.

16. REFERENCES

Nøkleby *et al* (2007). Safety review: Two outer membrane vesicle (OMV) vaccines against systemic *Neisseria meningitidis* serogroup B disease. Vaccine 25 (2007) 3080-3084

Figures

Figure 1 Cage Plan

Treatment Period

Group No.
Animal No.
Cage No.

Animal Rack 1

1<br>1<br>1	1<br>2<br>2
1<br>3<br>3	1<br>19<br>19
1<br>20<br>20	1<br>21<br>21

Animal Rack 2

1<br>10<br>10	1<br>11<br>11
1<br>12<br>12	1<br>28<br>28
1<br>29<br>29	1<br>30<br>30

Group No.
Animal No.
Cage No.

Animal Rack 3

2<br>4<br>4	2<br>5<br>5
2<br>6<br>6	2<br>22<br>22
2<br>23<br>23	2<br>24<br>24

Animal Rack 4

2<br>13<br>13	2<br>14<br>14
2<br>15<br>15	2<br>31<br>31
2<br>32<br>32	2<br>33<br>33

Group No.
Animal No.
Cage No.

Animal Rack 5

3<br>7<br>7	3<br>8<br>8
3<br>9<br>9	3<br>25<br>25
3<br>26<br>26	3<br>27<br>27

Group No.
Animal No.
Cage No.

Animal Rack 6

3<br>16<br>16	3<br>17<br>17
3<br>18<br>18	3<br>34<br>34
3<br>35<br>35	3<br>36<br>36

Figure 1 (continued) **Cage Plan**

Recovery Period

Legend for each cage: Group No. / Animal No. / Cage No.

Animal Rack 1

1<br>19<br>19	1<br>20<br>20
1<br>21<br>21	

Animal Rack 2

2<br>22<br>22	2<br>23<br>23
2<br>24<br>24	3<br>25<br>25
3<br>26<br>26	3<br>27<br>27

Animal Rack 3

1<br>28<br>28	1<br>29<br>29
1<br>30<br>30	

Animal Rack 4

2<br>31<br>31	2<br>32<br>32
2<br>33<br>33	3<br>34<br>34
3<br>35<br>35	3<br>36<br>36

Animal Rack 5

Animal Rack 6

Figure 2
Body Weights (kg): Group Mean Values: Males

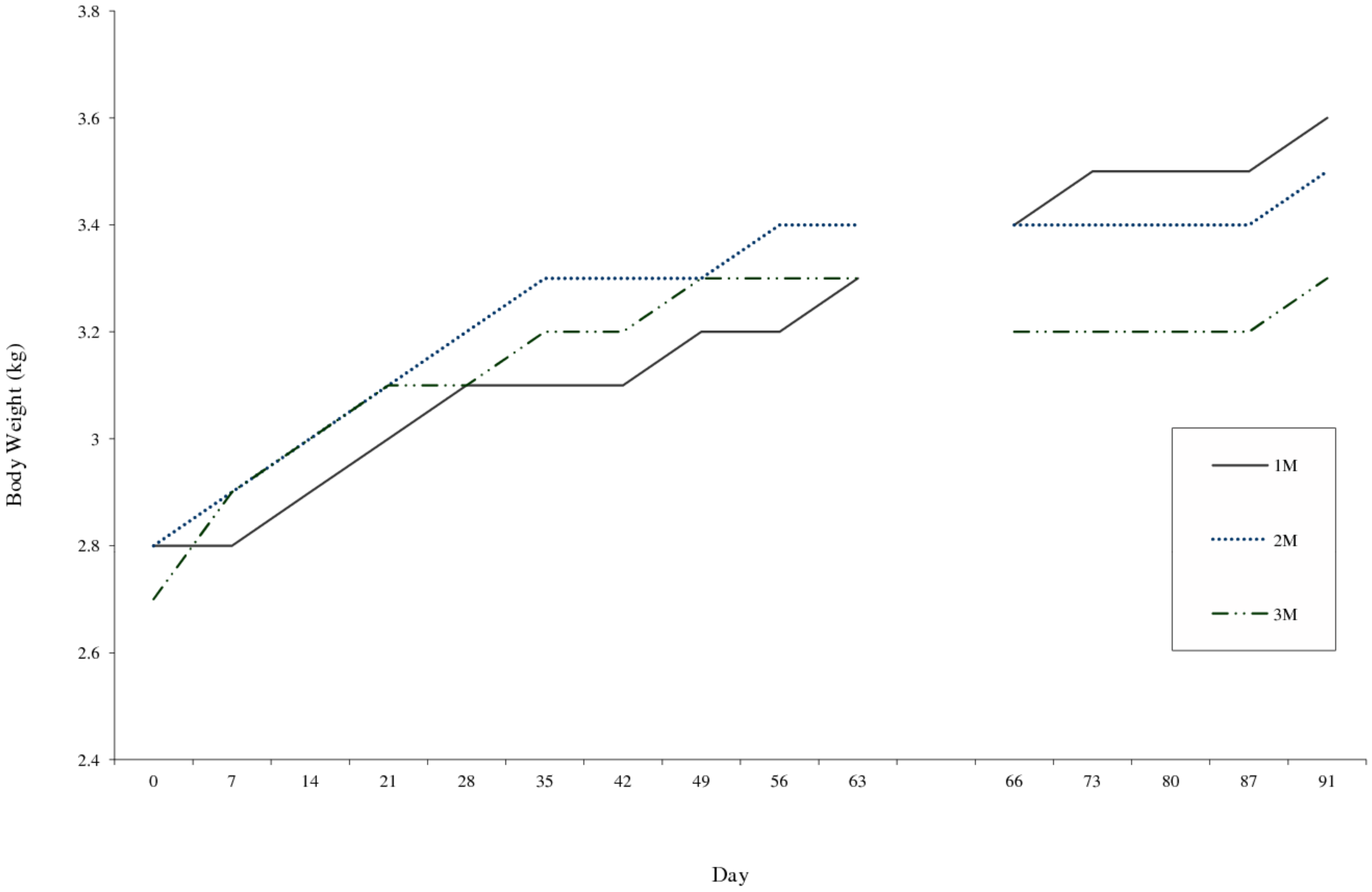

Figure 2 (continued)
Body Weights (kg): Group Mean Values: Females

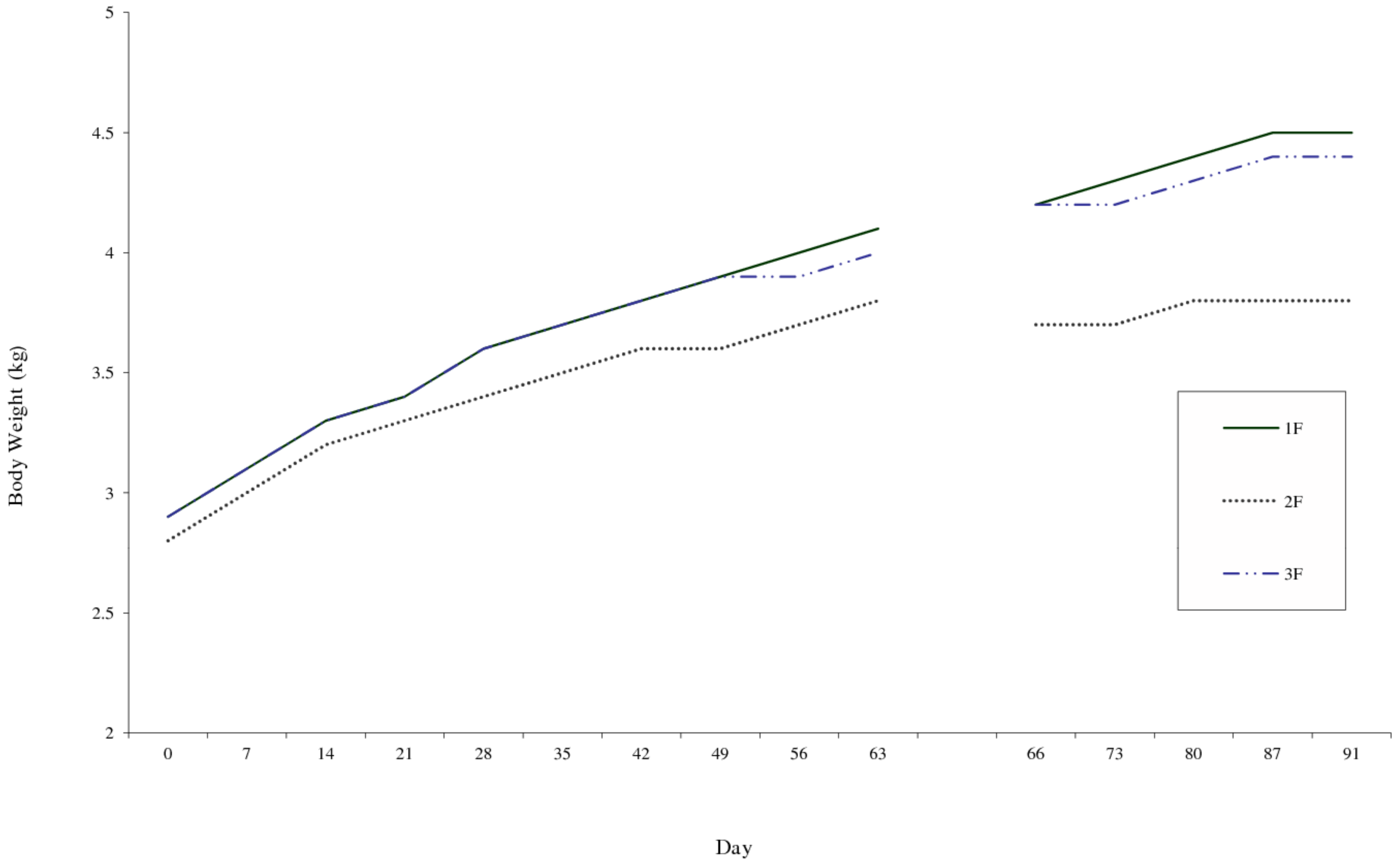

Tables

Table 1
Body Weights with Change (kg): Group Mean Values: Treatment Period

Group	:	1	2	3
Test Item	:	Control	MenPF-1	MenPF-1
Dosage (µg/dose)	:	0	25	50

Group / sex		Day -7	0	3	7	10	14	17	21	24	28	31	35	38
1M	Mean	2.6	2.8	2.8	2.8	2.9	2.9	3.0	3.0	3.0	3.1	3.1	3.1	3.1
	SD	0.1	0.1	0.1	0.0	0.1	0.1	0.1	0.1	0.1	0.1	0.1	0.1	0.1
	n	6	6	6	6	6	6	6	6	6	6	6	6	6
2M	Mean	2.6	2.8	2.8	2.9	3.0	3.0	3.0	3.1	3.1	3.2	3.2	3.3	3.3
	SD	0.1	0.1	0.0	0.1	0.1	0.1	0.1	0.1	0.1	0.1	0.1	0.1	0.1
	n	6	6	6	6	6	6	6	6	6	6	6	6	6
3M	Mean	2.6	2.7	2.8	2.9	2.9	3.0	3.0	3.1	3.1	3.1	3.1	3.2	3.2
	SD	0.1	0.1	0.1	0.1	0.1	0.1	0.1	0.1	0.1	0.1	0.1	0.1	0.2
	n	6	6	6	6	6	6	6	6	6	6	6	6	6

Significantly different from Group 1:a=p<0.05,b=p<0.01,c=p<0.001

Table 1 (continued)
Body Weights with Change (kg): Group Mean Values: Treatment Period

Group	:	1	2	3
Test Item	:	Control	MenPF-1	MenPF-1
Dosage (µg/dose)	:	0	25	50

Group / sex		Day 42	45	49	52	56	59	63	Change 0 - 63
1M	Mean	3.1	3.1	3.2	3.3	3.2	3.3	3.3	0.5
	SD	0.1	0.1	0.1	0.1	0.1	0.2	0.1	0.1
	n	6	6	6	6	6	6	6	6
2M	Mean	3.3	3.3	3.3	3.3	3.4	3.4	3.4	0.6
	SD	0.1	0.1	0.2	0.2	0.1	0.1	0.1	0.1
	n	6	6	6	6	6	6	6	6
3M	Mean	3.2	3.3	3.3	3.3	3.3	3.3	3.3	0.6
	SD	0.2	0.2	0.2	0.2	0.2	0.2	0.2	0.2
	n	6	6	6	6	6	6	6	6

Significantly different from Group 1:a=p<0.05,b=p<0.01,c=p<0.001

Table 1 (continued)
Body Weights with Change (kg): Group Mean Values: Treatment Period

Group	:	1	2	3
Test Item	:	Control	MenPF-1	MenPF-1
Dosage (µg/dose)	:	0	25	50

Group / sex		Day -7	0	3	7	10	14	17	21	24	28	31	35	38
1F	Mean	2.7	2.9	3.0	3.1	3.2	3.3	3.4	3.4	3.5	3.6	3.6	3.7	3.7
	SD	0.1	0.1	0.2	0.2	0.2	0.2	0.2	0.3	0.3	0.2	0.3	0.2	0.2
	n	6	6	6	6	6	6	6	6	6	6	6	6	6
2F	Mean	2.6	2.8	2.9	3.0	3.1	3.2	3.2	3.3	3.3	3.4	3.5	3.5	3.5
	SD	0.1	0.1	0.2	0.1	0.2	0.2	0.2	0.2	0.2	0.2	0.2	0.2	0.2
	n	6	6	6	6	6	6	6	6	6	6	6	6	6
3F	Mean	2.7	2.9	3.0	3.1	3.2	3.3	3.4	3.4	3.5	3.6	3.6	3.7	3.7
	SD	0.2	0.2	0.2	0.2	0.2	0.2	0.2	0.2	0.3	0.3	0.3	0.3	0.3
	n	6	6	6	6	6	6	6	6	6	6	6	6	6

Significantly different from Group 1:a=p<0.05,b=p<0.01,c=p<0.001

Table 1 (continued)
Body Weights with Change (kg): Group Mean Values: Treatment Period

Group	:	1	2	3
Test Item	:	Control	MenPF-1	MenPF-1
Dosage (µg/dose)	:	0	25	50

Group / sex		Day 42	45	49	52	56	59	63	Change 0 - 63
1F	Mean	3.8	3.8	3.9	3.9	4.0	4.1	4.1	1.2
	SD	0.3	0.3	0.3	0.3	0.3	0.3	0.3	0.2
	n	6	6	6	6	6	6	6	6
2F	Mean	3.6	3.6	3.6	3.6	3.7	3.8	3.8	1.0
	SD	0.3	0.2	0.3	0.3	0.3	0.3	0.3	0.3
	n	6	6	6	6	6	6	6	6
3F	Mean	3.8	3.8	3.9	3.9	3.9	4.0	4.0	1.1
	SD	0.3	0.3	0.4	0.4	0.4	0.4	0.4	0.3
	n	6	6	6	6	6	6	6	6

Significantly different from Group 1:a=p<0.05,b=p<0.01,c=p<0.001

Table 2
Body Weights with Change (kg): Group Mean Values: Recovery Period

Group	:	1	2	3
Test Item	:	Control	MenPF-1	MenPF-1
Dosage (µg/dose)	:	0	25	50

Group / sex		Day 66	70	73	77	80	84	87	91	Change 66 - 91
1M	Mean	3.4	3.4	3.5	3.5	3.5	3.5	3.5	3.6	0.2
	SD	0.2	0.2	0.2	0.2	0.2	0.2	0.2	0.2	0.1
	n	3	3	3	3	3	3	3	3	3
2M	Mean	3.4	3.4	3.4	3.5	3.4	3.5	3.4	3.5	0.1
	SD	0.2	0.2	0.2	0.2	0.2	0.2	0.3	0.2	0.1
	n	3	3	3	3	3	3	3	3	3
3M	Mean	3.2	3.2	3.2	3.2	3.2	3.3	3.2	3.3	0.1
	SD	0.2	0.3	0.2	0.2	0.2	0.3	0.2	0.3	0.1
	n	3	3	3	3	3	3	3	3	3

Significantly different from Group 1:a=p<0.05,b=p<0.01,c=p<0.001

Table 2 (continued)
Body Weights with Change (kg): Group Mean Values: Recovery Period

Group	:	1	2	3
Test Item	:	Control	MenPF-1	MenPF-1
Dosage (µg/dose)	:	0	25	50

Group / sex		Day 66	70	73	77	80	84	87	91	Change 66 - 91
1F	Mean	4.2	4.3	4.3	4.4	4.4	4.5	4.5	4.5	0.3
	SD	0.4	0.4	0.4	0.4	0.4	0.4	0.5	0.4	0.1
	n	3	3	3	3	3	3	3	3	3
2F	Mean	3.7	3.7	3.7	3.8	3.8	3.8	3.8	3.8	0.1
	SD	0.2	0.3	0.3	0.3	0.3	0.3	0.3	0.3	0.1
	n	3	3	3	3	3	3	3	3	3
3F	Mean	4.2	4.1	4.2	4.3	4.3	4.3	4.4	4.4	0.3
	SD	0.2	0.2	0.2	0.2	0.2	0.2	0.2	0.2	0.1
	n	3	3	3	3	3	3	3	3	3

Significantly different from Group 1:a=p<0.05,b=p<0.01,c=p<0.001

Table 3
Food Consumption (g/animal/day): Group Mean Values: Treatment Period

Group	:	1	2	3
Test Item	:	Control	MenPF-1	MenPF-1
Dosage (µg/dose)	:	0	25	50

Group / sex		Day 0	3	7	10	14	17	21	24	28	31	35	38	42
1M	Mean	128.9	123.4	124.8	119.7	138.5	127.2	139.8	124.5	132.1	124.9	133.3	127.9	131.8
	SD	16.9	14.1	10.6	17.6	14.4	16.1	13.2	18.3	17.1	20.2	14.9	21.2	17.1
	n	6	6	6	6	6	6	6	6	6	6	6	6	6
2M	Mean	140.8	132.5	140.1	134.4	149.1	131.6	145.6	130.0	135.1	134.4	135.1	130.6	137.7
	SD	13.9	22.1	17.7	25.8	24.9	18.3	22.8	19.4	25.1	21.5	20.0	15.2	20.8
	n	6	6	6	6	6	6	6	6	6	6	6	6	6
3M	Mean	129.1	122.6	132.8	128.1	137.8	131.2	137.5	122.1	128.7	126.4	126.8	126.1	131.1
	SD	11.2	5.8	11.8	11.4	14.8	16.1	13.9	16.2	9.0	13.6	10.0	16.4	20.0
	n	6	6	6	6	6	6	6	6	6	6	6	6	6

Significantly different from Group 1:a=p<0.05,b=p<0.01,c=p<0.001

Table 3 (continued)
Food Consumption (g/animal/day): Group Mean Values: Treatment Period

Group	:	1	2	3
Test Item	:	Control	MenPF-1	MenPF-1
Dosage (µg/dose)	:	0	25	50

Group / sex		Day 45	49	52	56	59	63
1M	Mean	128.7	135.8	141.7	135.0	131.6	140.9
	SD	20.1	21.3	19.7	17.6	15.9	17.1
	n	6	6	6	6	6	6
2M	Mean	125.2	131.3	137.5	136.3	145.8	139.1
	SD	26.8	18.9	28.3	18.4	18.0	17.7
	n	6	6	6	6	6	6
3M	Mean	123.2	129.0	133.2	130.0	130.2	122.0
	SD	13.9	12.6	12.7	18.8	17.2	16.6
	n	6	6	6	6	6	6

Significantly different from Group 1:a=p<0.05,b=p<0.01,c=p<0.001

Table 3 (continued)
Food Consumption (g/animal/day): Group Mean Values: Treatment Period

Group	:	1	2	3
Test Item	:	Control	MenPF-1	MenPF-1
Dosage (µg/dose)	:	0	25	50

Group / sex		Day 0	3	7	10	14	17	21	24	28	31	35	38	42
1F	Mean	154.5	139.0	160.0	144.1	166.2	153.7	168.3	152.6	161.2	155.7	161.3	159.5	167.7
	SD	17.3	21.4	28.9	27.6	25.0	28.4	27.4	25.5	29.1	22.2	24.8	27.3	29.1
	n	6	6	6	6	6	6	6	6	6	6	6	6	6
2F	Mean	149.1	136.1	151.7	144.7	160.9	144.1	162.9	140.4	147.9	147.2	163.4	148.5	150.7
	SD	10.0	12.4	11.3	15.8	15.4	15.6	12.8	17.2	15.0	25.3	14.1	21.9	25.3
	n	6	6	6	6	6	6	6	6	6	6	6	6	6
3F	Mean	164.7	157.0	176.4	162.3	184.7	168.3	180.8	163.7	175.3	172.8	180.5	174.6	176.0
	SD	25.9	25.4	34.1	25.6	45.0	27.5	37.2	32.7	36.0	38.5	38.1	35.5	40.2
	n	6	6	6	6	6	6	6	6	6	6	6	6	6

Significantly different from Group 1:a=$p<0.05$,b=$p<0.01$,c=$p<0.001$

Table 3 (continued)
Food Consumption (g/animal/day): Group Mean Values: Treatment Period

Group	:	1	2	3
Test Item	:	Control	MenPF-1	MenPF-1
Dosage (µg/dose)	:	0	25	50

Group / sex		Day 45	49	52	56	59	63
1F	Mean	166.1	170.3	167.9	180.1	176.0	169.4
	SD	20.5	27.8	26.7	28.7	23.7	25.6
	n	6	6	6	6	6	6
2F	Mean	136.9[a]	134.2	132.5	150.5	153.5	152.1
	SD	22.5	39.4	47.8	37.7	29.6	28.0
	n	6	6	6	6	6	6
3F	Mean	167.6	171.5	169.2	170.7	164.3	153.3
	SD	15.4	25.7	39.3	51.8	23.9	36.4
	n	6	6	6	6	6	6

Significantly different from Group 1:a=p<0.05,b=p<0.01,c=p<0.001

Table 4
Food Consumption (g/animal/day): Group Mean Values: Recovery Period

Group	:	1	2	3
Test Item	:	Control	MenPF-1	MenPF-1
Dosage (µg/dose)	:	0	25	50

Group / sex		Day 66	70	73	77	80	84	87	91
1M	Mean	148.6	133.4	121.8	131.4	114.2	139.8	129.7	143.8
	SD	28.8	20.0	29.2	28.7	25.8	11.1	14.4	4.6
	n	3	3	3	3	3	3	3	3
2M	Mean	126.9	112.1	122.0	130.1	111.3	120.9	111.7	126.8
	SD	6.6	21.3	24.4	22.1	20.6	17.6	29.0	28.9
	n	3	3	3	3	3	3	3	3
3M	Mean	108.3	108.5	105.7	114.9	89.5	117.5	112.1	119.4
	SD	8.7	4.9	7.2	16.5	11.9	13.2	17.4	15.7
	n	3	3	3	3	3	3	3	3

Significantly different from Group 1:a=p<0.05,b=p<0.01,c=p<0.001

Table 4 (continued)
Food Consumption (g/animal/day): Group Mean Values: Recovery Period

Group	:	1	2	3
Test Item	:	Control	MenPF-1	MenPF-1
Dosage (µg/dose)	:	0	25	50

Group / sex		Day							
		66	70	73	77	80	84	87	91
1F	Mean	164.0	172.3	172.0	177.7	161.3	172.9	165.8	171.0
	SD	29.4	34.2	26.3	25.8	33.3	24.5	28.0	20.2
	n	3	3	3	3	3	3	3	3
2F	Mean	117.8	131.0	111.9[a]	145.4	112.1	123.4	110.8[a]	126.4[a]
	SD	25.7	21.4	22.4	28.9	21.1	34.2	20.4	17.3
	n	3	3	3	3	3	3	3	3
3F	Mean	146.3	154.2	166.0	183.8	152.0	172.4	160.1	167.8
	SD	37.0	15.9	8.6	19.1	8.4	14.6	16.8	16.9
	n	3	3	3	3	3	3	3	3

Significantly different from Group 1:a=p<0.05,b=p<0.01,c=p<0.001

Table 5
Haematology and Coagulation : Group Mean Values: Pretrial

Group	:	1	2	3
Test Item	:	Control	MenPF-1	MenPF-1
Dosage (µg/dose)	:	0	25	50

Group / sex		Hb g/dL	RBC x10¹²/L	Hct L/L	MCH pg	MCV fL	MCHC g/dL	RDW %	Reti %	Ret x10⁹/L	WBC -----------	Neut -----------	Lymph -----------	Mono x10⁹/L	Eos -----------	Baso -----------
1M	Mean	13.0	6.18	0.377	21.1	61.1	34.5	12.7	2.5	150	6.50	1.08	4.67	0.12	0.17	0.46
	SD	0.4	0.33	0.014	1.0	2.1	0.7	0.9	1.4	75	1.09	0.28	1.18	0.07	0.05	0.06
	n	6	6	6	6	6	6	6	6	6	6	6	6	6	6	6
2M	Mean	12.8	6.15	0.369	20.8	60.2	34.5	12.5	1.9	113	6.35	1.67	3.92	0.09	0.15	0.51
	SD	0.4	0.35	0.009	1.0	2.2	0.7	0.2	0.3	14	0.97	1.06	0.44	0.05	0.05	0.08
	n	6	6	6	6	6	6	6	6	6	6	6	6	6	6	6
3M	Mean	12.8	6.14	0.371	20.8	60.5	34.4	12.6	2.1	127	6.63	1.63	4.17	0.10	0.15	0.57
	SD	0.6	0.16	0.010	0.8	1.2	0.7	0.9	0.5	29	1.21	0.55	0.50	0.03	0.08	0.19
	n	6	6	6	6	6	6	6	6	6	6	6	6	6	6	6

Significantly different from Group 1:a=p<0.05,b=p<0.01,c=p<0.001

Table 5 (continued)
Haematology and Coagulation : Group Mean Values: Pretrial

Group	:	1	2	3
Test Item	:	Control	MenPF-1	MenPF-1
Dosage (µg/dose)	:	0	25	50

Group / sex		LUC -----------	Plat x10⁹/L	PT s	APTT s	Fib mg/dL
1M	Mean	0.01	409	6.3	60.7	226
	SD	0.00	68	0.2	3.1	15
	n	6	6	6	6	6
2M	Mean	0.01	402	6.4	62.5	225
	SD	0.00	98	0.3	4.5	19
	n	5	6	6	6	6
3M	Mean	0.02	444	6.2	58.9	230
	SD	0.01	38	0.2	6.0	20
	n	6	6	6	6	6

Significantly different from Group 1:a=p<0.05,b=p<0.01,c=p<0.001

Table 5 (continued)
Haematology and Coagulation : Group Mean Values: Pretrial

Group	:	1	2	3
Test Item	:	Control	MenPF-1	MenPF-1
Dosage (µg/dose)	:	0	25	50

Group / sex		Hb g/dL	RBC x10¹²/L	Hct L/L	MCH pg	MCV fL	MCHC g/dL	RDW %	Reti %	Ret x10⁹/L	WBC -----------	Neut -----------	Lymph -----------	Mono x10⁹/L	Eos -----------	Baso -----------
1F	Mean	11.9	5.58	0.349	21.3	62.6	34.0	12.4	2.6	145	6.51	2.03	3.73	0.07	0.14	0.54
	SD	0.6	0.40	0.017	0.6	1.6	0.5	0.5	0.5	22	1.12	0.90	0.80	0.04	0.08	0.22
	n	6	6	6	6	6	6	6	6	6	6	6	6	6	6	6
2F	Mean	12.4	5.82	0.361	21.4	62.0	34.4	12.7	3.0	173	6.87	1.82	4.20	0.08	0.15	0.60
	SD	0.2	0.19	0.007	0.8	2.6	0.3	0.8	0.5	31	1.19	0.66	0.49	0.02	0.04	0.12
	n	6	6	6	6	6	6	6	6	6	6	6	6	6	6	6
3F	Mean	12.2	5.67	0.354	21.6	62.6	34.5	12.5	2.5	141	6.74	1.87	4.03	0.12	0.17	0.55
	SD	0.8	0.38	0.021	0.8	2.2	0.8	0.5	0.4	21	0.82	0.54	0.95	0.08	0.04	0.13
	n	6	6	6	6	6	6	6	6	6	6	6	6	6	6	6

Significantly different from Group 1:a=p<0.05,b=p<0.01,c=p<0.001

Table 5 (continued)
Haematology and Coagulation : Group Mean Values: Pretrial

Group	:	1	2	3
Test Item	:	Control	MenPF-1	MenPF-1
Dosage (µg/dose)	:	0	25	50

Group / sex		LUC -----------	Plat x10⁹/L	PT s	APTT s	Fib mg/dL
1F	Mean	0.01	431	6.2	55.5	168
	SD	0.00	73	0.3	2.3	16
	n	4	6	6	6	6
2F	Mean	0.02	463	6.4	55.5	171
	SD	0.01	73	0.1	3.6	18
	n	6	6	5	5	5
3F	Mean	0.02	454	6.2	51.4	179
	SD	0.01	69	0.2	7.2	15
	n	6	6	6	6	6

Significantly different from Group 1:a=p<0.05,b=p<0.01,c=p<0.001

Table 6
Haematology and Coagulation : Group Mean Values: Day 66

Group	:	1	2	3
Test Item	:	Control	MenPF-1	MenPF-1
Dosage (µg/dose)	:	0	25	50

Group / sex		Hb g/dL	RBC $x10^{12}/L$	Hct L/L	MCH pg	MCV fL	MCHC g/dL	RDW %	Reti %	Ret $x10^9/L$	WBC -----------	Neut -----------	Lymph -----------	Mono $x10^9/L$	Eos -----------	Baso -----------
1M	Mean	13.1	6.42	0.397	20.4	61.9	33.0	12.0	2.5	162	7.39	1.29	5.35	0.05	0.18	0.52
	SD	0.4	0.10	0.007	0.7	1.4	0.5	0.6	0.4	26	1.62	0.53	1.43	0.02	0.05	0.11
	n	6	6	6	6	6	6	6	6	6	6	6	6	6	6	6
2M	Mean	13.3	6.42	0.398	20.7	62.2	33.3	12.2	2.7	174	6.64	1.74	4.18	0.13[b]	0.15	0.44
	SD	0.6	0.40	0.015	0.8	2.1	0.5	0.5	0.6	36	1.50	0.44	1.13	0.10	0.03	0.14
	n	5	5	5	5	5	5	5	5	5	5	5	5	5	5	5
3M	Mean	13.1	6.44	0.399	20.3	62.1	32.8	12.1	2.5	162	8.31	2.43[b]	5.03	0.09[a]	0.19	0.56
	SD	0.6	0.26	0.015	0.7	1.3	0.7	0.4	0.6	42	1.53	0.73	0.84	0.02	0.08	0.18
	n	6	6	6	6	6	6	6	6	6	6	6	6	6	6	6

Significantly different from Group 1:a=p<0.05,b=p<0.01,c=p<0.001

Table 6 (continued)
Haematology and Coagulation : Group Mean Values: Day 66

Group	:	1	2	3
Test Item	:	Control	MenPF-1	MenPF-1
Dosage (µg/dose)	:	0	25	50

Group / sex		LUC -----------	Plat x10⁹/L	PT s	APTT s	Fib mg/dL
1M	Mean	0.01	364	6.1	56.2	199
	SD	0.00	37	0.3	4.5	35
	n	6	6	6	6	6
2M	Mean	0.01	414	5.8[a]	58.8	279[c]
	SD	0.01	102	0.0	2.5	29
	n	4	5	5	5	5
3M	Mean	0.01	382	5.9	51.8[a]	335[c]
	SD	0.01	49	0.1	2.4	30
	n	6	6	6	6	6

Significantly different from Group 1:a=p<0.05,b=p<0.01,c=p<0.001

Table 6 (continued)
Haematology and Coagulation : Group Mean Values: Day 66

Group	:	1	2	3
Test Item	:	Control	MenPF-1	MenPF-1
Dosage (µg/dose)	:	0	25	50

Group / sex		Hb g/dL	RBC x10¹²/L	Hct L/L	MCH pg	MCV fL	MCHC g/dL	RDW %	Reti %	Ret x10⁹/L	WBC -----------	Neut -----------	Lymph -----------	Mono x10⁹/L	Eos -----------	Baso -----------
1F	Mean	12.2	5.89	0.374	20.7	63.7	32.5	12.7	3.1	179	7.91	1.51	5.58	0.06	0.18	0.56
	SD	0.8	0.53	0.025	0.6	1.9	0.3	0.4	0.5	18	4.34	0.55	4.06	0.05	0.06	0.19
	n	6	6	6	6	6	6	6	6	6	6	6	6	6	6	6
2F	Mean	12.8	6.20	0.386	20.7	62.3	33.2[a]	12.4	2.9	178	6.89	1.76	4.34	0.08	0.17	0.53
	SD	0.4	0.30	0.013	0.9	2.3	0.4	0.6	0.4	28	0.70	0.57	0.70	0.03	0.05	0.12
	n	6	6	6	6	6	6	6	6	6	6	6	6	6	6	6
3F	Mean	12.2	5.90	0.370	20.7	62.7	32.9	12.3	3.1	184	6.61	1.62	4.18	0.13	0.15	0.51
	SD	0.3	0.33	0.014	0.8	1.8	0.6	0.9	1.1	72	1.38	0.47	1.04	0.09	0.06	0.08
	n	6	6	6	6	6	6	6	6	6	6	6	6	6	6	6

Significantly different from Group 1:a=p<0.05,b=p<0.01,c=p<0.001

Table 6 (continued)
Haematology and Coagulation : Group Mean Values: Day 66

Group	:	1	2	3
Test Item	:	Control	MenPF-1	MenPF-1
Dosage (µg/dose)	:	0	25	50

Group / sex		LUC<br>-----------	Plat<br>$x10^9/L$	PT<br>s	APTT<br>s	Fib<br>mg/dL
1F	Mean	0.02	423	6.1	55.6	129
	SD	0.01	91	0.2	4.2	16
	n	5	6	6	6	6
2F	Mean	0.01	402	5.9	56.4	215[c]
	SD	0.01	89	0.2	5.2	35
	n	5	6	6	6	6
3F	Mean	0.01	481	5.8[b]	58.6	222[c]
	SD	0.01	104	0.1	8.8	49
	n	6	6	6	6	6

Significantly different from Group 1:a=p<0.05,b=p<0.01,c=p<0.001

Table 7
Haematology and Coagulation: Group Mean Values: Day 92

Group	:	1	2	3
Test Item	:	Control	MenPF-1	MenPF-1
Dosage (µg/dose)	:	0	25	50

Group / sex		Hb g/dL	RBC $x10^{12}/L$	Hct L/L	MCH pg	MCV fL	MCHC g/dL	RDW %	Reti %	Ret $x10^9/L$	WBC -----------	Neut -----------	Lymph -----------	Mono $x10^9/L$	Eos -----------	Baso -----------
1M	Mean	13.6	6.40	0.408	21.2	63.7	33.2	11.9	2.7	170	6.64	1.15	4.73	0.03	0.21	0.51
	SD	0.7	0.25	0.018	0.6	0.6	0.6	0.8	0.4	22	1.06	0.38	0.64	0.02	0.02	0.05
	n	3	3	3	3	3	3	3	3	3	3	3	3	3	3	3
2M	Mean	13.5	6.44	0.410	21.0	63.7	33.0	11.9	2.1	131	6.03	1.26	4.10	0.05	0.15	0.48
	SD	1.1	0.72	0.030	0.6	2.5	0.4	0.1	0.4	37	1.46	0.70	0.49	0.01	0.06	0.21
	n	2	2	2	2	2	2	2	2	2	2	2	2	2	2	2
3M	Mean	13.4	6.40	0.403	21.0	62.9	33.3	12.2	2.2	142	6.48	1.09	4.62	0.04	0.19	0.52
	SD	0.8	0.28	0.014	0.6	1.0	0.8	1.0	0.1	13	1.24	0.46	0.72	0.01	0.05	0.10
	n	3	3	3	3	3	3	3	3	3	3	3	3	3	3	3

Significantly different from Group 1:a=p<0.05,b=p<0.01,c=p<0.001

Table 7 (continued)
Haematology and Coagulation: Group Mean Values: Day 92

Group	:	1	2	3
Test Item	:	Control	MenPF-1	MenPF-1
Dosage (µg/dose)	:	0	25	50

Group / sex		LUC -----------	Plat $x10^9/L$	PT s	APTT s	Fib mg/dL
1M	Mean	0.01	354	6.1	56.9	176
	SD	0.01	85	0.3	3.7	11
	n	3	3	3	3	3
2M	Mean	0.02	396	5.9	57.4	184
	SD	0.01	63	0.2	1.9	24
	n	2	2	3	3	3
3M	Mean	0.01	398	6.0	56.5	180
	SD	0.01	26	0.3	1.7	26
	n	3	3	3	3	3

Significantly different from Group 1:a=p<0.05,b=p<0.01,c=p<0.001

Table 7 (continued)
Haematology and Coagulation: Group Mean Values: Day 92

Group	:	1	2	3
Test Item	:	Control	MenPF-1	MenPF-1
Dosage (µg/dose)	:	0	25	50

Group / sex		Hb g/dL	RBC $x10^{12}$/L	Hct L/L	MCH pg	MCV fL	MCHC g/dL	RDW %	Reti %	Ret $x10^{9}$/L	WBC -----------	Neut -----------	Lymph -----------	Mono $x10^{9}$/L	Eos -----------	Baso -----------
1F	Mean	12.6	5.98	0.385	21.2	64.4	32.8	11.6	2.4	143	5.78	1.26	3.80	0.05	0.20	0.46
	SD	0.7	0.43	0.021	0.4	1.1	0.3	0.4	0.1	17	0.82	0.65	0.60	0.03	0.10	0.13
	n	3	3	3	3	3	3	3	3	3	3	3	3	3	3	3
2F	Mean	12.9	6.03	0.387	21.4	64.2	33.3	11.9	2.1	129	5.04	0.78	3.67	0.03	0.14	0.42
	SD	0.9	0.57	0.032	1.2	3.6	0.6	1.0	0.4	31	2.45	0.55	1.77	0.02	0.06	0.18
	n	3	3	3	3	3	3	3	3	3	3	3	3	3	3	3
3F	Mean	12.4	5.87	0.377	21.2	64.5	33.0	12.0	2.3	135	5.36	1.19	3.48	0.06	0.14	0.47
	SD	0.5	0.46	0.016	1.0	3.0	0.1	0.7	0.4	33	0.94	0.21	0.67	0.03	0.01	0.18
	n	3	3	3	3	3	3	3	3	3	3	3	3	3	3	3

Significantly different from Group 1:a=p<0.05,b=p<0.01,c=p<0.001

Table 7 (continued)
Haematology and Coagulation: Group Mean Values: Day 92

Group	:	1	2	3
Test Item	:	Control	MenPF-1	MenPF-1
Dosage (µg/dose)	:	0	25	50

Group / sex		LUC -----------	Plat x10⁹/L	PT s	APTT s	Fib mg/dL
1F	Mean	0.01	422	5.9	56.4	133
	SD	0.01	46	0.1	1.1	19
	n	3	3	3	3	3
2F	Mean	0.01	247	5.8	58.0	132
	SD	0.01	216	0.0	9.3	1
	n	3	3	2	2	2
3F	Mean	0.01	450	5.8	53.6	157
	SD	0.01	21	0.2	3.9	26
	n	3	3	3	3	3

Significantly different from Group 1:a=p<0.05,b=p<0.01,c=p<0.001

Table 8
Clinical Chemistry : Group Mean Values: Pretrial

Group	:	1	2	3
Test Item	:	Control	MenPF-1	MenPF-1
Dosage (µg/dose)	:	0	25	50

Group / sex		ALP U/L	ALT U/L	AST U/L	LDH U/L	CPK U/L	Urea mmol/L	Glu mmol/L	T.Bil µmol/L	Chol mmol/L	TP g/L	Alb g/L	Glob g/L	AG-R	Na mmol/L	K mmol/L
1M	Mean	164	26	11	70	633	7.1	8.01	1.7	0.8	54	42	13	3.3	142	4.6
	SD	43	8	2	17	203	0.5	0.40	0.0	0.3	3	2	1	0.3	2	0.2
	n	6	6	6	6	6	6	6	6	6	6	6	6	6	6	6
2M	Mean	153	29	11	60	496	7.4	8.15	1.7	0.7	58[a]	44	14	3.1	141	4.6
	SD	13	7	1	9	134	0.5	0.17	0.0	0.2	2	1	1	0.2	1	0.3
	n	6	6	6	6	6	6	6	6	6	6	6	6	6	6	6
3M	Mean	192	25	11	62	598	7.1	7.98	1.7	0.9	56	43	14	3.2	143	4.4
	SD	33	7	1	17	165	0.4	0.33	0.0	0.2	2	1	1	0.2	2	0.1
	n	6	6	6	6	6	6	6	6	6	6	6	6	6	6	6

Significantly different from Group 1:a=p<0.05,b=p<0.01,c=p<0.001

Table 8 (continued)
Clinical Chemistry : Group Mean Values: Pretrial

Group	:	1	2	3
Test Item	:	Control	MenPF-1	MenPF-1
Dosage (µg/dose)	:	0	25	50

Group / sex		Cl mmol/L	Phos mmol/L	Ca mmol/L	Crea µmol/L
1M	Mean	104	1.86	3.63	64
	SD	2	0.15	0.09	12
	n	6	6	6	6
2M	Mean	100	1.87	3.75[a]	60
	SD	2	0.15	0.04	10
	n	6	6	6	6
3M	Mean	102	1.94	3.69	64
	SD	2	0.18	0.09	3
	n	6	6	6	6

Significantly different from Group 1:a=p<0.05,b=p<0.01,c=p<0.001

Table 8 (continued)
Clinical Chemistry : Group Mean Values: Pretrial

Group	:	1	2	3
Test Item	:	Control	MenPF-1	MenPF-1
Dosage (µg/dose)	:	0	25	50

Group / sex		ALP U/L	ALT U/L	AST U/L	LDH U/L	CPK U/L	Urea mmol/L	Glu mmol/L	T.Bil µmol/L	Chol mmol/L	TP g/L	Alb g/L	Glob g/L	AG-R	Na mmol/L	K mmol/L
1F	Mean	232	23	11	70	961	7.0	7.71	1.7	1.3	55	42	14	3.1	142	4.4
	SD	64	7	2	4	724	1.1	0.56	0.0	0.3	2	2	1	0.3	1	0.1
	n	6	6	6	6	6	6	6	6	6	6	6	6	6	6	6
2F	Mean	219	29	11	69	555	6.7	8.56[a]	1.7	1.3	54	42	13	3.2	141	4.2
	SD	39	9	2	17	199	0.5	0.45	0.0	0.3	4	3	1	0.2	1	0.3
	n	6	6	6	6	6	6	6	6	6	6	6	6	6	6	6
3F	Mean	174	29	12	58	879	7.1	8.64[b]	1.7	1.6	59[a]	45[a]	14	3.3	141	4.3
	SD	23	9	2	6	392	1.1	0.57	0.0	0.4	2	1	1	0.3	1	0.3
	n	6	6	6	6	6	6	6	6	6	6	6	6	6	6	6

Significantly different from Group 1:a=p<0.05,b=p<0.01,c=p<0.001

Table 8 (continued)
Clinical Chemistry : Group Mean Values: Pretrial

Group	:	1	2	3
Test Item	:	Control	MenPF-1	MenPF-1
Dosage (µg/dose)	:	0	25	50

Group / sex		Cl mmol/L	Phos mmol/L	Ca mmol/L	Crea µmol/L
1F	Mean	102	1.95	3.66	60
	SD	3	0.29	0.05	6
	n	6	6	6	6
2F	Mean	100	2.04	3.61	63
	SD	3	0.11	0.15	2
	n	6	6	6	6
3F	Mean	100	1.99	3.72	59
	SD	2	0.26	0.05	9
	n	6	6	6	6

Significantly different from Group 1:a=p<0.05,b=p<0.01,c=p<0.001

Table 9
Clinical Chemistry : Group Mean Values: Day 66

Group	:	1	2	3
Test Item	:	Control	MenPF-1	MenPF-1
Dosage (µg/dose)	:	0	25	50

Group / sex		ALP U/L	ALT U/L	AST U/L	LDH U/L	CPK U/L	Urea mmol/L	Glu mmol/L	T.Bil µmol/L	Chol mmol/L	TP g/L	Alb g/L	Glob g/L	AG-R	Na mmol/L	K mmol/L
1M	Mean	62	37	14	56	610	7.5	7.52	1.7	0.4	57	45	12	3.9	144	4.6
	SD	11	17	4	7	159	1.1	0.57	0.0	0.1	3	2	1	0.3	1	0.3
	n	6	6	6	6	6	6	6	6	6	6	6	6	6	6	6
2M	Mean	62	39	13	59	528	7.6	7.26	1.7	0.6	61[b]	46	15[c]	3.0[c]	144	4.6
	SD	9	12	4	18	164	1.1	0.30	0.0	0.2	1	2	2	0.5	2	0.2
	n	6	6	6	6	6	6	6	6	6	6	6	6	6	6	6
3M	Mean	71	42	14	84	760	6.8	7.50	1.7	0.5	61[b]	46	15[c]	3.0[c]	146	4.3[a]
	SD	16	22	4	56	576	0.5	0.45	0.0	0.1	2	2	1	0.1	1	0.2
	n	6	6	6	6	6	6	6	6	6	6	6	6	6	6	6

Significantly different from Group 1:a=p<0.05,b=p<0.01,c=p<0.001

Table 9 (continued)
Clinical Chemistry : Group Mean Values: Day 66

Group	:	1	2	3
Test Item	:	Control	MenPF-1	MenPF-1
Dosage (µg/dose)	:	0	25	50

Group / sex		Cl mmol/L	Phos mmol/L	Ca mmol/L	Crea µmol/L
1M	Mean	107	1.27	3.60	67
	SD	1	0.10	0.09	13
	n	6	6	6	6
2M	Mean	105	1.37	3.70	62
	SD	2	0.10	0.04	16
	n	6	6	6	6
3M	Mean	105	1.36	3.65	68
	SD	2	0.11	0.10	6
	n	6	6	6	6

Significantly different from Group 1:a=p<0.05,b=p<0.01,c=p<0.001

Table 9 (continued)
Clinical Chemistry : Group Mean Values: Day 66

Group	:	1	2	3
Test Item	:	Control	MenPF-1	MenPF-1
Dosage (µg/dose)	:	0	25	50

Group / sex		ALP U/L	ALT U/L	AST U/L	LDH U/L	CPK U/L	Urea mmol/L	Glu mmol/L	T.Bil µmol/L	Chol mmol/L	TP g/L	Alb g/L	Glob g/L	AG-R	Na mmol/L	K mmol/L
1F	Mean	104	35	10	47	764	7.8	7.09	1.7	1.3	58	46	13	3.7	144	4.5
	SD	49	21	2	12	356	1.8	0.49	0.0	0.1	1	1	1	0.4	2	0.4
	n	6	6	6	6	6	6	6	6	6	6	6	6	6	6	6
2F	Mean	76	45	14[a]	60	651	8.0	7.58	1.7	1.2	58	45	14	3.3	145	4.2
	SD	16	15	2	17	264	0.9	0.39	0.0	0.2	3	2	2	0.3	1	0.2
	n	6	6	6	6	6	6	6	6	6	6	6	6	6	6	6
3F	Mean	84	59	17[b]	44	623	7.6	7.41	1.8	1.8	62[b]	46	16[c]	3.0[b]	144	4.3
	SD	17	33	8	11	159	1.2	0.44	0.2	0.8	2	2	1	0.2	2	0.3
	n	6	6	6	6	6	6	6	6	6	6	6	6	6	6	6

Significantly different from Group 1:a=p<0.05,b=p<0.01,c=p<0.001

Table 9 (continued)
Clinical Chemistry : Group Mean Values: Day 66

Group	:	1	2	3
Test Item	:	Control	MenPF-1	MenPF-1
Dosage (µg/dose)	:	0	25	50

Group / sex		Cl mmol/L	Phos mmol/L	Ca mmol/L	Crea µmol/L
1F	Mean	104	1.44	3.70	74
	SD	2	0.16	0.05	10
	n	6	6	6	6
2F	Mean	103	1.55	3.62	82
	SD	2	0.21	0.18	16
	n	6	6	6	6
3F	Mean	103	1.45	3.64	76
	SD	2	0.17	0.08	8
	n	6	6	6	6

Significantly different from Group 1:a=p<0.05,b=p<0.01,c=p<0.001

Table 10
Clinical Chemistry : Group Mean Values: Day 92

Group	:	1	2	3
Test Item	:	Control	MenPF-1	MenPF-1
Dosage (µg/dose)	:	0	25	50

Group / sex		ALP U/L	ALT U/L	AST U/L	LDH U/L	CPK U/L	Urea mmol/L	Glu mmol/L	T.Bil µmol/L	Chol mmol/L	TP g/L	Alb g/L	Glob g/L	AG-R	Na mmol/L	K mmol/L
1M	Mean	55	26	14	64	506	7.0	6.62	1.7	0.3	56	44	12	3.8	145	4.0
	SD	8	12	4	5	88	0.8	0.93	0.0	0.1	1	1	1	0.3	2	0.5
	n	3	3	3	3	3	3	3	3	3	3	3	3	3	3	3
2M	Mean	50	39	14	73	481	8.2	6.58	1.7	0.5	59	47[a]	12	4.0	145	4.6[a]
	SD	6	6	3	21	95	1.4	0.04	0.0	0.1	2	2	1	0.2	2	0.0
	n	3	3	3	3	3	3	3	3	3	3	3	3	3	3	3
3M	Mean	102	32	15	72	441	6.6	6.98	1.7	0.5	57	45	12	3.7	144	4.3
	SD	68	17	7	10	51	0.7	0.42	0.0	0.2	1	1	1	0.3	1	0.1
	n	3	3	3	3	3	3	3	3	3	3	3	3	3	3	3

Significantly different from Group 1:a=p<0.05,b=p<0.01,c=p<0.001

Table 10 (continued)
Clinical Chemistry : Group Mean Values: Day 92

Group	:	1	2	3
Test Item	:	Control	MenPF-1	MenPF-1
Dosage (µg/dose)	:	0	25	50

Group / sex		Cl mmol/L	Phos mmol/L	Ca mmol/L	Crea µmol/L
1M	Mean	105	1.19	3.60	67
	SD	1	0.14	0.13	8
	n	3	3	3	3
2M	Mean	105	1.13	3.88[b]	75
	SD	2	0.11	0.07	24
	n	3	3	3	3
3M	Mean	103	1.23	3.60	74
	SD	2	0.03	0.04	4
	n	3	3	3	3

Significantly different from Group 1:a=p<0.05,b=p<0.01,c=p<0.001

Table 10 (continued)
Clinical Chemistry : Group Mean Values: Day 92

Group	:	1	2	3
Test Item	:	Control	MenPF-1	MenPF-1
Dosage (µg/dose)	:	0	25	50

Group / sex		ALP U/L	ALT U/L	AST U/L	LDH U/L	CPK U/L	Urea mmol/L	Glu mmol/L	T.Bil µmol/L	Chol mmol/L	TP g/L	Alb g/L	Glob g/L	AG-R	Na mmol/L	K mmol/L
1F	Mean	82	33	11	58	551	9.2	6.32	1.7	1.3	60	48	12	4.0	144	4.4
	SD	18	4	2	19	189	1.1	0.41	0.0	0.1	1	2	1	0.3	1	0.1
	n	3	3	3	3	3	3	3	3	3	3	3	3	3	3	3
2F	Mean	62	39	17[b]	79	493	9.0	7.26[b]	1.7	1.0	55[b]	43[b]	12	3.7	143	4.3
	SD	4	10	3	71	102	1.0	0.28	0.0	0.2	2	1	1	0.2	2	0.1
	n	3	3	3	3	3	3	3	3	3	3	3	3	3	3	3
3F	Mean	87	32	14	42	393	8.7	6.51	1.7	1.6	62	47	14[a]	3.3	143	4.2
	SD	25	9	1	7	112	1.0	0.19	0.0	1.1	1	2	1	0.3	1	0.2
	n	3	3	3	3	3	3	3	3	3	3	3	3	3	3	3

Significantly different from Group 1:a=p<0.05,b=p<0.01,c=p<0.001

Table 10 (continued)
Clinical Chemistry : Group Mean Values: Day 92

Group	:	1	2	3
Test Item	:	Control	MenPF-1	MenPF-1
Dosage (µg/dose)	:	0	25	50

Group / sex		Cl mmol/L	Phos mmol/L	Ca mmol/L	Crea µmol/L
1F	Mean	104	1.16	3.78	83
	SD	3	0.15	0.04	9
	n	3	3	3	3
2F	Mean	104	1.35	3.49[b]	100
	SD	3	0.16	0.02	22
	n	3	3	3	3
3F	Mean	104	1.18	3.70	81
	SD	1	0.15	0.15	13
	n	3	3	3	3

Significantly different from Group 1:a=p<0.05,b=p<0.01,c=p<0.001

Table 11
Summary of Necropsy Findings: Day 66

		1	2	3
Group	:	1	2	3
Test Item	:	Control	MenPF-1	MenPF-1
Dosage (µg/dose)	:	0	25	50

NECROPSY FINDINGS	GROUP	GROUP TOTALS Males Grp 1	Grp 2	Grp 3	Females Grp 1	Grp 2	Grp 3
GENERAL COMMENTS							
Number of animals necropsied		3	3	3	3	3	3
LUNG							
Spongy					1	1	
Discolouration		3	3	2	3	2	2
LYMPH NODE (LUMBAR)							
Discolouration, one/both			1		1		1
Enlargement, left				2			1
LYMPH NODE (MESENTERIC)							
Discolouration					1	1	1
OVIDUCT							
Cyst, right							1

The absence of a numeral indicates that the lesion specified was not identified

Pathology File Ref.: PLAFOR_520419_MACMAIN_LL_KEEP2.SPL

Table 11 (continued)
Summary of Necropsy Findings: Day 66

Group	:	1	2	3
Test Item	:	Control	MenPF-1	MenPF-1
Dosage (µg/dose)	:	0	25	50

		GROUP TOTALS					
		Males			Females		
NECROPSY FINDINGS	GROUP	Grp 1	Grp 2	Grp 3	Grp 1	Grp 2	Grp 3
TESTIS							
Small, right		2					
THYMUS							
Discolouration		1					

The absence of a numeral indicates that the lesion specified was not identified

Pathology File Ref.: PLAFOR_520419_MACMAIN_LL_KEEP2.SPL

Table 12
Summary of Necropsy Findings: Day 92

Group	:	1	2	3
Test Item	:	Control	MenPF-1	MenPF-1
Dosage (µg/dose)	:	0	25	50

NECROPSY FINDINGS	GROUP	INCIDENCE Males Grp 1	Males Grp 2	Males Grp 3	Females Grp 1	Females Grp 2	Females Grp 3
GENERAL COMMENTS							
Number of animals necropsied		3	3	3	3	3	3
ADRENAL GLAND							
Discolouration, both		1					
LUNG							
Spongy		1		1	2	2	2
Discolouration		2	3	2	3	2	2
LYMPH NODE (INGUINAL)							
Discolouration, right				1			
LYMPH NODE (LUMBAR)							
Discolouration, one/both		1		3			

The absence of a numeral indicates that the lesion specified was not identified

Pathology File Ref.: PLAFOR_520419_MACREC_LL_KEEP1.SPL

Table 12
Summary of Necropsy Findings: Day 92

Group	:	1	2	3
Test Item	:	Control	MenPF-1	MenPF-1
Dosage (µg/dose)	:	0	25	50

NECROPSY FINDINGS	GROUP	INCIDENCE					
		Males			Females		
		Grp 1	Grp 2	Grp 3	Grp 1	Grp 2	Grp 3
LYMPH NODE (MANDIBULAR)							
Discolouration			1				1
OVARY							
Foci, dark, both						1	
OVIDUCT							
Cyst, right						1	
THYROID GLAND							
Small, right				1			
TRACHEA							
Fluid accumulation		2	1	1	2	2	2

The absence of a numeral indicates that the lesion specified was not identified

Pathology File Ref.: PLAFOR_520419_MACREC_LL_KEEP1.SPL

Table 13
Absolute Organ Weights (g) : Group Mean Values: Day 66

Group	:	1	2	3
Test Item	:	Control	MenPF-1	MenPF-1
Dosage (µg/dose)	:	0	25	50

Group / sex		Body Weight (kg)	Adrenals	Brain	Epididy-mides	Heart	Kidneys	Liver	Lung	Pituitary	Prostate
1M	Mean	3.2	0.2992	10.19	2.1413	8.96	18.87	98.37	22.54	0.030	0.97
	SD	0.1	0.0543	0.54	0.1608	1.13	2.12	25.11	1.60	0.001	0.49
	n	3	3	3	3	3	3	3	3	2	3
2M	Mean	3.4	0.3112	9.72	2.4714	9.85	21.88	140.20	27.85	0.039	1.30
	SD	0.1	0.0348	0.23	0.5107	0.91	2.64	24.51	3.39	0.006	0.26
	n	3	3	3	3	3	3	3	3	3	3
3M	Mean	3.4	0.2538	9.71	2.4485	8.79	19.20	112.52	22.51	0.026	0.88
	SD	0.2	0.0017	0.16	0.4247	0.66	1.29	20.63	6.36	0.001	0.15
	n	3	2	3	3	3	3	3	3	3	3

Significantly different from Group 1:a=p<0.05,b=p<0.01,c=p<0.001

Table 13 (continued)
Absolute Organ Weights (g) : Group Mean Values: Day 66

Group	:	1	2	3
Test Item	:	Control	MenPF-1	MenPF-1
Dosage (µg/dose)	:	0	25	50

Group / sex		Spleen	Testes	Thymus	Thyroid
1M	Mean	1.053	4.40	2.863	0.406
	SD	0.137	1.32	0.777	0.108
	n	3	3	3	3
2M	Mean	1.065	5.63	3.076	0.253
	SD	0.063	0.78	0.209	0.042
	n	3	3	3	3
3M	Mean	1.295	5.56	3.532	0.311
	SD	0.136	0.74	0.673	0.071
	n	3	3	3	3

Significantly different from Group 1:a=p<0.05,b=p<0.01,c=p<0.001

Table 13 (continued)
Absolute Organ Weights (g) : Group Mean Values: Day 66

Group	:	1	2	3
Test Item	:	Control	MenPF-1	MenPF-1
Dosage (µg/dose)	:	0	25	50

Group / sex		Body Weight (kg)	Adrenals	Brain	Heart	Kidneys	Liver	Lung	Ovaries	Pituitary	Spleen
1F	Mean	4.0	0.2487	10.29	9.74	21.80	130.57	27.93	0.380	0.039	1.870
	SD	0.1	0.0443	0.57	1.79	2.64	16.28	0.60	0.026	0.015	0.413
	n	3	3	3	3	3	3	3	3	3	3
2F	Mean	3.9	0.3442[a]	9.68	9.97	20.27	123.04	21.51	0.438	0.036	1.800
	SD	0.4	0.0331	0.36	1.11	3.54	47.05	8.16	0.114	0.005	0.273
	n	3	3	3	3	3	3	3	3	3	3
3F	Mean	3.8	0.3295[a]	9.57	9.09	22.36	129.88	21.31	0.504	0.031	1.387
	SD	0.6	0.0390	0.26	1.90	5.57	48.84	10.72	0.144	0.010	0.354
	n	3	3	3	3	3	3	3	3	3	3

Significantly different from Group 1:a=p<0.05,b=p<0.01,c=p<0.001

Table 13 (continued)
Absolute Organ Weights (g) : Group Mean Values: Day 66

Group	:	1	2	3
Test Item	:	Control	MenPF-1	MenPF-1
Dosage (µg/dose)	:	0	25	50

Group / sex		Thymus	Thyroid	Uterus
1F	Mean	3.100	0.363	8.05
	SD	0.790	0.083	0.76
	n	3	3	3
2F	Mean	3.542	0.426	10.28
	SD	0.722	0.050	1.58
	n	3	3	3
3F	Mean	3.613	0.403	8.36
	SD	1.302	0.042	1.26
	n	3	3	3

Significantly different from Group 1:a=p<0.05,b=p<0.01,c=p<0.001

Table 14
Organ Weights (Covariance Analysis): Group Mean Values: Day 66

Group	:	1	2	3
Test Item	:	Control	MenPF-1	MenPF-1
Dosage (µg/dose)	:	0	25	50

Group / sex		Adrenals	Brain	Epididy- mides	Heart	Kidneys	Liver	Lung	Pituitary	Prostate	Spleen
1M	Mean	0.2968	10.12	2.2396	8.94	19.66	106.23	21.44	0.031	1.07	1.03
	SE	0.0322	0.26	0.2893	0.70	1.46	16.70	3.12	0.004	0.24	0.09
	n	3	3	3	3	3	3	3	2	3	3
2M	Mean	0.3136	9.76	2.4223	9.86	21.48	136.27	28.40	0.038	1.25	1.08
	SE	0.0322	0.23	0.2537	0.61	1.28	14.65	2.73	0.003	0.21	0.08
	n	3	3	3	3	3	3	3	3	3	3
3M	Mean	0.2538	9.75	2.3994	8.80	18.80	108.59	23.06	0.026	0.83	1.31
	SE	0.0322	0.23	0.2537	0.61	1.28	14.65	2.73	0.003	0.21	0.08
	n	2	3	3	3	3	3	3	3	3	3

Significantly different from Group 1:a=p<0.05,b=p<0.01,c=p<0.001

Table 14 (continued)
Organ Weights (Covariance Analysis): Group Mean Values: Day 66

Group	:	1	2	3
Test Item	:	Control	MenPF-1	MenPF-1
Dosage (µg/dose)	:	0	25	50

Group / sex		Testes	Thymus	Thyroid
1M	Mean	4.04	3.09	0.423
	SE	0.69	0.43	0.058
	n	3	3	3
2M	Mean	5.81	2.96	0.245
	SE	0.60	0.37	0.051
	n	3	3	3
3M	Mean	5.74	3.42	0.303
	SE	0.60	0.37	0.051
	n	3	3	3

Significantly different from Group 1:a=p<0.05,b=p<0.01,c=p<0.001

Table 14 (continued)
Organ Weights (Covariance Analysis): Group Mean Values: Day 66

Group	:	1	2	3
Test Item	:	Control	MenPF-1	MenPF-1
Dosage (µg/dose)	:	0	25	50

Group / sex		Adrenals	Brain	Heart	Kidneys	Liver	Lung	Ovaries	Pituitary	Spleen	Thymus
1F	Mean	0.2505	10.29	9.56	21.22	124.35	26.98	0.367	0.040	1.88	2.99
	SE	0.0238	0.26	0.77	1.28	7.77	3.26	0.045	0.006	0.22	0.42
	n	3	3	3	3	3	3	3	3	3	3
2F	Mean	0.3442	9.68	9.97	20.27	123.04	21.51	0.438	0.036	1.80	3.54
	SE	0.0237	0.26	0.76	1.27	7.72	3.24	0.045	0.006	0.22	0.42
	n	3	3	3	3	3	3	3	3	3	3
3F	Mean	0.3277	9.58	9.27	22.94	136.11	22.26	0.517	0.031	1.38	3.73
	SE	0.0238	0.26	0.77	1.28	7.77	3.26	0.045	0.006	0.22	0.42
	n	3	3	3	3	3	3	3	3	3	3

Significantly different from Group 1:a=p<0.05,b=p<0.01,c=p<0.001

Table 14 (continued)
Organ Weights (Covariance Analysis): Group Mean Values: Day 66

Group	:	1	2	3
Test Item	:	Control	MenPF-1	MenPF-1
Dosage (µg/dose)	:	0	25	50

Group / sex		Thyroid	Uterus
1F	Mean	0.368	7.99
	SE	0.033	0.75
	n	3	3
2F	Mean	0.426	10.28
	SE	0.033	0.75
	n	3	3
3F	Mean	0.398	8.43
	SE	0.033	0.75
	n	3	3

Significantly different from Group 1:a=p<0.05,b=p<0.01,c=p<0.001

Table 15
Relative Organ Weights (% Body Weight): Group Mean Values: Day 66

Group	:	1	2	3
Test Item	:	Control	MenPF-1	MenPF-1
Dosage (µg/dose)	:	0	25	50

Group / sex		Adrenals	Brain	Epididy- mides	Heart	Kidneys	Liver	Lung	Pituitary	Prostate	Spleen
1M	Mean	0.0093	0.319	0.06697	0.281	0.589	3.073	0.706	0.0010	0.030	0.0330
	SD	0.0016	0.027	0.00552	0.044	0.061	0.775	0.072	0.0001	0.014	0.0045
	n	3	3	3	3	3	3	3	2	3	3
2M	Mean	0.0092	0.286	0.07293	0.290	0.643	4.132	0.818	0.0011	0.038	0.0313
	SD	0.0012	0.007	0.01633	0.030	0.075	0.792	0.087	0.0002	0.008	0.0022
	n	3	3	3	3	3	3	3	3	3	3
3M	Mean	0.0063	0.287	0.07171	0.259	0.565	3.293	0.667	0.0008	0.026	0.0382
	SD	0.0024	0.016	0.00832	0.007	0.024	0.415	0.214	0.0000	0.003	0.0051
	n	3	3	3	3	3	3	3	3	3	3

Significantly different from Group 1:a=p<0.05,b=p<0.01,c=p<0.001

Table 15 (continued)
Relative Organ Weights (% Body Weight): Group Mean Values: Day 66

Group	:	1	2	3
Test Item	:	Control	MenPF-1	MenPF-1
Dosage (µg/dose)	:	0	25	50

Group / sex		Testes	Thymus	Thyroid
1M	Mean	0.137	0.090	0.0127
	SD	0.041	0.024	0.0030
	n	3	3	3
2M	Mean	0.166	0.090	0.0074
	SD	0.028	0.007	0.0014
	n	3	3	3
3M	Mean	0.164	0.104	0.0092
	SD	0.029	0.017	0.0020
	n	3	3	3

Significantly different from Group 1:a=p<0.05,b=p<0.01,c=p<0.001

Table 15 (continued)
Relative Organ Weights (% Body Weight): Group Mean Values: Day 66

Group	:	1	2	3
Test Item	:	Control	MenPF-1	MenPF-1
Dosage (µg/dose)	:	0	25	50

Group / sex		Adrenals	Brain	Heart	Kidneys	Liver	Lung	Ovaries	Pituitary	Spleen	Thymus
1F	Mean	0.0063	0.260	0.245	0.549	3.291	0.705	0.0096	0.0010	0.0472	0.078
	SD	0.0009	0.021	0.038	0.054	0.401	0.034	0.0006	0.0004	0.0106	0.017
	n	3	3	3	3	3	3	3	3	3	3
2F	Mean	0.0090	0.250	0.256	0.519	3.097	0.547	0.0111	0.0009	0.0461	0.090
	SD	0.0017	0.030	0.018	0.060	0.823	0.180	0.0016	0.0002	0.0028	0.008
	n	3	3	3	3	3	3	3	3	3	3
3F	Mean	0.0088	0.253	0.237	0.578	3.306	0.540	0.0131	0.0009	0.0377	0.094
	SD	0.0019	0.032	0.031	0.070	0.865	0.217	0.0028	0.0004	0.0158	0.025
	n	3	3	3	3	3	3	3	3	3	3

Significantly different from Group 1:a=p<0.05,b=p<0.01,c=p<0.001

Table 15 (continued)
Relative Organ Weights (% Body Weight): Group Mean Values: Day 66

Group	:	1	2	3
Test Item	:	Control	MenPF-1	MenPF-1
Dosage (µg/dose)	:	0	25	50

Group / sex		Thyroid	Uterus
1F	Mean	0.0092	0.203
	SD	0.0023	0.020
	n	3	3
2F	Mean	0.0111	0.266
	SD	0.0023	0.055
	n	3	3
3F	Mean	0.0107	0.219
	SD	0.0024	0.012
	n	3	3

Significantly different from Group 1:a=p<0.05,b=p<0.01,c=p<0.001

Table 16
Absolute Organ Weights (g) : Group Mean Values: Day 92

Group	:	1	2	3
Test Item	:	Control	MenPF-1	MenPF-1
Dosage (µg/dose)	:	0	25	50

Group / sex		Body Weight (kg)	Adrenals	Brain	Epididy-mides	Heart	Kidneys	Liver	Lung	Pituitary	Prostate
1M	Mean	3.5	0.2622	10.11	2.1997	9.68	20.37	108.86	23.40	0.020	0.99
	SD	0.2	0.0381	0.38	0.2994	0.85	1.14	4.18	7.25	0.007	0.12
	n	3	3	3	3	3	3	3	3	3	3
2M	Mean	3.5	0.3237	9.75	1.9825	9.20	17.95	115.60	28.66	0.022	0.80
	SD	0.2	0.0305	0.60	0.3467	0.70	4.12	29.19	4.39	0.002	0.13
	n	3	3	3	3	3	3	3	3	3	3
3M	Mean	3.3	0.3486	10.17	2.1297	8.37	16.75	92.85	23.18	0.030	1.06
	SD	0.3	0.0588	0.24	0.3844	0.24	0.75	13.30	9.49	0.015	0.36
	n	3	3	3	3	3	3	3	3	3	3

Significantly different from Group 1:a=p<0.05,b=p<0.01,c=p<0.001

Table 16 (continued)
Absolute Organ Weights (g) : Group Mean Values: Day 92

Group	:	1	2	3
Test Item	:	Control	MenPF-1	MenPF-1
Dosage (µg/dose)	:	0	25	50

Group / sex		Spleen	Testes	Thymus	Thyroid
1M	Mean	1.006	6.35	3.437	0.252
	SD	0.432	0.63	0.927	0.094
	n	3	3	3	3
2M	Mean	1.185	5.14	3.175	0.276
	SD	0.083	0.82	1.546	0.084
	n	3	3	3	3
3M	Mean	1.151	5.42	2.733	0.207
	SD	0.324	0.34	0.887	0.024
	n	3	3	3	3

Significantly different from Group 1:a=p<0.05,b=p<0.01,c=p<0.001

Table 16 (continued)
Absolute Organ Weights (g) : Group Mean Values: Day 92

Group	:	1	2	3
Test Item	:	Control	MenPF-1	MenPF-1
Dosage (µg/dose)	:	0	25	50

Group / sex		Body Weight (kg)	Adrenals	Brain	Heart	Kidneys	Liver	Lung	Ovaries	Pituitary	Spleen
1F	Mean	4.5	0.3051	9.72	9.90	22.16	129.33	31.06	0.492	0.026	1.828
	SD	0.4	0.0517	0.77	1.27	2.95	14.95	8.26	0.093	0.009	0.319
	n	3	3	3	3	3	3	3	3	3	3
2F	Mean	3.9	0.2566	9.49	8.61	18.25	95.22[b]	22.69	0.395	0.031	1.808
	SD	0.4	0.0403	0.68	0.36	1.57	7.81	5.28	0.134	0.012	0.547
	n	3	3	3	3	3	3	3	3	3	3
3F	Mean	4.3	0.3020	9.47	10.53	20.53	139.77	30.24	0.538	0.021	1.310
	SD	0.2	0.0507	0.76	1.16	1.88	9.23	8.48	0.180	0.015	0.209
	n	3	3	3	3	3	3	3	3	3	3

Significantly different from Group 1:a=p<0.05,b=p<0.01,c=p<0.001

Table 16 (continued)
Absolute Organ Weights (g) : Group Mean Values: Day 92

Group	:	1	2	3
Test Item	:	Control	MenPF-1	MenPF-1
Dosage (µg/dose)	:	0	25	50

Group / sex		Thymus	Thyroid	Uterus
1F	Mean	4.497	0.354	9.25
	SD	1.202	0.073	2.12
	n	3	3	3
2F	Mean	2.728	0.350	9.70
	SD	1.017	0.081	1.93
	n	3	3	3
3F	Mean	3.079	0.370	9.75
	SD	0.482	0.114	1.88
	n	3	3	3

Significantly different from Group 1:a=p<0.05,b=p<0.01,c=p<0.001

Table 17
Organ Weights (Covariance Analysis): Group Mean Values: Day 92

Group	:	1	2	3
Test Item	:	Control	MenPF-1	MenPF-1
Dosage (µg/dose)	:	0	25	50

Group / sex		Adrenals	Brain	Epididy-mides	Heart	Kidneys	Liver	Lung	Pituitary	Prostate	Spleen
1M	Mean	0.2741	10.07	2.2842	9.59	19.60	102.70	21.54	0.020	1.04	1.10
	SE	0.0244	0.29	0.1986	0.41	1.29	9.08	4.17	0.006	0.14	0.16
	n	3	3	3	3	3	3	3	3	3	3
2M	Mean	0.3285	9.74	2.0163	9.16	17.64	113.14	27.92	0.022	0.82	1.22
	SE	0.0233	0.27	0.1897	0.40	1.23	8.68	3.99	0.006	0.13	0.16
	n	3	3	3	3	3	3	3	3	3	3
3M	Mean	0.3319	10.22	2.0114	8.51	17.83	101.47	25.78	0.030	0.98	1.02
	SE	0.0256	0.30	0.2082	0.43	1.35	9.52	4.38	0.007	0.14	0.17
	n	3	3	3	3	3	3	3	3	3	3

Significantly different from Group 1:a=p<0.05,b=p<0.01,c=p<0.001

Table 17 (continued)
Organ Weights (Covariance Analysis): Group Mean Values: Day 92

Group	:	1	2	3
Test Item	:	Control	MenPF-1	MenPF-1
Dosage (µg/dose)	:	0	25	50

Group / sex		Testes	Thymus	Thyroid
1M	Mean	6.48	3.04	0.253
	SE	0.38	0.55	0.049
	n	3	3	3
2M	Mean	5.19	3.02	0.277
	SE	0.36	0.52	0.047
	n	3	3	3
3M	Mean	5.24	3.29	0.206
	SE	0.40	0.57	0.052
	n	3	3	3

Significantly different from Group 1:a=p<0.05,b=p<0.01,c=p<0.001

Table 17 (continued)
Organ Weights (Covariance Analysis): Group Mean Values: Day 92

Group	:	1	2	3
Test Item	:	Control	MenPF-1	MenPF-1
Dosage (µg/dose)	:	0	25	50

Group / sex		Adrenals	Brain	Heart	Kidneys	Liver	Lung	Ovaries	Pituitary	Spleen	Thymus
1F	Mean	0.2828	10.03	9.53	20.71	122.40	33.90	0.530	0.030	1.89	3.81
	SE	0.0272	0.44	0.64	0.80	4.59	4.66	0.094	0.008	0.27	0.23
	n	3	3	3	3	3	3	3	3	3	3
2F	Mean	0.2873	9.07	9.12	20.25	104.74	18.78	0.342	0.025	1.72	3.67
	SE	0.0298	0.49	0.70	0.88	5.03	5.11	0.103	0.009	0.29	0.25
	n	3	3	3	3	3	3	3	3	3	3
3F	Mean	0.2936	9.59	10.39	19.99	137.17	31.30	0.552	0.022	1.34	2.82[a]
	SE	0.0244	0.40	0.58	0.72	4.12	4.19	0.084	0.007	0.24	0.21
	n	3	3	3	3	3	3	3	3	3	3

Significantly different from Group 1:a=p<0.05,b=p<0.01,c=p<0.001

Table 17 (continued)
Organ Weights (Covariance Analysis): Group Mean Values: Day 92

Group	:	1	2	3
Test Item	:	Control	MenPF-1	MenPF-1
Dosage (µg/dose)	:	0	25	50

Group / sex		Thyroid	Uterus
1F	Mean	0.306	9.31
	SE	0.047	1.42
	n	3	3
2F	Mean	0.416	9.61
	SE	0.052	1.56
	n	3	3
3F	Mean	0.352	9.77
	SE	0.042	1.28
	n	3	3

Significantly different from Group 1:a=p<0.05,b=p<0.01,c=p<0.001

Table 18
Relative Organ Weights (% Body Weight): Group Mean Values: Day 92

Group	:	1	2	3
Test Item	:	Control	MenPF-1	MenPF-1
Dosage (µg/dose)	:	0	25	50

Group / sex		Adrenals	Brain	Epididy-mides	Heart	Kidneys	Liver	Lung	Pituitary	Prostate	Spleen
1M	Mean	0.0075	0.286	0.06248	0.274	0.578	3.090	0.662	0.0005	0.028	0.0289
	SD	0.0015	0.007	0.01013	0.026	0.038	0.235	0.206	0.0002	0.003	0.0136
	n	3	3	3	3	3	3	3	3	3	3
2M	Mean	0.0094	0.282	0.05753	0.266	0.515	3.310	0.828	0.0006	0.023	0.0343
	SD	0.0009	0.025	0.01246	0.010	0.085	0.633	0.128	0.0000	0.004	0.0043
	n	3	3	3	3	3	3	3	3	3	3
3M	Mean	0.0108	0.313	0.06591	0.258	0.514	2.835	0.700	0.0009	0.033	0.0356
	SD	0.0026	0.029	0.01558	0.027	0.028	0.255	0.259	0.0006	0.014	0.0111
	n	3	3	3	3	3	3	3	3	3	3

Significantly different from Group 1:a=p<0.05,b=p<0.01,c=p<0.001

Table 18 (continued)
Relative Organ Weights (% Body Weight): Group Mean Values: Day 92

Group	:	1	2	3
Test Item	:	Control	MenPF-1	MenPF-1
Dosage (µg/dose)	:	0	25	50

Group / sex		Testes	Thymus	Thyroid
1M	Mean	0.180	0.097	0.0072
	SD	0.023	0.020	0.0029
	n	3	3	3
2M	Mean	0.149	0.091	0.0079
	SD	0.030	0.042	0.0021
	n	3	3	3
3M	Mean	0.167	0.083	0.0064
	SD	0.023	0.022	0.0012
	n	3	3	3

Significantly different from Group 1:a=p<0.05,b=p<0.01,c=p<0.001

Table 18 (continued)
Relative Organ Weights (% Body Weight): Group Mean Values: Day 92

Group	:	1	2	3
Test Item	:	Control	MenPF-1	MenPF-1
Dosage (µg/dose)	:	0	25	50

Group / sex		Adrenals	Brain	Heart	Kidneys	Liver	Lung	Ovaries	Pituitary	Spleen	Thymus
1F	Mean	0.0068	0.218	0.219	0.491	2.871	0.705	0.0110	0.0006	0.0406	0.099
	SD	0.0005	0.036	0.013	0.020	0.100	0.238	0.0023	0.0003	0.0057	0.017
	n	3	3	3	3	3	3	3	3	3	3
2F	Mean	0.0066	0.247	0.223	0.473	2.470[a]	0.583	0.0104	0.0008	0.0477	0.069
	SD	0.0009	0.037	0.012	0.033	0.206	0.094	0.0044	0.0004	0.0174	0.020
	n	3	3	3	3	3	3	3	3	3	3
3F	Mean	0.0070	0.219	0.244	0.473	3.225[a]	0.705	0.0125	0.0005	0.0304	0.071
	SD	0.0012	0.020	0.035	0.030	0.105	0.226	0.0046	0.0003	0.0057	0.008
	n	3	3	3	3	3	3	3	3	3	3

Significantly different from Group 1:a=p<0.05,b=p<0.01,c=p<0.001

Table 18 (continued)
Relative Organ Weights (% Body Weight): Group Mean Values: Day 92

Group	:	1	2	3
Test Item	:	Control	MenPF-1	MenPF-1
Dosage (µg/dose)	:	0	25	50

Group / sex		Thyroid	Uterus
1F	Mean	0.0078	0.205
	SD	0.0014	0.043
	n	3	3
2F	Mean	0.0090	0.254
	SD	0.0013	0.067
	n	3	3
3F	Mean	0.0085	0.226
	SD	0.0024	0.052
	n	3	3

Significantly different from Group 1:a=p<0.05,b=p<0.01,c=p<0.001

Table 19
Summary of Histological Findings: Day 66

Group	:	1	2	3
Test Item	:	Control	MenPF-1	MenPF-1
Dosage (µg/dose)	:	0	25	50

		GROUP TOTALS			
		Males		Females	
HISTOLOGICAL FINDINGS	GROUP	Grp 1	Grp 3	Grp 1	Grp 3
ADRENAL GLAND		(3)	(3)	(3)	(3)
No abnormality detected		3	3	2	2
Diffuse cortical cell hypertrophy		0	0	0	1
Cortical vacuolated cell focus		0	0	1	0
AORTA		(3)	(3)	(3)	(3)
No abnormality detected		3	3	3	2
Mineralisation, medial		0	0	0	1
APPENDIX		(3)	(3)	(3)	(3)
No abnormality detected		3	3	3	3
BRAIN		(3)	(3)	(3)	(3)
No abnormality detected		3	3	3	3
CAECUM		(3)	(3)	(3)	(3)
No abnormality detected		3	3	3	3

Figures in brackets represent the number of animals from which this tissue was examined microscopically

Pathology File Ref.: PLAFOR_520419_MICMAIN_LBE_KEEP1.SPL

Table 19 (continued)
Summary of Histological Findings: Day 66

Group	:	1	2	3
Test Item	:	Control	MenPF-1	MenPF-1
Dosage (µg/dose)	:	0	25	50

		GROUP TOTALS			
		Males		Females	
HISTOLOGICAL FINDINGS	GROUP	Grp 1	Grp 3	Grp 1	Grp 3
CERVIX				(3)	(3)
No abnormality detected				3	3
COLON		(3)	(3)	(3)	(3)
No abnormality detected		3	3	3	3
DUODENUM		(3)	(3)	(3)	(3)
No abnormality detected		3	3	3	3
EPIDIDYMIS		(3)	(3)		
No abnormality detected		1	3		
Aspermia, unilateral		2	0		
EYE		(3)	(3)	(3)	(3)
No abnormality detected		3	3	3	3

Figures in brackets represent the number of animals from which this tissue was examined microscopically

Pathology File Ref.: PLAFOR_520419_MICMAIN_LBE_KEEP1.SPL

Table 19 (continued)
Summary of Histological Findings: Day 66

Group	:	1	2	3
Test Item	:	Control	MenPF-1	MenPF-1
Dosage (µg/dose)	:	0	25	50

		GROUP TOTALS			
		Males		Females	
HISTOLOGICAL FINDINGS	GROUP	Grp 1	Grp 3	Grp 1	Grp 3
FEMUR		(3)	(3)	(3)	(3)
No abnormality detected		3	3	3	3
GALL BLADDER		(3)	(3)	(3)	(3)
No abnormality detected		3	3	3	3
HEART		(3)	(3)	(3)	(3)
No abnormality detected		3	3	2	3
Inflammatory cell foci, myocardial		0	0	1	0
ILEUM		(3)	(3)	(3)	(3)
No abnormality detected		3	3	3	3
INJECTION SITE 1		(3)	(3)	(3)	(3)
Macrophage accumulation, intramuscular					
minimal		0	0	0	2
mild		0	1	2	0

Figures in brackets represent the number of animals from which this tissue was examined microscopically

Pathology File Ref.: PLAFOR_520419_MICMAIN_LBE_KEEP1.SPL

Table 19 (continued)
Summary of Histological Findings: Day 66

Group	:	1	2	3
Test Item	:	Control	MenPF-1	MenPF-1
Dosage (µg/dose)	:	0	25	50

HISTOLOGICAL FINDINGS	GROUP	GROUP TOTALS			
		Males		Females	
		Grp 1	Grp 3	Grp 1	Grp 3
INJECTION SITE 1		(3)	(3)	(3)	(3)
Macrophage accumulation, intramuscular					
moderate		3	2	1	1
Total Incidence		3	3	3	3
Inflammation, polymorphonuclear leukocytic					
mild		0	0	0	1
moderate		0	1	0	1
marked		0	1	0	0
Total Incidence		0	2	0	2
Inflammation, polymorphonuclear leukocytic, dermal, focal					
minimal		0	0	1	0
Total Incidence		0	0	1	0
Inflammation, mononuclear cell					
minimal		0	0	0	1
mild		1	1	2	0
moderate		1	0	0	0
Total Incidence		2	1	2	1
Myofibre necrosis					
minimal		0	0	1	0
mild		1	1	0	1
moderate		0	1	0	0

Figures in brackets represent the number of animals from which this tissue was examined microscopically

Table 19 (continued)
Summary of Histological Findings: Day 66

Group	:	1	2	3
Test Item	:	Control	MenPF-1	MenPF-1
Dosage (µg/dose)	:	0	25	50

HISTOLOGICAL FINDINGS	GROUP	GROUP TOTALS			
		Males		Females	
		Grp 1	Grp 3	Grp 1	Grp 3
INJECTION SITE 1		(3)	(3)	(3)	(3)
Myofibre necrosis					
Total Incidence		1	2	1	1
Regeneration, myofibre					
minimal		0	0	1	2
mild		0	1	0	0
Total Incidence		0	1	1	2
Fibrosis, interstitial					
minimal		0	0	0	1
mild		0	1	0	1
marked		0	1	0	0
Total Incidence		0	2	0	2
Mineralisation		0	0	0	1
JEJUNUM		(3)	(3)	(3)	(3)
No abnormality detected		3	3	3	3
KIDNEY		(3)	(3)	(3)	(3)
No abnormality detected		1	1	3	1

Figures in brackets represent the number of animals from which this tissue was examined microscopically

Pathology File Ref.: PLAFOR_520419_MICMAIN_LBE_KEEP1.SPL

Table 19 (continued)
Summary of Histological Findings: Day 66

Group	:	1	2	3
Test Item	:	Control	MenPF-1	MenPF-1
Dosage (µg/dose)	:	0	25	50

HISTOLOGICAL FINDINGS	GROUP	GROUP TOTALS			
		Males		Females	
		Grp 1	Grp 3	Grp 1	Grp 3
KIDNEY		(3)	(3)	(3)	(3)
Nephropathy, focal		0	1	0	0
Basophilic tubules		2	1	0	2
Tubular mineralisation		0	1	0	2
LACRIMAL GLAND		(3)	(2)	(2)	(3)
No abnormality detected		3	2	2	3
LIVER		(3)	(3)	(3)	(3)
No abnormality detected		3	3	3	1
Oval cell hyperplasia		0	0	0	1
Inflammatory cell infiltration, periportal		0	0	0	1
LUNG		(3)	(3)	(3)	(3)
No abnormality detected		3	0	3	2
Inflammatory cell foci		0	2	0	1
Osseous metaplasia, focal		0	1	0	0

Figures in brackets represent the number of animals from which this tissue was examined microscopically

Pathology File Ref.: PLAFOR_520419_MICMAIN_LBE_KEEP1.SPL

Table 19 (continued)
Summary of Histological Findings: Day 66

Group	:	1	2	3
Test Item	:	Control	MenPF-1	MenPF-1
Dosage (µg/dose)	:	0	25	50

HISTOLOGICAL FINDINGS	GROUP	GROUP TOTALS			
		Males		Females	
		Grp 1	Grp 3	Grp 1	Grp 3
LYMPH NODE (INGUINAL)		(3)	(3)	(3)	(3)
No abnormality detected		3	3	3	3
LYMPH NODE (LUMBAR)		(3)	(3)	(3)	(2)
No abnormality detected		1	1	1	0
Macrophage accumulation					
minimal		1	2	0	2
mild		0	0	1	0
Total Incidence		1	2	1	2
Lymphoid hyperplasia					
mild		0	1	0	1
moderate		0	1	0	1
Total Incidence		0	2	0	2
Erythrocytosis/erythrophagocytosis		2	2	1	2
LYMPH NODE (MANDIBULAR)		(3)	(3)	(3)	(3)
No abnormality detected		3	3	3	3

Figures in brackets represent the number of animals from which this tissue was examined microscopically

Pathology File Ref.: PLAFOR_520419_MICMAIN_LBE_KEEP1.SPL

Table 19 (continued)
Summary of Histological Findings: Day 66

Group	:	1	2	3
Test Item	:	Control	MenPF-1	MenPF-1
Dosage (µg/dose)	:	0	25	50

HISTOLOGICAL FINDINGS	GROUP TOTALS			
	Males		Females	
GROUP	Grp 1	Grp 3	Grp 1	Grp 3
LYMPH NODE (MESENTERIC)	(3)	(3)	(2)	(3)
No abnormality detected	3	2	1	2
Erythrocytosis/erythrophagocytosis	0	1	1	1
MAMMARY GLAND			(3)	(3)
No abnormality detected			2	3
Duct ectasia			1	0
OESOPHAGUS	(3)	(3)	(3)	(3)
No abnormality detected	3	3	3	3
OPTIC NERVE	(3)	(3)	(3)	(3)
No abnormality detected	3	3	3	3
OVARY			(3)	(3)
No abnormality detected			3	3

Figures in brackets represent the number of animals from which this tissue was examined microscopically

Pathology File Ref.: PLAFOR_520419_MICMAIN_LBE_KEEP1.SPL

Table 19 (continued)
Summary of Histological Findings: Day 66

Group	:	1	2	3
Test Item	:	Control	MenPF-1	MenPF-1
Dosage (µg/dose)	:	0	25	50

HISTOLOGICAL FINDINGS	GROUP	GROUP TOTALS			
		Males		Females	
		Grp 1	Grp 3	Grp 1	Grp 3
OVIDUCT				(3)	(3)
No abnormality detected				3	2
Cyst				0	1
PANCREAS (ENDOCRINE)		(3)	(3)	(3)	(3)
No abnormality detected		3	3	3	3
PANCREAS (EXOCRINE)		(3)	(3)	(3)	(3)
No abnormality detected		3	3	3	3
PARATHYROID GLAND		(2)	(1)	(1)	(2)
No abnormality detected		2	1	1	2
PITUITARY GLAND		(2)	(3)	(3)	(3)
No abnormality detected		2	3	3	3

Figures in brackets represent the number of animals from which this tissue was examined microscopically

Pathology File Ref.: PLAFOR_520419_MICMAIN_LBE_KEEP1.SPL

Table 19 (continued)
Summary of Histological Findings: Day 66

Group	:	1	2	3
Test Item	:	Control	MenPF-1	MenPF-1
Dosage (µg/dose)	:	0	25	50

HISTOLOGICAL FINDINGS	GROUP	GROUP TOTALS			
		Males		Females	
		Grp 1	Grp 3	Grp 1	Grp 3
PROSTATE		(3)	(3)		
No abnormality detected		3	3		
RECTUM		(3)	(3)	(3)	(3)
No abnormality detected		3	3	3	3
SALIVARY GLAND (SUBMAXILLARY)		(3)	(3)	(3)	(3)
No abnormality detected		3	3	3	3
SCIATIC NERVE		(3)	(3)	(3)	(3)
No abnormality detected		3	3	3	3
SEMINAL VESICLE		(3)	(3)		
No abnormality detected		3	3		

Figures in brackets represent the number of animals from which this tissue was examined microscopically

Pathology File Ref.: PLAFOR_520419_MICMAIN_LBE_KEEP1.SPL

Table 19 (continued)
Summary of Histological Findings: Day 66

Group	:	1	2	3
Test Item	:	Control	MenPF-1	MenPF-1
Dosage (µg/dose)	:	0	25	50

HISTOLOGICAL FINDINGS	GROUP	GROUP TOTALS			
		Males		Females	
		Grp 1	Grp 3	Grp 1	Grp 3
SKELETAL MUSCLE		(3)	(3)	(3)	(3)
No abnormality detected		3	2	3	0
Inflammatory cell foci		0	1	0	3
SKIN AND SUBCUTIS		(3)	(3)	(3)	(3)
No abnormality detected		3	3	3	3
SPINAL CORD		(3)	(3)	(3)	(3)
No abnormality detected		3	3	3	3
SPLEEN		(3)	(3)	(3)	(3)
No abnormality detected		3	3	3	3
STERNUM		(3)	(3)	(3)	(3)
No abnormality detected		3	3	3	3

Figures in brackets represent the number of animals from which this tissue was examined microscopically

Pathology File Ref.: PLAFOR_520419_MICMAIN_LBE_KEEP1.SPL

Table 19 (continued)
Summary of Histological Findings: Day 66

Group	:	1	2	3
Test Item	:	Control	MenPF-1	MenPF-1
Dosage (µg/dose)	:	0	25	50

HISTOLOGICAL FINDINGS	GROUP	GROUP TOTALS			
		Males		Females	
		Grp 1	Grp 3	Grp 1	Grp 3
STOMACH		(3)	(3)	(3)	(3)
No abnormality detected		3	3	3	3
TESTIS		(3)	(3)		
No abnormality detected		1	2		
Seminiferous epithelial degeneration, unilateral		2	0		
Segmental hypoplasia, focal		0	1		
Immaturity, unilateral		1	0		
THYMUS		(3)	(3)	(3)	(3)
No abnormality detected		3	3	3	3
THYROID GLAND		(3)	(3)	(3)	(3)
No abnormality detected		3	2	3	3
Inflammatory cell foci		0	1	0	0

Figures in brackets represent the number of animals from which this tissue was examined microscopically

Pathology File Ref.: PLAFOR_520419_MICMAIN_LBE_KEEP1.SPL

Table 19 (continued)
Summary of Histological Findings: Day 66

Group	:	1	2	3
Test Item	:	Control	MenPF-1	MenPF-1
Dosage (µg/dose)	:	0	25	50

HISTOLOGICAL FINDINGS	GROUP	GROUP TOTALS			
		Males		Females	
		Grp 1	Grp 3	Grp 1	Grp 3
TONGUE		(3)	(3)	(3)	(3)
No abnormality detected		3	3	3	3
TRACHEA		(3)	(3)	(3)	(3)
No abnormality detected		3	3	3	3
URETER		(3)	(3)	(3)	(3)
No abnormality detected		3	3	3	3
URINARY BLADDER		(3)	(3)	(3)	(3)
No abnormality detected		3	2	3	3
Mineral deposits, epithelial, surface, multifocal		0	1	0	0
UTERUS				(3)	(3)
No abnormality detected				3	3

Figures in brackets represent the number of animals from which this tissue was examined microscopically

Pathology File Ref.: PLAFOR_520419_MICMAIN_LBE_KEEP1.SPL

Table 19 (continued)
Summary of Histological Findings: Day 66

Group	:	1	2	3
Test Item	:	Control	MenPF-1	MenPF-1
Dosage (µg/dose)	:	0	25	50

HISTOLOGICAL FINDINGS	GROUP	GROUP TOTALS			
		Males		Females	
		Grp 1	Grp 3	Grp 1	Grp 3
VAGINA				(3)	(3)
No abnormality detected				3	3
GUT ASSOCIATED LYMPHOID TISSUE		(3)	(3)	(3)	(3)
No abnormality detected		3	3	3	2
Inflammation, Peyer's patch, focal		0	0	0	1
SACCULUS ROTUNDUS		(3)	(3)	(3)	(3)
No abnormality detected		3	3	3	3

Figures in brackets represent the number of animals from which this tissue was examined microscopically

Pathology File Ref.: PLAFOR_520419_MICMAIN_LBE_KEEP1.SPL

Table 20
Summary of Histological Findings: Day 92

Group	:	1	2	3
Test Item	:	Control	MenPF-1	MenPF-1
Dosage (µg/dose)	:	0	25	50

		GROUP TOTALS			
		Males		Females	
HISTOLOGICAL FINDINGS	GROUP	Grp 1	Grp 3	Grp 1	Grp 3
INJECTION SITE 1		(3)	(3)	(3)	(3)
No abnormality detected		0	1	2	1
Macrophage accumulation, intramuscular					
minimal		0	0	0	1
mild		1	0	0	0
moderate		2	2	1	1
Total Incidence		3	2	1	2
Inflammation, with necrosis					
minimal		0	1	0	0
mild		0	1	0	1
Total Incidence		0	2	0	1
Inflammation, mononuclear cell					
minimal		0	0	0	1
Total Incidence		0	0	0	1
Myofibre necrosis					
minimal		0	1	0	1
Total Incidence		0	1	0	1
Regeneration, myofibre					
minimal		0	2	0	1
Total Incidence		0	2	0	1

Figures in brackets represent the number of animals from which this tissue was examined microscopically

Pathology File Ref.: PLAFOR_520419_MICREC_LBE_KEEP1.SPL

Table 20 (continued)
Summary of Histological Findings: Day 92

Group	:	1	2	3
Test Item	:	Control	MenPF-1	MenPF-1
Dosage (µg/dose)	:	0	25	50

HISTOLOGICAL FINDINGS	GROUP TOTALS			
	Males		Females	
GROUP	Grp 1	Grp 3	Grp 1	Grp 3
INJECTION SITE 1	(3)	(3)	(3)	(3)
Fibrosis, interstitial				
minimal	0	0	0	1
mild	0	2	0	0
Total Incidence	0	2	0	1
INJECTION SITE 2		(1)		
No abnormality detected		1		
LYMPH NODE (INGUINAL)	(3)	(3)	(3)	(3)
No abnormality detected	3	3	3	2
Macrophage accumulation				
minimal	0	0	0	1
Total Incidence	0	0	0	1
LYMPH NODE (LUMBAR)	(3)	(3)	(2)	(1)
No abnormality detected	0	0	1	1

Figures in brackets represent the number of animals from which this tissue was examined microscopically

Pathology File Ref.: PLAFOR_520419_MICREC_LBE_KEEP1.SPL

Table 20 (continued)
Summary of Histological Findings: Day 92

Group	:	1	2	3
Test Item	:	Control	MenPF-1	MenPF-1
Dosage (µg/dose)	:	0	25	50

HISTOLOGICAL FINDINGS	GROUP TOTALS			
	Males		Females	
GROUP	Grp 1	Grp 3	Grp 1	Grp 3
LYMPH NODE (LUMBAR)	(3)	(3)	(2)	(1)
Macrophage accumulation				
minimal	1	1	1	0
mild	2	0	0	0
Total Incidence	3	1	1	0
Erythrocytosis/erythrophagocytosis	1	3	0	0

Figures in brackets represent the number of animals from which this tissue was examined microscopically

Pathology File Ref.: PLAFOR_520419_MICREC_LBE_KEEP1.SPL

Appendices

Appendix 1
Protocol, Amendments and Deviations

FINAL PROTOCOL

Test Facility Study No. 520419

A 9 Week Study of MenPF-1 Vaccine by Intramuscular Injection in Rabbits with a 4 Week Recovery Period

SPONSOR:
Oxford Vaccine Group
Department of Paediatrics
University of Oxford
Room 02-46-07
Children's Hospital
Oxford, OX3 9DU
UK

TEST FACILITY:
Charles River Laboratories
Preclinical Services, Tranent (PCS-EDI)
Edinburgh, EH33 2NE
UK

03 August 2011

Appendix 1 (continued)
Protocol, Amendments and Deviations

TABLE OF CONTENTS

1. OBJECTIVE(S) 3
2. PROPOSED STUDY SCHEDULE 3
3. GUIDELINES FOR STUDY DESIGN 3
4. REGULATORY COMPLIANCE 4
5. QUALITY ASSURANCE 4
6. SPONSOR 4
7. RESPONSIBLE PERSONNEL 5
8. TEST AND CONTROL ITEMS 6
9. SAFETY 8
10. DOSE FORMULATION AND ANALYSIS 8
11. TEST SYSTEM 9
12. HUSBANDRY 10
13. EXPERIMENTAL DESIGN 12
14. IN-LIFE PROCEDURES, OBSERVATIONS, AND MEASUREMENTS 13
15. LABORATORY EVALUATIONS 15
16. TERMINAL PROCEDURES 17
17. HISTOLOGY AND HISTOPATHOLOGY 20
18. COMPUTERISED SYSTEMS 21
19. STATISTICAL ANALYSIS 21
20. AMENDMENTS AND DEVIATIONS 22
21. RETENTION OF RECORDS, SAMPLES AND SPECIMENS 22
22. REPORTING 23
23. ANIMAL WELFARE 23
24. REFERENCES 25
25. TEST FACILITY APPROVAL 26
26. SPONSOR APPROVAL 27

Appendix 1 (continued)
Protocol, Amendments and Deviations

1. OBJECTIVE(S)

A prophylactic vaccine for the prevention of infection from bacterial meningitis is under development by the Sponsor. The objective of this study is to determine the potential toxicity of MenPF-1 Vaccine when given by intramuscular injection for 4 occasions over a 9 week period to rabbits to evaluate the potential reversibility of any findings, and to provide data to support the use of MenPF-1 in humans. In addition immunogenicity will be characterised.

2. PROPOSED STUDY SCHEDULE

Proposed study dates are listed below. Actual applicable dates will be included in the Final Report.

Experimental Start Date:	10 Aug 2011<br>(First date of study-specific data collection)
Experimental Completion Date:	Nov 2011<br>(Last date data are collected from the study)
Animal Arrival/Transfer:	02 Aug 2011
Initiation of Dosing:	17 Aug 2011
Completion of In-life:	16 Nov 2011<br>(Last date of necropsy)
Unaudited Draft Report:	09 Dec 2011
Final Report	10 Feb 2012<br>(Expected date of Study Director signature)

3. GUIDELINES FOR STUDY DESIGN

The design of this study was based on the study objective(s), the overall product development strategy for the test item. and the following study design guidelines:

- Committee for Medicinal Products for Human Use (CHMP). *Note for Guidance on Repeated Dose Toxicity.* CPMP/SWP/1042/99rev1.
- ICH Harmonised Tripartite Guideline S6. *Preclinical Safety Evaluation of Biotechnology-Derived Pharmaceuticals.*
- CPMP Note for Guidance on Preclinical Pharmacological and Toxicological Testing of Vaccines (CPMP/ICH/302/95). December 1997.
- WHO guidelines on nonclinical evaluation of vaccines (WHO Technical report series No. 927, 2005)
- CPMP Note for Guidance on Non-Clinical Local Tolerance Testing of Medicinal Products (CPMP/SWP/2145/00). March 2001.

Appendix 1 (continued)
Protocol, Amendments and Deviations

4. REGULATORY COMPLIANCE

This study will be performed in accordance with the OECD Principles of Good Laboratory Practice as incorporated into the United Kingdom Statutory Instrument for GLP and as accepted by Regulatory Authorities throughout the European Community, United States of America (FDA and EPA) and Japan (MHLW, MAFF and METI).

The test site for antibody determination is not a member of the UK GLP Compliance Programme, however, it is the responsibility of Charles River to implement adequate study management, monitoring and QAU mechanisms to ensure work undertaken at this test site is conducted in accordance with the principles of GLP.

5. QUALITY ASSURANCE

5.1. Test Facility

The Test Facility Quality Assurance Unit (QAU) will monitor the study to assure the facilities, equipment, personnel, methods, practices, records, and controls are in conformance with Good Laboratory Practice regulations. The QAU will review the protocol, conduct inspections at intervals adequate to assure the integrity of the study, and audit the Final Report to assure that it accurately describes the methods and standard operating procedures and that the reported results accurately reflect the raw data of the study.

5.2. Test Site

The test facility QAU conducted a pre-study facility inspection of the test site (National Institute of Biological Standards and Controls).

The conduct of the following study phase will be audited by the Test Facility QAU:

- Antibody determination

For the study phase inspected by the Test Facility QAU, copies of each inspection report will be made available to the Study Director and Test Facility Management. The Test Facility QAU will also audit the data generated and relevant sections of the report for this phase of the study.

6. SPONSOR

Sponsor Representatives

Andrew J Pollard, FRCPCH PhD
Professor of Paediatric Infection & Immunity
Oxford Vaccine Group
Department of Paediatrics
University of Oxford
Room 02-46-07
Level 2, Children's Hospital
Oxford, OX3 9DU

Appendix 1 (continued)
Protocol, Amendments and Deviations

UK
E-mail: andrew.pollard@paediatrics.ox.ac.uk

Cathy Owen, PA
Tel: +44 (0) 1865 234226/226909
Mobile: +44 (0) 7920421260

Rachel Sandford. Clinical Secretary
Tel: +44 (0) 1865 231693
Fax: +44 (0) 1865 234235

7. RESPONSIBLE PERSONNEL

Study Director

Bruce Robertson, BSc
Charles River Laboratories
Address as cited for Test Facility
Tel: +44 (0) 1875 618327
Fax: +44 (0) 1875 614555
E-mail: bruce.robertson@crl.com

Management Contact

Carol Strepka, BSc CBiol MSB
Charles River Laboratories
Address as cited for Test Facility
Tel: +44 (0) 1875 618453
Fax: +44 (0) 1875 614555
E-mail: carol.strepka@crl.com

Quality Assurance

Colin Brown, BSc
Charles River Laboratories
Address as cited for Test Facility
Tel: +44 (0) 1875 618409
Fax: +44 (0) 1875 614555
E-mail: colin.brown@crl.com

Report Peer Review

Adam Woolley MSc DABT FRCPath ERT CBiol MSB
ForthTox Limited
Linlithgow
West Lothian, EH49 7YU
UK

Appendix 1 (continued)
Protocol, Amendments and Deviations

Test Facility-designated Individual Scientists (IS)

Pathologist	TBC<br>Charles River Laboratories<br>Address as cited for Test Facility<br>Tel: +44 (0)1875 ######<br>Fax: +44 (0)1875 614555<br>E-mail: name@crl.com

Each IS is required to report any deviations or other circumstances that could affect the quality or integrity of the study to the Study Director in a timely manner. Each IS will provide a report addressing their assigned phase of the study, which will be included as an appendix to the Final Report.

Sponsor-designated Responsible Scientist (RS)

Antibody Analysis	Caroline Vipond PhD<br>Department of Bacteriology<br>National Institute of Biological Standards and Control<br>Blance Lane<br>Potters Bar<br>South Mimms<br>Hertfordshire, EN6 3QG<br>UK<br>Tel: 01707 641567<br>E-mail: caroline.vipond@nibsc.hpa.org.uk

The RS is required to report any deviations or other circumstances that could affect the quality or integrity of the study to the Study Director in a timely manner. The RS will provide results in table format (QC checked), including description of methods used addressing their assigned phase of the study, which will be included as an appendix to the Final Report.

All records from the antibody determination (copy of protocol and amendments, raw data, original QC'd data, calibration records etc.) will be returned to Charles River for archiving with remaining study data.

8. TEST AND CONTROL ITEMS

8.1. Test Item

Identification:	MenPF-1
Supplier:	Department for Biopharmaceutical production. Norwegian Institute of Public Health, Oslo, Norway
Batch (Lot) Number:	FMOX1102
Expiration Date:	Concomitant assessment, ongoing
Physical Description:	Opaque, even, milky suspension; easily redispersed

Appendix 1 (continued)
Protocol, Amendments and Deviations

Purity:	The active pharmaceutical ingredient (API), formulated as outer membrane vesicles, is a mixture of *Neisseria meningitidis* serogroup B outer membrane proteins that shows >93% adsorption degree to aluminium hydroxide adjuvant. The API contains 8.0% 70kD FetA F3-3 variant protein, 21.7% Class 1 P1.7.16 variant protein and 32.6% Class 3 P3.15 protein. The test item batch (i.e., vaccine product) contains 1.0 mg/mL aluminium. Dose calculations will not be corrected for purity.
Correction Factor:	Not applicable
Concentration:	25 µg protein/dose of 0.5 mL
Storage Conditions:	In a refrigerator set to maintain 4°C

8.2. Control Item

Identification:	MOX Control
Supplier:	Department for Biopharmaceutical production, Norwegian Institute of Public Health, Oslo, Norway
Batch (Lot) Number:	FMOX1103
Expiration Date:	Concomitant assessment, ongoing
Physical Description:	Opaque, even milky suspension, easily redispersed
Purity:	The product contains Alhydrogel, specifically containing 1.1 mg/mL aluminium. Dose calculations will not be corrected for purity.
Concentration:	0.333 % w/v Alhydrogel in 3% sucrose solution
Storage Conditions:	In a refrigerator set to maintain 4°C

8.3. Test and Control Item Characterisation

The Sponsor will provide to the Test Facility documentation of the identity, strength, purity, composition, and stability for the test and control item(s). A Certificate of Analysis (CoA) or equivalent documentation will be provided for inclusion in the Final Report. The Sponsor will also provide information concerning the regulatory standard that was followed for these evaluations. Potency data (immunogenicity) will not be provided as part of the CoA on delivery of the test item.

The Sponsor has appropriate documentation on file concerning the method of synthesis, fabrication or derivation of the test and control items, and this information is available to the appropriate regulatory agencies should it be requested.

8.4. Reserve Samples

For each batch (lot) of test and control item, a reserve sample (approximately 1 vial) will be collected and maintained under the appropriate storage conditions by the Test Facility.

Appendix 1 (continued)
Protocol, Amendments and Deviations

8.5. Test and Control Item Inventory and Disposition

Records of the receipt, distribution, and storage of test and control items will be maintained. With the exception of reserve samples, all unused Sponsor-supplied test and control items will be returned to the Sponsor after finalisation of the study report.

Shipping Contact

Andrew J Pollard, FRCPCH PhD
Professor of Paediatric Infection & Immunity
Oxford Vaccine Group
Department of Paediatrics
University of Oxford
Room 02-46-07
Level 2, Children's Hospital
Oxford, OX3 9DU
UK

Cathy Owen, PA
Tel: +44 (0) 1865 234226/226909
Mobile: +44 (0) 7920421260

Rachel Sandfold, Clinical Secretary
Tel: +44 (0) 1865 231693
Fax: +44 (0) 1865 234235

9. SAFETY

Safety instructions for this study are provided on the Sponsor supplied safety data sheet. An internal COSHH safety sheet will be prepared at Charles River.

10. DOSE FORMULATION AND ANALYSIS

10.1. Preparation of Control Item

The control item, MOX control, is provided in single dose vials for administration to Group 1 control animals. No aliquoting of the control item is required and 0.5 mL will be withdrawn from each vial. The control item vials will be stored in a refrigerator set to maintain 4°C until use. The aliquots will be removed from the refrigerator and allowed to warm to room temperature for at least 30 minutes before dosing. To ensure homogeneity, the vials must be shaken before drawing the volume intended for injecting.

Any residual volumes will be discarded before issuance of the Final Report.

10.2. Preparation of Test Item

The test item, MenPF-1 Vaccine, is provided in single dose vials and will be administered as received. No aliquoting of the test item is required. An adequate amount of the test item will be dispensed; 0.5 mL of suspension for 25 microgrammes of protein. The vials will be removed

Appendix 1 (continued)
Protocol, Amendments and Deviations

from the refrigerator and allowed to warm to room temperature for at least 30 minutes before dosing. To ensure homogeneity, the vials must be shaken before drawing the volume intended for injecting.

Any residual volumes will be discarded before issuance of the Final Report.

10.3. Sample Collection and Analysis

The test and control items will be used as received from the Sponsor: therefore, samples for dose formulation analysis will not be collected by the Test Facility.

11. TEST SYSTEM

Species:	Rabbit
Strain:	New Zealand White
Source:	Harlan UK
Number of Males Ordered:	18
Number of Females Ordered:	18
Target Age at the Initiation of Dosing:	12 weeks
Target Weight at the Initiation of Dosing:	2.5 kg

The actual age, weight, and number of animals received will be listed in the Final Report.

11.1. Justification of Test System and Number of Animals

At this time, studies in laboratory animals provide the best available basis for extrapolation to humans and are required to support regulatory submissions. Acceptable models which do not use live animals currently do not exist.

The rabbit has been selected by the Study Director in consultation with the Sponsor as the test model:
- to satisfy regulatory requirements for toxicity testing.
- because of the availability of background data and proven suitability in toxicology studies.

Immunogenicity can also be investigated in this species.

The number of animals chosen for this study is the smallest number considered necessary to provide sufficient data.

11.2. Animal Identification

Each animal will receive a unique ear tag which will identify it individually within the study and which corresponds to that animal's number.

Appendix 1 (continued)
Protocol, Amendments and Deviations

11.3. Environmental Acclimation

The animals will be allowed to acclimate to the Charles River rabbit toxicology accommodation for a period of up to 2 weeks before the commencement of dosing.

11.4. Selection, Assignment, Replacement and Disposition of Animals

Animals will be removed in random order from their transport boxes and allocated to dose group on arrival by placing them in separate cages. Cages will be housed on racks according to treatment and labelled with the study number, animal number and group number.

Control animals will be housed on a separate rack.

Animals suspected of being diseased will be culled from the study. If significant numbers of animals are unsuitable, the entire batch will be rejected by the Study Director and a new batch obtained.

During the week before the commencement of dosing, the animals will be approved for entry into the experiment on the basis of satisfactory clinical observation records and body weight profile.

The disposition of all animals will be documented in the study records.

12. HUSBANDRY

12.1. Housing

Cage type:	Stainless steel cages containing an automatic watering valve, mesh tops and a metal food hopper with a 'Noryl' dual level interior and perforated floor. Beneath each cage will be a suspended tray containing absorbent paper. Paper will be changed at least once each week.
Cage size:	Approximate dimensions 77 x 70 x 48 cm.
Cage rack:	Cages will be suspended on movable racks
Animal housing:	Individually

Cages and racks will be changed as necessary throughout the course of the study as detailed in Charles River SOPs.

The animal room floor and work surfaces will be washed as necessary with disinfectant solution.

12.2. Environmental Conditions

The targeted conditions for animal room environment will be as follows:

Temperature:	16°- 20°C
Humidity:	40%-85%
Ventilation:	A minimum of 15 air changes per hour

Appendix 1 (continued)
Protocol, Amendments and Deviations

Light Cycle: 12 hours light and 12 hours dark (except when interrupted by study procedures/activities)

There will be automatic control of temperature which will be continuously monitored and recorded. Humidity will be continuously monitored and recorded. Deviations from target temperature and humidity ranges will be presented in the study report.

There will be automatic control of light cycle.

12.3. Food

Each animal will receive Harlan Diet supplied by Harlan, UK.

The food will be available to the animals *ad libitum*. Each animal will also be offered a supplement of hay at least 3 times per week.

The diet used is considered not to contain any additional substances, in sufficient concentration, to have any influence on the outcome of the study.

The diet will be supplied with a batch analysis for major nutritive components and significant contaminants and will be used within the manufacturers' designated shelf-life. The hay is not analysed.

An analytical certificate for each batch of diet used will be retained at Charles River, Edinburgh.

12.4. Water

The animals will have access to water *ad libitum* from the public supply.

The water used by Charles River Edinburgh is analysed at regular intervals for dissolved materials, heavy metals, pesticide residues, pH, nitrates and nitrites. Microbiological screening is also conducted. An analytical certificate for each analysis will be retained at Charles River, Edinburgh.

The water used is considered not to contain any additional substances, in sufficient concentration, to have any influence on the outcome of the study.

12.5. Animal Enrichment

Wooden chewsticks and bunny blocks will be provided with a certificate of analysis for significant contaminants. An analytical certificate for each batch of chewsticks and bunny blocks used will be retained at Charles River, Edinburgh.

Other items may be included to enrich the cage environment. Details will be given in the study report.

12.6. Veterinary Care

All animals are under the care of Charles River clinical veterinary surgeons, who are available at all times to provide advice and assistance. All treatment used to prevent or control intercurrent diseases will be implemented at the discretion of the Study Director, and where possible after consultation with the Sponsor. Records will be maintained for all affected individual animals

Appendix 1 (continued)
Protocol, Amendments and Deviations

and will include date of first observation and duration of the condition, the nature and dates of the treatment administered and the outcome of the treatment in relation to the disease and to the test results.

13. EXPERIMENTAL DESIGN

Experimental Design

Group Number	Animal Numbers				Test Item	Dosage (ug/dose)	Conc. (ug/mL)	Dose Volume (mL/dose)
	Main Study		Recovery					
	M	F	M	F				
1	1-3	19-21	4-6	28-30	MOX Control	0	0	0.5 mL
2	7-9	22-24	10-12	31-33	MenPF-1	25	50	0.5 mL
3	13-15	25-27	16-18	34-36	MenPF-1	50	50	2 x 0.5 mL

13.1. Administration of Test and Control Items

The test and control items will be administered to the appropriate rabbits by intramuscular injection on Days 1, 22, 43 and 64. The dose volume will be 0.5 mL or 2 x 0.5 mL. The first day of dosing for each animal will be designated as Day 1. The injection site will be the left hind limb (Injection Site 1). The same site will be used each injection. The site will be clipped free from hair and marked afterwards.

Vaccine vials will be inverted before dosing.

For necropsy the site will be clipped free from hair and marked.

13.2. Justification of Route and Dosage Levels

The intramuscular route of administration has been selected for this study as this route has been defined by the Sponsor as the route of clinical application/human exposure.

The dose levels have been agreed with the Sponsor and took into account the maximum tolerated dose in the test model and other factors such as anticipated therapeutic dose. The test item has been produced with a similar methodology as for the vaccine product MenBvac (Norwegian Institute of Public Health), based on deoxycholate extracted outer membrane vesicles from *Neisseria meningitidis*. MenBvac is known to be moderately reactogenic but safe in humans (Nøkleby et al. Vaccine 2007: 16: 3080-4).

Clinical injections are planned every 6 weeks, with three doses intended. In this preclinical study injections will be given over a shorter period and one more injection (n + 1) will be given. The intended clinical dose is 25 µg/dose. This amount and 2x this amount is being given in full in this preclinical study and based on body weight of rabbit 3 kg: human 60 kg and the administration of an additional injection this is considered to provide adequate safety data.

Appendix 1 (continued)
Protocol, Amendments and Deviations

14. IN-LIFE PROCEDURES, OBSERVATIONS, AND MEASUREMENTS

The in-life procedures, observations, and measurements listed below will be performed for all main study and recovery animals.

14.1. Mortality/Moribundity Checks

Frequency: All animals will be checked early morning and as late as possible each day for viability.

Procedure: Any animal showing signs of severe debility or intoxication and if determined to be moribund or suffering excessively will be euthanised.

14.2. Clinical Observations

14.2.1. Detailed Clinical Observations

Frequency: Once weekly commencing during the last week of the prestudy period.

Procedure: Animals removed from the cage for examination.

14.2.2. Postdose Observations

Frequency: Dosing days - Regularly throughout the day.
Non-dosing days - Once each day.

Procedure: All the animals will be examined for reaction to treatment. The onset, intensity and duration of these signs will be recorded (if appropriate), particular attention being paid to the animals during and for the first hour after dosing.

14.3. Dermal Scoring

Frequency: At each injection: 0 h (before dosing). 24 h, 48 h after dosing.

Procedure: Skin will be assessed for erythema and eschar formation, oedema formation, skin thickening, desquamation and any other reaction to treatment.

Erythema and Eschar Formation	Grade
No erythema	0
Very slight erythema (barely perceptible)	1
Well defined erythema	2
Moderate to severe erythema	3
Severe erythema (beet redness) to slight eschar formation (injuries in depth)	4

Oedema Formation	Grade
No oedema	0

Appendix 1 (continued)
Protocol, Amendments and Deviations

Very slight oedema (barely perceptible)	1
Slight oedema (edges of area well defined by definite raising)	2
Moderate oedema (raised approximately 1 mm)	3
Severe oedema (raised more than 1 mm and extending beyond the area of exposure)	4

14.4. Body Weights

Frequency: Pretrial – Once
Dosing Period – Twice weekly
Recovery Period – Twice weekly

Procedure: Animals showing weight loss or deterioration in condition will be weighed more frequently as necessary.

14.5. Food Consumption

Frequency: Pretrial – Twice weekly
Dosing Period – Twice weekly
Recovery Period – Twice weekly

Procedure: The quantity of food consumed by each animal will be measured and recorded.

14.6. Water Consumption

Procedure: Water consumption will not be measured as all animals are on an automatic watering system.

14.7. Ophthalmic Examinations

Frequency: Pretrial – Once
Dosing Period – At end of dosing period

Procedure: The eyes will be examined using an indirect ophthalmoscope after the application of a mydriatic agent (1% Tropicamide, Mydriacyl®). The anterior, lenticular and fundic areas will be examined.

14.8. Body Temperature

Frequency: Pretrial – All animals once
Dosing Period –0 h (before dosing), 1 h, 3 h, 24 h and 48 h after dosing

Procedure: Measured by digital thermometer inserted into ear.

Appendix 1 (continued)
Protocol, Amendments and Deviations

15. LABORATORY EVALUATIONS

15.1. Clinical Pathology

15.1.1. Sample Collection

Blood will be collected from an auricular artery. Additional blood samples may be obtained (e.g. due to clotting of non-serum samples) if permissible sampling frequency and blood volume are not exceeded. After collection, samples will be transferred to the appropriate laboratory for processing.

Animals will not be fasted. Samples will be collected according to the following table.

Samples for Clinical Pathology Evaluation

Group Nos.	Time Point	Haematology	Coagulation	Clinical Chemistry
1-3	Pretrial	X	X	X
1-3	Day 66	X	X	X
1-3	Day 92	X	X	X
Unscheduled euthanasia (when possible)	Before euthanasia	X	X	X

X = sample to be collected.

Any residual/retained clinical pathology samples will be discarded before issuance of the Final Report.

15.1.2. Haematology

Target Volume: 0.5 mL

Anticoagulant: EDTA

Haematology Parameters

Red blood cell count	White blood cell count
Haemoglobin	Neutrophils
Haematocrit	Lymphocytes
Mean cell volume	Monocytes
Mean cell haemoglobin concentration	Eosinophils
Mean cell haemoglobin	Basophils
Reticulocytes	Large unstained cells
Reticulocyte count (absolute)	Other cells (as appropriate)
Red blood cell distribution width	
Platelets	
Blood Smear (see ^ below)	

^ A blood smear will be prepared from each haematology specimen. Blood smears will be labelled, stained, stored and archived. The smears may be subsequently evaluated and this will be described in a protocol amendment with approval of the Study Director and Sponsor. A

Appendix 1 (continued)
Protocol, Amendments and Deviations

decision to evaluate the blood smears will be based upon the possibility that evaluation may further elucidate changes that have occurred in the numerical haematology parameters.

15.1.3. Coagulation

Target Volume: 0.9 mL

Anticoagulant: 3.8% (w/v) trisodium citrate

Processing: To plasma

Coagulation Parameters

Activated partial thromboplastin time<br>Fibrinogen	Prothrombin time

15.1.4. Clinical Chemistry

Target Volume: 1.5 mL

Anticoagulant: Lithium Heparin

Processing: To plasma

Clinical Chemistry Parameters

Urea	Total protein
Glucose	Albumin
Aspartate aminotransferase	Globulin
Alanine aminotransferase	Albumin/globulin ratio
Alkaline phosphatase	Cholesterol
Creatine phosphokinase	Creatinine
Lactate dehydrogenase	Total bilirubin
Sodium	Calcium
Potassium	Inorganic phosphate
Chloride	

15.1.5. Bone Marrow Smear Evaluation

Bone marrow smears will be collected as described in the Tissue Collection and Preservation table (Section 16.5). Evaluation of stained smears may be added by amendment at the discretion of the Study Director in consultation with the pathologist and the Sponsor.

15.2. Antibody Sample Collection, Processing, and Analysis

Blood will be collected from all animals from an auricular artery.

Time Points: Pretrial, before dosing on Day 22 and Day 64 and on Day 92.

Target Volume: 2 mL

Anticoagulant: None

Processing: To serum – centrifugation at least 1500 g/2°-8°C/10 min

Appendix 1 (continued)
Protocol, Amendments and Deviations

The serum samples will be stored in a freezer set to maintain -80°C and then shipped on dry ice. All samples must remain frozen (temperature required -80°C) and temperature must be recorded during transportation.

Shipping Contact
Caroline Vipond, PhD
Department of Bacteriology
National Institute of Biological Standards and Control
Blance Lane
Potters Bar
South Mimms
Hertfordshire, EN6 3QG
UK
Tel: 01707 641567
E-mail: caroline.vipond@nibsc.hpa.org.uk

The immunology laboratory will be notified before shipment of the samples. Upon receipt at the immunology laboratory, the samples will be stored ≤ -20°C.

The samples will be analysed for antibodies against MenPF-1 using a validated ELISA analytical method. No validation has been performed for the plate reader software, however the calibration performed by the service engineer confirms Operational Qualification (OQ) and standards, and QC samples run with each batch of samples confirm Performance Qualification (PQ) of the reader.

Any residual/retained anti-therapeutic antibody samples will be retained for research purposes. The results from any subsequent analyses of these samples will not be covered in this study.

16. TERMINAL PROCEDURES

Terminal procedures are summarised in the following table:

Terminal Procedures for Main Study and Recovery Animals

Group Number	Number of Animals		Scheduled Euthanasia Day	Necropsy Procedures			Histology	Histopathology
	M	F		Necropsy	Tissue Collection	Organ Weights		
1	3	3	66	X	X	X	Full Tissue	Full Tissue[a]
2	3	3					None	None
3	3	3					Full Tissue	Full Tissue[a]
1	3	3	92	X	X	X	Select Tissues	Select Tissues[b]
2	3	3					None	None
3	3	3					Select Tissues	Select Tissues[b]
Unscheduled Deaths				X	X	-	Full Tissue	Full Tissue

X = procedure to be conducted; - = not applicable.
[a] See Tissue Collection and Preservation table for listing of tissues.

Appendix 1 (continued)
Protocol, Amendments and Deviations

[b] Injection site and lumbar and inguinal lymph node.

16.1. Unscheduled Deaths

If a main study or recovery animal dies on study, a necropsy will be conducted and specified tissues will be saved. If necessary, the animal will be refrigerated to minimise autolysis.

Main study or recovery animals may be euthanised for humane reasons as per Test Facility SOPs. The body weight will be recorded and samples for evaluation of clinical pathology parameters and antibody analysis will be obtained if possible as specified in Section 15. These animals will undergo necropsy, and specified tissues will be retained. If necessary, the animal will be refrigerated to minimise autolysis.

16.2. Scheduled Euthanasia

Main study and recovery animals surviving until scheduled euthanasia will have a terminal body weight recorded, and will be euthanised by an intravenous overdose of a barbiturate, followed by exsanguination. When possible, the animals will be euthanised rotating across dose groups such that similar numbers of animals from each group, including controls will be necropsied throughout the day. Animals will not be fasted before their scheduled necropsy.

16.3. Necropsy

Main study and recovery animals will be subjected to a complete necropsy examination, which will include evaluation of the carcass and musculoskeletal system; all external surfaces and orifices; cranial cavity and external surfaces of the brain; and thoracic, abdominal, and pelvic cavities with their associated organs and tissues. Necropsy examinations will be conducted by a trained technician and will consist of an external and internal examination and recording of observations for all animals. A veterinary pathologist will be available for consultation during normal working hours.

At the discretion of the necropsy supervising pathologist, images may be generated for illustration of or consultation on gross observations. Generation of such images will be documented and communicated to the Study Director. Images and associated documentation will be retained and archived.

16.4. Organ Weights

The organs identified for weighing in the Tissues Collection and Preservation table will be weighed at necropsy for all scheduled euthanasia animals. Organ weights will not be recorded for animals found dead or euthanised in poor condition or in extremis. Paired organs will be reported together. Terminal body weights will be used for organ weight analysis.

16.5. Tissue Collection and Preservation

Representative samples of the tissues identified in the Tissue Collection and Preservation table will be collected from all animals and preserved in 10% neutral buffered formalin, unless otherwise indicated. Additional tissue samples may be collected to elucidate abnormal findings.

Appendix 1 (continued)
Protocol, Amendments and Deviations

Tissue Collection and Preservation

Tissue	Weigh	Collect	Microscopic Evaluation	Comment
Administration site	-	X	X	Injection Site 1. Collect additional muscle around marked area as contingency.
Animal identification	-	X	-	-
Artery, aorta	-	X	X	From thoracic segment.
Bone marrow smear	-	X	-	One bone marrow smear will be collected from the femur at scheduled necropsies only (for possible examination). Smears will not be collected from animals that are found dead. Bone marrow smears are allowed to air dry and are not fixed in formalin.
Bone marrow, femur	-	X	X	Collect with bone, femur
Bone marrow, sternum	-	X	X	Collect with bone, sternum
Bone, femur with articulating surface	-	X	X	Collect distal end to include femoral tibial joint.
Bone, sternum	-	X	X	-
Brain	X	X	X	Forebrain, midbrain, cerebellum, and medulla oblongata.
Cervix	-	X	X	Collect with uterus.
Epididymis	X	X	X	Separate weights and examination.
Eye	-	X	X	Separate examination; Preserve in Davidson's fixative.
Gallbladder	-	X	X	
Gland, adrenal	X	X	X	Separate weights and examination.
Gland, lacrimal	-	X	X	Only 1 required for examination.
Gland, mammary	-	X	X	Collect with thoracic skin and include nipple; mammary gland will be examined in females only
Gland, parathyroid	-	X	X	Collect with thyroid: Examine only if present in the routine section of thyroid.
Gland, pituitary	X	X	X	-
Gland, prostate	X	X	X	-
Gland, salivary	-	X	X	Submandibular: Only 1 required for examination.
Gland, seminal vesicle	-	X	X	
Gland, thyroid	X	X	X	Separate weights and examination; weight includes parathyroid
Gross lesions/masses	-	X	X	-
Gut-associated lymphoid tissue	-	X	X	Collect with small intestine.
Heart	X	X	X	-
Kidney	X	X	X	Separate weights and examination.
Large intestine, appendix	-	X	X	
Large intestine, caecum	-	X	X	-
Large intestine, colon	-	X	X	-
Large intestine, rectum	-	X	X	-
Large intestine, sacculus rotundus	-	X	X	
Liver	X	X	X	Drain gallbladder before weighing
Lung	X	X	X	Infuse with 10% neutral buffered formalin after weighing.
Lymph node, mandibular	-	X	X	Only 1 required for examination.

Appendix 1 (continued)
Protocol, Amendments and Deviations

Tissue	Weigh	Collect	Microscopic Evaluation	Comment
Lymph node, mesenteric	-	X	X	-
Lymph node, lumbar	-	X	X	Identify left and right.
Lymph node, inguinal	-	X	X	Identify left and right.
Muscle, skeletal	-	X	X	From thigh
Nerve, optic	-	X	X	Preserve in Davidson's fixative; Examine only if present in the routine section of the eye.
Nerve, sciatic	-	X	X	Only 1 required for examination.
Oesophagus	-	X	X	-
Ovary	X	X	X	Separate weights and examination.
Oviduct	-	X	X	Only 1 required for examination. Collect with uterus
Pancreas	-	X	X	-
Skin	-	X	X	Collect with mammary gland.
Small intestine, duodenum	-	X	X	-
Small intestine, ileum	-	X	X	-
Small intestine, jejunum	-	X	X	-
Spinal cord	-	X	X	Cervical, thoracic, lumbar.
Spleen	X	X	X	-
Stomach	-	X	X	Fundus and pylorus
Testis	X	X	X	Separate weights and examination; Preserve in Modified Davidson's fixative.
Thymus	X	X	X	-
Tongue	-	X	X	-
Trachea	-	X	X	-
Ureter	-	X	X	Only 1 required for examination.
Urinary bladder	-	X	X	-
Uterus	X	X	X	-
Vagina	-	X	X	-

X = procedure to be conducted; - = not applicable.

17. HISTOLOGY AND HISTOPATHOLOGY

17.1. Histology

Tissues in the Tissue Collection and Preservation table from animals identified in the Terminal Procedures table will be embedded in paraffin, sectioned, mounted on glass slides. and stained with haematoxylin and eosin.

17.2. Histopathology

Histopathological evaluation will be performed by a veterinary pathologist with training and experience in laboratory animal pathology. Any additional stains or evaluations, if deemed necessary by the pathologist, will be added by protocol amendment following discussion with the Study Director and in consultation with the Sponsor.

At the discretion of the study pathologist and after acknowledgement by the study director, images may be captured for consultation purposes.

Appendix 1 (continued)
Protocol, Amendments and Deviations

17.3. Pathology Peer Review

A pathology peer review, as per the appropriate SOP of the Pathology Department, will be conducted by a second pathologist at:

Charles River Laboratories
Preclinical Services
Tranent
Edinburgh, EH33 2NE
UK

The peer review statement or equivalent documentation will be included as an appendix to the Final Report.

18. COMPUTERISED SYSTEMS

The following critical computerised systems will be used in the study. Any additional critical computerised systems used during the course of the study will be added by protocol amendment. The actual critical computerised systems used will be specified in the Final Report.

Data for parameters not required by protocol, which are automatically generated by analytical devices used will be retained on file but not reported. Statistical analysis results that are generated by the program but are not required by protocol and/or are not scientifically relevant will be retained on file but will not be included in the tabulations.

Critical Computerised Systems

System Name	Description of Data Collected and/or Analysed
Dispense	Dose Formulation
Provantis	In-life data collection and reporting
Nautilus 2003	Clinical Pathology Laboratory Information Management System (LIMS)
PLACES 2000	Histopathology/Organ Weights

19. STATISTICAL ANALYSIS

Unless otherwise stated, all statistical tests will be two-sided and performed at the 5% significance level using in-house software. Males and females will be analysed separately.

Pairwise comparisons will only be performed against the control group (Group 1). The following pairwise comparisons will be performed:

Control Group v Group 2
Control Group v Group 3

Body weight, food consumption, haematology, coagulation and clinical chemistry will be analysed for homogeneity of variance using the 'F-Max' test. If the group variances appear homogeneous, a parametric ANOVA will be used and pairwise comparisons will be made using Fisher's F protected LSD method via Student's t test i.e. pairwise comparisons will be made only

Appendix 1 (continued)
Protocol, Amendments and Deviations

if the overall F-test is significant. If the variances are heterogeneous, log or square root transformations will be used in an attempt to stabilise the variances. If the variances remain heterogeneous, then a Kruskal-Wallis non-parametric ANOVA will be used and pairwise comparisons will be made using chi squared protection (*via* z tests, the non-parametric equivalent of Student's t test).

In circumstances where it is not possible to perform the F Max test due to zero standard deviation in at least one group, the non-parametric ANOVA results will be reported.

Organ weights will be analysed using ANOVA as above and by analysis of covariance (ANCOVA) using terminal kill body weight as covariate. In addition, organ weights as a percentage of terminal body weight will be analysed using ANOVA.

In circumstances where the variances in the ANCOVA remain heterogeneous following log or square root transformations, the data will be subjected to a rank transformation prior to analysis. Where it is not possible to perform the F-Max test due to the small sample size (less than 3 animals in any group), the untransformed parametric ANCOVA results will be reported.

In the ANOVA and ANCOVA summary tables, the results of the analysis will be reported indicating the level of statistical significance ($p<0.05$, $p<0.01$ and $p<0.001$) of each pairwise comparison.

Actual p-values will not be reported in the summary tables for these analyses.

More extensive analysis will be carried out only after consultation with the sponsor and will involve additional costs.

20. AMENDMENTS AND DEVIATIONS

Changes to the approved protocol shall be made in the form of an amendment, which will be signed and dated by the Study Director. Every reasonable effort will be made to discuss any necessary protocol changes in advance with the Sponsor.

All protocol and SOP deviations will be documented in the study records. Deviations from the protocol and/or SOP related to the phase(s) of the study conducted at a Test Site shall be documented, acknowledged by the RS, and reported to the Study Director for authorisation/acknowledgement. The Study Director will notify the Sponsor of deviations that may result in a significant impact on the study as soon as possible.

21. RETENTION OF RECORDS, SAMPLES AND SPECIMENS

All study-specific raw data, documentation, protocol, samples, specimens, and interim (if applicable) and final reports from this study are the property of the Sponsor. These materials will be available at the Test Facility during the study and will be transferred to the Test Facility archive by no later than the date of final report issuance and will be archived for a period of 2 years. After this period, the Sponsor will be contacted to determine the disposition of these materials.

Electronic data generated by the Test Facility will be archived and the software and hardware required to produce it in a readable form will be maintained and available.

Appendix 1 (continued)
Protocol, Amendments and Deviations

All records, samples, specimens and reports generated from phases or segments performed by Test Facility-designated subcontractors and the Test Site will be returned to the Test Facility for archiving.

Records to be maintained will include, but will not be limited to, documentation and data for the following:

- Protocol, protocol amendments, and deviations
- Study schedule
- Study-related correspondence
- Test system receipt, health, and husbandry
- Test and control item receipt, identification, preparation, and analysis
- In-life measurements and observations
- Clinical pathology sample collection and evaluation
- Bioanalytical sample collection and evaluation
- Gross and microscopic observations and related data (including internal peer review notes)
- Organ weight measurements
- Statistical analysis results

22. REPORTING

A comprehensive Draft Report will be prepared following completion of the study and will be finalised following consultation with the Sponsor. The report will include all information necessary to provide a complete and accurate description of the experimental methods and results and any circumstances that may have affected the quality or integrity of the study.

The Sponsor will receive an electronic version of the Draft and Final Report provided in Adobe Acrobat PDF format (bookmarked and searchable at final) along with a Microsoft Word version of the text. The PDF document will be created from native electronic files to the extent possible, including text and tables generated by the Test Facility. Report components not available in native electronic files and/or original signature pages will be scanned and converted to PDF image files for incorporation. An original copy of the report with the Test Facility's handwritten signatures will be retained.

23. ANIMAL WELFARE

The UK Home Office controls scientific procedures on animals in the UK and does so by the issue of licences under the Animals (Scientific Procedures) Act 1986. The regulations conform to the European Convention for the Protection of Vertebrate Animals Used for Experimental and Other Scientific Purposes (Strasbourg, Council of Europe) and achieve the standard of care required by the US Department of Health and Human Services' Guide for the Care and Use of Laboratory Animals.

Appendix 1 (continued)
Protocol, Amendments and Deviations

The Home Office licence governing this study strictly specifies the limits of severity of effects on the animals. From the available information, the procedures described in the protocol are not anticipated to cause any effects which exceed the severity limit of the procedure. Any animal which shows unacceptable reactions may be euthanised or other actions taken as required by the Home Office to alleviate distress.

23.1. Home Office Project Licence No.

PPL 60/4185, Toxicology of Pharmaceuticals, Protocol 1.

Appendix 1 (continued)
Protocol, Amendments and Deviations

24. REFERENCES

None.

Appendix 1 (continued)
Protocol, Amendments and Deviations

25. TEST FACILITY APPROVAL

The signature below indicates that Test Facility Management approves the Study Director identified in this protocol.

Date: 03 August 2011

Andy Danks, BSc
Test Facility Management

The signature below indicates that the Study Director approves the study protocol.

Date: 03 AUG 2011

Bruce Robertson, BSc
Study Director

Appendix 1 (continued)
Protocol, Amendments and Deviations

26. SPONSOR APPROVAL

The signature of the Sponsor Representative below indicates approval of this protocol. The protocol was approved by the Sponsor on 02 Aug 2011.

Date: 2/8/11.

Andrew J Pollard, FRCPCH PhD
Sponsor Representative

Appendix 1 (continued)
Protocol, Amendments and Deviations

charles river

PROTOCOL AMENDMENT NO. 1

A 9 Week Study of MenPF-1 Vaccine by Intramuscular Injection in Rabbits with a 4 Week Recovery Period

Test Facility Study No. 520419

Note: Additions are indicated in bold text. Deletions are indicated in strikethrough text.

1. Section 13. Experimental Design

Experimental Design

Group Number	Animal Numbers				Test Item	Dosage (ug/dose)	Conc. (ug/mL)	Dose Volume (mL/dose)
	Main Study		Recovery					
	M	F	M	F				
1	~~1-3~~ **1-3**	~~19-21~~ **10-12**	~~4-6~~ **19-21**	~~28-30~~ **28-30**	MOX Control	0	0	0.5 mL
2	~~7-9~~ **4-6**	~~22-24~~ **13-15**	~~10-12~~ **22-24**	~~31-33~~ **31-33**	MenPF-1	25	50	0.5 mL
3	~~13-15~~ **7-9**	~~25-27~~ **16-18**	~~16-18~~ **25-27**	~~34-36~~ **34-36**	MenPF-1	50	50	2 x 0.5 mL

Justification(s):

To amend animal numbers to a more suitable order.

2. Section 15.1.1. Sample Collection

Blood will be collected from an auricular artery. Additional blood samples may be obtained (e.g. due to clotting of non-serum samples) if permissible sampling frequency and blood volume are not exceeded. After collection, samples will be transferred to the appropriate laboratory for processing.

Animals will not be fasted. Samples will be collected according to the following table.

Samples for Clinical Pathology Evaluation

Group Nos.	Time Point	Haematology	Coagulation	Clinical Chemistry
1-3	Pretrial	X	X	X
1-3	Day **64** ~~66~~	X	X	X
1-3	Day 92	X	X	X

Appendix 1 (continued)
Protocol, Amendments and Deviations

Group Nos.	Time Point	Haematology	Coagulation	Clinical Chemistry
Unscheduled euthanasia (when possible)	Before euthanasia	X	X	X

X = sample to be collected.

Any residual/retained clinical pathology samples will be discarded before issuance of the Final Report.

Justification(s):

To allow animal unit to take blood sample for Clinical Pathology and Anti-Antibody analysis at the same collection time.

Appendix 1 (continued)
Protocol, Amendments and Deviations

Amendment Approval:

Date: 05 AUG 2011.

Bruce Robertson, BSc
Study Director

Date: 20/1/12

Andrew J Pollard, FRCPCH PhD
Sponsor Representative

Appendix 1 (continued)
Protocol, Amendments and Deviations

charles river

PROTOCOL AMENDMENT NO. 2

A 9 Week Study of MenPF-1 Vaccine by Intramuscular Injection in Rabbits with a 4 Week Recovery Period

Test Facility Study No. 520419

Note: Additions are indicated in bold text. Deletions are indicated in strikethrough text.

1. Amendment 1, Item 2, Section 15.1.1. Sample Collection

Blood will be collected from an auricular artery. Additional blood samples may be obtained (e.g. due to clotting of non-serum samples) if permissible sampling frequency and blood volume are not exceeded. After collection, samples will be transferred to the appropriate laboratory for processing.

Animals will not be fasted. Samples will be collected according to the following table.

Samples for Clinical Pathology Evaluation

Group Nos.	Time Point	Haematology	Coagulation	Clinical Chemistry
1-3	Pretrial	X	X	X
1-3	Day ~~64~~ **66**	X	X	X
1-3	Day 92	X	X	X
Unscheduled euthanasia (when possible)	Before euthanasia	X	X	X

X = sample to be collected.

Any residual/retained clinical pathology samples will be discarded before issuance of the Final Report.

Justification(s):

Amendment 1 incorrectly changed the day of clinical pathology sampling at Day 66 to Day 64. A bleed on Day 64 would not be considered fit for purpose as animals would not have received their final dose. Following discussions with the Sponsor, the bleed was re-instated on Day 66.

Appendix 1 (continued)
Protocol, Amendments and Deviations

Protocol Amendment No. 2

Page 2
Test Facility Study No. 520419

Amendment Approval:

Bruce Rob. Date: 10 Aug 2011.
Bruce Robertson, BSc
Study Director

Date: 15/8/11
Andrew J Pollard, FRCPCH PhD
Sponsor Representative

Appendix 1 (continued)
Protocol, Amendments and Deviations

charles river

PROTOCOL AMENDMENT NO. 3

A 9 Week Study of MenPF-1 Vaccine by Intramuscular Injection in Rabbits with a 4 Week Recovery Period

Test Facility Study No. 520419

Note: Additions are indicated in bold text. Deletions are indicated in strikethrough text.

1. Section 7. Responsible Personnel

Study Director

~~Bruce Robertson, BSc~~
~~Charles River Laboratories~~
~~Address as cited for Test Facility~~
~~Tel: +44 (0) 1875 618327~~
~~Fax: +44 (0) 1875 614555~~
~~E-mail: bruce.robertson@crl.com~~

Elizabeth Donald, BSc
Charles River Laboratories
Preclinical Services
Tranent
Edinburgh EH33 2NE
UK
Tel: +44 (0) 1875 618732
Fax: +44 (0) 1875 614555
E-mail: elizabeth.donald@crl.com

Justification(s):

To change Study Director to cover for a period of temporary absence.

Appendix 1 (continued)
Protocol, Amendments and Deviations

Protocol Amendment No. 3

Page 2
Test Facility Study No. 520419

Amendment Approval:

The signature below indicates that Test Facility Management approves the Study Director identified in this protocol amendment.

Date: 02 September 2011
Andy Danks, BSc
Test Facility Management

Date: 02 Sep 2011
Elizabeth Donald, BSc
Study Director

Date: 5/9/11
Andrew J Pollard, FRCPCH PhD
Sponsor Representative

Appendix 1 (continued)
Protocol, Amendments and Deviations

charles river

PROTOCOL AMENDMENT NO. 4

A 9 Week Study of MenPF-1 Vaccine by Intramuscular Injection in Rabbits with a 4 Week Recovery Period

Test Facility Study No. 520419

Note: Additions are indicated in bold text. Deletions are indicated in strikethrough text.

1. Section: Various

At the second injection Animal 27M (Group 3) received a skin nick during clipping of the injection site, which resulted in the injection being given into the right hind limb. The animal was inspected by the veterinary surgeon and any treatment was recorded and will be reported.

The right hind limb injection site will be designated Injection site 2 and clipping and marking will be as is occurring for Injection site 1. The skin nick has healed and subsequent injections will be given in the left hind limb (Injection site 1).

For this animal, both injection sites will be collected at necropsy and examined histologically.

The Study Director agreed this change with the technical staff at the time of injection and the Sponsor was informed.

Justification(s):

To formally document an agreed change and to inform all parties.

2. Section 7. Responsible Personnel

Study Director

~~Elizabeth Donald, BSc~~
~~Charles River Laboratories~~
~~Preclinical Services~~
~~Tranent~~
~~Edinburgh EH33 2NE~~
~~UK~~
~~Tel: +44 (0) 1875 618732~~
~~Fax: +44 (0) 1875 614555~~
~~E-mail: elizabeth.donald@crl.com~~

Bruce Robertson, BSc
Charles River Laboratories
Address as cited for Test Facility
Tel: +44 (0) 1875 618327

Appendix 1 (continued)
Protocol, Amendments and Deviations

Fax: +44 (0) 1875 614555
E-mail: bruce.robertson@crl.com

Justification(s):

The original Study Director has returned to work following a period of temporary absence.

Appendix 1 (continued)
Protocol, Amendments and Deviations

Amendment Approval:

The signature below indicates that Test Facility Management approves the Study Director identified in this protocol amendment.

Date: 23 September 2011

Andy Danks, BSc
Test Facility Management

Date: 23 Sep 2011

Bruce Robertson, BSc
Study Director

Date: 23/9/11

Andrew J Pollard, FRCPCH PhD
Sponsor Representative

Appendix 1 (continued)
Protocol, Amendments and Deviations

charles river

PROTOCOL AMENDMENT NO. 5

A 9 Week Study of MenPF-1 Vaccine by Intramuscular Injection in Rabbits with a 4 Week Recovery Period

Test Facility Study No. 520419

Note: Additions are indicated in bold text. Deletions are indicated in strikethrough text.

1. **Section. 27. Attachment**

Justification(s):

To include the Responsible Scientists Standard Operating Procedure for the Anti-MenPF1 Rabbit Immunoglobin ELISA.

Appendix 1 (continued)
Protocol, Amendments and Deviations

National Institute for Biological Standards and Control

Division of Bacteriology

Standard Operating Procedure

Title: **Anti-MenPF-1 Rabbit Immunoglobulin ELISA**

(Relevant to Study Number: 520419)

Changes to previous version are tracked in red with deletions shown as ^

Appendix 1 (continued)
Protocol, Amendments and Deviations

National Institute for Biological Standards and Control

1. INTRODUCTION

MenPF-1 is a developmental vaccine against disease caused by *Neisseria meningitidis* (the meningococcus). The major antigens in this vaccine are the outer membrane proteins PorA and FetA. The vaccine contains outer membrane vesicles (OMVs) from a meningococcal strain genetically modified to over-express the FetA antigen.

MenPF-1 OMVs, adsorbed to aluminium hydroxide ($Al(OH)_3$) adjuvant, have been produced by the Norwegian Institute of Public Health (NIPH). A validation batch (Lot number FMOX1102) of the MenPF-1 vaccine will be tested for *in vivo* toxicity in rabbits. This toxicology study has been contracted to Charles River Laboratories (CRL) by the University of Oxford **(Charles River Study Number: 520419)**. The results of this study will be used to support an application by the University of Oxford for the use of MenPF-1 in a Phase 1 clinical trial in humans.

During the toxicology study, New Zealand White rabbits are given four doses of $Al(OH)_3$-only control inoculum or $Al(OH)_3$-adjuvanted MenPF-1 OMVs. Rabbits receiving MenPF-1 OMVs are given either a single human dose (25µg total protein) or double human dose (50µg total protein). Doses are given on days 1, 22, 43 and 64. Blood samples are collected from each rabbit pre-immunisations, before dosing on day 22, before dosing on day 64 and on day 92. Blood samples are processed at Charles River Laboratories, and extracted serum samples are stored at -20°C.

The immunological testing of serum samples has been sub-contracted to NIBSC by CRL. An *in vitro* Enzyme Linked Immunosorbent Assay (ELISA) is used at NIBSC to determine seroconversion of rabbits in the study. Seroconversion is defined as the development of detectable specific antibodies raised against the vaccine in response to immunisation. The ELISA will be used to demonstrate seroconversion in the rabbits which should switch from MenPF-1 seronegative to MenPF-1 seropositive if successfully immunised. The binding of antibodies in pre- and post-vaccination sera to MenPF-1 OMVs will be assessed using a validated assay of suitable sensitivity and specificity.

2. SAMPLE RECEIPT AND DOCUMENTATION

Serum samples are shipped by CRL to NIBSC on dry ice and delivered to the responsible operator in Bacteriology. On receipt within the division of Bacteriology, receipt will be recorded according to BACT/REC (Document S/N 369). All documents relevant to this study will be labelled with the Charles River Study Number: 520419. Scanned copies of all documents will be stored in the bact\MenPFtox drive. Samples are stored at -20°C in freezer BT077.

3. FORMS USED IN CONNECTION WITH THIS SOP

Buffer and reagent forms:

- SOP: BACT/BUF (Document S/N 388)
- BACT/MEDIACB (Document S/N 498)
- BACT/MEDIA10xPBS (Document S/N 2965)
- BACT/MEDIA50mMPBS (Document S/N 2964)
- BACT/MEDIA 1M SA (Document S/N 2966)

Other forms:

- MenPF-1 Rabbit ELISA test record form (Document S/N 6116)
- BACT/REC (Document S/N 369)
- SOP: TDI/SOP/RANDOM (Document S/N 4628)

Appendix 1 (continued)
Protocol, Amendments and Deviations

4. MATERIALS

Unless otherwise stated in the SOP, there is no requirement to use volumetric glassware in traceable calibration for the preparation of reagents, solutions or dilutions. Semi-automated pipettes, disposable plastic graduated pipettes, and measuring cylinders, appropriate to the volume being used, are adequate for this purpose. Volumes of less than 1.0 ml are dispensed using suitable pipettes in calibration. All salts used in the preparation of buffers should be of minimum General Purpose Reagent grade, unless otherwise stated.

5. EQUIPMENT

*TREND-monitored +4℃ refrigerator BT076.
*TREND-monitored -20℃ freezer BT077.
*Suitable pipettes in calibration.
*Multichannel pipette in calibration.
*Bibbyjet pipettor.
*Lab Timer.
*Plate washer.
*Microplate reader.
*Vortex mixer.
96 well microtitre plates (Nunc Maxisorb).
Measuring cylinders.
Marker pen.
Racks for tubes and universals.
Buffer reservoirs for multichannel pipettes.
Disposable, sterile serological pipettes.
Container for incubating plates.
Paper towels.
Plastic microtubes.
Plastic universals.
Plastic bijoux.
Glass beakers.

*Equipment records available.

6. RISK ASSESSMENT

Safety glasses and gloves must be worn when handling material of animal origin. A risk assessment for this procedure can be found on the NIBSC Safety Organiser database.

7. CRITICAL REAGENTS

Coating Antigen: Unadsorbed MenPF-1 OMVs (Validation batch 1), sterile, in 3% Sucrose/0.01% Thimerosal with a total protein concentration of 0.45mg/ml. OMVs were produced at NIPH and shipped to NIBSC on 10/06/2011. The OMVs are assigned an expiry date of 6 months from receipt. Upon receipt, OMVs are stored at +4°C in a suitable container to protect from light. The container is labelled with content details and dates of receipt and expiry.

Positive control: Anti-MenPF-1 Rabbit serum (NIBSC 11/1475). A pool of serum from four rabbits is used as the positive control. Aliquots of the positive serum are stored at -20°C, and allowed to thaw at room temperature before use. Aliquots are marked each time they are thawed, and discarded after three freeze/thaw cycles. Serum is assigned a shelf life of one year when stored at -20°C.

Appendix 1 (continued)
Protocol, Amendments and Deviations

Negative control: Normal Rabbit serum (Sigma #R9133, Lot number 089K6004). Stored at -20°C in suitable aliquots. Aliquots are marked each time they are thawed, and discarded after three freeze/thaw cycles. An expiry date of 1 year from receipt is assigned to this reagent.

8. OTHER REAGENTS

All buffers should be prepared when required according to SOP: BACT/BUF (Document S/N 388). Reagents marked with ** can be obtained from Scientific Support Services (SSS), or can be made according to the SOPs.

Coating Buffer: Prepared following instructions on form BACT/MEDIACB (Document S/N 498).

Phosphate Buffered Saline (PBS) (x 10 concentrate): Prepared following instructions on form BACT/MEDIA10xPBS (Document S/N 2965).

Phosphate Buffered Saline (PBS) (x 1): Prepared following instructions on form BACT/MEDIA50mMPBS (Document S/N 2964).

Wash Buffer: 1 x PBS containing 0.01% polyethylene sorbitan monolaurate (Tween 20, supplied by Sigma Aldrich, #P1379) (PBST). Prepared on the day of the assay by diluting 10 x PBS 1/10 in purified water containing 0.01% (v/v) Tween 20.

Newborn Bovine or Foetal Calf Serum: Supplied by SSS. Each new batch needs to be validated by testing 3x in parallel using the previously validated batch three months prior to replacing the batch being used. Stored at –20°C.

Dilution Buffer: PBS containing 5% (v/v) Foetal calf serum; prepared on the day of the assay.

Goat Anti-rabbit HRP conjugate (Sigma #A6154 or equivalent): Stored at -20°C in suitable aliquots. Aliquots are marked each time they are thawed, and discarded after three freeze/thaw cycles. An expiry date of 1 year from receipt is assigned to this reagent.

TMBlue Substrate: Supplied by Universal Biologicals Ltd #T118. Stored at +4°C. An expiry date of 1 year from receipt is assigned to this reagent.

1M Sulphuric acid: Prepared following instructions on form BACT/MEDIA 1M SA (Document S/N 2966).

Appendix 1 (continued)
Protocol, Amendments and Deviations

9. PROCEDURE – **Enzyme Linked Immunosorbent Assay**

Record all details of the test, including samples tested, dilutions made, critical timings, pipette serial numbers, and buffers and reagents used on the MenPF-1 Rabbit ELISA test record form (Document S/N 6116).

a). Prepare a solution of 2µg/ml MenPF-1 OMV in coating buffer according to the following table:

Number of Plates	Total Solution Volume (ml)	Volume Coating Buffer (ml)	Volume OMV stock (µl)
1	12	11.947	53
2	24	23.893	107
3	34	33.849	151
4	45	44.800	200
5	55	54.756	244
6	65	64.711	289

Coat the appropriate wells of microtitre plates with 100µl of solution. Cover and incubate the plates at +4°C for a minimum of 16 hours in a sealed container which has been labelled to be identifiable to the test operator.

b). Wash the ELISA plates with Wash Buffer using the Skatran Plate washer. If the machine has been switched off or the connected buffer has been changed from that required by this assay, a blank plate must first be used to Rinse the machine with pure water, and then Prime the machine with the required Wash Buffer. All buffer changes should be recorded on the test record form.

c). Block plates with 100µl per well of Dilution Buffer. Cover the plates and incubate for a minimum of 1 hour (+10 minutes) at room temperature in a sealed container.

d). Wash the ELISA plates as in step b).

e). Prepare dilutions of the sera to be tested and the positive control by diluting in Dilution Buffer as follows:
- For positive control sera, dilute 1:500 (1:10 followed by 1:50);
- For negative control sera, dilute 1:100 (1:10 followed by 1:10);
- For test sera taken on day 0 (Test Sample 1), dilute 1:100 (1:10 followed by 1:10);
- For test sera taken on day 22 (Test Sample 2), dilute 1:300 (1:10 followed by 1:30);
- For test sera taken on day 64 (Test Sample 3), dilute 1:900 (1:10 followed by 1:90);
- Fest test sera taken on day 92 (Test Sample 4), dilute 1:900 (1:10 followed by 1:90).

f). Prepare ELISA plates. One 96-well plates is required to test all serum samples extracted from each rabbit and the positive control serum at a maximum of 8 dilutions for each serum sample. All samples (except Blank and negative controls) are tested in duplicate columns (see example plate layout in Figure 1). Samples are assigned randomly to columns following the method described in SOP: TDI/SOP/RANDOM (Document S/N 4628). For a standard assay, random plate layouts have been generated and can be found on the MenPF-1 Rabbit ELISA test record form (Document S/N 6116). For rabbits for which less than four serum samples are available, columns listed as “Test Sample 4” are left Blank. Record which template is being used in the assay on the MenPF-1 Rabbit ELISA test record form (Document S/N 6116). Use a different template for each assay and rotate in the order 1 through to 8.

Appendix 1 (continued)
Protocol, Amendments and Deviations

Figure 1: Example plate layout (all samples and controls are randomised across the plate). See appendix for detailed plate layout templates.

	TS2	+ve	-ve	-ve	TS1	TS3	TS4	+ve	TS2	TS4	TS3	TS1
	1	2	3	4	5	6	7	8	9	10	11	12
A	1/1*	1/1*	1/1*	1/1*	1/1*	1/1*	1/1*	1/1*	1/1*	1/1*	1/1*	1/1*
B	1/3	1/3	1/3	1/3	1/3	1/3	1/3	1/3	1/3	1/3	1/3	1/3
C	1/9	1/9	1/9	1/9	1/9	1/9	1/9	1/9	1/9	1/9	1/9	1/9
D	1/27	1/27	1/27	1/27	1/27	1/27	1/27	1/27	1/27	1/27	1/27	1/27
E	etc.	etc.	etc.	etc.	etc.	etc.	etc.	etc.	etc.	etc.	etc.	etc.
F												
G												
H												

*Pre-diluted test sample or positive control.
TS = Test serum
+ve = Positive control serum
-ve = Negative control serum

h). Fill wells in rows B - H with 100µl of Dilution Buffer, leaving row A empty.

i). 150µl of each diluted preparation is added to wells in row A. 50µl of each sample is then removed and transferred to the appropriate wells in row B. Following mixing for a minimum of 5 times, 50µl volumes are transferred to the next row (C). This procedure is repeated down the plate. Following mixing of row H 50µl of sample is discarded from each well. Each well in rows A through to H should now contain 100µl volumes. Plates are then covered and incubated at room temperature in the sealed container for a minimum of 1 hours (+10 minutes).

j). Wash the ELISA plates as in step b).

k). Dilute goat anti-rabbit HRP conjugate 1:2000 in Dilution Buffer. Add 100µl to all wells. Cover and incubate plates for a minimum of 1 hour (+10 minutes) in a sealed container at room temperature.

l). Wash the ELISA plates as in step b).

m). Add 100µl TMBlue substrate to all wells. Incubate at room temperature for up to ten minutes. Following colour development, add 100µl 1M sulphuric acid to all wells to stop the reaction. The plates are read at 450 nm using a microplate reader. Logon to the computer linked to the plate reader and read the plates using Genesis (or equivalent) plate reader software. Each plate that is read is automatically assigned (by the software) a file name with the date and a sequential number, e.g. 20JUN08W.001. Operator's initials should be added to the beginning of this file name, e.g. HS_20JUN08W.001. Record the file name(s) and software protocol used to read the plate(s) on the test record form. Data generated by the plate reader is saved by default to the C:\ drive on the computer linked to the plate reader and can be found in C:\genesis\protocol*, where * is the protocol used for reading the plates. Copy the data from C:\ to the "Raw Data" file in the bact\MenPFtox drive.

n). Raw data should be printed out immediately, signed and dated.

10. DATA ANALYSIS, VALIDITY AND DETERMINATION OF SEROCONVERSION

Absorbance levels across the dilution series from each test sample are used to directly compare the levels of IgG binding following immunisation of each rabbit to pre-trial sera, in order to determine whether each rabbit was seroconverted. All calculations will be recorded on the print-out of the raw data and reviewed by the Study Director. No computer software is required for data analysis.

10.1. DATA ANALYSIS

a) Referring to the dilution series listed below, for each test sample determine the highest dilution factor at which the absorbance at 450nm is higher than 0.70 (where at least two consecutive dilutions are higher than the threshold, except where only a 1/100 dilution of a sample has an absorbance higher than 0.70). The dilution factor is recorded as "IG". If a sample does not result in absorbance higher than 0.70 at a dilution of 1:100, IG is recorded as 100. If higher or lower dilutions (to a minimum of 1/100) are required to determine IG, the test sample must be repeated with appropriate dilutions.

For duplicates of a single sample, if IG values are one dilution apart, a mean value is taken as the IG for that sample. If IG values for duplicates of a single sample are greater than one dilution apart, that sample must be repeated.

	Row	Positive	Test Sample 1 /Negative	Test Sample 2	Test Sample 3/4
Start dilution →	A	500	100	300	900
Dilution series ↓ (1:3)	B	1500	300	900	2700
	C	4500	900	2700	8100
	D	13500	2700	8100	24300
	E	40500	8100	24300	72900
	F	121500	24300	72900	218700
	G	364500	72900	218700	656100
	H	1093500	218700	656100	1968300

b) For each test sample 2, 3 and 4, calculate the increase in binding following vaccination as follows:

$$\Delta IG = IG_{(\text{Test Sample } n)} / IG_{(\text{Test Sample } 1)}$$

Where "*n*" = 2, 3 or 4.

For the positive control serum, ΔIG is calculated as follows:

$$\Delta IG = IG_{(\text{Positive control serum})} / IG_{(\text{Negative control serum})}$$

c) Record values for ΔIG on the test record form.

10.2. VALIDITY REQUIREMENTS

In order for the test to be valid:

Appendix 1 (continued)
Protocol, Amendments and Deviations

i). The maximum absorbance at 450nm for the positive control serum must be greater than 3.0 for both repeats.

ii). The minimum absorbance at 450nm for the positive control serum must be less than 0.7 for both repeats.

iii). The maximum absorbance at 450nm for the negative control serum must be greater than 0.7.

iv). The ΔIG value calculated for the positive control serum must be between 90 and 810.

Validity of the assay is recorded on the test record form.

A test is repeated if it does not meet the validity requirements, if IG values are greater than one dilution apart for duplicates of any test sample, or if alternative dilutions are required to determine IG values for any test sample.

10.3. DETERMINATION OF SEROCONVERSION

When analysis of serum samples from all animals is complete, seroconversion is determined for each time point after initiation of the trial (Day 22, Day 64 and Day 92). For each post-vaccination serum sample, when $\Delta IG \geq 4$ seroconverion is determined to have occurred.

11. RECORDING OF RESULTS

Copies of all raw and analysed data, as well as scanned copies of all test record forms, will be stored in the bact\MenPFtox drive. Hard copies of all test record forms and raw data will be stored in B38. When analysis of all serum samples is complete all printed and electronic data will be sent to Charles River Laboratories for review and incorporation into their test report.

12. INTERNAL DATA MONITORING

IG values obtained for positive and negative control sera will be recorded in the file "Data Monitoring" in bact\MenPFtox\Raw Data\Data Monitoring. A table of the results can be viewed at any time.

13. COMPETENCY

This test has been developed for a single use over a time period of less than six months. Initial competency has been determined during assay development and will remain valid throughout the time period required for test completion. If the use of this test is delayed, or if it becomes necessary to repeat the test at a later date, competency for this test may be obtained through completion of similar assays. If no similar assay has been completed by the operator within 12 months prior to the start of the test, the competency of that operator must be re-evaluated before testing can begin.

14. UNCERTAINTY OF MEASUREMENT

Uncertainty in the procedure covered by this SOP may result from a number of general factors, such as:

- Variability in assay system
- Human factors
- Homogeneity of the sample

Appendix 1 (continued)
Protocol, Amendments and Deviations

- Environmental factors-temperature of laboratory
- Dilutions of test samples and references preparations
- Instrumental factors

Instrumental factors are listed in the Table below:

Measurement	Equipment	Estimated Uncertainty	Documentation
Volumes	Gilson pipettes	<2.5%	Certification of calibration of pipettes, SOP:BACT/PIP and associated log books
Volumes	Disposable plastic pipettes	<1%	Manufacturers specifications
Volumes	Measuring cylinder	1%	Manufacturers specifications
pH (of buffers)	pH meter	0.25%	Certificate of calibration of buffers.
Weight (of reagents in buffers)	Balance	1%	Certificate of calibration of weight.

The contributions to the error in the final result made by instrumental factors are small. Furthermore, the result for any test sample in this assay is expressed as a post-vaccination dilution factor relative to a pre-vaccination sample included in the same assay, and so any sources of error due to environmental factors will cancel out. The remaining sources of error are random human operational error and variability in the assay system.

The uncertainty of measurement for this ELISA test has been considered. The contributions of instrumental sources or error to the final result of the test are small and can be considered negligible.

Appendix 1 (continued)
Protocol, Amendments and Deviations

National Institute for Biological Standards and Control

Appendix

ELISA PLATE LAYOUT TEMPLATES

Random plate layouts for Anti-MenPF-1 Rabbit Immunoglobulin ELISA

Test sample, reference, and positive control added to row A of 96 well plates as indicated below:

Key:

+: **Positive serum**
B: **Blank wells**
TS: **Test sample**

	1	2	3	4	5	6	7	8	9	10	11	12
**Template 1**	TS2	+	B	B	TS1	TS3	TS4	+	TS2	TS4	TS3	TS1
**Template 2**	B	TS3	TS4	+	B	TS3	TS1	TS4	+	TS1	TS2	TS2
**Template 3**	TS3	TS2	TS1	B	TS4	TS2	TS1	TS3	+	TS4	B	+
**Template 4**	TS1	TS3	B	TS4	+	TS2	+	TS1	TS2	TS4	B	TS3
**Template 5**	B	TS1	TS4	TS2	B	+	TS3	TS2	TS4	TS1	TS3	+
**Template 6**	B	TS2	TS4	TS2	TS1	TS3	+	TS1	TS3	B	TS4	+
**Template 7**	TS4	+	TS4	B	TS2	TS2	+	TS3	B	TS1	TS3	TS1
**Template 8**	TS3	TS3	TS4	TS1	+	TS1	TS2	+	TS4	B	B	TS2

Appendix 1 (continued)
Protocol, Amendments and Deviations

Amendment Approval:

Bruce Rh
____ Date: 18 NOV 2011.
Bruce Robertson, BSc
Study Director

____ Date: 8/2/12
Andrew J Pollard, FRCPCH PhD
Sponsor Representative

Appendix 1 (continued)
Protocol, Amendments and Deviations

charles river

PROTOCOL AMENDMENT NO. 6

A 9 Week Study of MenPF-1 Vaccine by Intramuscular Injection in Rabbits with a 4 Week Recovery Period

Test Facility Study No. 520419

Note: Additions are indicated in bold text. Deletions are indicated in strikethrough text.

1. **Section 7. Responsible Personnel**

Test Facility-designated Individual Scientists (IS)

Pathologist	~~TBC~~ **Lise Bertrand, DVM, MSc, DESV, DiplECVP**<br>Charles River Laboratories<br>Address as cited for Test Facility<br>Tel: +44 (0)1875 ~~######~~ **618512**<br>Fax: +44 (0)1875 614555<br>E-mail: ~~name~~ **lise.bertrand**@crl.com

Each IS is required to report any deviations or other circumstances that could affect the quality or integrity of the study to the Study Director in a timely manner. Each IS will provide a report addressing their assigned phase of the study, which will be included as an appendix to the Final Report.

Justification(s):

To include details of the study pathologist following confirmation, for completeness.

Appendix 1 (continued)
Protocol, Amendments and Deviations

Amendment Approval:

Bruce Robertson Date: 25 Nov 2011.
Bruce Robertson, BSc
Study Director

Date: 29/11/11
Andrew J Pollard, FRCPCH PhD
Sponsor Representative

Appendix 1 (continued)
Protocol, Amendments and Deviations

Protocol Deviations

Protocol section 11 Test System

The target age and weight for the animals at the start of dosing was 12 weeks and 2.5 kg, respectively. At the initiation of dosing animals were approximately 13-14 weeks of age and weighed 2.6-2.8 kg for males and 2.7-3.1 kg for females. The difference between the actual age and weight and the target was considered to be small and rabbits at 13-14 weeks old are still considered to be young adults. This deviation was considered not to have impacted on the outcome or integrity of the study.

Protocol section 11.3 Environmental Acclimatisation

The protocol stated animals would be acclimatised for a period of up to 2 weeks before the first administration. Animals were acclimatised for 15 days before administration of MOX control or MenPF-1 vaccine. This additional day before the start of dosing had no observable effect on the animals and was considered not to have impacted on the outcome or integrity of the study.

Protocol section 12.2 Environmental Conditions

On several occasions the humidity and temperature in the animal room was outside the target range of 16-20°C for temperature and 40-85% humidity. The actual temperature range was 14-22°C, while the humidity range was 29-71%. The environmental deviations did not cause any overt effect in any animal, consequently it was considered that the study outcome was unaffected.

Protocol section 15.2 Antibody Sample Collection, Processing and Analysis

A discrepancy between the protocol and the Standard Operating Procedure (SOP) provided by NIBSC and authorised in Amendment 5 (dated 18 November 2011) was noted in the storage temperatures of antibody samples at NIBSC. The protocol stated on receipt of antibody samples at NIBSC, samples would be stored at ≤-20°C, whereas the SOP stated samples would be stored at -20°C. A review of the trend data monitoring the freezers at NIBSC indicated the samples were stored in a freezer which was running at *ca* -26°C. Although this was consistent with the Protocol, this was a deviation to the SOP which NIBSC were using for antibody sample analysis. As samples were stored at Charles River in a freezer set to maintain -80°C, and as the samples were held at NIBSC only slightly cooler than the SOP, samples were still held frozen and within ranges which samples were held at Charles River and NIBSC. As a result this deviation was considered not to have impacted on the outcome or integrity of the study or conclusions drawn.

Appendix 2
Certificates of Analysis for Test and Control Items

Norwegian Institute of Public Health

08SKJ-GEN-133, ver 2.0
Page of 2

CERTIFICATE OF ANALYSIS

CoA

Tested in accordance with GMP

Product: *Men PF-1* **Storage:** *2-8°C*

Batch number: *FMOX1102* **Expiry date:** *N.a*

**Test**	**Specification:** *07SPE-MOX-003* **Version:** *2.0*	**Test result**	**Journal number**
Aluminium	< 1.25 mg/ml	*1.0 mg/ml*	*J17-11/005*
Endotoxin	< 1 x 10^5 IU/ml	*<1 x 10^5 IU/ml*	*J44-11/037*
Identity	70 kDa (Fet A F3-3): Detected<br>Class 1, (P1.16): Detected<br>Class 3 (P3.15): Detected	*Detected*<br>*Detected*<br>*Detected*	*J3-11/017*
pH	To be reported	*6.1*	*J13-11/023*
Potency	To be reported	*See comments*	*N.a*
Pyrogenicity	Pass	*Pass*	*J41-11/007*
Sterility	Pass	*Pass*	*J40-11/038*
Appearance	Opaque, even, milky suspension; easily redispersed	*Opaque, even, milky suspension; easily redispersed*	*J6-11/019*
Extractable volume	≥ 0.50 ml	*≥ 0.50 ml*	*J7-11/009*
Degree of adsorption	To be reported	*> 93%*	*J16-11/006*

Appendix 2 (continued)
Certificates of Analysis for Test and Control Items

Norwegian Institute of Public Health

08SKJ-GEN-133, ver 2.0
Page 2 of 2

CERTIFICATE OF ANALYSIS
CoA

Tested in accordance with GMP

Product: *Men PF-1* **Storage:** *2-8°C*

Batch number: *FMOX1102* **Expiry date:** *N.a*

Deviations from SOP, manufacturing formula, specifications. Deviation No:

A11/098				

	Date	Sign.
The values are correctly transferred from specification and primary data	06/07/2011	VB
	6/7-11	NMJ
The test results fulfil the specifications for the product: **Yes / ~~No~~**	06/07/2011	Vegard Bråthen<br>**Head of QC**

Comments:
Test Abnormal toxicity: Pass (J26-11/001)

A11/098: One cassette had too high Al-content. This cassette is discarded.

Potency test: The test is not established yet and will be performed in August 2011.

The quality control of the product is: **Approved** / ~~**Not Approved**~~

06/07/2011
Date

Vegard Bråthen
Head of Quality Control

Appendix 2 (continued)
Certificates of Analysis for Test and Control Items

Norwegian Institute of Public Health

08SKJ-GEN-133, ver 2.0
Page 1 of 2

CERTIFICATE OF ANALYSIS

CoA

Tested in accordance with GMP

Product: *Men PF-1, bulk* **Storage:** *2-8°C*

Batch number: *MOX1101* **Expiry date:** *N.a*

Test	Specification: 07SPE-MOX-002 Version: 2.0	Test result	Journal number
Inactivation control	Inactivated material	*Pass*	*J42-11/007*
Total protein, pre formulation	To be reported	*0.74 mg/ml*	*J9-11/018*
Bioburden, pre sterile filtration	TAMC < 10^1/ml<br>TYMC < 10^1/ml<br>Total < 10^1/ml	*TAMC < 10^1/ml*<br>*TYMC < 10^1/ml*<br>*Total < 10^1/ml*	*J46-11/019*
Antigen pattern<br>70kD Fet A (F 3-3)<br>Class 1 P1.16<br>Class 3 P 3.15	 To be reported<br>To be reported<br>To be reported	 *8.0%*<br>*21.7%*<br>*32.6%*	 *J3-11/017*
Antigen pattern<br>70kD Fet A F 3-3<br>Class 1 (P1.16)<br>Class 3 (P 3.15)<br>LPS 3,7,9	 Detected<br>Detected<br>Detected<br>Detected	 *Detected*<br>*Detected*<br>*Detected*<br>*Detected*	 *J2-11/005*
Deoxycholate	< 0.4 µg/µg protein	*0.26 µg/µg protein*	*J14-11/007*
LPS (Lipopoly saccharide)	Total: < 0.12<br>LPS 3,7,9: to be reported	*0.044 µg/µg protein*<br>*LPS 3,7,9:*<br>*0.043 µg/µg protein*	*J5-11/006*
Total protein	0.45 - 1.24 mg/ml	*0.60 mg/ml*	*J9-11/019*
Appearance	Turbid, white to yellow, even suspension, easily redispersed	*Turbid, white to yellow, even suspension, easily redispersed*	*J6-11/020*
pH	To be reported	*7.3*	*J13-11/024*

Appendix 2 (continued)
Certificates of Analysis for Test and Control Items

Norwegian Institute of Public Health

08SKJ-GEN-133, ver 2.0
Page 2 of 2

CERTIFICATE OF ANALYSIS

CoA

Tested in accordance with GMP

Product: *Men PF-1, bulk* **Storage:** *2-8°C*

Batch number: *MOX1101* **Expiry date:** *N.a*

Deviations from SOP, manufacturing formula, specifications. Deviation No:

	Date	Sign.
The values are correctly transferred from specification and primary data	01/07/2011	VB
	1/7-11.	S.D.
The test results fulfil the specifications for the product: **Yes** / ~~**No**~~	01/07/2011	Vegard Bråthen<br>**Head of QC**

Comments:

The quality control of the product is: **Approved** / ~~**Not Approved**~~

01/07/2011
Date

Vegard Bråthen
Head of Quality Control

Appendix 2 (continued)
Certificates of Analysis for Test and Control Items

Norwegian Institute of Public Health

08SKJ-GEN-133, ver 2.0
Page 2 of 2

CERTIFICATE OF ANALYSIS

CoA

Tested in accordance with GMP

Product: *MOX Control* **Storage:** *2-8°C*

Batch number: *FMOX1103* **Expiry date:** *N.a*

Test	Specification: 07SPE-MOX-004 Version: 2.0	Test result	Journal number
Extractable volume	≥ 0.5 ml	*≥ 0.5 ml*	*J7-11/008*
Aluminium	< 1.25 mg/ml	*1.1 mg/ml*	*J17-11/006*
Test for sterility	Pass	*Pass*	*J40-11/042*
pH	To be reported	*pH 6.0*	*J13-11/028*
Endotoxin	≤ 5 IU/ml	*≤ 5 IU/ml*	*J44-11/035*
Appearance	Opaque, even, milky suspension; easily redispersed	*Opaque, even, milky suspension; easily redispersed*	*J6-11/024*

Deviations from SOP, manufacturing formula, specifications. Deviation No:

Appendix 2 (continued)
Certificates of Analysis for Test and Control Items

Norwegian Institute of Public Health

08SKJ-GEN-133, ver 2.0
Page 2 of 2

CERTIFICATE OF ANALYSIS

CoA

Tested in accordance with GMP

Product: *MOX Control* **Storage:** *2-8°C*

Batch number: *FMOX1103* **Expiry date:** *N.a*

	Date	Sign.
The values are correctly transferred from specification and primary data	06/07/2011	VB
	6/7-11	NMJ
The test results fulfil the specifications for the product: **Yes** / ~~No~~	06/07/2011	Vegard Bråthen<br>**Head of QC**

Comments:
N.a

The quality control of the product is: **Approved** / ~~**Not Approved**~~

06/07/2011
Date

Vegard Bråthen
Head of Quality Control

Appendix 3
Individual Clinical Observations: Treatment Period

Key to Appendix 3

Pre = Predose
IPD = Immediate post dose
+3h = 3 hour post dose

The in-life data capture system records the first day of treatment as Day 0.

Appendix 3 (continued)
Individual Clinical Observations: Treatment Period

Group	:	1	2	3
Test Item	:	Control	MenPF-1	MenPF-1
Dosage (µg/dose)	:	0	25	50

Sex	Group	Animal	Observation	Days
M	1	1	General Observation, No Abnormality Detected.	-7-0,7,14,21,28,35,42,49,56,63
			No dosing abnormalities.	0(Pre)-20,21(Pre)-41,42(Pre)-62, 63(Pre)-65
			terminal kill.	65
M	1	2	General Observation, No Abnormality Detected.	-7-0,7,14,21,28,35,42,49,56,63
			No dosing abnormalities.	0(Pre)-20,21(Pre)-41,42(Pre)-62, 63(Pre)-65
			terminal kill.	65
M	1	3	General Observation, No Abnormality Detected.	-7-0,7,14,21,28,35,42,49,56,63
			No dosing abnormalities.	0(Pre)-20,21(Pre)-41,42(Pre)-62, 63(Pre)-65
			terminal kill.	65
M	1	19	General Observation, No Abnormality Detected.	-7-0,7,14,21,28,35,42,49,56,63
			No dosing abnormalities.	0(Pre)-20,21(Pre)-41,42(Pre)-62, 63(Pre)-65
M	1	20	General Observation, No Abnormality Detected.	-7-0,7,14,21,28,35,42,49,56,63
			No dosing abnormalities.	0(Pre)-20,21(Pre)-41,42(Pre)-62, 63(Pre)-65
M	1	21	General Observation, No Abnormality Detected.	0,7,14,21,28,35,42,49,56,63
			No dosing abnormalities.	0(Pre)-20,21(Pre)-41,42(Pre)-62, 63(Pre)-65
			Few area(s) of sparse hair	-7
M	2	4	General Observation, No Abnormality Detected.	-7-0,7,14,21,28,35,42,49,56,63
			No dosing abnormalities.	0(Pre)-20,21(Pre)-41,42(Pre)-62, 63(Pre)-65
			terminal kill.	65
M	2	5	General Observation, No Abnormality Detected.	-7-0,7,14,21,28,35,42,49,56,63
			No dosing abnormalities.	0(Pre)-20,21(Pre)-41,42(Pre)-62, 63(Pre)-65
			terminal kill.	65
M	2	6	General Observation, No Abnormality Detected.	-7-0,7,14,21,28,35,42

Appendix 3 (continued)
Individual Clinical Observations: Treatment Period

Group	:	1	2	3
Test Item	:	Control	MenPF-1	MenPF-1
Dosage (µg/dose)	:	0	25	50

Sex	Group	Animal	Observation	Days
M	2	6	No dosing abnormalities.	0(Pre)-20,21(Pre)-41,42(Pre)-62, 63(Pre)-65
			discoloured skin on, limb(s), dose site 1	56,63
			One lesion(s) on, limb(s), dose site	49
			terminal kill.	65
M	2	22	General Observation, No Abnormality Detected.	-7-0,7,14,21,28,35,42,49,56,63
			No dosing abnormalities.	0(Pre)-20,21(Pre)-41,42(Pre)-62, 63(Pre)-65
M	2	23	General Observation, No Abnormality Detected.	-7-0,7,14,21,28,35,42,49,56,63
			No dosing abnormalities.	0(Pre)-20,21(Pre)-41,42(Pre)-62, 63(Pre)-65
M	2	24	General Observation, No Abnormality Detected.	-7-0,7,14,21,28,35,42,49,56,63
			No dosing abnormalities.	0(Pre)-20,21(Pre)-41,42(Pre)-62, 63(Pre)-65
M	3	7	General Observation, No Abnormality Detected.	-7-0,7,14,21,28,35,42,49,56,63
			No dosing abnormalities.	0(Pre)-20,21(Pre)-41,42(Pre)-62, 63(Pre)-65
			terminal kill.	65
M	3	8	General Observation, No Abnormality Detected.	-7-0,7,14,21,28,35,42,49,56,63
			No dosing abnormalities.	0(Pre)-20,21(Pre)-41,42(Pre)-62, 63(Pre)-65
			terminal kill.	65
M	3	9	General Observation, No Abnormality Detected.	-7-0,7,14,21,28,35,42,49,56,63
			No dosing abnormalities.	0(Pre)-20,21(Pre)-41,42(Pre)-62, 63(Pre)-65
			terminal kill.	65
M	3	25	General Observation, No Abnormality Detected.	-7-0,7,14,21,28,35,42,49,56,63
			No dosing abnormalities.	0(Pre)-20,21(Pre)-41,42(Pre)-62, 63(Pre)-65
M	3	26	General Observation, No Abnormality Detected.	-7-0,7,14,21,28,35,42,49,56,63

Appendix 3 (continued)
Individual Clinical Observations: Treatment Period

Group	:	1	2	3
Test Item	:	Control	MenPF-1	MenPF-1
Dosage (µg/dose)	:	0	25	50

Sex	Group	Animal	Observation	Days
M	3	26	No dosing abnormalities.	0(Pre)-20,21(Pre)-41,42(Pre)-62, 63(Pre)-65
M	3	27	General Observation, No Abnormality Detected.	-7-0,7,14,21,42,49,56,63
			No dosing abnormalities.	0(Pre)-20,21(Pre)-41,42(Pre)-62, 63(Pre)-65
			discoloured skin on, limb(s), left hind limb	35
			One lesion(s) on, limb(s), left hind	21(IPD),22-23
			One scab(s) on, limb(s), left hind limb	24-25,28
F	1	10	General Observation, No Abnormality Detected.	-7-0,7,14,21,28,35,42,49,56,63
			No dosing abnormalities.	0(Pre)-20,21(Pre)-41,42(Pre)-62, 63(Pre)-65
			terminal kill.	65
F	1	11	General Observation, No Abnormality Detected.	-7-0,7,14,21,28,35,42,49,56,63
			No dosing abnormalities.	0(Pre)-20,21(Pre)-41,42(Pre)-62, 63(Pre)-65
			terminal kill.	65
F	1	12	General Observation, No Abnormality Detected.	-7-0,7,14,21,28,35,42,49,56,63
			No dosing abnormalities.	0(Pre)-20,21(Pre)-41,42(Pre)-62, 63(Pre)-65
			terminal kill.	65
F	1	28	General Observation, No Abnormality Detected.	-7-0,7,14,21,28,35,42,49,56,63
			No dosing abnormalities.	0(Pre)-20,21(Pre)-41,42(Pre)-62, 63(Pre)-65
F	1	29	General Observation, No Abnormality Detected.	14,21,28,35,42,49,56,63
			No dosing abnormalities.	0(Pre)-20,21(Pre)-41,42(Pre)-62, 63(Pre)-65
			One area(s) of sparse hair	-7-0,7
F	1	30	General Observation, No Abnormality Detected.	-7-0,7,14,21,28,35,42,49,56,63
			No dosing abnormalities.	0(Pre)-20,21(Pre)-41,42(Pre)-62, 63(Pre)-65
F	2	13	General Observation, No Abnormality Detected.	-7-0,7,14,21,28,35,49,56,63

Appendix 3 (continued)
Individual Clinical Observations: Treatment Period

Group	:	1	2	3
Test Item	:	Control	MenPF-1	MenPF-1
Dosage (µg/dose)	:	0	25	50

Sex	Group	Animal	Observation	Days
F	2	13	Muzzle, swollen	40,42
			No dosing abnormalities.	0(Pre)-20,21(Pre)-41,42(Pre)-62, 63(Pre)-65
			One scab(s) on, muzzle	38,40
			terminal kill.	65
F	2	14	General Observation, No Abnormality Detected.	-7-0,7,14,21,28,35,42,49,56,63
			No dosing abnormalities.	0(Pre)-20,21(Pre)-41,42(Pre)-62, 63(Pre)-65
			terminal kill.	65
F	2	15	General Observation, No Abnormality Detected.	-7-0,7,14,21,28,35,42,49,56,63
			No dosing abnormalities.	0(Pre)-20,21(Pre)-41,42(Pre)-62, 63(Pre)-65
			terminal kill.	65
F	2	31	General Observation, No Abnormality Detected.	-7-0,7,14,21,28,35,42,49,56,63
			No dosing abnormalities.	0(Pre)-20,21(Pre)-41,42(Pre)-62, 63(Pre)-65
F	2	32	General Observation, No Abnormality Detected.	-7-0,7,14,21,28,35,42,49,56,63
			No dosing abnormalities.	0(Pre)-20,21(Pre)-21(+3H), 49-62,63(Pre)-65
			Skin, scab at dose site 1	22-27
			Skin, discoloured dose site 1	28-41,42(Pre)-48
F	2	33	General Observation, No Abnormality Detected.	-7-0,7,14,21,28,35,42,49,56,63
			No dosing abnormalities.	0(Pre)-20,21(Pre)-41,42(Pre)-62, 63(Pre)-65
F	3	16	General Observation, No Abnormality Detected.	-7-0,7,14,21,28,35,42,49,56,63
			No dosing abnormalities.	0(Pre)-20,21(Pre)-41,42(Pre)-62, 63(Pre)-65
			terminal kill.	65
F	3	17	General Observation, No Abnormality Detected.	-7-0,7,14,21,28,35,42,49,56,63
			No dosing abnormalities.	0(Pre)-20,21(Pre)-41,42(Pre)-62, 63(Pre)-65

Appendix 3 (continued)
Individual Clinical Observations: Treatment Period

Group	:	1	2	3
Test Item	:	Control	MenPF-1	MenPF-1
Dosage (µg/dose)	:	0	25	50

Sex	Group	Animal	Observation	Days
F	3	17	terminal kill.	65
F	3	18	General Observation, No Abnormality Detected.	-7-0,7,14,21,28,35,42,49,56,63
			No dosing abnormalities.	0(Pre)-20,21(Pre)-41,42(Pre)-62, 63(Pre)-65
			terminal kill.	65
F	3	34	General Observation, No Abnormality Detected.	-7-0,7,14,21,28,35,42,49,56,63
			No dosing abnormalities.	0(Pre)-20,21(Pre)-41,42(Pre)-62, 63(Pre)-65
F	3	35	General Observation, No Abnormality Detected.	-7-0,7,14,21,28,35,42,49,56,63
			No dosing abnormalities.	0(Pre)-20,21(Pre)-41,42(Pre)-62, 63(Pre)-65
F	3	36	General Observation, No Abnormality Detected.	-7-0,7,14,21,28,35,42,49,56,63
			No dosing abnormalities.	0(Pre)-20,21(Pre)-41,42(Pre)-62, 63(Pre)-65

Appendix 4
Individual Clinical Observations: Recovery Period

Group	:	1	2	3
Test Item	:	Control	MenPF-1	MenPF-1
Dosage (µg/dose)	:	0	25	50

Key to Appendix 4

Pre = Predose

The in-life data capture system records the first day of treatment as Day 0.

Appendix 4 (continued)

Individual Clinical Observations: Recovery Period

Group	:	1	2	3
Test Item	:	Control	MenPF-1	MenPF-1
Dosage (µg/dose)	:	0	25	50

Sex	Group	Animal	Observation	Days
M	1	19	General Observation, No Abnormality Detected.	63,70,77,84,91
			No dosing abnormalities.	63(Pre)-91
			recovery kill.	91
M	1	20	General Observation, No Abnormality Detected.	63,70,77,84,91
			No dosing abnormalities.	63(Pre)-91
			recovery kill.	91
M	1	21	General Observation, No Abnormality Detected.	63,70,77,84,91
			No dosing abnormalities.	63(Pre)-91
			recovery kill.	91
M	2	22	General Observation, No Abnormality Detected.	63,70,77,84,91
			No dosing abnormalities.	63(Pre)-91
			recovery kill.	91
M	2	23	General Observation, No Abnormality Detected.	63,70,77,84,91
			No dosing abnormalities.	63(Pre)-91
			recovery kill.	91
M	2	24	General Observation, No Abnormality Detected.	63,70,77,84,91
			No dosing abnormalities.	63(Pre)-91
			recovery kill.	91
M	3	25	General Observation, No Abnormality Detected.	63,70,77,84,91
			No dosing abnormalities.	63(Pre)-91
			recovery kill.	91
M	3	26	General Observation, No Abnormality Detected.	63,70,77,84,91
			No dosing abnormalities.	63(Pre)-91
			recovery kill.	91
M	3	27	General Observation, No Abnormality Detected.	63,70,77,84,91
			No dosing abnormalities.	63(Pre)-91
			recovery kill.	91
F	1	28	General Observation, No Abnormality Detected.	63,70,77,84,91
			No dosing abnormalities.	63(Pre)-91
			recovery kill.	91
F	1	29	General Observation, No Abnormality Detected.	63,70,77,84,91

Appendix 4 (continued)
Individual Clinical Observations: Recovery Period

Group	:	1	2	3
Test Item	:	Control	MenPF-1	MenPF-1
Dosage (µg/dose)	:	0	25	50

Sex	Group	Animal	Observation	Days
F	1	29	No dosing abnormalities.	63(Pre)-91
			recovery kill.	91
F	1	30	General Observation, No Abnormality Detected.	63,70,77,84,91
			No dosing abnormalities.	63(Pre)-91
			recovery kill.	91
F	2	31	General Observation, No Abnormality Detected.	63,70,77,84,91
			No dosing abnormalities.	63(Pre)-91
			recovery kill.	91
F	2	32	General Observation, No Abnormality Detected.	63,70,77,84,91
			No dosing abnormalities.	63(Pre)-91
			recovery kill.	91
F	2	33	General Observation, No Abnormality Detected.	63,70,77,84,91
			No dosing abnormalities.	63(Pre)-91
			recovery kill.	91
F	3	34	General Observation, No Abnormality Detected.	63,70,77,84,91
			No dosing abnormalities.	63(Pre)-91
			recovery kill.	91
F	3	35	General Observation, No Abnormality Detected.	63,70,77,84,91
			No dosing abnormalities.	63(Pre)-91
			recovery kill.	91
F	3	36	General Observation, No Abnormality Detected.	63,70,77,84,91
			No dosing abnormalities.	63(Pre)-91
			recovery kill.	91

Appendix 5
Injection Site Reaction Scores: Individual Findings

Group	:	1	2	3
Test Item	:	Control	MenPF-1	MenPF-1
Dosage (µg/dose)	:	0	25	50

Group / sex	Animal No.	Day 1						Day 22						Day 43						Day 64					
		+ 0 h		+ 24 h		+ 48 h		+ 0 h		+ 24 h		+ 48 h		+ 0 h		+ 24 h		+ 48 h		+ 0 h		+ 24 h		+ 48 h	
		E	O	E	O	E	O	E	O	E	O	E	O	E	O	E	O	E	O	E	O	E	O	E	O
1M	1	0	0	0	0	0	0	0	0	0	0	0	0	0	0	0	0	0	0	0	0	0	0	0	0
	2	0	0	0	0	0	0	0	0	0	0	0	0	0	0	0	0	0	0	0	0	0	0	0	0
	3	0	0	0	0	0	0	0	0	0	0	0	0	0	0	0	0	0	0	0	0	0	0	0	0
	19	0	0	0	0	0	0	0	0	0	0	0	0	0	0	0	0	0	0	0	0	0	0	0	0
	20	0	0	0	0	0	0	0	0	0	0	0	0	0	0	0	0	0	0	0	0	0	0	0	0
	21	0	0	0	0	0	0	0	0	0	0	0	0	0	0	0	0	0	0	0	0	0	0	0	0
2M	4	0	0	0	0	0	0	0	0	0	0	0	0	0	0	0	0	0	0	0	0	0	0	0	0
	5	0	0	0	0	0	0	0	0	0	0	0	0	0	0	0	0	0	0	0	0	0	0	0	0
	6	0	0	0	0	0	0	0	0	0	0	0	0	0	0	1	0	0	0	0	0	0	0	0	0
	22	0	0	0	0	0	0	0	0	0	0	0	0	0	0	0	0	0	0	0	0	0	0	0	0
	23	0	0	0	0	0	0	0	0	0	0	0	0	0	0	0	0	0	0	0	0	0	0	0	0
	24	0	0	0	0	0	0	0	0	0	0	0	0	0	0	0	0	0	0	0	0	0	0	0	0
3M	7	0	0	0	0	0	0	0	0	0	0	0	0	0	0	0	0	0	0	0	0	0	0	0	0
	8	0	0	0	0	0	0	0	0	0	0	0	0	0	0	0	0	0	0	0	0	0	0	0	0
	9	0	0	0	0	0	0	0	0	0	0	0	0	0	0	0	0	0	0	0	0	0	0	0	0
	25	0	0	0	0	0	0	0	0	0	0	0	0	0	0	0	0	0	0	0	0	0	0	0	0
	26	0	0	0	0	0	0	0	0	0	0	0	0	0	0	0	0	0	0	0	0	0	0	0	0
	27	0	0	0	0	0	0	0	0	0	0	0	0	0	0	0	0	0	0	0	0	0	0	0	0

E = Erythema (0 = None, 1 = very slight, barely perceptible); O = Oedema (0 = none)

Appendix 5 (continued)

Injection Site Reaction Scores: Individual Findings

Group	:	1	2	3
Test Item	:	Control	MenPF-1	MenPF-1
Dosage (µg/dose)	:	0	25	50

Group / sex	Animal No.	Day 1						Day 22						Day 43						Day 64					
		+ 0 h		+ 24 h		+ 48 h		+ 0 h		+ 24 h		+ 48 h		+ 0 h		+ 24 h		+ 48 h		+ 0 h		+ 24 h		+ 48 h	
		E	O	E	O	E	O	E	O	E	O	E	O	E	O	E	O	E	O	E	O	E	O	E	O
1F	10	0	0	0	0	0	0	0	0	0	0	0	0	0	0	0	0	0	0	0	0	0	0	0	0
	11	0	0	0	0	0	0	0	0	0	0	0	0	0	0	0	0	0	0	0	0	0	0	0	0
	12	0	0	0	0	0	0	0	0	0	0	0	0	0	0	0	0	0	0	0	0	0	0	0	0
	28	0	0	0	0	0	0	0	0	0	0	0	0	0	0	0	0	0	0	0	0	0	0	0	0
	29	0	0	0	0	0	0	0	0	0	0	0	0	0	0	1	0	0	0	0	0	0	0	0	0
	30	0	0	0	0	0	0	0	0	0	0	0	0	0	0	0	0	0	0	0	0	0	0	0	0
2F	13	0	0	0	0	0	0	0	0	0	0	0	0	0	0	0	0	0	0	0	0	0	0	0	0
	14	0	0	0	0	0	0	0	0	0	0	1	0	0	0	0	0	0	0	0	0	0	0	0	0
	15	0	0	0	0	0	0	0	0	0	0	0	0	0	0	0	0	0	0	0	0	0	0	0	0
	31	0	0	0	0	0	0	0	0	1	0	0	0	0	0	0	0	0	0	0	0	0	0	0	0
	32[a]	0	0	0	0	0	0	0	0	1	1	1	0	0	0	0	0	0	0	0	0	0	0	0	0
	33	0	0	0	0	0	0	0	0	0	0	0	0	0	0	0	0	0	0	0	0	0	0	0	0
3F	16	0	0	0	0	0	0	0	0	0	0	0	0	0	0	0	0	0	0	0	0	0	0	0	0
	17	0	0	0	0	0	0	0	0	0	0	0	0	0	0	0	0	0	0	0	0	0	0	0	0
	18[b]	0	0	0	0	0	0	0	0	1	0	1	0	0	0	0	0	0	0	0	0	0	0	0	0
	34	0	0	0	0	0	0	0	0	0	0	0	0	0	0	0	0	0	0	0	0	0	0	0	0
	35	0	0	0	0	0	0	0	0	0	0	0	0	0	0	0	0	0	0	0	0	0	0	0	0
	36	0	0	0	0	0	0	0	0	0	0	0	0	0	0	0	0	0	0	0	0	0	0	0	0

E = Erythema (0 = None, 1 = very slight, barely perceptible); O = Oedema (0 = None, 1 = very slight, barely perceptible)

[a]Animal 32 – Erythema recorded up to 120 hours following dose on Day 22; results maintained in the study data

[b]Animal 18 – Erythema recorded up to 72 hours following dose on Day 22; results maintained in the study data

Appendix 6
Body Weights with Change (kg): Individual Values: Treatment Period

Group	:	1	2	3
Test Item	:	Control	MenPF-1	MenPF-1
Dosage (µg/dose)	:	0	25	50

Group / sex	Animal No.	Day -7	0	3	7	10	14	17	21	24	28	31	35	38
1M	1	2.6	2.7	2.8	2.8	2.8	2.9	2.9	2.9	2.9	2.9	2.9	3.0	3.0
	2	2.5	2.7	2.7	2.8	2.8	2.9	3.0	3.0	3.0	3.0	3.0	3.0	3.1
	3	2.6	2.8	2.8	2.8	2.9	2.9	3.0	3.0	3.0	3.1	3.1	3.1	3.1
	19	2.7	2.8	2.9	2.9	3.0	3.0	3.1	3.2	3.2	3.2	3.3	3.3	3.3
	20	2.6	2.7	2.8	2.8	2.9	3.0	3.0	3.0	3.0	3.1	3.1	3.1	3.1
	21	2.7	2.8	2.8	2.8	2.9	2.9	3.0	3.0	3.1	3.1	3.1	3.1	3.1
2M	4	2.6	2.8	2.8	2.9	3.0	3.1	3.1	3.2	3.3	3.3	3.4	3.4	3.4
	5	2.8	2.8	2.8	2.9	2.9	3.0	3.0	3.0	3.0	3.2	3.1	3.2	3.2
	6	2.6	2.7	2.8	2.9	2.9	3.0	3.0	3.1	3.1	3.2	3.2	3.3	3.2
	22	2.6	2.8	2.9	3.0	3.1	3.1	3.2	3.2	3.3	3.3	3.3	3.4	3.4
	23	2.6	2.7	2.8	2.9	2.9	3.0	3.0	3.0	3.1	3.1	3.1	3.2	3.2
	24	2.6	2.7	2.8	2.8	2.9	2.9	2.9	3.0	3.0	3.0	3.1	3.1	3.1
3M	7	2.6	2.7	2.8	2.9	2.9	3.1	3.1	3.2	3.2	3.2	3.2	3.3	3.3
	8	2.4	2.6	2.7	2.8	2.9	3.0	2.9	2.9	3.0	3.0	3.0	3.1	3.1
	9	2.7	2.8	2.8	2.9	2.9	3.0	3.0	3.0	3.1	3.1	3.2	3.2	3.2
	25	2.7	2.8	2.9	2.9	3.0	3.1	3.1	3.1	3.1	3.1	3.2	3.2	3.2
	26	2.6	2.7	2.7	2.8	2.8	2.8	2.8	2.9	2.9	2.9	2.9	3.0	2.9
	27	2.7	2.8	2.9	3.0	3.0	3.2	3.1	3.2	3.2	3.3	3.2	3.3	3.3

Appendix 6 (continued)
Body Weights with Change (kg): Individual Values: Treatment Period

Group	:	1	2	3
Test Item	:	Control	MenPF-1	MenPF-1
Dosage (µg/dose)	:	0	25	50

Group / sex	Animal No.	Day 42	45	49	52	56	59	63	Change 0 - 63
1M	1	3.0	3.0	3.1	3.1	3.1	3.1	3.1	0.4
	2	3.1	3.1	3.2	3.2	3.2	3.3	3.3	0.6
	3	3.1	3.1	3.2	3.3	3.2	3.2	3.2	0.4
	19	3.4	3.4	3.4	3.5	3.5	3.6	3.5	0.7
	20	3.1	3.1	3.2	3.2	3.2	3.2	3.3	0.6
	21	3.1	3.1	3.2	3.2	3.2	3.2	3.2	0.4
2M	4	3.5	3.5	3.5	3.5	3.5	3.5	3.5	0.7
	5	3.3	3.3	3.3	3.3	3.3	3.3	3.3	0.5
	6	3.2	3.3	3.3	3.4	3.4	3.4	3.4	0.7
	22	3.4	3.4	3.5	3.5	3.6	3.6	3.6	0.8
	23	3.2	3.2	3.2	3.2	3.3	3.3	3.3	0.6
	24	3.1	3.1	3.1	3.1	3.2	3.3	3.2	0.5
3M	7	3.4	3.5	3.5	3.5	3.6	3.6	3.6	0.9
	8	3.1	3.2	3.2	3.2	3.2	3.3	3.2	0.6
	9	3.3	3.3	3.4	3.4	3.4	3.4	3.4	0.6
	25	3.2	3.2	3.2	3.2	3.3	3.3	3.2	0.4
	26	2.9	3.0	3.0	3.0	3.0	3.0	3.0	0.3
	27	3.3	3.4	3.4	3.4	3.4	3.4	3.4	0.6

Appendix 6 (continued)

Body Weights with Change (kg): Individual Values: Treatment Period

Group	:	1	2	3
Test Item	:	Control	MenPF-1	MenPF-1
Dosage (µg/dose)	:	0	25	50

Group / sex	Animal No.	Day -7	0	3	7	10	14	17	21	24	28	31	35	38
1F	10	2.6	2.8	2.8	3.0	3.0	3.2	3.3	3.3	3.4	3.5	3.5	3.6	3.5
	11	2.7	2.9	3.0	3.2	3.2	3.3	3.4	3.5	3.5	3.6	3.6	3.7	3.7
	12	2.6	2.8	2.8	2.9	3.0	3.0	3.1	3.1	3.2	3.3	3.3	3.4	3.4
	28	2.7	2.9	3.0	3.1	3.1	3.3	3.3	3.4	3.4	3.4	3.5	3.6	3.6
	29	2.7	2.9	3.0	3.0	3.1	3.2	3.3	3.3	3.4	3.5	3.5	3.6	3.7
	30	2.9	3.1	3.2	3.4	3.5	3.6	3.8	3.9	4.0	4.0	4.1	4.1	4.1
2F	13	2.7	2.8	2.9	3.0	3.0	3.1	3.2	3.2	3.2	3.3	3.4	3.4	3.5
	14	2.6	2.7	2.8	2.9	3.0	3.1	3.2	3.2	3.3	3.3	3.4	3.5	3.5
	15	2.8	3.0	3.2	3.3	3.4	3.5	3.6	3.7	3.7	3.8	3.9	3.9	3.9
	31	2.5	2.7	2.8	3.0	2.9	3.1	3.1	3.2	3.3	3.3	3.4	3.4	3.4
	32	2.5	2.7	2.8	2.9	3.0	3.1	3.2	3.3	3.3	3.4	3.4	3.5	3.5
	33	2.6	2.8	2.8	3.0	3.0	3.1	3.1	3.2	3.2	3.3	3.4	3.4	3.4
3F	16	2.6	2.7	2.8	2.8	2.9	2.9	3.0	3.0	3.0	3.0	3.1	3.1	3.2
	17	2.9	3.0	3.0	3.2	3.3	3.4	3.4	3.5	3.5	3.7	3.6	3.8	3.8
	18	2.4	2.7	2.8	3.0	3.1	3.3	3.4	3.4	3.5	3.6	3.7	3.8	3.9
	34	2.8	2.9	3.0	3.1	3.2	3.3	3.4	3.4	3.5	3.6	3.6	3.7	3.7
	35	2.7	3.0	3.1	3.3	3.3	3.5	3.5	3.6	3.7	3.8	3.8	3.9	3.9
	36	2.9	3.1	3.3	3.4	3.5	3.6	3.6	3.7	3.7	3.8	3.9	3.9	3.9

Appendix 6 (continued)
Body Weights with Change (kg): Individual Values: Treatment Period

Group	:	1	2	3
Test Item	:	Control	MenPF-1	MenPF-1
Dosage (µg/dose)	:	0	25	50

Group / sex	Animal No.	Day 42	45	49	52	56	59	63	Change 0 - 63
1F	10	3.7	3.6	3.7	3.8	3.9	3.9	3.9	1.1
	11	3.8	3.8	3.9	3.9	4.0	4.1	4.1	1.2
	12	3.5	3.6	3.6	3.7	3.7	3.8	3.8	1.0
	28	3.6	3.7	3.7	3.8	3.9	4.0	3.9	1.0
	29	3.7	3.8	3.8	3.8	3.9	4.0	4.0	1.1
	30	4.3	4.3	4.4	4.5	4.6	4.6	4.6	1.5
2F	13	3.5	3.5	3.4	3.5	3.6	3.6	3.6	0.8
	14	3.5	3.5	3.5	3.6	3.7	3.8	3.8	1.1
	15	4.1	4.0	4.1	4.2	4.3	4.4	4.4	1.4
	31	3.5	3.6	3.6	3.6	3.7	3.7	3.7	1.0
	32	3.6	3.7	3.7	3.7	3.8	3.9	3.9	1.2
	33	3.4	3.4	3.2	3.2	3.3	3.5	3.5	0.7
3F	16	3.1	3.2	3.2	3.2	3.2	3.2	3.2	0.5
	17	3.8	3.9	3.9	3.9	3.9	4.0	4.0	1.0
	18	3.9	4.0	4.0	4.1	4.2	4.2	4.2	1.5
	34	3.7	3.8	3.9	3.9	3.9	4.0	4.0	1.1
	35	4.0	4.0	4.1	4.2	4.2	4.2	4.0	1.0
	36	4.0	4.1	4.2	4.2	4.2	4.3	4.3	1.2

Appendix 7
Body Weights with Change (kg): Individual Values: Recovery Period

Group	:	1	2	3
Test Item	:	Control	MenPF-1	MenPF-1
Dosage (µg/dose)	:	0	25	50

Group / sex	Animal No.	Day 66	70	73	77	80	84	87	91	Change 66 - 91
1M	19	3.6	3.6	3.7	3.7	3.7	3.7	3.7	3.8	0.2
	20	3.2	3.3	3.3	3.3	3.3	3.4	3.4	3.5	0.3
	21	3.3	3.3	3.4	3.4	3.4	3.4	3.3	3.5	0.2
2M	22	3.6	3.6	3.6	3.7	3.6	3.7	3.7	3.7	0.1
	23	3.3	3.3	3.3	3.4	3.3	3.4	3.3	3.4	0.1
	24	3.3	3.2	3.3	3.3	3.3	3.3	3.2	3.3	0.0
3M	25	3.2	3.2	3.2	3.3	3.3	3.4	3.3	3.4	0.2
	26	3.0	2.9	3.0	3.0	2.9	3.0	3.0	3.0	0.0
	27	3.4	3.4	3.4	3.4	3.3	3.5	3.4	3.5	0.1

Appendix 7 (continued)
Body Weights with Change (kg): Individual Values: Recovery Period

Group	:	1	2	3
Test Item	:	Control	MenPF-1	MenPF-1
Dosage (µg/dose)	:	0	25	50

Group / sex	Animal No.	Day 66	70	73	77	80	84	87	91	Change 66 - 91
1F	28	4.0	4.0	4.0	4.1	4.2	4.2	4.2	4.2	0.2
	29	4.0	4.1	4.1	4.2	4.1	4.3	4.2	4.3	0.3
	30	4.7	4.7	4.7	4.9	4.9	5.0	5.0	5.0	0.3
2F	31	3.8	3.7	3.8	3.9	3.9	3.9	3.9	3.9	0.1
	32	3.9	3.9	3.9	4.1	4.0	4.1	4.0	4.1	0.2
	33	3.5	3.4	3.4	3.5	3.4	3.5	3.4	3.5	0.0
3F	34	4.1	4.0	4.1	4.2	4.2	4.2	4.2	4.3	0.2
	35	4.0	4.0	4.2	4.3	4.2	4.3	4.4	4.4	0.4
	36	4.4	4.4	4.4	4.5	4.5	4.5	4.5	4.6	0.2

Appendix 8
Food Consumption (g/animal/day): Individual Values: Treatment Period

	:			
Group	:	1	2	3
Test Item	:	Control	MenPF-1	MenPF-1
Dosage (µg/dose)	:	0	25	50

Group / sex	Animal No.	Day -4	0	3	7	10	14	17	21	24	28	31	35	38
1M	1	112.3	114.0	103.6	117.1	87.7	114.5	100.0	122.8	101.0	108.3	91.0	116.3	91.7
	2	122.0	117.3	115.0	125.0	132.3	135.0	130.0	132.8	123.0	123.8	123.0	138.8	121.7
	3	126.7	111.0	121.8	110.7	116.0	133.5	131.7	130.3	121.0	126.5	140.3	140.0	139.0
	19	142.3	151.5	133.5	124.1	117.7	148.0	145.7	156.8	149.7	148.8	146.3	157.0	153.7
	20	142.0	141.8	143.9	141.1	128.0	144.3	118.3	145.3	110.7	130.3	134.3	122.3	123.7
	21	121.7	137.8	122.4	130.5	136.3	155.8	137.3	150.8	141.3	154.8	114.3	125.5	137.7
2M	4	153.3	149.0	136.6	138.8	147.3	148.8	142.7	161.0	135.3	155.5	161.3	139.8	129.0
	5	152.0	148.0	147.8	141.7	145.7	160.8	140.7	145.0	135.0	152.3	139.7	136.5	139.0
	6	136.7	150.5	146.1	154.7	151.7	162.5	147.7	152.0	146.0	148.3	137.0	147.3	134.0
	22	145.0	149.3	154.2	163.6	157.7	182.0	141.7	174.5	150.7	148.0	150.3	162.8	151.0
	23	134.7	131.5	109.7	126.3	109.0	124.0	113.3	131.0	111.0	108.5	110.0	111.8	105.7
	24	122.0	116.5	100.5	115.4	95.0	116.3	103.7	109.8	102.0	97.8	108.0	112.3	124.7
3M	7	155.3	138.8	132.2	146.3	132.7	154.0	152.3	162.8	152.0	138.8	135.7	139.3	134.0
	8	98.7	112.5	115.6	114.3	122.3	122.3	124.7	128.0	124.3	118.3	115.7	119.0	119.0
	9	137.0	130.0	124.5	131.6	132.7	135.8	132.7	138.3	122.7	135.5	145.3	131.3	138.0
	25	150.3	143.8	118.3	132.1	124.7	142.3	122.0	132.0	109.7	135.5	130.7	120.8	119.7
	26	122.3	123.0	124.2	127.8	111.3	119.3	109.0	123.3	106.7	119.8	108.0	114.8	100.3
	27	131.7	126.5	120.9	144.6	145.0	153.0	146.3	140.5	117.0	124.3	122.7	135.8	145.7

Appendix 8 (continued)
Food Consumption (g/animal/day): Individual Values: Treatment Period

Group	:	1	2	3
Test Item	:	Control	MenPF-1	MenPF-1
Dosage (µg/dose)	:	0	25	50

Group / sex	Animal No.	Day 42	45	49	52	56	59	63
1M	1	110.0	98.8	100.5	119.7	118.3	107.3	116.5
	2	126.3	132.4	135.5	140.3	133.8	130.3	133.0
	3	137.0	128.5	130.3	143.7	117.0	134.0	139.3
	19	161.7	160.5	164.8	175.7	165.3	157.0	166.0
	20	125.9	119.1	136.0	125.0	135.3	127.0	137.5
	21	129.6	132.6	147.8	146.0	140.3	133.7	153.3
2M	4	144.7	141.0	143.3	141.0	130.5	138.0	136.5
	5	163.4	126.4	139.0	155.3	149.0	150.7	151.3
	6	124.1	138.1	128.0	158.3	145.8	169.7	156.0
	22	158.2	158.9	157.3	165.3	160.3	161.3	152.8
	23	123.0	93.0	112.3	98.7	113.5	122.7	110.8
	24	112.6	93.7	108.0	106.3	118.5	132.3	127.3
3M	7	151.3	136.5	145.3	144.7	154.8	144.3	150.8
	8	133.4	134.1	128.8	140.3	137.0	148.7	130.3
	9	142.3	130.7	131.3	131.3	139.8	139.0	118.5
	25	129.0	115.2	117.5	127.7	128.5	124.7	111.3
	26	93.4	99.8	112.0	111.3	100.3	102.7	103.5
	27	137.4	123.1	139.3	144.0	119.3	121.7	117.8

Appendix 8 (continued)
Food Consumption (g/animal/day): Individual Values: Treatment Period

Group	:	1	2	3
Test Item	:	Control	MenPF-1	MenPF-1
Dosage (µg/dose)	:	0	25	50

Group / sex	Animal No.	Day -4	0	3	7	10	14	17	21	24	28	31	35	38
1F	10	149.3	161.0	117.1	174.7	142.7	163.8	162.7	180.8	152.7	170.0	171.7	140.8	153.0
	11	151.0	154.8	142.7	171.5	142.7	168.0	157.0	173.5	157.0	165.3	160.7	171.0	158.3
	12	121.0	126.8	123.5	110.1	121.7	133.0	108.0	133.3	122.0	119.8	121.3	142.0	123.3
	28	150.0	150.3	130.6	152.6	129.3	155.5	142.0	151.3	141.3	140.0	140.0	138.3	147.3
	29	158.3	154.0	143.0	155.5	130.3	166.8	157.7	158.5	144.3	167.0	157.3	177.0	169.3
	30	165.3	180.3	177.2	195.4	198.0	209.8	195.0	212.5	198.3	205.3	183.3	198.8	205.7
2F	13	120.0	155.3	136.9	145.4	125.7	153.8	149.0	160.0	122.0	140.3	150.7	143.3	131.7
	14	147.0	156.8	134.7	162.2	140.7	162.5	147.7	159.8	134.3	158.8	160.7	177.0	156.0
	15	129.7	159.0	159.3	169.0	172.7	189.3	164.0	171.5	171.3	171.8	186.0	179.3	183.7
	31	126.7	136.5	122.9	145.1	144.3	151.0	120.0	149.3	133.7	129.3	113.0	156.3	126.3
	32	120.3	137.0	132.2	147.9	136.3	162.8	151.7	184.0	148.0	144.3	143.3	169.3	159.0
	33	127.3	150.0	130.3	140.4	148.3	146.0	132.3	153.0	133.3	143.0	129.3	155.0	134.0
3F	16	129.3	117.5	113.3	122.6	119.3	110.5	123.0	116.0	105.7	110.3	116.7	115.5	115.0
	17	159.7	174.5	148.2	163.1	161.0	176.3	156.3	178.0	157.3	172.5	151.7	182.8	166.0
	18	135.3	155.3	180.9	205.1	183.0	247.0	201.3	213.5	200.7	211.5	222.3	222.8	212.0
	34	164.3	168.5	150.4	158.3	148.0	172.5	162.7	165.3	162.3	168.3	157.0	161.0	162.7
	35	184.7	187.5	176.4	203.7	188.0	202.3	181.3	213.8	187.0	202.5	204.0	206.3	206.3
	36	182.3	184.8	172.7	205.4	174.3	199.3	185.3	198.3	169.3	186.8	185.3	194.8	185.3

Appendix 8 (continued)
Food Consumption (g/animal/day): Individual Values: Treatment Period

Group	:	1	2	3
Test Item	:	Control	MenPF-1	MenPF-1
Dosage (µg/dose)	:	0	25	50

Group / sex	Animal No.	Day 42	45	49	52	56	59	63
1F	10	166.8	163.5	149.8	168.0	171.5	169.7	160.5
	11	166.2	161.0	174.0	171.0	179.8	183.7	183.8
	12	138.2	156.7	157.0	134.0	159.3	154.7	151.0
	28	158.6	159.2	146.8	154.7	162.3	156.3	147.0
	29	153.2	149.5	172.0	164.7	170.8	172.3	159.5
	30	223.1	206.8	222.3	215.0	236.8	219.0	214.8
2F	13	136.8	120.3	107.5	102.0	135.0	113.7	118.8
	14	134.2	131.9	117.5	142.0	153.3	158.7	142.0
	15	189.9	154.9	178.3	197.0	206.3	192.7	199.3
	31	143.5	137.5	157.8	128.7	135.5	124.3	135.0
	32	173.6	169.2	166.5	164.7	175.8	171.7	165.8
	33	125.9	107.5	77.3	60.3	97.0	160.0	151.8
3F	16	106.2	144.8	125.8	96.0	87.8	122.7	102.3
	17	172.2	160.1	169.0	163.0	153.8	156.7	160.8
	18	223.9	183.7	188.5	199.0	245.5	189.3	179.8
	34	164.3	162.6	162.3	166.0	161.3	160.0	166.8
	35	189.3	168.7	189.8	200.0	187.0	181.0	116.0
	36	200.2	185.7	193.8	191.3	189.0	176.3	194.3

Appendix 9
Food Consumption (g/animal/day): Individual Values: Recovery Period

Group	:	1	2	3
Test Item	:	Control	MenPF-1	MenPF-1
Dosage (µg/dose)	:	0	25	50

Group / sex	Animal No.	Day 66	70	73	77	80	84	87	91
1M	19	179.7	156.5	150.7	163.5	144.0	152.5	137.7	147.0
	20	123.0	120.5	122.3	108.3	100.3	131.8	138.3	145.8
	21	143.0	123.3	92.3	122.3	98.3	135.0	113.0	138.5
2M	22	134.0	133.0	150.0	155.5	135.0	139.8	145.0	157.5
	23	121.0	112.8	105.0	115.8	101.7	105.0	92.7	123.0
	24	125.7	90.5	111.0	119.0	97.3	117.8	97.3	100.0
3M	25	102.0	110.3	107.7	126.3	99.3	126.3	123.3	129.5
	26	104.7	103.0	97.7	96.0	76.3	102.3	92.0	101.3
	27	118.3	112.3	111.7	122.5	93.0	123.8	121.0	127.3

Appendix 9 (continued)
Food Consumption (g/animal/day): Individual Values: Recovery Period

Group	:	1	2	3
Test Item	:	Control	MenPF-1	MenPF-1
Dosage (µg/dose)	:	0	25	50

Group / sex	Animal No.	Day 66	70	73	77	80	84	87	91
1F	28	146.7	141.3	151.7	158.3	152.0	150.0	147.0	156.0
	29	147.3	166.5	162.7	167.8	133.7	170.0	152.3	163.0
	30	198.0	209.0	201.7	207.0	198.3	198.8	198.0	194.0
2F	31	110.7	133.5	114.3	149.0	121.3	132.8	120.3	127.0
	32	146.3	151.0	133.0	172.3	127.0	152.0	124.7	143.3
	33	96.3	108.5	88.3	114.8	88.0	85.5	87.3	108.8
3F	34	160.7	145.3	158.3	164.0	147.0	156.3	141.0	149.3
	35	104.3	144.8	175.3	202.0	147.3	176.0	172.3	182.5
	36	174.0	172.5	164.3	185.5	161.7	184.8	167.0	171.5

Appendix 10
Individual Ophthalmoscopy Findings

Group	:	1	2	3
Test Item	:	Control	MenPF-1	MenPF-1
Dosage (µg/dose)	:	0	25	50

Group	Animal No./Sex		Finding	Timepoint
1	1M	Right eye:	Persistent pupillary membranes in the iris.	Pretrial
		Left eye:	NAD	Pretrial, Week 10
		Right eye:	NAD	Week 10
	21M	Right eye:	NAD	Pretrial, Week 10
		Left eye:	Focal opacity, posterior in the cortex of the lens.	
2	4M	Right eye:	NAD	Pretrial, Week 10
		Left eye:	Focal opacity, posterior in the cortex of the lens.	
	22M	Right eye:	Persistent pupillary membranes in the iris.	Pretrial
		Right eye:	Retinal dysplasia in the fundus.	Pretrial, Week 10
		Left eye:	Retinal dysplasia in the fundus.	Pretrial, Week 10
3	7M	Right eye:	NAD	Pretrial, Week 10
		Left eye:	Focal opacity, posterior in the cortex of the lens.	
	26M	Right eye:	NAD	Pretrial, Week 10
		Left eye:	Focal opacity, posterior in the cortex of the lens.	

NAD – No abnormalities detected
Animals not reported were normal

Appendix 10 (continued)
Individual Ophthalmoscopy Findings

Group	:	1	2	3
Test Item	:	Control	MenPF-1	MenPF-1
Dosage (µg/dose)	:	0	25	50

Group	Animal No./Sex		Finding	Timepoint
1	11F	Right eye:	Persistent pupillary membranes in the iris	Pretrial, Week 10
		Left eye:	NAD	
	30F	Right eye:	NAD	Pretrial, Week 10
		Left eye:	Focal opacity, anterior in the cortex of the lens.	
2	14F	Both eyes:	Persistent pupillary membranes in the iris	Pretrial, Week 10
	32F	Right eye:	Multi-focal opacity, anterior in the cortex of the lens	Pretrial
		Left eye:	Focal opacity, anterior in the cortex of the lens.	Pretrial, Week 10
		Right eye:	NAD	Week 10
	33F	Both eyes:	Focal opacity, posterior in the cortex of the lens	Pretrial, Week 10
3	18F	Both eyes:	Retinal dysplasia in the Fundus.	Pretrial, Week 10
	34F	Right eye:	NAD	Pretrial
		Left eye:	Focal opacity, anterior in the cortex of the lens. Diffuse opacity, posterior in the cortex of the lens	
		Left eye:	Diffuse opacity, posterior in the cortex of the lens	Week 10
	36F	Right eye:	Persistent pupillary membranes in the iris.	Pretrial, Week 10
		Left eye:	NAD	

NAD – No abnormalities detected
Animals not reported were normal

Appendix 11
Body Temperatures (°C): Individual Recordings

Group	:	1	2	3
Test Item	:	Control	MenPF-1	MenPF-1
Dosage (µg/dose)	:	0	25	50

Group / sex	Animal No.	Pretrial	Day 1					Day 22					Day 43				
			IBD	+ 1 h	+ 3 h	+ 24 h	+ 48 h	IBD	+ 1 h	+ 3 h	+ 24 h	+ 48 h	IBD	+ 1 h	+ 3 h	+ 24 h	+ 48 h
1M	1	38.7	39.4	39.1	38.6	35.2	39.5	40.6	37.6	37.7	39.5	37.9	39.5	38.5	38.8	37.0	35.8
	2	37.3	38.6	38.6	36.6	36.6	40.1	39.4	37.6	37.6	39.7	37.1	38.4	36.8	38.5	36.9	37.5
	3	35.3	36.3	38.2	36.8	37.9	37.3	39.6	38.0	37.7	35.2	36.9	39.6	36.5	35.8	36.6	37.6
	19	37.0	39.1	38.8	38.5	40.3	39.9	40.5	36.9	37.8	39.6	38.8	39.9	38.2	39.2	39.4	37.5
	20	36.6	39.5	39.2	39.1	37.1	38.1	35.4	37.9	38.6	38.7	35.6	36.6	38.2	39.1	36.9	37.5
	21	37.3	39.1	38.3	39.0	39.8	39.5	39.9	38.1	39.8	38.5	38.2	40.5	38.1	38.5	38.6	37.6
2M	4	35.0	39.9	37.4	39.8	39.4	39.7	38.3	38.0	39.5	37.5	38.8	40.3	39.4	38.3	38.6	38.3
	5	36.0	39.5	37.9	38.5.	39.1	39.1	40.0	38.4	39.2	40.3	39.2	39.9	39.4	36.0	38.8	38.9
	6	36.2	40.0	38.1	40.1	39.3	37.3	38.7	38.8	36.1	39.5	38.2	39.6	37.8	37.8	39.5	39.9
	22	36.3	37.6	36.1	38.6	39.6	38.1	38.7	38.2	38.4	39.4	38.2	38.4	38.9	37.4	39.1	39.7
	23	36.8	36.1	37.9	37.1	39.3	38.8	38.0	37.5	37.2	36.7	36.2	39.4	38.3	37.6	39.2	38.5
	24	34.9	37.9	38.4	38.6	39.5	38.5	38.2	36.2	39.5	39.5	39.5	38.0	38.4	37.7	39.3	39.8
3M	7	35.1	37.2	38.2	36.4	40.8	39.1	39.6	37.5	38.2	39.0	38.7	38.7	39.5	38.9	39.6	39.7
	8	34.8	37.2	39.0	37.0	38.5	39.2	39.9	38.0	39.3	39.0	39.2	39.6	39.0	38.7	39.0	38.4
	9	34.8	39.4	38.2	36.9	38.5	39.4	40.2	38.5	38.4	38.6	37.1	38.9	38.6	38.9	39.1	39.3
	25	35.7	37.4	38.1	34.8	38.7	39.7	39.7	38.4	38.1	38.9	39.3	38.5	38.1	38.7	38.5	39.4
	26	35.4	35.7	38.4	37.5	37.6	37.5	37.4	37.9	39.8	36.2	38.6	39.5	37.9	38.8	39.4	39.5
	27	34.1	37.1	38.1	36.1	38.1	37.5	39.4	37.2	38.4	39.1	38.9	38.3	36.6	39.0	39.4	39.2

IBD = immediately before dose

Appendix 11 (continued)
Body Temperatures (°C): Individual Recordings

Group	:	1	2	3
Test Item	:	Control	MenPF-1	MenPF-1
Dosage (µg/dose)	:	0	25	50

Group / sex	Animal No.	Day 64				
		IBD	+ 1 h	+ 3 h	+ 24 h	+ 48 h
1M	1	38.8	37.7	39.5	39.0	37.4
	2	39.6	36.2	38.7	39.1	37.9
	3	39.9	36.7	38.6	39.1	37.2
	19	38.1	37.2	38.0	39.4	37.3
	20	36.8	37.0	38.2	38.1	37.0
	21	39.5	36.3	38.3	39.8	38.0
2M	4	39.1	37.4	38.6	39.3	38.0
	5	38.1	37.3	37.3	38.2	38.3
	6	39.3	37.4	38.8	37.4	39.6
	22	38.6	37.9	39.3	37.4	39.5
	23	38.4	37.1	38.0	37.3	38.1
	24	38.6	36.3	38.2	38.4	39.3
3M	7	38.0	39.0	39.2	38.7	39.1
	8	38.0	38.4	38.2	38.6	39.0
	9	38.2	38.3	38.0	38.6	38.9
	25	38.2	38.8	38.0	40.4	38.8
	26	36.6	38.5	38.1	37.4	38.9
	27	39.0	38.0	38.0	38.6	38.8

IBD = immediately before dose

Appendix 11 (continued)
Body Temperatures (°C): Individual Recordings

Group	:	1	2	3
Test Item	:	Control	MenPF-1	MenPF-1
Dosage (µg/dose)	:	0	25	50

Group / sex	Animal No.	Pretrial	Day 1					Day 22					Day 43				
			IBD	+ 1 h	+ 3 h	+ 24 h	+ 48 h	IBD	+ 1 h	+ 3 h	+ 24 h	+ 48 h	IBD	+ 1 h	+ 3 h	+ 24 h	+ 48 h
1F	10	35.2	37.8	38.7	37.1	37.0	38.0	36.5	38.2	35.1	35.2	36.5	39.8	38.1	38.4	37.3	37.4
	11	37.8	39.5	38.8	37.6	37.4	39.9	38.5	38.8	39.5	39.0	37.5	39.8	38.3	39.0	39.2	37.7
	12	35.5	37.7	38.5	36.5	36.4	37.3	37.3	37.4	39.7	36.8	34.8	40.0	38.6	37.4	37.1	38.5
	28	36.1	37.0	38.3	36.9	36.7	37.9	39.3	37.8	39.3	38.1	35.9	39.8	38.5	36.6	39.1	38.7
	29	38.6	39.0	38.1	37.0	40.1	40.0	37.7	37.0	39.4	39.6	37.9	37.5	39.4	37.9	38.7	38.6
	30	37.3	38.3	37.6	38.1	38.2	37.7	38.0	37.2	39.2	38.0	36.5	38.8	38.3	37.3	37.2	38.5
2F	13	34.7	36.8	38.2	39.1	39.6	37.6	39.9	37.7	35.6	39.9	36.6	38.9	37.2	38.7	39.3	39.2
	14	36.7	37.0	37.9	39.0	37.5	39.2	39.7	37.3	35.8	39.1	37.7	40.0	37.5	37.9	39.1	39.2
	15	37.6	37.6	38.4	39.4	38.2	39.6	37.7	38.1	36.5	39.4	38.6	39.3	38.3	37.5	39.1	38.9
	31	34.4	37.6	38.9	37.6	37.5	39.5	39.9	38.4	38.3	39.7	39.1	40.0	37.5	37.5	38.5	38.4
	32	35.2	36.1	37.2	39.7	36.0	37.6	38.7	37.5	40.2	39.6	37.0	38.7	38.6	38.0	39.7	38.5
	33	36.0	37.0	37.0	38.9	39.0	39.0	38.1	37.7	38.4	38.7	39.0	38.6	36.8	37.4	38.6	39.3
3F	16	36.6	37.2	38.5	37.0	37.9	38.8	39.8	38.6	39.6	40.3	39.3	39.5	39.0	38.7	39.3	40.1
	17	37.0	38.8	38.5	38.5	39.8	40.1	40.3	37.7	38.8	38.5	38.9	40.2	38.9	38.7	38.9	39.6
	18	35.1	38.7	38.5	38.5	38.5	37.8	39.7	39.0	38.1	38.6	38.0	39.6	37.3	38.3	38.6	39.6
	34	34.2	38.2	37.3	38.0	38.3	37.7	39.1	37.7	38.9	37.7	37.8	37.5	36.6	37.5	38.3	37.9
	35	35.1	37.4	37.8	39.3	38.8	38.1	38.0	38.5	37.1	36.6	37.2	39.0	36.1	38.5	39.3	38.9
	36	35.0	36.0	38.0	39.0	38.4	37.8	38.5	38.8	39.5	38.0	38.5	38.2	37.8	37.7	37.8	37.5

IBD = immediately before dose

Appendix 11 (continued)
Body Temperatures (°C): Individual Recordings

Group	:	1	2	3
Test Item	:	Control	MenPF-1	MenPF-1
Dosage (µg/dose)	:	0	25	50

Group / sex	Animal No.	Day 64				
		IBD	+ 1 h	+ 3 h	+ 24 h	+ 48 h
1F	10	39.7	36.8	38.5	37.4	37.2
	11	39.3	37.2	39.4	38.8	38.9
	12	38.3	37.0	38.2	37.3	37.8
	28	37.9	36.7	37.5	37.4	37.2
	29	38.0	37.7	38.2	37.2	37.1
	30	38.3	37.7	38.4	37.1	36.8
2F	13	37.7	38.3	37.7	37.7	38.0
	14	38.6	38.2	38.7	38.6	39.7
	15	39.0	38.1	38.6	38.6	37.9
	31	39.1	38.9	38.1	39.0	38.7
	32	38.6	38.9	38.1	37.0	38.8
	33	38.1	38.7	38.1	38.6	38.7
3F	16	36.7	38.9	39.3	38.2	39.4
	17	37.3	38.7	38.0	38.5	38.8
	18	40.3	38.6	38.1	39.0	39.1
	34	39.1	37.8	37.7	39.3	39.1
	35	38.8	38.8	38.8	38.5	39.0
	36	37.9	38.7	38.8	38.4	37.2

IBD = immediately before dose

Appendix 12
Methods, Units and Abbreviations Used for Laboratory Investigations

Group	:	1	2	3
Test Item	:	Control	MenPF-1	MenPF-1
Dosage (µg/dose)	:	0	25	50

Haematology

Parameters	***Methods***	***Units***
Red Blood Cell Count: (RBC)	Siemens, ADVIA 120 haematology analyser developed from Tycko et al 1985, Applied Optics 24(9):1355-1365.	$x10^{12}/L$
Haemoglobin: (Hb)	Siemens, ADVIA 120 haematology analyser obtained from the direct measurements of red cell volume and haemoglobin concentration using the RBC/pH method.	g/dL
Haematocrit: (Hct)	Siemens, ADVIA 120 haematology analyser derived from the measured red cell volume (MCV) and the red cell count (RBC).	L/L
Mean Cell Volume: (MCV)	Siemens, ADVIA 120 haematology analyser derived from mean of RBC volume histogram.	fL
Mean Cell Haemoglobin Concentration: (MCHC)	Siemens, ADVIA 120 haematology analyser. Calculated parameter from haemoglobin concentration, red blood cell count and mean cell volume.	g/dL
Mean Cell Haemoglobin: (MCH)	Siemens, ADVIA 120 haematology analyser. Calculated parameter from haemoglobin concentration and red blood cell count.	pg

Appendix 12 (continued)
Methods, Units and Abbreviations Used for Laboratory Investigations

Group	:	1	2	3
Test Item	:	Control	MenPF-1	MenPF-1
Dosage (µg/dose)	:	0	25	50

Haematology

Parameters	***Methods***	***Units***
Reticulocytes: (Reti)	Siemens, ADVIA 120 haematology analyser measured by light absorption which is proportional to RNA content.	%
Reticulocyte Count: (Ret)	Siemens, ADVIA 120 haematology analyser measured by light absorption which is proportional to RNA content.	$x10^9/L$
Red Cell Distribution Width: (RDW)	Siemens, ADVIA 120 haematology analyser measured from the amount of variation in size or volume of RBC's. This is the coefficient of variation of the RBC volume distribution.	%
Platelet Count: (Plat)	Siemens, ADVIA 120 haematology analyser measured using the MIE theory of light scattering for homogenous spheres.	$x10^9/L$
White Blood Cell Count: (WBC)	Siemens, ADVIA 120 haematology analyser analysed using two angle laser light signals	$x10^9/L$
Neutrophils: (Neut)	Siemens, ADVIA 120 haematology analyser measured quantitatively using both the Peroxidase method and the Basophil/Lobularity method.	$x10^9/L$

Appendix 12 (continued)
Methods, Units and Abbreviations Used for Laboratory Investigations

Group	:	1	2	3
Test Item	:	Control	MenPF-1	MenPF-1
Dosage (µg/dose)	:	0	25	50

Haematology

Parameters	***Methods***	***Units***
Lymphocytes: (Lymp)	Siemens, ADVIA 120 haematology analyser measured quantitatively using both the Peroxidase method and the Basophil/Lobularity method.	$x10^9/L$
Monocytes: (Mono)	Siemens, ADVIA 120 haematology analyser measured quantitatively using both the Peroxidase method and the Basophil/Lobularity method.	$x10^9/L$
Eosinophils: (Eos)	Siemens, ADVIA 120 haematology analyser measured quantitatively using both the Peroxidase method and the Basophil/Lobularity method.	$x10^9/L$
Basophils: (Baso)	Siemens, ADVIA 120 haematology analyser measured quantitatively using both the Peroxidase method and the Basophil/Lobularity method.	$x10^9/L$
Large Unclassified Cells: (LUC)	Siemens, ADVIA 120 haematology analyser measured quantitatively using both the Peroxidase method and the Basophil/Lobularity method.	$x10^9/L$

Appendix 12 (continued)
Methods, Units and Abbreviations Used for Laboratory Investigations

Group	:	1	2	3
Test Item	:	Control	MenPF-1	MenPF-1
Dosage (µg/dose)	:	0	25	50

Coagulation

Parameters	***Methods***	***Units***
Activated Partial Thromboplastin Time: (APTT)	Instrumentation Laboratory, ACL Advance coagulation analyser. Cephalin with micronized silica for the in vitro determination of APTT in plasma	s
Fibrinogen: (Fib)	HemosIL PT-Fibrinogen high sensitivity reagent HS PLUS cat# 0008469810 ©. Calcium thromboplastin (rabbit brain) for the simultaneous in vitro determination of PT and fibrinogen in plasma using ACL Advance/Futura coagulation analyser	mg/dL
Prothrombin Time: (PT)	HemosIL PT-Fibrinogen high sensitivity reagent HS PLUS cat# 0008469810 ©. Calcium thromboplastin (rabbit brain) for the simultaneous in vitro determination of PT and fibrinogen in plasma using ACL Advance/Futura coagulation analyser	s

Appendix 12 (continued)
Methods, Units and Abbreviations Used for Laboratory Investigations

Group	:	1	2	3
Test Item	:	Control	MenPF-1	MenPF-1
Dosage (µg/dose)	:	0	25	50

Clinical Chemistry

Parameters	*Methods*	*Units*
Urea:<br>(Urea)	Roche /Hitachi P Modular 800 Clinical Chemistry Analyser using Roche Test Kit. Urease kinetic UV Assay developed from Talke H, Schubert GE. Klin Wschr 1965;43:174-175.	mmol/L
Glucose:<br>(Glu)	Roche/Hitachi P Modular 800 Clinical Chemistry Analyser using Roche Test Kit . Hexokinase UV assay, Schmidt F H 1961 Klin Wschr 39:1244	mmol/L
Aspartate Aminotransferase:<br>(AST)	Roche/Hitachi P Modular 800 Clinical Chemistry Analyser using Roche Test Kit. IFCC Method.	U/L
Alanine Aminotransferase:<br>(ALT)	Roche/Hitachi P Modular 800 Clinical Chemistry Analyser using Roche Test Kit. IFCC Method.	U/L
Alkaline Phosphatase:<br>(ALP)	Roche/Hitachi P Modular 800 Clinical Chemistry Analyser using Roche Test Kit. IFCC Method.	U/L
Creatine Phosphokinase:<br>(CPK)	Roche/Hitachi P Modular 800 Clinical Chemistry Analyser using Roche Test Kit. IFCC Method. Thomas, L ed. Labor und Diagnose, 4th ed. Marburg: Die Medizinische Verlagsgesellschaft. Szasz G et al. Clin Chem 1976;22:650	U/L

Appendix 12 (continued)
Methods, Units and Abbreviations Used for Laboratory Investigations

Group	:	1	2	3
Test Item	:	Control	MenPF-1	MenPF-1
Dosage (µg/dose)	:	0	25	50

Clinical Chemistry

Parameters	***Methods***	***Units***
Lactate Dehydrogenase: (LDH)	Roche/Hitachi P Modular 800 Clinical Chemistry Analyser using Roche Test Kit. IFCC Method.	U/L
Sodium: (Na)	Roche/Hitachi P Modular 800 Clinical Chemistry Analyser using indirect Ion Selective Electrode. Application of the Nernst equation to an electrode with crown ether membrane type.	mmol/L
Potassium: (K)	Roche/Hitachi P Modular 800 Clinical Chemistry Analyser using indirect Ion Selective Electrode. Application of the Nernst equation to an electrode with valinomycin liquid membrane type.	mmol/L
Chloride: (Cl)	Roche/Hitachi P Modular 800 Clinical Chemistry Analyserusing indirect Ion Selective Electrode. Application of the Nernst equation to an electrode with quaternary ammonium salt ion exchanger.	mmol/L
Total Protein: (TP)	Roche/Hitachi P Modular 800 Clinical Chemistry Analyser using Roche Test Kit. Biuret colorimetric assay for the formation of protein - biuret reagent complex.	g/L

Appendix 12 (continued)
Methods, Units and Abbreviations Used for Laboratory Investigations

Group	:	1	2	3
Test Item	:	Control	MenPF-1	MenPF-1
Dosage (µg/dose)	:	0	25	50

Clinical Chemistry

Parameters	***Methods***	***Units***
Albumin:<br>(Alb)	Roche/Hitachi P Modular 800 Clinical Chemistry Analyser using Roche Test Kit Cat. No.11970909 216. Bromcresol green colorimetric assay with endpoint method. Doumas B.T. et al 1971, Clin Chem Acta 31:87.	g/L
Globulin:<br>(Glob)	Calculated by subtraction of the Albumin concentration from the Total Protein concentration.	g/L
Albumin Globulin Ratio:<br>(AG-R)	Calculated using Clinical Chemistry Plasma/Serum Total Protein and Albumin Concentrations. Calculated Parameter (Albumin/(Total Protein-Albumin))	
Cholesterol:<br>(Chol)	Roche/Hitachi P Modular 800 Clinical Chemistry Analyser using Roche Test Kit. CHOD-PAP colorimetric assay for the measurement of cholesterol in serum or plasma.	mmol/L
Creatinine:<br>(Crea)	Roche/Hitachi P Modular 800 Clinical Chemistry Analyser using Roche Test Kit. Jaffe kinetic colorimetric method. Rate blanked and compensated. Bartels et al 1972, Clin Chem Acta 37:193	µmol/L
Total Bilirubin:<br>(T.Bil)	Roche/Hitachi P Modular 800 Clinical Chemistry Analyser using Roche Test Kit. Modified Jendrassik-Grof colorimetric method in strongly acidic conditions.	µmol/L

Appendix 12 (continued)
Methods, Units and Abbreviations Used for Laboratory Investigations

Group	:	1	2	3
Test Item	:	Control	MenPF-1	MenPF-1
Dosage (µg/dose)	:	0	25	50

Clinical Chemistry

Parameters	***Methods***	***Units***
	Jendrassik L et al. Biochem Z 1938;297:81.	
Calcium:<br>(Ca)	Roche/Hitachi P Modular 800 Clinical Chemistry Analyser using Roche Test Kit. O-cresolphthalein complexone coloimetric assay. Gindler E M and King J D 1972, Am. J. Clin. Pathol. 58:376	mmol/L
Phosphate:<br>(Phos)	Roche/Hitachi P Modular 800 Clinical Chemistry Analyser using Roche Test Kit. Molybdate UV method. Henry, R.J. 1974. pg 723 in "Clinical Chemistry" 2nd Edition.	mmol/L

The limit of quantification (LOQ) for the following assays was observed and reported as follows with the LOQ used to calculate means and standard deviations for values below the LOQ:

Assay	***Limit of Detection***	***Non-detectable values reported as***
Total Bilirubin	1.7 µmol /L	<1.7 µmol /L

Appendix 13
Haematology and Coagulation : Individual Values: Pretrial

Group	:	1	2	3
Test Item	:	Control	MenPF-1	MenPF-1
Dosage (µg/dose)	:	0	25	50

Group / sex	Animal No.	Hb g/dL	RBC $x10^{12}/L$	Hct L/L	MCH pg	MCV fL	MCHC g/dL	RDW %	Reti %	Ret $x10^9/L$	WBC ----------	Neut ----------	Lymph ----------	Mono $x10^9/L$	Eos ----------	Baso ----------
1M	1	13.2	6.72	0.391	19.7	58.1	33.9	11.9	2.0	136	7.50	0.69	6.05	0.09	0.24	0.42
	2	13.1	6.32	0.381	20.7	60.3	34.3	12.3	1.3	83	8.06	1.08	6.20	0.08	0.19	0.50
	3	13.0	6.26	0.388	20.8	62.0	33.5	13.8	1.8	112	6.27	1.27	4.30	0.22	0.10	0.37
	19	12.8	5.77	0.364	22.2	63.1	35.2	13.7	5.1	294	6.18	1.51	3.79	0.19	0.16	0.53
	20	12.4	5.98	0.356	20.7	59.5	34.7	12.8	1.9	116	5.92	0.94	4.25	0.06	0.17	0.50
	21	13.5	6.04	0.382	22.3	63.3	35.3	11.5	2.6	158	5.08	1.01	3.40	0.07	0.15	0.44
2M	4	12.9	6.39	0.379	20.2	59.3	34.0	12.5	1.8	114	5.60	0.91	4.01	0.10	0.13	0.43
	5	13.2	6.12	0.373	21.5	61.0	35.3	12.3	2.2	133	5.43	1.09	3.69	0.00	0.11	0.54
	6	12.7	6.51	0.373	19.4	57.3	33.9	12.5	1.4	91	7.65	2.44	4.31	0.12	0.20	0.55
	22	12.7	5.73	0.360	22.2	62.8	35.3	12.2	1.9	111	7.43	3.48	3.15	0.10	0.14	0.55
	23	13.0	6.42	0.375	20.2	58.4	34.7	12.7	1.9	119	6.25	1.19	4.17	0.10	0.21	0.58
	24	12.1	5.73	0.356	21.2	62.2	34.0	12.7	1.9	107	5.73	0.92	4.20	0.14	0.08	0.38
3M	7	12.3	6.08	0.367	20.2	60.4	33.5	11.6	2.1	126	4.28	0.65	3.17	0.07	0.07	0.31
	8	12.3	6.20	0.364	19.8	58.8	33.7	11.3	1.5	93	6.36	1.46	4.25	0.08	0.09	0.47
	9	13.1	6.19	0.375	21.1	60.7	34.8	13.3	1.8	114	7.09	2.11	4.28	0.07	0.11	0.51
	25	12.4	5.84	0.359	21.2	61.5	34.5	13.6	2.8	162	7.27	1.67	4.49	0.12	0.19	0.79
	26	13.7	6.24	0.386	21.9	61.9	35.4	12.7	2.6	162	7.41	2.17	4.42	0.09	0.18	0.53
	27	12.8	6.29	0.375	20.4	59.5	34.3	13.1	1.7	105	7.34	1.70	4.39	0.15	0.27	0.81

Animal 19 - Initial haematology sample clotted; repeat sample collected

Appendix 13 (continued)
Haematology and Coagulation : Individual Values: Pretrial

Group	:	1	2	3
Test Item	:	Control	MenPF-1	MenPF-1
Dosage (µg/dose)	:	0	25	50

Group / sex	Animal No.	LUC ----------	Plat $x10^9/L$	PT s	APTT s	Fib mg/dL
1M	1	0.02	367	6.7	62.9	248
	2	0.01	357	6.3	56.8	211
	3	0.01	471	6.0	63.4	232
	19	0.01	502	6.3	62.1	233
	20	0.01	421	6.3	56.7	225
	21	0.01	333	6.1	62.5	206
2M	4	0.01	307	6.3	62.9	220
	5	-	474	6.1	58.5	222
	6	0.02	259	6.7	65.1	244
	22	0.01	461	6.1	55.5	245
	23	0.01	413	6.3	65.6	223
	24	0.01	500	6.7	67.1	193
3M	7	0.01	381	6.1	54.3	232
	8	0.01	487	6.0	50.6	265
	9	0.01	438	6.1	60.6	231
	25	0.02	474	5.9	60.8	209
	26	0.02	426	6.5	67.9	231
	27	0.02	460	6.3	59.2	210

Animal 19 - Initial haematology sample clotted; repeat sample collected
Animal 2 - Initial coagulation sample clotted; repeat sample collected
Animal 5 - Manual differential performed, LUC cancelled

Appendix 13 (continued)
Haematology and Coagulation : Individual Values: Pretrial

Group	:	1	2	3
Test Item	:	Control	MenPF-1	MenPF-1
Dosage (µg/dose)	:	0	25	50

Group / sex	Animal No.	Hb g/dL	RBC x10¹²/L	Hct L/L	MCH pg	MCV fL	MCHC g/dL	RDW %	Reti %	Ret x10⁹/L	WBC ----------	Neut ----------	Lymph ----------	Mono x10⁹/L	Eos ----------	Baso ----------
1F	10	12.2	5.84	0.357	20.8	61.1	34.1	12.4	2.5	148	5.03	0.77	3.53	0.04	0.13	0.54
	11	11.1	5.02	0.328	22.0	65.4	33.7	13.3	2.7	137	6.96	1.75	4.34	0.08	0.23	0.54
	12	12.5	6.00	0.368	20.9	61.2	34.1	12.3	2.0	118	8.14	3.12	4.19	0.09	0.11	0.62
	28	12.0	5.46	0.342	21.9	62.6	35.0	12.1	3.2	177	6.73	2.96	2.56	0.13	0.20	0.87
	29	12.2	5.91	0.365	20.6	61.8	33.4	12.0	2.2	128	5.45	2.13	3.11	0.00	0.00	0.22
	30	11.3	5.26	0.333	21.4	63.4	33.8	12.3	3.0	160	6.72	1.43	4.63	0.08	0.15	0.42
2F	13	12.2	5.96	0.353	20.5	59.1	34.7	13.5	3.0	180	8.65	2.82	4.94	0.10	0.12	0.64
	14	12.5	5.70	0.365	21.9	64.0	34.2	13.4	3.5	199	7.13	1.68	4.61	0.08	0.18	0.56
	15	12.3	5.87	0.354	21.0	60.3	34.8	12.4	3.3	193	6.15	1.54	3.91	0.06	0.13	0.49
	31	12.4	5.49	0.362	22.5	65.8	34.2	12.1	2.0	112	5.09	0.82	3.58	0.08	0.10	0.49
	32	12.5	6.01	0.362	20.7	60.2	34.4	13.4	2.9	172	6.99	1.92	4.16	0.07	0.22	0.61
	33	12.7	5.91	0.371	21.5	62.8	34.3	11.6	3.1	181	7.19	2.13	4.02	0.06	0.14	0.82
3F	16	12.6	5.64	0.358	22.4	63.4	35.2	11.5	2.5	141	6.46	1.62	4.09	0.06	0.17	0.52
	17	12.0	5.61	0.347	21.3	61.8	34.5	12.8	2.8	159	7.06	1.98	4.41	0.08	0.12	0.46
	18	13.7	6.34	0.392	21.7	61.9	35.0	12.4	1.9	119	5.49	2.54	2.20	0.06	0.14	0.55
	34	11.5	5.56	0.330	20.7	59.4	34.8	12.5	2.2	122	7.80	2.44	4.47	0.17	0.22	0.47
	35	11.8	5.69	0.357	20.8	62.8	33.1	12.8	3.0	172	7.29	1.31	4.95	0.07	0.15	0.80
	36	11.7	5.15	0.341	22.7	66.2	34.3	12.8	2.5	131	6.35	1.34	4.07	0.25	0.19	0.50

Appendix 13 (continued)
Haematology and Coagulation : Individual Values: Pretrial

Group	:	1	2	3
Test Item	:	Control	MenPF-1	MenPF-1
Dosage (µg/dose)	:	0	25	50

Group / sex	Animal No.	LUC ----------	Plat $x10^9/L$	PT s	APTT s	Fib mg/dL
1F	10	0.01	455	6.1	53.6	156
	11	0.01	395	6.3	59.7	162
	12	0.01	308	6.5	54.2	149
	28	-	526	5.8	53.9	186
	29	-	444	6.0	55.2	189
	30	0.01	457	6.5	56.1	165
2F	13	0.03	494	6.3	54.2	190
	14	0.02	565	-	-	-
	15	0.02	483	6.5	61.7	185
	31	0.01	466	6.6	54.6	150
	32	0.02	350	6.3	52.1	175
	33	0.02	417	6.3	55.0	153
3F	16	0.02	353	6.0	53.5	198
	17	0.01	567	6.5	51.1	157
	18	0.01	428	6.2	60.3	191
	34	0.03	448	6.0	42.8	184
	35	0.02	458	6.3	43.3	175
	36	0.00	467	6.4	57.1	168

Animals 28 and 29 - Manual differential performed, LUC cancelled
Animals 14, 15 and 31 - Initial coagulation sample clotted, repeat samples collected
Animal 14 - Repeat sample clotted

Appendix 14
Haematology and Coagulation: Individual Values: Day 66

Group	:	1	2	3
Test Item	:	Control	MenPF-1	MenPF-1
Dosage (µg/dose)	:	0	25	50

Group / sex	Animal No.	Hb g/dL	RBC x10¹²/L	Hct L/L	MCH pg	MCV fL	MCHC g/dL	RDW %	Reti %	Ret x10⁹/L	WBC ----------	Neut ----------	Lymph ----------	Mono x10⁹/L	Eos ----------	Baso ----------
1M	1	12.8	6.54	0.388	19.5	59.3	32.9	11.5	2.6	169	8.36	1.06	6.65	0.03	0.20	0.42
	2	12.8	6.41	0.396	19.9	61.9	32.2	11.9	2.0	131	9.41	1.23	7.39	0.03	0.19	0.56
	3	12.9	6.28	0.392	20.6	62.5	32.9	13.1	2.2	140	6.55	1.15	4.90	0.04	0.08	0.38
	19	13.3	6.31	0.397	21.0	62.9	33.4	12.0	2.8	179	8.61	2.34	5.32	0.08	0.18	0.68
	20	13.0	6.48	0.397	20.1	61.3	32.7	11.8	2.4	154	5.91	1.06	4.07	0.03	0.20	0.53
	21	13.8	6.48	0.410	21.3	63.2	33.7	11.4	3.1	201	5.47	0.88	3.77	0.06	0.23	0.52
2M	4	13.2	6.70	0.402	19.8	60.0	32.9	11.7	2.1	143	7.97	1.98	5.31	0.07	0.15	0.44
	5	13.8	6.59	0.408	20.9	61.8	33.9	11.9	3.3	218	5.20	1.66	2.96	0.16	0.16	0.26
	6	-	-	-	-	-	-	-	-	-	-	-	-	-	-	-
	22	13.5	6.18	0.401	21.9	64.9	33.7	13.0	3.4	208	5.14	1.17	3.02	0.29	0.14	0.52
	23	13.6	6.78	0.409	20.0	60.3	33.2	11.9	2.2	150	8.34	2.32	5.13	0.07	0.20	0.61
	24	12.2	5.83	0.372	20.8	63.8	32.6	12.5	2.6	152	6.57	1.56	4.48	0.05	0.12	0.36
3M	7	12.8	6.30	0.399	20.3	63.4	32.0	12.1	2.5	156	8.92	2.76	5.51	0.09	0.11	0.44
	8	12.3	6.31	0.382	19.4	60.6	32.1	11.4	2.2	137	5.87	1.58	3.69	0.07	0.13	0.38
	9	13.3	6.47	0.399	20.6	61.8	33.3	12.1	2.2	144	8.17	2.83	4.50	0.09	0.20	0.54
	25	12.6	6.08	0.383	20.7	63.0	32.9	12.4	2.8	167	9.40	2.38	6.07	0.10	0.19	0.64
	26	14.1	6.65	0.419	21.2	63.0	33.7	12.3	3.6	242	7.36	1.59	5.05	0.07	0.16	0.48
	27	13.4	6.80	0.412	19.7	60.5	32.6	12.3	1.8	125	10.12	3.41	5.36	0.13	0.33	0.87

Animals 6 and 22 - Initial haematology and coagulation samples clotted; repeat samples collected from Animal 22 only

Appendix 14 (continued)
Haematology and Coagulation: Individual Values: Day 66

Group	:	1	2	3
Test Item	:	Control	MenPF-1	MenPF-1
Dosage (µg/dose)	:	0	25	50

Group / sex	Animal No.	LUC ----------	Plat $x10^9/L$	PT s	APTT s	Fib mg/dL
1M	1	0.00	326	6.2	57.9	251
	2	0.01	373	6.4	51.3	141
	3	0.01	381	5.8	53.0	195
	19	0.01	384	6.3	57.1	195
	20	0.01	408	6.0	54.0	203
	21	0.01	312	5.8	63.8	207
2M	4	0.01	267	5.8	59.5	282
	5	-	376	5.7	55.9	316
	6	-	-	-	-	-
	22	0.01	534	5.8	60.5	249
	23	0.00	413	5.8	61.7	295
	24	0.00	479	5.8	56.4	251
3M	7	0.01	311	5.8	50.5	290
	8	0.00	357	5.8	47.6	361
	9	0.01	376	5.9	52.6	343
	25	0.01	447	5.8	52.7	315
	26	0.01	428	6.0	54.5	370
	27	0.02	371	6.0	52.9	330

Animals 6 and 22 - Initial haematology and coagulation samples clotted; repeat samples collected from Animal 22 only

Animal 21 - Initial coagulation sample clotted; repeat sample collected

Animal 5 - Manual differential performed, LUC cancelled

Appendix 14 (continued)
Haematology and Coagulation: Individual Values: Day 66

Group	:	1	2	3
Test Item	:	Control	MenPF-1	MenPF-1
Dosage (µg/dose)	:	0	25	50

Group / sex	Animal No.	Hb g/dL	RBC x10¹²/L	Hct L/L	MCH pg	MCV fL	MCHC g/dL	RDW %	Reti %	Ret x10⁹/L	WBC ----------	Neut ----------	Lymph ----------	Mono x10⁹/L	Eos ----------	Baso ----------
1F	10	12.6	6.21	0.381	20.2	61.3	33.0	12.4	2.9	182	4.90	0.71	3.63	0.03	0.13	0.40
	11	11.7	5.43	0.359	21.5	66.1	32.5	13.5	2.9	160	6.45	1.77	3.87	0.06	0.20	0.55
	12	12.7	6.22	0.396	20.4	63.7	32.1	12.5	2.7	171	6.58	2.37	3.42	0.00	0.13	0.66
	28	11.0	5.23	0.337	21.1	64.6	32.7	12.8	4.0	210	6.51	1.47	4.20	0.07	0.15	0.59
	29	13.1	6.59	0.405	19.9	61.5	32.3	12.3	2.5	167	16.66	1.50	13.83	0.16	0.29	0.85
	30	11.8	5.66	0.367	20.8	64.8	32.2	12.7	3.3	184	6.33	1.22	4.53	0.06	0.18	0.33
2F	13	12.9	6.31	0.381	20.5	60.4	33.9	12.3	3.5	220	7.50	1.76	4.73	0.12	0.18	0.70
	14	13.0	6.12	0.391	21.3	63.8	33.3	13.3	2.9	179	7.23	1.76	4.58	0.10	0.25	0.51
	15	12.0	6.10	0.366	19.7	60.0	32.9	12.8	3.1	187	5.97	1.30	4.03	0.05	0.10	0.49
	31	12.6	5.72	0.378	22.1	66.1	33.4	12.2	2.6	149	7.59	1.33	5.40	0.07	0.16	0.62
	32	13.2	6.60	0.403	20.0	61.0	32.9	12.3	2.8	187	6.08	1.58	3.73	0.06	0.18	0.51
	33	13.0	6.34	0.395	20.5	62.2	33.0	11.7	2.3	143	6.95	2.85	3.54	0.07	0.14	0.35
3F	16	12.2	5.83	0.361	20.9	61.9	33.7	11.0	2.9	170	5.99	0.98	4.36	0.06	0.10	0.49
	17	12.1	5.86	0.364	20.6	62.1	33.1	11.6	2.4	140	8.46	2.27	5.24	0.27	0.15	0.51
	18	12.5	6.00	0.383	20.9	63.8	32.7	12.8	2.9	174	4.72	1.32	2.75	0.06	0.11	0.47
	34	12.2	6.22	0.377	19.7	60.6	32.5	12.5	2.5	155	7.69	1.51	5.36	0.21	0.11	0.47
	35	12.3	6.17	0.384	20.0	62.2	32.1	13.4	5.3	328	7.04	2.01	4.08	0.08	0.19	0.67
	36	11.6	5.30	0.348	21.9	65.7	33.4	12.4	2.6	139	5.73	1.64	3.29	0.08	0.24	0.47

Animal 30 - Insufficient haematolgy sample to check analysis; repeat samples collected
Animals 35 and 36 - Haematology and coagulaton samples clotted; repeat samples taken

Appendix 14 (continued)
Haematology and Coagulation: Individual Values: Day 66

Group	:	1	2	3
Test Item	:	Control	MenPF-1	MenPF-1
Dosage (µg/dose)	:	0	25	50

Group / sex	Animal No.	LUC ----------	Plat $x10^9/L$	PT s	APTT s	Fib mg/dL
1F	10	0.01	500	6.3	55.2	123
	11	0.01	367	6.1	59.5	144
	12	-	315	6.3	57.3	116
	28	0.02	494	5.8	48.1	144
	29	0.04	342	6.0	54.1	141
	30	0.01	520	6.2	59.2	108
2F	13	0.02	375	5.6	61.5	267
	14	0.02	524	5.7	49.7	246
	15	0.00	486	6.1	59.3	187
	31	0.02	370	6.0	52.7	211
	32	0.01	279	6.0	62.2	174
	33	-	375	6.1	53.2	203
3F	16	0.01	319	5.7	52.5	302
	17	0.01	508	5.9	52.7	199
	18	0.01	470	5.7	58.8	179
	34	0.03	426	5.8	49.2	173
	35	0.01	538	5.8	69.5	231
	36	0.01	624	5.6	69.1	245

Animal 30 - Insufficient haematolgy sample to check analysis; repeat samples collected
Animals 35 and 36 - Haematology and coagulaton samples clotted; repeat samples taken
Animals 12 and 33 - Manual differential performed, LUC cancelled

Appendix 15
Haematology and Coagulation : Individual Values: Day 92

Group	:	1	2	3
Test Item	:	Control	MenPF-1	MenPF-1
Dosage (µg/dose)	:	0	25	50

Group / sex	Animal No.	Hb g/dL	RBC x10¹²/L	Hct L/L	MCH pg	MCV fL	MCHC g/dL	RDW %	Reti %	Ret x10⁹/L	WBC ----------	Neut ----------	Lymph ----------	Mono x10⁹/L	Eos ----------	Baso ----------
1M	19	13.6	6.24	0.401	21.7	64.2	33.8	12.3	3.1	194	7.86	1.59	5.45	0.05	0.19	0.57
	20	12.9	6.26	0.394	20.6	63.0	32.7	12.4	2.4	150	5.94	0.96	4.22	0.02	0.23	0.49
	21	14.2	6.69	0.428	21.2	64.0	33.2	11.0	2.5	166	6.13	0.90	4.52	0.03	0.21	0.47
2M	22	-	-	-	-	-	-	-	-	-	-	-	-	-	-	-
	23	14.3	6.95	0.431	20.6	61.9	33.3	12.0	2.3	157	7.06	1.75	4.44	0.05	0.19	0.62
	24	12.7	5.93	0.388	21.4	65.5	32.7	11.8	1.8	105	5.00	0.76	3.75	0.04	0.11	0.33
3M	25	12.9	6.09	0.390	21.1	64.0	33.0	11.8	2.1	128	6.64	0.92	4.97	0.04	0.15	0.54
	26	14.3	6.64	0.417	21.5	62.8	34.2	11.4	2.3	151	5.17	0.75	3.80	0.03	0.17	0.41
	27	13.1	6.46	0.401	20.3	62.0	32.7	13.3	2.3	148	7.63	1.61	5.10	0.05	0.25	0.60

Animal 22 - Insufficient haematology sample to check analysis

Appendix 15 (continued)
Haematology and Coagulation : Individual Values: Day 92

Group	:	1	2	3
Test Item	:	Control	MenPF-1	MenPF-1
Dosage (µg/dose)	:	0	25	50

Group / sex	Animal No.	LUC ----------	Plat $x10^9/L$	PT s	APTT s	Fib mg/dL
1M	19	0.02	341	6.4	61.1	174
	20	0.01	445	6.0	54.1	188
	21	0.01	277	5.8	55.6	167
2M	22	-	-	5.8	57.0	211
	23	0.01	351	5.8	59.5	176
	24	0.02	440	6.1	55.7	166
3M	25	0.01	427	5.8	54.5	194
	26	0.01	379	6.3	57.1	195
	27	0.02	387	6.0	57.8	150

Animal 22 - Insufficient haematology sample to check analysis

Appendix 15 (continued)
Haematology and Coagulation : Individual Values: Day 92

Group	:	1	2	3
Test Item	:	Control	MenPF-1	MenPF-1
Dosage (µg/dose)	:	0	25	50

Group / sex	Animal No.	Hb g/dL	RBC x10¹²/L	Hct L/L	MCH pg	MCV fL	MCHC g/dL	RDW %	Reti %	Ret x10⁹/L	WBC ----------	Neut ----------	Lymph ----------	Mono x10⁹/L	Eos ----------	Baso ----------
1F	28	12.4	5.82	0.376	21.4	64.6	33.1	11.5	2.4	138	6.08	2.00	3.41	0.03	0.15	0.47
	29	13.4	6.46	0.409	20.7	63.3	32.7	12.1	2.5	162	6.41	0.93	4.49	0.08	0.32	0.58
	30	12.1	5.65	0.370	21.4	65.4	32.6	11.3	2.3	130	4.85	0.84	3.50	0.05	0.14	0.32
2F	31	12.7	5.58	0.380	22.8	68.2	33.4	12.4	2.3	130	6.43	0.69	4.98	0.04	0.16	0.54
	32	13.8	6.67	0.421	20.6	63.1	32.7	12.5	2.4	159	6.48	1.36	4.38	0.05	0.19	0.50
	33	12.1	5.85	0.359	20.7	61.3	33.8	10.8	1.7	97	2.22	0.28	1.65	0.01	0.07	0.21
3F	34	12.3	6.00	0.375	20.5	62.5	32.9	12.6	2.0	119	5.77	1.11	4.01	0.09	0.15	0.39
	35	13.0	6.25	0.394	20.8	63.1	33.0	11.3	2.8	173	6.02	1.42	3.71	0.05	0.15	0.67
	36	12.0	5.35	0.363	22.4	68.0	33.0	12.0	2.1	113	4.28	1.03	2.73	0.04	0.13	0.34

Appendix 15 (continued)
Haematology and Coagulation : Individual Values: Day 92

Group	:	1	2	3
Test Item	:	Control	MenPF-1	MenPF-1
Dosage (µg/dose)	:	0	25	50

Group / sex	Animal No.	LUC ----------	Plat $x10^9/L$	PT s	APTT s	Fib mg/dL
1F	28	0.01	467	5.8	56.3	132
	29	0.01	375	5.9	55.4	152
	30	0.00	423	5.9	57.6	114
2F	31	0.02	436	5.8	51.4	133
	32	0.01	293	5.8	64.5	131
	33	0.00	11	-	-	-
3F	34	0.01	432	5.8	49.5	129
	35	0.02	445	5.7	57.2	181
	36	0.01	473	6.0	54.0	160

Animal 33 - Coagulation sample clotted

Appendix 16
Clinical Chemistry : Individual Values: Pretrial

	:			
Group	:	1	2	3
Test Item	:	Control	MenPF-1	MenPF-1
Dosage (µg/dose)	:	0	25	50

Group / sex	Animal No.	ALP U/L	ALT U/L	AST U/L	LDH U/L	CPK U/L	Urea mmol/L	Glu mmol/L	T.Bil µmol/L	Chol mmol/L	TP g/L	Alb g/L	Glob g/L	AG-R	Na mmol/L	K mmol/L
1M	1	220	25	12	82	737	7.6	7.79	<1.7	0.8	51	38	13	2.9	140	4.5
	2	139	39	10	56	365	6.8	7.65	<1.7	1.3	50	40	11	3.7	140	4.5
	3	105	20	8	59	399	7.2	8.20	<1.7	0.9	57	44	13	3.3	143	4.9
	19	200	30	13	62	795	7.7	8.48	<1.7	0.5	56	44	13	3.5	145	4.3
	20	139	18	11	60	838	6.5	7.56	<1.7	0.7	56	43	14	3.2	142	4.5
	21	178	23	13	100	665	6.5	8.39	<1.7	0.6	54	42	12	3.4	142	4.9
2M	4	166	22	12	75	437	8.0	8.39	<1.7	0.8	60	46	14	3.3	140	4.8
	5	141	27	11	55	442	7.1	8.13	<1.7	0.8	57	43	14	3.1	141	4.3
	6	172	43	12	65	557	7.3	8.08	<1.7	0.5	60	44	16	2.7	141	4.8
	22	142	28	12	47	322	6.6	8.18	<1.7	0.5	58	44	14	3.2	141	4.0
	23	148	27	9	59	500	7.8	7.88	<1.7	0.6	57	43	13	3.2	143	4.7
	24	151	28	11	59	717	7.7	8.24	<1.7	0.8	55	42	13	3.2	142	4.7
3M	7	188	22	12	50	826	7.4	8.33	<1.7	1.0	58	44	14	3.2	141	4.5
	8	163	31	13	93	770	7.3	7.62	<1.7	0.9	55	41	14	2.9	146	4.3
	9	148	29	11	65	436	7.1	7.73	<1.7	0.5	57	44	13	3.4	142	4.4
	25	193	16	9	49	548	7.3	8.40	<1.7	1.0	59	44	15	2.9	144	4.5
	26	234	32	12	62	563	6.4	7.78	<1.7	0.8	56	43	13	3.3	142	4.3
	27	224	19	11	52	442	7.3	7.99	<1.7	1.0	53	41	13	3.2	143	4.4

Appendix 16 (continued)
Clinical Chemistry : Individual Values: Pretrial

Group	:	1	2	3
Test Item	:	Control	MenPF-1	MenPF-1
Dosage (µg/dose)	:	0	25	50

Group / sex	Animal No.	Cl mmol/L	Phos mmol/L	Ca mmol/L	Crea µmol/L
1M	1	104	1.75	3.57	81
	2	104	2.02	3.51	69
	3	101	2.01	3.76	69
	19	105	1.70	3.68	67
	20	102	1.95	3.63	48
	21	105	1.73	3.64	52
2M	4	97	1.76	3.82	52
	5	101	1.94	3.70	51
	6	100	2.13	3.73	70
	22	99	1.89	3.74	54
	23	103	1.71	3.73	58
	24	102	1.78	3.79	75
3M	7	101	1.97	3.76	62
	8	105	1.63	3.60	67
	9	99	1.90	3.75	65
	25	99	2.14	3.78	64
	26	102	1.95	3.68	66
	27	103	2.07	3.57	58

Appendix 16 (continued)
Clinical Chemistry : Individual Values: Pretrial

Group	:	1	2	3
Test Item	:	Control	MenPF-1	MenPF-1
Dosage (µg/dose)	:	0	25	50

Group / sex	Animal No.	ALP U/L	ALT U/L	AST U/L	LDH U/L	CPK U/L	Urea mmol/L	Glu mmol/L	T.Bil µmol/L	Chol mmol/L	TP g/L	Alb g/L	Glob g/L	AG-R	Na mmol/L	K mmol/L
1F	10	153	13	13	71	2407	7.9	8.78	<1.7	1.4	55	42	13	3.2	141	4.6
	11	325	18	11	64	465	6.4	7.29	<1.7	1.1	52	40	12	3.4	142	4.3
	12	250	30	12	69	768	6.2	7.75	<1.7	1.1	55	40	15	2.6	143	4.2
	28	208	22	10	73	531	8.3	7.53	<1.7	1.8	58	44	15	2.9	141	4.4
	29	275	28	8	75	868	5.4	7.67	<1.7	1.4	57	43	14	3.1	140	4.5
	30	178	27	11	67	726	7.6	7.23	<1.7	1.0	55	42	13	3.3	143	4.3
2F	13	181	36	12	52	295	6.7	8.68	<1.7	1.1	61	46	15	3.0	141	4.2
	14	173	22	11	64	421	7.6	8.42	<1.7	1.8	55	42	13	3.3	141	4.7
	15	202	41	13	84	671	6.6	8.61	<1.7	1.2	52	39	12	3.2	140	4.0
	31	261	23	8	60	741	6.6	8.42	<1.7	1.3	52	40	13	3.0	140	4.2
	32	238	32	11	94	774	6.0	9.30	<1.7	1.2	51	39	12	3.3	142	4.0
	33	260	17	13	57	428	6.4	7.92	<1.7	1.1	55	43	12	3.5	141	4.1
3F	16	177	29	13	64	581	6.5	8.03	<1.7	1.3	59	44	14	3.1	140	4.2
	17	170	43	12	54	540	8.3	9.05	<1.7	2.1	61	46	14	3.2	140	4.3
	18	177	32	10	61	1231	5.8	9.12	<1.7	1.7	58	45	13	3.4	141	4.1
	34	156	19	13	55	1363	6.5	7.88	<1.7	2.1	62	46	16	3.0	139	4.1
	35	214	19	15	65	1086	6.8	9.14	<1.7	1.1	56	45	12	3.8	142	4.1
	36	149	30	10	49	475	8.4	8.59	<1.7	1.2	59	45	13	3.4	141	4.8

Appendix 16 (continued)
Clinical Chemistry : Individual Values: Pretrial

	:			
Group	:	1	2	3
Test Item	:	Control	MenPF-1	MenPF-1
Dosage (µg/dose)	:	0	25	50

Group / sex	Animal No.	Cl mmol/L	Phos mmol/L	Ca mmol/L	Crea µmol/L
1F	10	99	2.42	3.69	59
	11	106	1.78	3.62	54
	12	103	1.71	3.67	60
	28	98	2.14	3.72	68
	29	105	1.65	3.67	53
	30	103	1.99	3.59	64
2F	13	95	2.10	3.87	61
	14	102	2.15	3.59	64
	15	99	1.96	3.69	62
	31	103	1.86	3.46	65
	32	103	2.02	3.49	62
	33	99	2.13	3.54	62
3F	16	98	1.89	3.74	59
	17	103	1.57	3.70	47
	18	99	1.98	3.63	57
	34	97	2.04	3.78	70
	35	98	2.36	3.73	68
	36	102	2.09	3.75	54

Appendix 17
Clinical Chemistry : Individual Values: Day 66

Group	:	1	2	3
Test Item	:	Control	MenPF-1	MenPF-1
Dosage (µg/dose)	:	0	25	50

Group / sex	Animal No.	ALP U/L	ALT U/L	AST U/L	LDH U/L	CPK U/L	Urea mmol/L	Glu mmol/L	T.Bil µmol/L	Chol mmol/L	TP g/L	Alb g/L	Glob g/L	AG-R	Na mmol/L	K mmol/L
1M	1	70	37	16	67	793	8.7	8.48	<1.7	0.4	54	43	11	3.8	144	4.2
	2	51	70	12	57	389	7.5	7.34	<1.7	0.6	53	44	10	4.4	145	4.7
	3	54	33	11	54	442	7.4	7.79	<1.7	0.5	60	48	12	3.8	146	4.7
	19	80	34	11	52	671	8.6	7.19	<1.7	0.3	57	45	12	3.6	143	4.3
	20	56	20	11	46	650	6.6	6.83	<1.7	0.4	58	46	12	3.8	144	4.8
	21	62	29	21	60	715	5.9	7.46	<1.7	0.4	58	45	12	3.7	143	4.7
2M	4	71	24	14	45	554	6.8	7.10	<1.7	0.9	62	45	16	2.7	142	4.5
	5	72	42	9	51	367	7.0	7.02	<1.7	0.7	62	48	14	3.4	143	4.3
	6	59	59	19	82	581	9.2	7.03	<1.7	0.5	63	46	17	2.6	143	4.6
	22	62	31	13	81	482	6.5	7.79	<1.7	0.6	61	47	14	3.5	144	4.6
	23	49	42	9	46	373	7.7	7.22	<1.7	0.4	60	42	17	2.4	147	4.9
	24	56	34	13	46	810	8.5	7.42	<1.7	0.4	60	46	14	3.2	146	4.8
3M	7	65	36	9	59	636	6.7	7.90	<1.7	0.4	62	47	15	3.1	145	4.5
	8	60	36	14	197	1925	6.1	7.60	<1.7	0.4	60	45	15	3.0	146	4.1
	9	55	38	17	79	576	6.9	6.85	<1.7	0.4	62	47	15	3.2	146	4.1
	25	63	26	11	46	529	7.5	7.13	<1.7	0.7	61	45	16	2.8	146	4.1
	26	87	86	19	58	412	6.9	8.03	<1.7	0.4	62	46	16	2.9	145	4.5
	27	95	27	15	66	484	6.5	7.50	<1.7	0.5	57	43	14	3.0	145	4.3

Appendix 17 (continued)
Clinical Chemistry : Individual Values: Day 66

Group	:	1	2	3
Test Item	:	Control	MenPF-1	MenPF-1
Dosage (µg/dose)	:	0	25	50

Group / sex	Animal No.	Cl mmol/L	Phos mmol/L	Ca mmol/L	Crea µmol/L
1M	1	107	1.20	3.53	91
	2	106	1.44	3.55	70
	3	105	1.30	3.78	67
	19	108	1.18	3.58	63
	20	108	1.21	3.60	52
	21	106	1.29	3.58	58
2M	4	103	1.27	3.68	51
	5	105	1.28	3.64	50
	6	104	1.35	3.73	63
	22	105	1.54	3.67	50
	23	104	1.43	3.73	69
	24	108	1.34	3.74	90
3M	7	103	1.46	3.79	64
	8	106	1.48	3.62	69
	9	103	1.35	3.71	63
	25	107	1.22	3.58	66
	26	103	1.39	3.70	80
	27	106	1.24	3.51	64

Appendix 17 (continued)
Clinical Chemistry : Individual Values: Day 66

Group	:	1	2	3
Test Item	:	Control	MenPF-1	MenPF-1
Dosage (µg/dose)	:	0	25	50

Group / sex	Animal No.	ALP U/L	ALT U/L	AST U/L	LDH U/L	CPK U/L	Urea mmol/L	Glu mmol/L	T.Bil µmol/L	Chol mmol/L	TP g/L	Alb g/L	Glob g/L	AG-R	Na mmol/L	K mmol/L
1F	10	89	19	8	49	639	6.2	6.57	<1.7	1.1	57	46	11	4.4	145	4.2
	11	109	22	8	37	341	6.9	6.79	<1.7	1.2	58	46	13	3.7	143	4.4
	12	64	36	9	42	1056	8.0	6.99	<1.7	1.3	58	45	13	3.4	142	5.3
	28	96	26	10	70	553	9.2	6.82	<1.7	1.5	58	45	13	3.4	146	4.1
	29	198	75	13	47	1315	5.9	7.82	<1.7	1.3	58	45	13	3.5	143	4.4
	30	69	32	9	36	679	10.6	7.54	<1.7	1.3	60	47	13	3.5	143	4.5
2F	13	59	46	13	47	377	6.9	8.01	1.7	1.3	62	47	15	3.1	144	4.3
	14	60	29	12	73	582	8.1	7.02	<1.7	1.3	59	45	15	3.1	145	3.8
	15	97	47	12	50	815	8.3	7.47	<1.7	1.3	58	45	12	3.7	146	4.4
	31	81	69	17	87	1088	9.3	7.71	<1.7	1.2	54	41	13	3.1	145	4.2
	32	71	52	12	47	615	7.1	7.95	<1.7	1.3	57	45	12	3.7	144	4.3
	33	89	29	16	54	431	8.2	7.30	<1.7	0.7	60	45	15	3.1	145	3.9
3F	16	83	114	32	54	711	8.3	6.93	<1.7	1.2	60	43	17	2.6	144	4.8
	17	80	47	14	60	610	7.3	7.85	<1.7	2.5	63	47	16	2.9	142	4.3
	18	108	36	12	43	781	6.5	6.99	<1.7	2.0	60	46	14	3.2	144	3.9
	34	91	46	14	36	552	8.5	7.12	2.2	2.9	64	48	16	3.0	143	4.6
	35	87	27	14	31	737	5.9	7.74	<1.7	1.0	62	47	15	3.2	147	4.0
	36	57	82	13	41	348	9.1	7.85	1.7	1.2	61	45	15	3.0	143	4.4

Appendix 17 (continued)
Clinical Chemistry : Individual Values: Day 66

Group	:	1	2	3
Test Item	:	Control	MenPF-1	MenPF-1
Dosage (µg/dose)	:	0	25	50

Group / sex	Animal No.	Cl mmol/L	Phos mmol/L	Ca mmol/L	Crea µmol/L
1F	10	101	1.60	3.69	66
	11	107	1.33	3.64	60
	12	107	1.18	3.78	74
	28	103	1.42	3.67	80
	29	104	1.55	3.68	80
	30	103	1.53	3.72	85
2F	13	100	1.59	3.82	79
	14	101	1.48	3.51	81
	15	101	1.41	3.89	75
	31	104	1.95	3.46	114
	32	105	1.40	3.57	69
	33	104	1.49	3.49	76
3F	16	105	1.29	3.68	83
	17	101	1.35	3.67	73
	18	102	1.73	3.57	64
	34	103	1.31	3.76	85
	35	107	1.49	3.55	77
	36	102	1.54	3.62	72

Appendix 18

Clinical Chemistry : Individual Values: Day 92

Group	:	1	2	3
Test Item	:	Control	MenPF-1	MenPF-1
Dosage (µg/dose)	:	0	25	50

Group / sex	Animal No.	ALP U/L	ALT U/L	AST U/L	LDH U/L	CPK U/L	Urea mmol/L	Glu mmol/L	T.Bil µmol/L	Chol mmol/L	TP g/L	Alb g/L	Glob g/L	AG-R	Na mmol/L	K mmol/L
1M	19	64	38	14	70	588	7.5	6.16	<1.7	0.3	56	44	12	3.7	147	3.5
	20	49	14	10	62	517	7.4	6.01	<1.7	0.4	57	45	13	3.5	144	4.3
	21	53	26	18	61	413	6.1	7.69	<1.7	0.3	55	44	11	4.1	145	4.3
2M	22	57	32	13	56	423	6.9	6.58	<1.7	0.6	61	49	13	3.8	144	4.6
	23	49	42	12	66	429	8.0	6.55	<1.7	0.4	58	47	12	4.0	145	4.6
	24	45	42	17	96	591	9.7	6.62	<1.7	0.5	57	46	11	4.1	147	4.6
3M	25	59	18	8	63	465	7.3	6.52	<1.7	0.7	58	46	12	3.7	143	4.4
	26	67	50	21	83	476	5.9	7.33	<1.7	0.3	58	45	13	3.4	143	4.2
	27	181	27	17	70	382	6.6	7.10	<1.7	0.4	56	45	12	3.9	145	4.3

Appendix 18 (continued)
Clinical Chemistry : Individual Values: Day 92

Group	:	1	2	3
Test Item	:	Control	MenPF-1	MenPF-1
Dosage (µg/dose)	:	0	25	50

Group / sex	Animal No.	Cl mmol/L	Phos mmol/L	Ca mmol/L	Crea µmol/L
1M	19	106	1.19	3.51	76
	20	105	1.05	3.74	62
	21	105	1.33	3.54	63
2M	22	105	1.03	3.95	52
	23	103	1.25	3.81	73
	24	106	1.10	3.87	100
3M	25	101	1.23	3.64	71
	26	103	1.21	3.56	78
	27	104	1.26	3.61	73

Appendix 18 (continued)
Clinical Chemistry : Individual Values: Day 92

Group	:	1	2	3
Test Item	:	Control	MenPF-1	MenPF-1
Dosage (µg/dose)	:	0	25	50

Group / sex	Animal No.	ALP U/L	ALT U/L	AST U/L	LDH U/L	CPK U/L	Urea mmol/L	Glu mmol/L	T.Bil µmol/L	Chol mmol/L	TP g/L	Alb g/L	Glob g/L	AG-R	Na mmol/L	K mmol/L
1F	28	84	30	11	51	387	9.9	5.96	<1.7	1.3	61	49	12	4.2	145	4.3
	29	99	38	13	79	757	8.0	6.24	<1.7	1.3	61	49	12	4.1	143	4.4
	30	63	31	10	43	508	9.8	6.77	<1.7	1.2	59	46	13	3.6	144	4.4
2F	31	63	36	14	35	446	10.0	7.53	<1.7	1.1	53	42	12	3.6	144	4.4
	32	58	50	18	40	423	9.1	7.27	<1.7	1.1	56	44	13	3.5	143	4.3
	33	65	31	19	161	610	8.0	6.97	<1.7	0.8	55	44	11	3.9	141	4.3
3F	34	91	24	14	34	345	8.5	6.34	1.7	2.8	63	49	14	3.4	143	4.2
	35	109	29	15	44	521	7.9	6.48	<1.7	1.0	61	47	14	3.5	144	4.1
	36	60	42	14	47	313	9.8	6.71	<1.7	0.9	61	46	15	3.0	142	4.4

Appendix 18 (continued)
Clinical Chemistry : Individual Values: Day 92

Group	:	1	2	3
Test Item	:	Control	MenPF-1	MenPF-1
Dosage (µg/dose)	:	0	25	50

Group / sex	Animal No.	Cl mmol/L	Phos mmol/L	Ca mmol/L	Crea µmol/L
1F	28	102	1.24	3.83	87
	29	107	0.99	3.77	73
	30	103	1.26	3.75	90
2F	31	106	1.36	3.49	125
	32	106	1.18	3.51	84
	33	101	1.50	3.47	90
3F	34	103	1.05	3.82	94
	35	104	1.34	3.75	79
	36	105	1.15	3.54	69

Appendix 19
Antibody Analysis

1 INTRODUCTION

MenPF-1 is a developmental vaccine against disease caused by Neisseria meningitidis (the meningococcus). The major antigens in this vaccine are the outer membrane proteins PorA and FetA. The vaccine contains outer membrane vesicles (OMVs) from a meningococcal strain genetically modified to over-express the FetA antigen.

MenPF-1 OMVs, adsorbed to aluminium hydroxide ($Al(OH)_3$) adjuvant, have been produced by the Norwegian Institute of Public Health (NIPH). A validation batch (Lot number FMOX1102) of the MenPF-1 vaccine was tested for in vivo toxicity in rabbits. The results of this study will be used to support an application by the University of Oxford for the use of MenPF-1 in a Phase 1 clinical trial in humans.

During the toxicology study, New Zealand White rabbits were given four doses of $Al(OH)_3$-only control inoculum or $Al(OH)_3$-adjuvanted MenPF-1 OMVs. Rabbits receiving MenPF-1 OMVs were given either a single human dose (25µg total protein) or double human dose (50µg total protein). Doses were given on days 1, 22, 43 and 64. Blood samples were collected from each rabbit pre-immunisation, before dosing on days 22, 64 and on day 92. Blood samples were processed, and extracted serum samples stored at -80°C.

The immunological testing of serum samples was performed by the National Institute of Biological Standards and Control (NIBSC). An in vitro Enzyme Linked Immunosorbent Assay (ELISA) was used at NIBSC to determine seroconversion of rabbits in the study. Seroconversion is defined as the development of detectable specific antibodies raised against the vaccine in response to immunisation. The ELISA was used to demonstrate seroconversion in the rabbits which should switch from MenPF-1 seronegative to MenPF-1 seropositive if successfully immunised. The binding of antibodies in pre- and post-vaccination sera to MenPF-1 OMVs was assessed using a validated assay of suitable sensitivity and specificity.

Appendix 19 (continued)
Antibody Analysis

2 PROCEDURE – ENZYME LINKED IMMUNOSORBENT ASASY

All details of the test, including samples tested, dilutions made, critical timings, pipette serial numbers, and buffers and reagents used on the MenPF-1 Rabbit ELISA test were recorded on the test record form (Document S/N 6116).

a). A 2μg/ml solution of MenPF-1 OMV in coating buffer was prepared according to the following table:

Number of Plates	Total Solution Volume (ml)	Volume Coating Buffer (ml)	Volume OMV stock (μl)
1	12	11.947	53
2	24	23.893	107
3	34	33.849	151
4	45	44.800	200
5	55	54.756	244
6	65	64.711	289

The appropriate wells of microtitre plates were coated with 100μl of solution, covered and incubated at +4°C for a minimum of 16 hours in a sealed container which was labelled to be identifiable to the test operator.

b). The ELISA plates were washed with Wash Buffer using the Skatran Plate washer. If the machine was switched off or the connected buffer had been changed from that required by this assay, a blank plate of pure water was first used to rinse the machine; the machine was then primed with the required Wash Buffer. All buffer changes were recorded on the test record form.

c). Plates were blocked with 100μl per well of Dilution Buffer, covered and incubated for a minimum of 1 hour (+10 minutes) at room temperature in a sealed container.

d). The ELISA plates were washed as in step b).

e). Dilutions of the sera to be tested, and the positive control, were prepared by dilution of the Buffer as follows:
- Positive control sera, diluted 1:500 (1:10 followed by 1:50);
- Negative control sera, diluted 1:100 (1:10 followed by 1:10);
- Test sera taken on day 0 (Test Sample 1), diluted 1:100 (1:10 followed by 1:10);
- Test sera taken on day 22 (Test Sample 2), diluted 1:300 (1:10 followed by 1:30);

Appendix 19 (continued)
Antibody Analysis

- Test sera taken on day 64 (Test Sample 3), diluted 1:900 (1:10 followed by 1:90);
- Test sera taken on day 92 (Test Sample 4), diluted 1:900 (1:10 followed by 1:90).

f). ELISA plates were then prepared. One 96-well plate was required to test all serum samples extracted from each rabbit, including the positive control serum, at a maximum of 8 dilutions for each serum sample. All samples were tested in duplicate columns (see example plate layout in Figure 1). Samples were assigned randomly to columns (see Appendix 1). For the standard assay, random plate layouts were generated and can be found on the MenPF-1 Rabbit ELISA test record form (retained in the study data). For rabbits for which less than four serum samples were available, columns listed as "Test Sample 4" were left Blank. The template used in the assay on the MenPF-1 Rabbit ELISA was noted on the test record form (retained in the study data). A different template for each assay was used in rotation in the order 1 through to 8.

Figure 1: Example plate layout (all samples and controls are randomised across the plate). See Appendix 1 for detailed plate layout templates.

	TS2	+ve	-ve	-ve	TS1	TS3	TS4	+ve	TS2	TS4	TS3	TS1
	1	2	3	4	5	6	7	8	9	10	11	12
A	1/1*	1/1*	1/1*	1/1*	1/1*	1/1*	1/1*	1/1*	1/1*	1/1*	1/1*	1/1*
B	1/3	1/3	1/3	1/3	1/3	1/3	1/3	1/3	1/3	1/3	1/3	1/3
C	1/9	1/9	1/9	1/9	1/9	1/9	1/9	1/9	1/9	1/9	1/9	1/9
D	1/27	1/27	1/27	1/27	1/27	1/27	1/27	1/27	1/27	1/27	1/27	1/27
E	etc.	etc.	etc.	etc.	etc.	etc.	etc.	etc.	etc.	etc.	etc.	etc.
F												
G												
H												

*Pre-diluted test sample or positive control.
TS = Test serum
+ve = Positive control serum
-ve = Negative control serum

h). Wells in rows B - H were filled with 100µl of Dilution Buffer; row A was left empty.

Appendix 19 (continued)
Antibody Analysis

i). 150µl of each diluted preparation was added to the wells in row A. 50µl of each sample was then removed and transferred to the appropriate wells in row B. Wells were mixed for a maximum of 5 times. 50µl volumes were then transferred to the next row (C). This procedure was repeated down the plate. Following mixing of row H, 50µl of the sample was discarded from each well. Each well in rows A through to H now contained 100µl volumes. Plates were then covered and incubated at room temperature in the sealed container for a minimum of 1 hours (+10 minutes).

j). ELISA plates were washed as in step b).

k). Goat anti-rabbit HRP conjugate was diluted 1:2000 in Dilution Buffer and 100µl added to all wells, covered and incubated for a minimum of 1 hour (+10 minutes) in the sealed container at room temperature.

l). ELISA plates were washed as in step b).

m). 100µl TMBlue substrate was then added to all wells and incubated at room temperature for up to ten minutes. Following colour development, 100µl 1M sulphuric acid was added to all the wells to stop the reaction. The plates were then read at 450 nm using a microplate reader

n). Raw data was printed immediately, signed and dated.

3 10. DATA ANALYSIS, VALIDITY AND DETERMINATION OF SEROCONVERSION

Absorbance levels across the dilution series from each test sample were used to directly compare the levels of IgG binding following immunisation of each rabbit to pre-trial sera, in order to determine whether each rabbit was seroconverted. All calculations were recorded on the print-out of the raw data and reviewed by the Responsible Scientist. No computer software was required for data analysis.

10.1. DATA ANALYSIS

a) Referring to the dilution series listed below, for each test sample, the highest dilution factor at which the absorbance at 450nm was higher than 0.70 was determined (where at least two consecutive dilutions were higher than the threshold, except where only a 1/100 dilution of a sample had absorbance higher than 0.70). The dilution factor was recorded as "IG". If a sample did not result in absorbance higher than 0.70 at a dilution of 1:100, IG was recorded as 100. If higher or lower dilutions (to a minimum of 1/100) were required to determine IG, the test sample was repeated with appropriate dilutions.

Appendix 19 (continued)
Antibody Analysis

For duplicates of a single sample, if IG values were one dilution apart, a mean value was taken as the IG for that sample. If IG values for duplicates of a single sample were greater than one dilution apart, that sample was repeated.

	Row	Positive	Test Sample 1 /Negative	Test Sample 2	Test Sample 3/4
Start dilution →	A	500	100	300	900
Dilution series ↓ (1:3)	B	1500	300	900	2700
	C	4500	900	2700	8100
	D	13500	2700	8100	24300
	E	40500	8100	24300	72900
	F	121500	24300	72900	218700
	G	364500	72900	218700	656100
	H	1093500	218700	656100	1968300

b) For each test sample 2, 3 and 4, the increase in binding following vaccination was calculated as follows:

$$\Delta IG = IG_{(\text{Test Sample n})} / IG_{(\text{Test Sample 1})}$$

Where "n" = 2, 3 or 4.

For the positive control serum, ΔIG was calculated as follows:

$$\Delta IG = IG_{(\text{Positive control serum})} / IG_{(\text{Negative control serum})}$$

c) Values for ΔIG were recorded on the test record form.

4 10.2. VALIDITY REQUIREMENTS

In order for the test to be valid:

i). The maximum absorbance at 450nm for the positive control serum must be greater than 3.0 for both repeats.
ii). The minimum absorbance at 450nm for the positive control serum must be less than 0.7 for both repeats.
iii). The maximum absorbance at 450nm for the negative control serum must be greater than 0.7.
iv). The ΔIG value calculated for the positive control serum must be between 90 and 810.

Validity of the assay was recorded on the test record form.

Appendix 19 (continued)
Antibody Analysis

Test Facility Study No. 520419

A test was repeated if it did not meet the validity requirements, if IG values were greater than one dilution apart for duplicates of any test sample, or if alternative dilutions were required to determine IG values for any test sample.

10.3. DETERMINATION OF SEROCONVERSION

When analysis of serum samples from all animals was completed, seroconversion was determined for each time point after initiation of the trial (Day 22, Day 64 and Day 92). For each post-vaccination serum sample, when IG $\geq$ 4 seroconverion was determined to have occurred.

5 11. RECORDING OF RESULTS

Copies of all raw and analysed data, as well as scanned copies of all test record forms, were stored in the bact\MenPFtox drive. Hard copies of all test record forms and raw data were stored in B38. On completion of analysis of all serum samples, all printed and electronic data were sent to Charles River Laboratories for review and incorporation into the toxicology report.

Appendix 19 (continued)
Antibody Analysis

Test Facility Study No. 520419

6 TABLES

Table 1 Geometric **Mean ΔIG** Values: Males – Main Study

Group	Treatment (µg/dose of MenPF-1)	Day of Antibody Bleed			
		Pre-trial	22	64	92
1	0	-	1.44	1	-
2	25	-	81	1516	-
3	50	-	81	7479	-

\- = Not applicable

Appendix 19 (continued)
Antibody Analysis

Test Facility Study No. 520419

Table 2 Geometric **Mean ΔIG** Values : Females – Main Study

Group	Treatment (µg/dose of MenPF-1)	Day of Antibody Bleed			
		Pre-trial	22	64	92
1	0	-	1	2.08	-
2	25	-	71	441	-
3	50	-	102	5186	-

- = Not applicable

Appendix 19 (continued)
Antibody Analysis

Test Facility Study No. 520419

Table 3 Geometric **Mean ΔIG** Values : Males – Recovery Study

Group	Treatment (µg/dose of MenPF-1)	Day of Antibody Bleed			
		Pre-trial	22	64	92
1	0	-	1.65	1.65	1.65
2	25	-	27	1669	1199
3	50	-	505	4880	2854

- = Not applicable

Appendix 19 (continued)
Antibody Analysis

Test Facility Study No. 520419

Table 4 Geometric **Mean ΔIG** Values : Females – Recovery Study

Group	Treatment (µg/dose of MenPF-1)	Day of Antibody Bleed			
		Pre-trial	22	64	92
1	0	-	1	3	3
2	25	-	24	951	1669
3	50	-	56	2407	1325

- = Not applicable

Appendix 19 (continued)
Antibody Analysis

7 APPENDICES

Appendix 1 ELISA PLATE LAYOUT TEMPLATES

Random plate layouts for Anti-MenPF-1 Rabbit Immunoglobulin ELISA

Test sample, reference, and positive control added to row A of 96 well plates as indicated below:

Key:

+: Positive serum
-: Negative Serum
TS: Test sample

	1	2	3	4	5	6	7	8	9	10	11	12
**Template 1**	TS2	+	-	-	TS1	TS3	TS4	+	TS2	TS4	TS3	TS1
**Template 2**	-	TS3	TS4	+	-	TS3	TS1	TS4	+	TS1	TS2	TS2
**Template 3**	TS3	TS2	TS1	-	TS4	TS2	TS1	TS3	+	TS4	-	+
**Template 4**	TS1	TS3	-	TS4	+	TS2	+	TS1	TS2	TS4	-	TS3
**Template 5**	-	TS1	TS4	TS2	-	+	TS3	TS2	TS4	TS1	TS3	+
**Template 6**	-	TS2	TS4	TS2	TS1	TS3	+	TS1	TS3	-	TS4	+
**Template 7**	TS4	+	TS4	-	TS2	TS2	+	TS3	-	TS1	TS3	TS1
**Template 8**	TS3	TS3	TS4	TS1	+	TS1	TS2	+	TS4	-	-	TS2

Appendix 19 (continued)
Antibody Analysis

Test Facility Study No. 520419

Appendix 2 Individual ΔIG Values : Males – Main Study

Group	Animal Number	Treatment (µg/dose of MenPF-1)	Day of Antibody Bleed			
			Pre-trial	22	64	92
1	1	0	-	1	1	-
	2		-	1	1	-
	3		-	3	1	-
2	4	25	-	243	2187	-
	5		-	27	729	-
	6		-	81	2187	-
3	7	50	-	27	1458	-
	8		-	243	13122	-
	9		-	81	21870	-

- = Not applicable

Appendix 19 (continued)
Antibody Analysis

Test Facility Study No. 520419

Appendix 3 Individual ΔIG Values : Females – Main Study

Group	Animal Number	Treatment (μg/dose of MenPF-1)	Day of Antibody Bleed			
			Pre-trial	22	64	92
1	10	0	-	1	1	-
	11		-	1	3	-
	12		-	1	3	-
2	13	25	-	9	9	-
	14		-	27	729	-
	15		-	1458	13122	-
3	16	50	-	81	13122	-
	17		-	162	810	-
	18		-	81	13122	-

- = Not applicable

Appendix 19 (continued)
Antibody Analysis

Test Facility Study No. 520419

Appendix 4 Individual ΔIG Values : Males – Recovery Study

Group	Animal Number	Treatment (µg/dose of MenPF-1)	Day of Antibody Bleed			
			Pre-trial	22	64	92
1	19	0	-	4.5	4.5	4.5
	20		-	1	1	1
	21		-	1	1	1
2	22	25	-	27	2187	2187
	23		-	9	162	486
	24		-	81	13122	1620
3	25	50	-	1093.5	2187	2187
	26		-	486	7290	4374
	27		-	243	7290	2430

\- = Not applicable

Appendix 19 (continued)
Antibody Analysis

Test Facility Study No. 520419

Appendix 5 Individual ΔIG Values : Females – Recovery Study

Group	Animal Number	Treatment (µg/dose of MenPF-1)	Day of Antibody Bleed			
			Pre-trial	22	64	92
1	28	0	-	1	1	1
	29		-	1	27	27
	30		-	1	1	1
2	31	25	-	6	1458	729
	32		-	27	2430	4374
	33		-	81	243	1458
3	34	50	-	27	486	486
	35		-	243	13122	729
	36		-	27	2187	6561

\- = Not applicable

Appendix 20
Individual Necropsy and Histological Findings: Day 66

Abbreviations Used

ADR	=	Adrenal Gland	OVA	=	Ovary
AOR	=	Aorta	OVD	=	Oviduct
APP	=	Appendix	PAR	=	Parathyroid Gland
BRA	=	Brain	PCEN	=	Pancreas (Endocrine)
CAE	=	Caecum	PCEX	=	Pancreas (Exocrine)
CER	=	Cervix	PIT	=	Pituitary Gland
COL	=	Colon	PRO	=	Prostate
DUO	=	Duodenum	REC	=	Rectum
EPI	=	Epididymis	sac	=	Sacculus Rutundus
EYE	=	Eye	SCI	=	Sciatic Nerve
FEM	=	Femur	SEM	=	Seminal Vesicle
GALT	=	Gut Associated Lymphoid Tissue	SGSM	=	Salivary gland (Submaxillary)
GBL	=	Gallbladder	SKI	=	Skin and Subcutis
HEA	=	Heart	SKM	=	Skeletal Muscle
ILE	=	Ileum	SPL	=	Spleen
JEJ	=	Jejunum	SPN	=	Spinal Cord
KID	=	Kidney	STO	=	Stomach
LAC	=	Lacrimal Gland	STR	=	Sternum
LIV	=	Liver	TES	=	Testis
LUN	=	Lung	THM	=	Thymus
LNIN	=	Inguinal Lymph Node	THR	=	Thyroid Gland
LULU	=	Lumbar Lymph Node	TOG	=	Tongue
LNMA	=	Mandibular Lymph Nodes	TRA	=	Trachea
LNMS	=	Mesenteric Lymph Nodes	URE	=	Ureter
MAM	=	Mammary Gland	URB	=	Urinary Bladder
OES	=	Oesophagus	UTE	=	Uterus
OPT	=	Optic Nerve	VAG	=	Vagina

Appendix 20 (continued)
Individual Necropsy and Histological Findings: Day 66

PROJECT NUMBER:	520419
TREATMENT:	Group 1 (0 ug/dose) MALES

ANIMAL NO:	FINDINGS:
1	Terminal Kill<br>Day of Necropsy: 66
NECROPSY FINDINGS:	
GENERAL COMMENTS :	Tissues not listed below were normal
LUNG :	Discolouration, all lobes, (mottled)
TESTIS :	Small, right
HISTOLOGICAL FINDINGS:	
EPIDIDYMIS :	Aspermia, unilateral
INJECTION SITE 1 :	Macrophage accumulation, intramuscular, moderate, (with cytoplasmic foreign material and multinucleated giant cells)
KIDNEY :	Basophilic tubules, multifocal, minimal
LUNG :	Agonal congestion/haemorrhage, (relates to necropsy finding)
LYMPH NODE (LUMBAR) :	Only one examined
PITUITARY GLAND :	Only anterior lobe examined
TESTIS :	Seminiferous epithelial degeneration, unilateral, moderate, (relates to necropsy finding)

ORGANS EXAMINED AND NO ABNORMALITY DETECTED:
ADR , AOR , APP , BRA , CAE , COL , DUO , EYE , FEM , GBL , HEA , ILE , JEJ , LAC , LIV , LUN , LNIN, LNLU, LNMA, LNMS, OES , OPT , PCEN, PCEX, PAR , PIT , PRO , REC , SGSM, SCI , SEM , SKM , SKI , SPN , SPL , STR , STO , THM , THR , TOG , TRA , URE , URB , GALT, sac

Appendix 20 (continued)
Individual Necropsy and Histological Findings: Day 66

PROJECT NUMBER: 520419
TREATMENT: Group 1 (0 ug/dose) MALES

ANIMAL NO:	FINDINGS:
2	Terminal Kill<br>Day of Necropsy: 66
NECROPSY FINDINGS:	
GENERAL COMMENTS :	Tissues not listed below were normal
LUNG :	Discolouration, dark, all lobes, red
TESTIS :	Small, right
THYMUS :	Discolouration, dark, left lobe, red
HISTOLOGICAL FINDINGS:	
EPIDIDYMIS :	Aspermia, unilateral
INJECTION SITE 1 :	Macrophage accumulation, intramuscular, moderate, (with cytoplasmic foreign material and multinucleated giant cells)<br>Inflammation, mononuclear cell, moderate<br>Myofibre necrosis, mild
LUNG :	Agonal congestion/haemorrhage, (relates to necropsy finding)
LYMPH NODE (LUMBAR) :	Macrophage accumulation, minimal, (with multinucleated cells and intracytoplasmic foreign material)<br>Erythrocytosis/erythrophagocytosis, minimal
PARATHYROID GLAND :	Tissue absent from section. No more available
TESTIS :	Immaturity, unilateral, (relates to necropsy finding)<br>Seminiferous epithelial degeneration, unilateral, focal, mild
THYMUS :	Agonal congestion/haemorrhage, (relates to necropsy finding)

(CONTINUED)

Appendix 20 (continued)
Individual Necropsy and Histological Findings: Day 66

PROJECT NUMBER: 520419
TREATMENT: Group 1 (0 ug/dose) MALES

ANIMAL NO:	FINDINGS:
2	(CONTINUED)
HISTOLOGICAL FINDINGS:	
ORGANS EXAMINED AND NO ABNORMALITY DETECTED:	ADR , AOR , APP , BRA , CAE , COL , DUO , EYE , FEM , GBL , HEA , ILE , JEJ , KID , LAC , LIV , LUN , LNIN, LNMA, LNMS, OES , OPT , PCEN, PCEX, PIT , PRO , REC , SGSM, SCI , SEM , SKM , SKI , SPN , SPL , STR , STO , THM , THR , TOG , TRA , URE , URB , GALT, sac
3	Terminal Kill<br>Day of Necropsy: 66
NECROPSY FINDINGS:	
GENERAL COMMENTS :	Tissues not listed below were normal
LUNG :	Discolouration, dark, all lobes
HISTOLOGICAL FINDINGS:	
INJECTION SITE 1 :	Macrophage accumulation, intramuscular, moderate, (with cytoplasmic foreign material and multinucleated giant cells)<br>Inflammation, mononuclear cell, mild
KIDNEY :	Basophilic tubules, focal, minimal
LUNG :	Agonal congestion/haemorrhage, (relates to necropsy finding)
LYMPH NODE (LUMBAR) :	Erythrocytosis/erythrophagocytosis, minimal
PARATHYROID GLAND :	Only one examined
PITUITARY GLAND :	Tissue lost at necropsy

(CONTINUED)

Appendix 20 (continued)
Individual Necropsy and Histological Findings: Day 66

PROJECT NUMBER: 520419
TREATMENT: Group 1 (0 ug/dose) MALES

ANIMAL NO:	FINDINGS:
3	(CONTINUED)

HISTOLOGICAL FINDINGS:
ORGANS EXAMINED AND NO ABNORMALITY DETECTED:
ADR , AOR , APP , BRA , CAE , COL ,
DUO , EPI , EYE , FEM , GBL , HEA ,
ILE , JEJ , LAC , LIV , LUN , LNIN,
LNMA, LNMS, OES , OPT , PCEN, PCEX,
PAR , PRO , REC , SGSM, SCI , SEM ,
SKM , SKI , SPN , SPL , STR , STO ,
TES , THM , THR , TOG , TRA , URE ,
URB , GALT, sac

Appendix 20 (continued)
Individual Necropsy and Histological Findings: Day 66

PROJECT NUMBER:	520419
TREATMENT:	Group 1 (0 ug/dose) FEMALES
ANIMAL NO:	FINDINGS:
10	Terminal Kill<br>Day of Necropsy: 66
NECROPSY FINDINGS:	
GENERAL COMMENTS :	Tissues not listed below were normal
LUNG :	Discolouration, dark, all lobes, red
LYMPH NODE (INGUINAL) :	Not found at necropsy, right
LYMPH NODE (LUMBAR) :	Discolouration, dark, red
HISTOLOGICAL FINDINGS:	
ADRENAL GLAND :	Cortical vacuolated cell focus, minimal
INJECTION SITE 1 :	Macrophage accumulation, intramuscular, mild, (with cytoplasmic foreign material and multinucleated giant cells)<br>Inflammation, mononuclear cell, mild<br>Myofibre necrosis, minimal<br>Regeneration, myofibre, minimal
LUNG :	Agonal congestion/haemorrhage, (relates to necropsy finding)
LYMPH NODE (INGUINAL) :	Only one examined
LYMPH NODE (LUMBAR) :	Erythrocytosis/erythrophagocytosis, mild, (relates to necropsy finding)
LYMPH NODE (MESENTERIC) :	Tissue lost during processing
PARATHYROID GLAND :	Tissue absent from section. No more available

ORGANS EXAMINED AND NO ABNORMALITY DETECTED:
AOR , APP , BRA , CAE , CER , COL , DUO , EYE , FEM , GBL , HEA , ILE , JEJ , KID , LAC , LIV , LUN , LNIN, LNMA, MAM , OES , OPT , OVA , OVD , PCEN, PCEX, PIT , REC , SGSM, SCI ,

(CONTINUED)

Appendix 20 (continued)
Individual Necropsy and Histological Findings: Day 66

PROJECT NUMBER:	520419
TREATMENT:	Group 1 (0 ug/dose) FEMALES

ANIMAL NO:	FINDINGS:
10	(CONTINUED)
HISTOLOGICAL FINDINGS:	
ORGANS EXAMINED AND NO ABNORMALITY DETECTED:	
	SKM , SKI , SPN , SPL , STR , STO , THM , THR , TOG , TRA , URE , URB , UTE , VAG , GALT, sac
11	Terminal Kill<br>Day of Necropsy: 66
NECROPSY FINDINGS:	
GENERAL COMMENTS :	Tissues not listed below were normal
LUNG :	Discolouration, all lobes, red, (mottled)
LYMPH NODE (MESENTERIC) :	Discolouration, dark, red
HISTOLOGICAL FINDINGS:	
ADRENAL GLAND :	Only one medulla examined
HEART :	Inflammatory cell foci, myocardial, minimal
INJECTION SITE 1 :	Inflammation, polymorphonuclear leukocytic, dermal, focal, minimal<br>Macrophage accumulation, intramuscular, moderate, (with cytoplasmic foreign material and multinucleated giant cells)
LUNG :	Agonal congestion/haemorrhage, (relates to necropsy finding)
LYMPH NODE (LUMBAR) :	Macrophage accumulation, mild, (with multinucleated cells and intracytoplasmic foreign material)

(CONTINUED)

Appendix 20 (continued)
Individual Necropsy and Histological Findings: Day 66

PROJECT NUMBER: 520419
TREATMENT: Group 1 (0 ug/dose) FEMALES

ANIMAL NO:	FINDINGS:
11	(CONTINUED)
HISTOLOGICAL FINDINGS:	
LYMPH NODE (MESENTERIC) :	Erythrocytosis/erythrophagocytosis, minimal, (relates to necropsy finding)
PITUITARY GLAND :	Only anterior lobe examined
ORGANS EXAMINED AND NO ABNORMALITY DETECTED:	ADR , AOR , APP , BRA , CAE , CER , COL , DUO , EYE , FEM , GBL , ILE , JEJ , KID , LAC , LIV , LUN , LNIN, LNMA, MAM , OES , OPT , OVA , OVD , PCEN, PCEX, PAR , PIT , REC , SGSM, SCI , SKM , SKI , SPN , SPL , STR , STO , THM , THR , TOG , TRA , URE , URB , UTE , VAG , GALT, sac
12	Terminal Kill<br>Day of Necropsy: 66
NECROPSY FINDINGS:	
GENERAL COMMENTS :	Tissues not listed below were normal
LUNG :	Spongy, all lobes<br>Discolouration, dark, all lobes, red
HISTOLOGICAL FINDINGS:	
ADRENAL GLAND :	Medulla not present
INJECTION SITE 1 :	Macrophage accumulation, intramuscular, mild, (with cytoplasmic foreign material and multinucleated giant cells)<br>Inflammation, mononuclear cell, mild

(CONTINUED)

Appendix 20 (continued)
Individual Necropsy and Histological Findings: Day 66

PROJECT NUMBER:	520419
TREATMENT:	Group 1 (0 ug/dose) FEMALES

ANIMAL NO:	FINDINGS:
12	(CONTINUED)
HISTOLOGICAL FINDINGS:	
LACRIMAL GLAND :	Tissue absent from section. No more available
LUNG :	Agonal congestion/haemorrhage, (relates to necropsy findings)
MAMMARY GLAND :	Duct ectasia, mild
PARATHYROID GLAND :	Tissue absent from section. No more available

ORGANS EXAMINED AND NO ABNORMALITY DETECTED:
ADR , AOR , APP , BRA , CAE , CER , COL , DUO , EYE , FEM , GBL , HEA , ILE , JEJ , KID , LIV , LUN , LNIN, LNLU, LNMA, LNMS, OES , OPT , OVA , OVD , PCEN, PCEX, PIT , REC , SGSM, SCI , SKM , SKI , SPN , SPL , STR , STO , THM , THR , TOG , TRA , URE , URB , UTE , VAG , GALT, sac

Appendix 20 (continued)
Individual Necropsy and Histological Findings: Day 66

PROJECT NUMBER:	520419
TREATMENT:	Group 2 (25 ug/dose) MALES

ANIMAL NO:	FINDINGS:
4	Terminal Kill<br>Day of Necropsy: 66
NECROPSY FINDINGS:	
GENERAL COMMENTS :	Tissues not listed below were normal
LUNG :	Discolouration, dark, all lobes
5	Terminal Kill<br>Day of Necropsy: 66
NECROPSY FINDINGS:	
GENERAL COMMENTS :	Tissues not listed below were normal
LUNG :	Discolouration, all lobes, (mottled)
LYMPH NODE (LUMBAR) :	Discolouration, dark, right, red
6	Terminal Kill<br>Day of Necropsy: 66
NECROPSY FINDINGS:	
GENERAL COMMENTS :	Tissues not listed below were normal
LUNG :	Discolouration, dark, all lobes

Appendix 20 (continued)
Individual Necropsy and Histological Findings: Day 66

PROJECT NUMBER:	520419
TREATMENT:	Group 2 (25 ug/dose) FEMALES

ANIMAL NO:	FINDINGS:
13	Terminal Kill<br>Day of Necropsy: 66
NECROPSY FINDINGS:	
GENERAL COMMENTS :	Tissues not listed below were normal
LUNG :	Discolouration, dark, all lobes, red
14	Terminal Kill<br>Day of Necropsy: 66
NECROPSY FINDINGS:	
GENERAL COMMENTS :	All tissues normal
15	Terminal Kill<br>Day of Necropsy: 66
NECROPSY FINDINGS:	
GENERAL COMMENTS :	Tissues not listed below were normal
LUNG :	Discolouration, dark, all lobes, red<br>Spongy, all lobes
LYMPH NODE (MESENTERIC) :	Discolouration, dark, red

Appendix 20 (continued)
Individual Necropsy and Histological Findings: Day 66

PROJECT NUMBER:	520419
TREATMENT:	Group 3 (50 ug/dose) MALES

ANIMAL NO:	FINDINGS:
7	Terminal Kill<br>Day of Necropsy: 66
NECROPSY FINDINGS:	
GENERAL COMMENTS :	Tissues not listed below were normal
ADRENAL GLAND :	Not found at necropsy, left
LUNG :	Discolouration, dark, all lobes
LYMPH NODE (LUMBAR) :	Not found at necropsy, left
HISTOLOGICAL FINDINGS:	
ADRENAL GLAND :	Only one examined
INJECTION SITE 1 :	Macrophage accumulation, intramuscular, mild, (with cytoplasmic foreign material and multinucleated giant cells)<br>Inflammation, polymorphonuclear leukocytic, moderate<br>Myofibre necrosis, mild<br>Fibrosis, interstitial, mild
KIDNEY :	Tubular mineralisation, medullary, minimal<br>Basophilic tubules, multifocal, minimal
LACRIMAL GLAND :	Tissue lost during processing
LUNG :	Agonal congestion/haemorrhage, (relates to necropsy finding)<br>Osseous metaplasia, focal, minimal
LYMPH NODE (LUMBAR) :	Only one examined
LYMPH NODE (MESENTERIC) :	Erythrocytosis/erythrophagocytosis, minimal
THYROID GLAND :	Inflammatory cell foci, minimal
URINARY BLADDER :	Mineral deposits, epithelial, surface, multifocal, minimal

(CONTINUED)

Appendix 20 (continued)
Individual Necropsy and Histological Findings: Day 66

PROJECT NUMBER: 520419
TREATMENT: Group 3 (50 ug/dose) MALES

ANIMAL NO:	FINDINGS:
7	(CONTINUED)
HISTOLOGICAL FINDINGS:	
ORGANS EXAMINED AND NO ABNORMALITY DETECTED:	
	ADR , AOR , APP , BRA , CAE , COL , DUO , EPI , EYE , FEM , GBL , HEA , ILE , JEJ , LIV , LNIN, LNLU, LNMA, OES , OPT , PCEN, PCEX, PAR , PIT , PRO , REC , SGSM, SCI , SEM , SKM , SKI , SPN , SPL , STR , STO , TES , THM , TOG , TRA , URE , GALT, sac
8	Terminal Kill<br>Day of Necropsy: 66
NECROPSY FINDINGS:	
GENERAL COMMENTS :	Tissues not listed below were normal
LUNG :	Discolouration, all lobes, (mottled)
LYMPH NODE (LUMBAR) :	Enlargement, left, (20 x 6 x 4 mm)
HISTOLOGICAL FINDINGS:	
INJECTION SITE 1 :	Macrophage accumulation, intramuscular, moderate, (with cytoplasmic foreign material and multinucleated giant cells)<br>Inflammation, mononuclear cell, mild<br>Regeneration, myofibre, mild
LUNG :	Inflammatory cell foci, minimal<br>Agonal congestion/haemorrhage, (relates to necropsy finding)
LYMPH NODE (INGUINAL) :	Only one examined
LYMPH NODE (LUMBAR) :	Erythrocytosis/erythrophagocytosis, minimal

(CONTINUED)

Appendix 20 (continued)
Individual Necropsy and Histological Findings: Day 66

PROJECT NUMBER: 520419
TREATMENT: Group 3 (50 ug/dose) MALES

ANIMAL NO:	FINDINGS:
8	(CONTINUED)
HISTOLOGICAL FINDINGS:	
LYMPH NODE (LUMBAR) :	Macrophage accumulation, minimal, (with multinucleated cells and intracytoplasmic foreign material)<br>Lymphoid hyperplasia, moderate, (relates to necropsy finding)
PARATHYROID GLAND :	Tissue absent from section. No more available
ORGANS EXAMINED AND NO ABNORMALITY DETECTED:	ADR , AOR , APP , BRA , CAE , COL , DUO , EPI , EYE , FEM , GBL , HEA , ILE , JEJ , KID , LAC , LIV , LNIN, LNMA, LNMS, OES , OPT , PCEN, PCEX, PIT , PRO , REC , SGSM, SCI , SEM , SKM , SKI , SPN , SPL , STR , STO , TES , THM , THR , TOG , TRA , URE , URB , GALT, sac
9	Terminal Kill<br>Day of Necropsy: 66
NECROPSY FINDINGS:	
GENERAL COMMENTS :	Tissues not listed below were normal
LYMPH NODE (LUMBAR) :	Enlargement, left, (8 x 4 x 3 mm)
HISTOLOGICAL FINDINGS:	
INJECTION SITE 1 :	Macrophage accumulation, intramuscular, moderate, (with cytoplasmic foreign material and multinucleated giant cells)

(CONTINUED)

Appendix 20 (continued)
Individual Necropsy and Histological Findings: Day 66

PROJECT NUMBER:	520419
TREATMENT:	Group 3 (50 ug/dose) MALES

ANIMAL NO:	FINDINGS:
9	(CONTINUED)
HISTOLOGICAL FINDINGS:	
INJECTION SITE 1 :	Fibrosis, interstitial, marked<br>Inflammation, polymorphonuclear leukocytic, marked<br>Myofibre necrosis, moderate
KIDNEY :	Nephropathy, focal, minimal
LUNG :	Inflammatory cell foci, minimal
LYMPH NODE (LUMBAR) :	Erythrocytosis/erythrophagocytosis, minimal<br>Lymphoid hyperplasia, mild, (relates to necropsy finding)<br>Macrophage accumulation, minimal, (with intracytoplasmic foreign material)
PARATHYROID GLAND :	Tissue absent from section. No more available
SKELETAL MUSCLE :	Inflammatory cell foci, minimal
TESTIS :	Segmental hypoplasia, focal, mild

ORGANS EXAMINED AND NO ABNORMALITY DETECTED:
ADR , AOR , APP , BRA , CAE , COL , DUO , EPI , EYE , FEM , GBL , HEA , ILE , JEJ , LAC , LIV , LNIN, LNMA, LNMS, OES , OPT , PCEN, PCEX, PIT , PRO , REC , SGSM, SCI , SEM , SKI , SPN , SPL , STR , STO , THM , THR , TOG , TRA , URE , URB , GALT, sac

Appendix 20 (continued)
Individual Necropsy and Histological Findings: Day 66

PROJECT NUMBER:	520419
TREATMENT:	Group 3 (50 ug/dose) FEMALES

ANIMAL NO:	FINDINGS:
16	Terminal Kill<br>Day of Necropsy: 66
NECROPSY FINDINGS:	
GENERAL COMMENTS :	Tissues not listed below were normal
LYMPH NODE (LUMBAR) :	Discolouration, dark, left<br>Enlargement, left, (10 x 5 x 3 mm)
LYMPH NODE (MESENTERIC) :	Discolouration, dark, red
HISTOLOGICAL FINDINGS:	
ADRENAL GLAND :	Only one medulla examined
INJECTION SITE 1 :	Macrophage accumulation, intramuscular, minimal, (with cytoplasmic foreign material and multinucleated giant cells)<br>Inflammation, polymorphonuclear leukocytic, moderate<br>Myofibre necrosis, mild<br>Regeneration, myofibre, minimal<br>Fibrosis, interstitial, mild
LIVER :	Inflammatory cell infiltration, periportal, minimal
LUNG :	Inflammatory cell foci, perivascular, mild
LYMPH NODE (LUMBAR) :	Only one examined<br>Erythrocytosis/erythrophagocytosis, mild, (relates to necropsy finding discolouration dark)<br>Lymphoid hyperplasia, moderate, (relates to necropsy finding enlargement)<br>Macrophage accumulation, minimal, (with intracytoplasmic foreign material)

(CONTINUED)

Appendix 20 (continued)
Individual Necropsy and Histological Findings: Day 66

PROJECT NUMBER:	520419
TREATMENT:	Group 3 (50 ug/dose) FEMALES

ANIMAL NO:	FINDINGS:
16	(CONTINUED)
HISTOLOGICAL FINDINGS:	
LYMPH NODE (MESENTERIC) :	Erythrocytosis/erythrophagocytosis, minimal, (relates to necropsy finding)
PARATHYROID GLAND :	Only one examined
SKELETAL MUSCLE :	Inflammatory cell foci, minimal
ORGANS EXAMINED AND NO ABNORMALITY DETECTED:	
	ADR , AOR , APP , BRA , CAE , CER , COL , DUO , EYE , FEM , GBL , HEA , ILE , JEJ , KID , LAC , LNIN, LNMA, MAM , OES , OPT , OVA , OVD , PCEN, PCEX, PAR , PIT , REC , SGSM, SCI , SKI , SPN , SPL , STR , STO , THM , THR , TOG , TRA , URE , URB , UTE , VAG , GALT, sac
17	Terminal Kill<br>Day of Necropsy: 66
NECROPSY FINDINGS:	
GENERAL COMMENTS :	Tissues not listed below were normal
LUNG :	Discolouration, all lobes, red, (mottled)
HISTOLOGICAL FINDINGS:	
ADRENAL GLAND :	Diffuse cortical cell hypertrophy, mild

(CONTINUED)

Appendix 20 (continued)
Individual Necropsy and Histological Findings: Day 66

PROJECT NUMBER: 520419
TREATMENT: Group 3 (50 ug/dose) FEMALES

ANIMAL NO:	FINDINGS:
17	(CONTINUED)
HISTOLOGICAL FINDINGS:	
INJECTION SITE 1 :	Macrophage accumulation, intramuscular, focal, minimal, (with cytoplasmic foreign material and multinucleated giant cells)<br>Inflammation, mononuclear cell, minimal, (with focal polymorphonuclear cells)
KIDNEY :	Tubular mineralisation, medullary, minimal<br>Basophilic tubules, multifocal, minimal
LIVER :	Oval cell hyperplasia, minimal
LUNG :	Agonal congestion/haemorrhage, (relates to necropsy finding)
LYMPH NODE (INGUINAL) :	Only one examined
LYMPH NODE (LUMBAR) :	Tissue absent from section. No more available
PARATHYROID GLAND :	Tissue absent from section. No more available
SKELETAL MUSCLE :	Inflammatory cell foci, minimal
GUT ASSOCIATED LYMPHOID TISSUE :	Inflammation, Peyer's patch, focal, minimal
ORGANS EXAMINED AND NO ABNORMALITY DETECTED:	AOR , APP , BRA , CAE , CER , COL , DUO , EYE , FEM , GBL , HEA , ILE , JEJ , LAC , LUN , LNIN, LNMA, LNMS, MAM , OES , OPT , OVA , OVD , PCEN, PCEX, PIT , REC , SGSM, SCI , SKI , SPN , SPL , STR , STO , THM , THR , TOG , TRA , URE , URB , UTE , VAG , sac

Appendix 20 (continued)
Individual Necropsy and Histological Findings: Day 66

PROJECT NUMBER:	520419
TREATMENT:	Group 3 (50 ug/dose) FEMALES

ANIMAL NO:	FINDINGS:
18	Terminal Kill<br>Day of Necropsy: 66
NECROPSY FINDINGS:	
GENERAL COMMENTS :	Tissues not listed below were normal
LUNG :	Discolouration, dark, all lobes, red
OVIDUCT :	Cyst, right, clear, (one, 5 mm)
HISTOLOGICAL FINDINGS:	
AORTA :	Mineralisation, medial, mild
INJECTION SITE 1 :	Macrophage accumulation, intramuscular, moderate, (with cytoplasmic foreign material and multinucleated giant cells)<br>Inflammation, polymorphonuclear leukocytic, mild<br>Regeneration, myofibre, minimal<br>Fibrosis, interstitial, minimal<br>Mineralisation, minimal
KIDNEY :	Tubular mineralisation, cortical, minimal<br>Basophilic tubules, minimal
LUNG :	Agonal congestion/haemorrhage, (relates to necropsy finding)
LYMPH NODE (INGUINAL) :	Only one examined
LYMPH NODE (LUMBAR) :	Erythrocytosis/erythrophagocytosis, minimal<br>Macrophage accumulation, minimal, (with multinucleated cells and intracytoplasmic foreign material)<br>Lymphoid hyperplasia, mild
OVIDUCT :	Cyst, (relates to necropsy finding)
PARATHYROID GLAND :	Only one examined
PITUITARY GLAND :	Posterior lobe not present
SKELETAL MUSCLE :	Inflammatory cell foci, minimal

(CONTINUED)

Appendix 20 (continued)
Individual Necropsy and Histological Findings: Day 66

PROJECT NUMBER:	520419
TREATMENT:	Group 3 (50 ug/dose) FEMALES

ANIMAL NO:	FINDINGS:
18	(CONTINUED)

HISTOLOGICAL FINDINGS:
ORGANS EXAMINED AND NO ABNORMALITY DETECTED:
ADR , APP , BRA , CAE , CER , COL ,
DUO , EYE , FEM , GBL , HEA , ILE ,
JEJ , LAC , LIV , LUN , LNIN, LNMA,
LNMS, MAM , OES , OPT , OVA , PCEN,
PCEX, PAR , PIT , REC , SGSM, SCI ,
SKI , SPN , SPL , STR , STO , THM ,
THR , TOG , TRA , URE , URB , UTE ,
VAG , GALT, sac

Appendix 21
Individual Necropsy and Histological Findings: Day 92

Abbreviations Used

INJ1	=	Injection Site 1
INJ2	=	Injection Site 2
LNIN	=	Inguinal Lymph Node
LULU	=	Lumbar Lymph Node

Appendix 21 (continued)
Individual Necropsy and Histological Findings: Day 92

PROJECT NUMBER:	520419
TREATMENT:	Group 1 (0 ug/dose) MALES

ANIMAL NO:	FINDINGS:
19	Recovery Kill<br>Day of Necropsy: 92
NECROPSY FINDINGS:	
GENERAL COMMENTS :	Tissues not listed below were normal
ADRENAL GLAND :	Discolouration, both, dark, red
LUNG :	Discolouration, dark, all lobes, red
TRACHEA :	Fluid accumulation, pale, (frothy)
HISTOLOGICAL FINDINGS:	
INJECTION SITE 1 :	Macrophage accumulation, intramuscular, moderate, (with cytoplasmic foreign material and multinucleated giant cells)
LYMPH NODE (LUMBAR) :	Only one examined<br>Macrophage accumulation, minimal, (with multinucleated cells and intracytoplasmic foreign material)
ORGANS EXAMINED AND NO ABNORMALITY DETECTED:	LNIN
20	Recovery Kill<br>Day of Necropsy: 92
NECROPSY FINDINGS:	
GENERAL COMMENTS :	Tissues not listed below were normal
LUNG :	Spongy, all lobes<br>Discolouration, all lobes, (mottled)
LYMPH NODE (LUMBAR) :	Discolouration, both, dark, red
TRACHEA :	Fluid accumulation, pale, (froth filled)

(CONTINUED)

Appendix 21 (continued)
Individual Necropsy and Histological Findings: Day 92

PROJECT NUMBER:	520419
TREATMENT:	Group 1 (0 ug/dose) MALES

ANIMAL NO:	FINDINGS:
20	(CONTINUED)
HISTOLOGICAL FINDINGS:	
INJECTION SITE 1 :	Macrophage accumulation, intramuscular, moderate, (with cytoplasmic foreign material and multinucleated giant cells)
LYMPH NODE (LUMBAR) :	Erythrocytosis/erythrophagocytosis, moderate, (relates to necropsy finding)
	Macrophage accumulation, mild, (with multinucleated cells and intracytoplasmic foreign material)
ORGANS EXAMINED AND NO ABNORMALITY DETECTED:	
	LNIN
21	Recovery Kill<br>Day of Necropsy: 92
NECROPSY FINDINGS:	
GENERAL COMMENTS :	All tissues normal
HISTOLOGICAL FINDINGS:	
INJECTION SITE 1 :	Macrophage accumulation, intramuscular, mild, (with cytoplasmic foreign material and multinucleated giant cells)
LYMPH NODE (LUMBAR) :	Macrophage accumulation, mild, (with multinucleated cells and intracytoplasmic foreign material)

(CONTINUED)

Appendix 21 (continued)
Individual Necropsy and Histological Findings: Day 92

PROJECT NUMBER: 520419
TREATMENT: Group 1 (0 ug/dose) MALES

ANIMAL NO:	FINDINGS:
21	(CONTINUED)

HISTOLOGICAL FINDINGS:
ORGANS EXAMINED AND NO ABNORMALITY DETECTED:
LNIN

Appendix 21 (continued)
Individual Necropsy and Histological Findings: Day 92

PROJECT NUMBER: 520419
TREATMENT: Group 1 (0 ug/dose) FEMALES

ANIMAL NO:	FINDINGS:
28	Recovery Kill<br>Day of Necropsy: 92
NECROPSY FINDINGS:	
GENERAL COMMENTS :	Tissues not listed below were normal
LUNG :	Discolouration, all lobes, (mottled)<br>Spongy, all lobes
TRACHEA :	Fluid accumulation, pale, (froth filled)
HISTOLOGICAL FINDINGS:	
LYMPH NODE (LUMBAR) :	Macrophage accumulation, minimal, (with multinucleated cells and intracytoplasmic foreign material)
ORGANS EXAMINED AND NO ABNORMALITY DETECTED:	INJ1, LNIN
29	Recovery Kill<br>Day of Necropsy: 92
NECROPSY FINDINGS:	
GENERAL COMMENTS :	Tissues not listed below were normal
LUNG :	Discolouration, all lobes, (mottled)<br>Spongy, all lobes
TRACHEA :	Fluid accumulation, dark, red, (froth filled)

(CONTINUED)

Appendix 21 (continued)
Individual Necropsy and Histological Findings: Day 92

PROJECT NUMBER:	520419
TREATMENT:	Group 1 (0 ug/dose) FEMALES

ANIMAL NO:	FINDINGS:
29	(CONTINUED)
HISTOLOGICAL FINDINGS:	
INJECTION SITE 1 :	Macrophage accumulation, intramuscular, moderate, (with cytoplasmic foreign material and multinucleated giant cells)
LYMPH NODE (LUMBAR) :	Tissue absent from section. No more available
ORGANS EXAMINED AND NO ABNORMALITY DETECTED:	LNIN
30	Recovery Kill<br>Day of Necropsy: 92
NECROPSY FINDINGS:	
GENERAL COMMENTS :	Tissues not listed below were normal
LUNG :	Discolouration, (mottled)
HISTOLOGICAL FINDINGS:	
LYMPH NODE (LUMBAR) :	Only one examined
ORGANS EXAMINED AND NO ABNORMALITY DETECTED:	INJ1, LNIN, LNLU

Appendix 21 (continued)
Individual Necropsy and Histological Findings: Day 92

PROJECT NUMBER:	520419
TREATMENT:	Group 2 (25 ug/dose) MALES

ANIMAL NO:	FINDINGS:
22	Recovery Kill<br>Day of Necropsy: 92
NECROPSY FINDINGS:	
GENERAL COMMENTS :	Tissues not listed below were normal
LUNG :	Discolouration, dark, all lobes, red
LYMPH NODE (MANDIBULAR) :	Discolouration, dark, red
23	Recovery Kill<br>Day of Necropsy: 92
NECROPSY FINDINGS:	
GENERAL COMMENTS :	Tissues not listed below were normal
LUNG :	Discolouration, dark, all lobes, red
TRACHEA :	Fluid accumulation, pale, (frothy)
24	Recovery Kill<br>Day of Necropsy: 92
NECROPSY FINDINGS:	
GENERAL COMMENTS :	Tissues not listed below were normal
LUNG :	Discolouration, dark, all lobes, red

Appendix 21 (continued)
Individual Necropsy and Histological Findings: Day 92

PROJECT NUMBER:	520419
TREATMENT:	Group 2 (25 ug/dose) FEMALES

ANIMAL NO:	FINDINGS:
31	Recovery Kill<br>Day of Necropsy: 92
NECROPSY FINDINGS:	
GENERAL COMMENTS :	Tissues not listed below were normal
LUNG :	Spongy, all lobes<br>Discolouration, all lobes, (mottled)
OVIDUCT :	Cyst, right, clear, (one, 3 mm)
TRACHEA :	Fluid accumulation, pale, (froth filled)
32	Recovery Kill<br>Day of Necropsy: 92
NECROPSY FINDINGS:	
GENERAL COMMENTS :	Tissues not listed below were normal
LUNG :	Discolouration, all lobes, (mottled)<br>Spongy, all lobes
TRACHEA :	Fluid accumulation, dark, red, (froth filled)
33	Recovery Kill<br>Day of Necropsy: 92
NECROPSY FINDINGS:	
GENERAL COMMENTS :	Tissues not listed below were normal
OVARY :	Foci, dark, both, few, (2 mm)

Appendix 21 (continued)
Individual Necropsy and Histological Findings: Day 92

PROJECT NUMBER:	520419
TREATMENT:	Group 3 (50 ug/dose) MALES

ANIMAL NO:	FINDINGS:
25	Recovery Kill<br>Day of Necropsy: 92
NECROPSY FINDINGS:	
GENERAL COMMENTS :	Tissues not listed below were normal
LUNG :	Discolouration, dark, all lobes, red
LYMPH NODE (LUMBAR) :	Discolouration, right, dark, red
THYROID GLAND :	Small, right
HISTOLOGICAL FINDINGS:	
INJECTION SITE 1 :	Macrophage accumulation, intramuscular, moderate, (with cytoplasmic foreign material and multinucleated giant cells)<br>Fibrosis, interstitial, mild<br>Regeneration, myofibre, minimal<br>Inflammation, with necrosis, minimal
LYMPH NODE (LUMBAR) :	Erythrocytosis/erythrophagocytosis, mild, (relates to necropsy finding)<br>Macrophage accumulation, minimal, (with multinucleated cells and intracytoplasmic foreign material)

ORGANS EXAMINED AND NO ABNORMALITY DETECTED:
LNIN

Appendix 21 (continued)
Individual Necropsy and Histological Findings: Day 92

PROJECT NUMBER: 520419
TREATMENT: Group 3 (50 ug/dose) MALES

ANIMAL NO:	FINDINGS:
26	Recovery Kill<br>Day of Necropsy: 92
NECROPSY FINDINGS:	
GENERAL COMMENTS :	Tissues not listed below were normal
LYMPH NODE (LUMBAR) :	Discolouration, both, dark, red
HISTOLOGICAL FINDINGS:	
LYMPH NODE (LUMBAR) :	Erythrocytosis/erythrophagocytosis, mild, (relates to necropsy finding)
ORGANS EXAMINED AND NO ABNORMALITY DETECTED:	INJ1, LNIN
27	Recovery Kill<br>Day of Necropsy: 92
NECROPSY FINDINGS:	
GENERAL COMMENTS :	Tissues not listed below were normal
LUNG :	Spongy, all lobes<br>Discolouration, dark, all lobes, red
LYMPH NODE (INGUINAL) :	Discolouration, right, dark, red
LYMPH NODE (LUMBAR) :	Discolouration, both, dark, red
TRACHEA :	Fluid accumulation, dark
HISTOLOGICAL FINDINGS:	
INJECTION SITE 1 :	Macrophage accumulation, intramuscular, moderate, (with cytoplasmic foreign material and multinucleated giant cells)<br>Fibrosis, interstitial, mild<br>Inflammation, with necrosis, mild

(CONTINUED)

Appendix 21 (continued)
Individual Necropsy and Historical Findings: Day 92

PROJECT NUMBER:	520419
TREATMENT:	Group 3 (50 ug/dose) MALES

ANIMAL NO:	FINDINGS:
27	(CONTINUED)
HISTOLOGICAL FINDINGS:	
INJECTION SITE 1 :	Regeneration, myofibre, minimal<br>Myofibre necrosis, minimal
LYMPH NODE (INGUINAL) :	No histological correlation with necropsy finding
LYMPH NODE (LUMBAR) :	Erythrocytosis/erythrophagocytosis, minimal, (relates to necropsy finding)<br>Only one examined
ORGANS EXAMINED AND NO ABNORMALITY DETECTED:	INJ2, LNIN

Appendix 21 (continued)
Individual Necropsy and Histological Findings: Day 92

PROJECT NUMBER:	520419
TREATMENT:	Group 3 (50 ug/dose) FEMALES

ANIMAL NO:	FINDINGS:
34	Recovery Kill<br>Day of Necropsy: 92
NECROPSY FINDINGS:	
GENERAL COMMENTS :	Tissues not listed below were normal
LUNG :	Discolouration, dark, all lobes, red<br>Spongy, all lobes
TRACHEA :	Fluid accumulation, pale, (froth filled)
HISTOLOGICAL FINDINGS:	
INJECTION SITE 1 :	Macrophage accumulation, intramuscular, moderate, (with cytoplasmic foreign material and multinucleated giant cells)<br>Inflammation, with necrosis, mild<br>Myofibre necrosis, minimal<br>Regeneration, myofibre, minimal<br>Fibrosis, interstitial, minimal
LYMPH NODE (LUMBAR) :	Tissue absent from section. No more available
ORGANS EXAMINED AND NO ABNORMALITY DETECTED:	LNIN
35	Recovery Kill<br>Day of Necropsy: 92
NECROPSY FINDINGS:	
GENERAL COMMENTS :	Tissues not listed below were normal
LUNG :	Spongy, all lobes<br>Discolouration, all lobes, (mottled)

(CONTINUED)

Appendix 21 (continued)
Individual Necropsy and Histological Findings: Day 92

PROJECT NUMBER:	520419
TREATMENT:	Group 3 (50 ug/dose) FEMALES

ANIMAL NO:	FINDINGS:
35	(CONTINUED)
NECROPSY FINDINGS:	
LYMPH NODE (MANDIBULAR) :	Discolouration, dark, red
TRACHEA :	Fluid accumulation, pale, red, (frothy)
HISTOLOGICAL FINDINGS:	
INJECTION SITE 1 :	Macrophage accumulation, intramuscular, minimal, (with cytoplasmic foreign material and multinucleated giant cells)<br>Inflammation, mononuclear cell, minimal
LYMPH NODE (INGUINAL) :	Macrophage accumulation, minimal, (with cytoplasmic foreign material and multinucleated giant cells)
ORGANS EXAMINED AND NO ABNORMALITY DETECTED:	LNLU
36	Recovery Kill<br>Day of Necropsy: 92
NECROPSY FINDINGS:	
GENERAL COMMENTS :	All tissues normal
HISTOLOGICAL FINDINGS:	
LYMPH NODE (LUMBAR) :	Tissue absent from section. No more available
ORGANS EXAMINED AND NO ABNORMALITY DETECTED:	INJ1, LNIN

Appendix 22

Absolute Organ Weights (g) : Individual Values: Day 66

Group	:	1	2	3
Test Item	:	Control	MenPF-1	MenPF-1
Dosage (µg/dose)	:	0	25	50

Group / sex	Animal No.	Body Weight (kg)	Adrenals	Brain	Epididy-mides	Heart	Kidneys	Liver	Lung	Pituitary	Prostate
1M	1	3.1	0.2527	10.77	2.0622	10.03	16.87	79.66	24.15	0.031	0.84
	2	3.3	0.2860	9.70	2.0353	7.78	18.64	88.53	20.96	0.029	1.51
	3	3.2	0.3589	10.11	2.3263	9.08	21.10	126.91	22.51	-	0.56
2M	4	3.5	0.2732	9.74	1.9023	9.11	23.99	142.73	28.60	0.040	1.05
	5	3.3	0.3187	9.49	2.6223	9.58	22.72	163.34	24.14	0.032	1.27
	6	3.4	0.3416	9.94	2.8897	10.86	18.92	114.52	30.80	0.044	1.57
3M	7	3.6	0.1259	9.85	2.9247	9.32	20.67	132.93	23.03	0.027	1.05
	8	3.2	0.2550	9.75	2.1088	8.05	18.64	91.68	28.60	0.026	0.82
	9	3.4	0.2526	9.54	2.3121	9.00	18.28	112.96	15.91	0.026	0.78

Animal 3 - Piuitary gland weight not recorded in error at necropsy

Animal 7 - Left adrenal gland lost at necropsy; excluded from statistical analysis

Appendix 22 (continued)
Absolute Organ Weights (g) : Individual Values: Day 66

Group	:	1	2	3
Test Item	:	Control	MenPF-1	MenPF-1
Dosage (µg/dose)	:	0	25	50

Group / sex	Animal No.	Spleen	Testes	Thymus	Thyroid
1M	1	1.00	3.43	3.03	0.346
	2	0.95	3.87	3.55	0.530
	3	1.21	5.90	2.02	0.341
2M	4	1.02	5.11	2.92	0.229
	5	1.04	6.52	2.99	0.301
	6	1.14	5.25	3.31	0.229
3M	7	1.35	4.72	4.23	0.281
	8	1.40	5.85	3.47	0.261
	9	1.14	6.10	2.89	0.392

Appendix 22 (continued)
Absolute Organ Weights (g) : Individual Values: Day 66

Group	:	1	2	3
Test Item	:	Control	MenPF-1	MenPF-1
Dosage (µg/dose)	:	0	25	50

Group / sex	Animal No.	Body Weight (kg)	Adrenals	Brain	Heart	Kidneys	Liver	Lung	Ovaries	Pituitary	Spleen
1F	10	3.9	0.2162	10.39	8.11	22.07	143.53	28.53	0.350	0.056	1.45
	11	4.1	0.2991	9.68	11.66	24.29	135.89	27.33	0.391	0.035	1.88
	12	3.9	0.2307	10.81	9.46	19.03	112.30	27.94	0.399	0.026	2.28
2F	13	3.6	0.3718	10.06	8.75	20.37	101.47	23.54	0.355	0.039	1.71
	14	3.7	0.3533	9.35	10.23	16.68	90.64	12.53	0.391	0.030	1.58
	15	4.4	0.3075	9.64	10.93	23.76	177.00	28.46	0.568	0.038	2.11
3F	16	3.2	0.3428	9.27	7.61	16.37	74.32	11.55	0.415	0.042	1.78
	17	4.1	0.2856	9.74	8.44	23.33	149.32	19.58	0.427	0.023	1.11
	18	4.2	0.3600	9.71	11.23	27.39	166.01	32.79	0.671	0.029	1.27

Appendix 22 (continued)
Absolute Organ Weights (g) : Individual Values: Day 66

Group	:	1	2	3
Test Item	:	Control	MenPF-1	MenPF-1
Dosage (µg/dose)	:	0	25	50

Group / sex	Animal No.	Thymus	Thyroid	Uterus
1F	10	2.61	0.437	8.76
	11	4.01	0.273	8.15
	12	2.68	0.378	7.25
2F	13	3.05	0.450	9.14
	14	3.21	0.460	12.08
	15	4.37	0.369	9.63
3F	16	2.74	0.428	7.09
	17	2.99	0.354	8.40
	18	5.11	0.427	9.60

Appendix 23
Absolute Organ Weights (g) : Individual Values: Day 92

Group	:	1	2	3
Test Item	:	Control	MenPF-1	MenPF-1
Dosage (µg/dose)	:	0	25	50

Group / sex	Animal No.	Body Weight (kg)	Adrenals	Brain	Epididy-mides	Heart	Kidneys	Liver	Lung	Pituitary	Prostate
1M	19	3.8	0.2218	10.55	2.1469	9.99	20.95	108.62	25.63	0.028	1.00
	20	3.4	0.2673	9.91	1.9302	8.72	19.06	104.81	29.27	0.015	0.87
	21	3.4	0.2975	9.86	2.5220	10.34	21.11	113.16	15.30	0.016	1.10
2M	22	3.7	0.3432	9.51	1.8618	9.98	22.64	147.33	31.12	0.024	0.80
	23	3.4	0.2885	10.44	1.7122	8.62	16.32	109.57	23.60	0.021	0.92
	24	3.3	0.3394	9.31	2.3734	9.01	14.90	89.90	31.27	0.021	0.67
3M	25	3.3	0.2986	9.91	2.3776	8.18	16.26	102.19	30.25	0.014	0.88
	26	3.0	0.4134	10.38	2.3246	8.64	16.38	77.62	12.40	0.044	1.48
	27	3.5	0.3338	10.22	1.6869	8.29	17.61	98.74	26.90	0.032	0.82

Appendix 23 (continued)
Absolute Organ Weights (g) : Individual Values: Day 92

Group	:	1	2	3
Test Item	:	Control	MenPF-1	MenPF-1
Dosage (µg/dose)	:	0	25	50

Group / sex	Animal No.	Spleen	Testes	Thymus	Thyroid
1M	19	0.62	6.28	4.44	0.222
	20	0.93	5.76	3.25	0.176
	21	1.47	7.02	2.62	0.357
2M	22	1.09	4.77	4.34	0.351
	23	1.22	4.57	1.42	0.186
	24	1.24	6.08	3.76	0.292
3M	25	1.45	5.16	2.91	0.207
	26	1.19	5.80	1.77	0.231
	27	0.81	5.30	3.52	0.184

Appendix 23 (continued)
Absolute Organ Weights (g) : Individual Values: Day 92

Group	:	1	2	3
Test Item	:	Control	MenPF-1	MenPF-1
Dosage (µg/dose)	:	0	25	50

Group / sex	Animal No.	Body Weight (kg)	Adrenals	Brain	Heart	Kidneys	Liver	Lung	Ovaries	Pituitary	Spleen
1F	28	4.2	0.2788	10.43	8.63	19.75	115.85	35.29	0.400	0.027	1.91
	29	4.3	0.2719	9.84	9.89	21.29	126.72	36.34	0.585	0.034	1.48
	30	5.0	0.3647	8.90	11.17	25.45	145.41	21.54	0.490	0.017	2.10
2F	31	3.9	0.2977	8.79	8.75	17.10	103.43	19.49	0.269	0.031	2.11
	32	4.2	0.2550	9.54	8.88	20.04	94.36	28.78	0.379	0.019	1.18
	33	3.5	0.2172	10.15	8.21	17.61	87.88	19.79	0.536	0.042	2.13
3F	34	4.2	0.3504	8.66	11.87	18.50	139.43	37.57	0.462	0.004	1.17
	35	4.2	0.2493	10.18	9.82	20.89	130.71	32.19	0.743	0.028	1.55
	36	4.6	0.3062	9.57	9.90	22.21	149.16	20.95	0.409	0.031	1.22

Appendix 23 (continued)
Absolute Organ Weights (g) : Individual Values: Day 92

Group	:	1	2	3
Test Item	:	Control	MenPF-1	MenPF-1
Dosage (µg/dose)	:	0	25	50

Group / sex	Animal No.	Thymus	Thyroid	Uterus
1F	28	3.95	0.270	6.80
	29	3.66	0.390	10.62
	30	5.88	0.402	10.32
2F	31	2.39	0.350	7.60
	32	3.87	0.431	10.09
	33	1.93	0.269	11.40
3F	34	3.08	0.416	9.15
	35	2.60	0.240	11.85
	36	3.56	0.454	8.24

Appendix 24
Relative Organ Weights (% Body Weights) : Individual Values: Day 66

Group	:	1	2	3
Test Item	:	Control	MenPF-1	MenPF-1
Dosage (µg/dose)	:	0	25	50

Group / sex	Animal No.	Adrenals	Brain	Epididy-mides	Heart	Kidneys	Liver	Lung	Pituitary	Prostate	Spleen
1M	1	0.0082	0.347	0.06652	0.324	0.544	2.570	0.779	0.0010	0.027	0.032
	2	0.0087	0.294	0.06168	0.236	0.565	2.683	0.635	0.0009	0.046	0.029
	3	0.0112	0.316	0.07270	0.284	0.659	3.966	0.703		0.018	0.038
2M	4	0.0078	0.278	0.05435	0.260	0.685	4.078	0.817	0.0011	0.030	0.029
	5	0.0097	0.288	0.07946	0.290	0.688	4.950	0.732	0.0010	0.038	0.032
	6	0.0101	0.292	0.08499	0.319	0.556	3.368	0.906	0.0013	0.046	0.034
3M	7	0.0035	0.274	0.08124	0.259	0.574	3.693	0.640	0.0008	0.029	0.038
	8	0.0080	0.305	0.06590	0.252	0.583	2.865	0.894	0.0008	0.026	0.044
	9	0.0074	0.281	0.06800	0.265	0.538	3.322	0.468	0.0008	0.023	0.034

Animal 3 - Pituitary gland weight not recorded in error at necropsy
Animal 7 - Left adrenal gland lost at necropsy; excluded from statistical analysis

Appendix 24 (continued)
Relative Organ Weights (% Body Weights) : Individual Values: Day 66

Group	:	1	2	3
Test Item	:	Control	MenPF-1	MenPF-1
Dosage (µg/dose)	:	0	25	50

Group / sex	Animal No.	Testes	Thymus	Thyroid
1M	1	0.111	0.098	0.0112
	2	0.117	0.108	0.0161
	3	0.184	0.063	0.0107
2M	4	0.146	0.083	0.0065
	5	0.198	0.091	0.0091
	6	0.154	0.097	0.0067
3M	7	0.131	0.118	0.0078
	8	0.183	0.108	0.0082
	9	0.179	0.085	0.0115

Appendix 24 (continued)
Relative Organ Weights (% Body Weights) : Individual Values: Day 66

Group	:	1	2	3
Test Item	:	Control	MenPF-1	MenPF-1
Dosage (µg/dose)	:	0	25	50

Group / sex	Animal No.	Adrenals	Brain	Heart	Kidneys	Liver	Lung	Ovaries	Pituitary	Spleen	Thymus
1F	10	0.0055	0.266	0.208	0.566	3.680	0.732	0.0090	0.0014	0.037	0.067
	11	0.0073	0.236	0.284	0.592	3.314	0.667	0.0095	0.0009	0.046	0.098
	12	0.0059	0.277	0.243	0.488	2.879	0.716	0.0102	0.0007	0.058	0.069
2F	13	0.0103	0.279	0.243	0.566	2.819	0.654	0.0099	0.0011	0.048	0.085
	14	0.0096	0.253	0.276	0.451	2.450	0.339	0.0106	0.0008	0.043	0.087
	15	0.0070	0.219	0.248	0.540	4.023	0.647	0.0129	0.0009	0.048	0.099
3F	16	0.0107	0.290	0.238	0.512	2.323	0.361	0.0130	0.0013	0.056	0.086
	17	0.0070	0.238	0.206	0.569	3.642	0.478	0.0104	0.0006	0.027	0.073
	18	0.0086	0.231	0.267	0.652	3.953	0.781	0.0160	0.0007	0.030	0.122

Appendix 24 (continued)
Relative Organ Weights (% Body Weights) : Individual Values: Day 66

Group	:	1	2	3
Test Item	:	Control	MenPF-1	MenPF-1
Dosage (µg/dose)	:	0	25	50

Group / sex	Animal No.	Thyroid	Uterus
1F	10	0.0112	0.225
	11	0.0067	0.199
	12	0.0097	0.186
2F	13	0.0125	0.254
	14	0.0124	0.326
	15	0.0084	0.219
3F	16	0.0134	0.222
	17	0.0086	0.205
	18	0.0102	0.229

Appendix 25
Relative Organ Weights (% Body Weights) : Individual Values: Day 92

Group	:	1	2	3
Test Item	:	Control	MenPF-1	MenPF-1
Dosage (µg/dose)	:	0	25	50

Group / sex	Animal No.	Adrenals	Brain	Epididy-mides	Heart	Kidneys	Liver	Lung	Pituitary	Prostate	Spleen
1M	19	0.0058	0.278	0.05650	0.263	0.551	2.858	0.674	0.0007	0.026	0.016
	20	0.0079	0.291	0.05677	0.256	0.561	3.083	0.861	0.0004	0.026	0.027
	21	0.0088	0.290	0.07418	0.304	0.621	3.328	0.450	0.0005	0.032	0.043
2M	22	0.0093	0.257	0.05032	0.270	0.612	3.982	0.841	0.0006	0.022	0.029
	23	0.0085	0.307	0.05036	0.254	0.480	3.223	0.694	0.0006	0.027	0.036
	24	0.0103	0.282	0.07192	0.273	0.452	2.724	0.948	0.0006	0.020	0.038
3M	25	0.0091	0.300	0.07205	0.248	0.493	3.097	0.917	0.0004	0.027	0.044
	26	0.0138	0.346	0.07749	0.288	0.546	2.587	0.413	0.0015	0.049	0.040
	27	0.0095	0.292	0.04820	0.237	0.503	2.821	0.769	0.0009	0.023	0.023

Appendix 25 (continued)
Relative Organ Weights (% Body Weights) : Individual Values: Day 92

Group	:	1	2	3
Test Item	:	Control	MenPF-1	MenPF-1
Dosage (µg/dose)	:	0	25	50

Group / sex	Animal No.	Testes	Thymus	Thyroid
1M	19	0.165	0.117	0.0058
	20	0.169	0.096	0.0052
	21	0.206	0.077	0.0105
2M	22	0.129	0.117	0.0095
	23	0.134	0.042	0.0055
	24	0.184	0.114	0.0088
3M	25	0.156	0.088	0.0063
	26	0.193	0.059	0.0077
	27	0.151	0.101	0.0053

Appendix 25 (continued)
Relative Organ Weights (% Body Weights) : Individual Values: Day 92

Group	:	1	2	3
Test Item	:	Control	MenPF-1	MenPF-1
Dosage (µg/dose)	:	0	25	50

Group / sex	Animal No.	Adrenals	Brain	Heart	Kidneys	Liver	Lung	Ovaries	Pituitary	Spleen	Thymus
1F	28	0.0066	0.248	0.205	0.470	2.758	0.840	0.0095	0.0006	0.045	0.094
	29	0.0063	0.229	0.230	0.495	2.947	0.845	0.0136	0.0008	0.034	0.085
	30	0.0073	0.178	0.223	0.509	2.908	0.431	0.0098	0.0003	0.042	0.118
2F	31	0.0076	0.225	0.224	0.438	2.652	0.500	0.0069	0.0008	0.054	0.061
	32	0.0061	0.227	0.211	0.477	2.247	0.685	0.0090	0.0005	0.028	0.092
	33	0.0062	0.290	0.235	0.503	2.511	0.565	0.0153	0.0012	0.061	0.055
3F	34	0.0083	0.206	0.283	0.440	3.320	0.895	0.0110	0.0001	0.028	0.073
	35	0.0059	0.242	0.234	0.497	3.112	0.766	0.0177	0.0007	0.037	0.062
	36	0.0067	0.208	0.215	0.483	3.243	0.455	0.0089	0.0007	0.027	0.077

Appendix 25 (continued)
Relative Organ Weights (% Body Weights) : Individual Values: Day 92

Group	:	1	2	3
Test Item	:	Control	MenPF-1	MenPF-1
Dosage (µg/dose)	:	0	25	50

Group / sex	Animal No.	Thyroid	Uterus
1F	28	0.0064	0.162
	29	0.0091	0.247
	30	0.0080	0.206
2F	31	0.0090	0.195
	32	0.0103	0.240
	33	0.0077	0.326
3F	34	0.0099	0.218
	35	0.0057	0.282
	36	0.0099	0.179

Appendix 26
Pathology Report

charles river

FINAL REPORT

Study Phase: Pathology

Test Facility Study No. 520419

A 9 Week Study of MenPF-1 Vaccine by Intramuscular Injection in Rabbits with a 4 Week Recovery Period

SPONSOR:
Oxford Vaccine Group
Department of Paediatrics
University of Oxford
Room 02-46-07
Children's Hospital
Oxford, OX3 9DU
UK

TEST FACILITY:
Charles River
Preclinical Services, Tranent
Edinburgh, EH33 2NE
UK

Appendix 26 (continued)
Pathology Report

TABLE OF CONTENTS

1. RESPONSIBLE PERSONNEL 3
2. SUMMARY 3
2.1. Main Study (Day 66) 3
2.2. Recovery Study (Day 92) 3
3. INTRODUCTION 3
4. MATERIALS AND METHODS 4
4.1. Peer Review 6
4.2. Computerized Systems 6
5. RESULTS AND DISCUSSIONS 6
5.1. Gross Pathology 6
5.1.1. Scheduled Euthanasia (Day 66) 6
5.1.2. Scheduled Euthanasia (Day 92) 7
5.2. Organ Weights 7
5.3. Histopathology 7
5.3.1. Scheduled Euthanasia (Day 66) 7
5.3.2. Scheduled Euthanasia (Day 92) 9
6. CONCLUSIONS 10
7. REPORT APPROVAL 11

Appendix 26 (continued)
Pathology Report

1. RESPONSIBLE PERSONNEL

Study Pathologist — Lise Bertrand, DVM, MSc, DESV, Dipl ECVP
Charles River, Edinburgh, UK

2. SUMMARY

2.1. Main Study (Day 66)

Intramuscular administration of MenPF-1 vaccine to rabbits on 4 occasions at 50 µg/dose resulted in the accumulation of foreign material-laden macrophages and giant cells at the injection site for all animals. Polymorphonuclear inflammation was noted in 2/3 males and 2/3 females, and mononuclear inflammation was noted in 1/3 males and 1/3 females. Myofibre necrosis and/or regeneration, interstitial fibrosis and/or mineralisation were also observed in treated injection sites.

Lumbar lymph node enlargement was observed at necropsy in 2/3 males and 1/3 females, with corresponding lymphoid hyperplasia. Accumulation of foreign material-laden macrophages and giant cells was noted in the lumbar lymph nodes of 2/3 males and 2/2 females.

2.2. Recovery Study (Day 92)

After a 4 week recovery period, a number of findings persisted in treated injection sites (inflammation with or without necrosis, myofibre necrosis and/or regeneration, and/or interstitial fibrosis).

Accumulation of foreign material-laden macrophages and giant cells was noted in the lumbar and inguinal lymph nodes of 1/3 males and 1/3 females, respectively.

3. INTRODUCTION

This report presents the pathology findings in rabbits assigned to the study entitled *A 9 Week Study of MenPF-1 Vaccine by Intramuscular Injection in Rabbits with a 4 Week Recovery Period* (Study No. 520419). The objective of this study was to determine the potential toxicity of MenPF-1 Vaccine, a prophylactic vaccine for the prevention of infection from bacterial meningitis, when given by intramuscular injection for 4 occasions over a 9 week period to rabbits, to evaluate the potential reversibility of any findings, and to provide data to support the use of MenPF-1 in humans.

The study was sponsored by Oxford Vaccine Group, UK where Andrew J Pollard, FRCPCH, PhD, served as the Sponsor representative. Bruce Robertson, BSc, Charles River, Edinburgh, UK served as the Study Director.

Appendix 26 (continued)
Pathology Report

4. MATERIALS AND METHODS

Experimental procedures applicable to pathology investigations are summarised in Text Table 1 and Text Table 2.

Text Table 1
Experimental Design

Group Number	Number of Animals				Test Item	Dosage (µg/dose)	Conc. (µg/mL)	Dose Volume (mL/dose)
	Main Study		Recovery					
	M	F	M	F				
1	3	3	3	3	MOX Control	0	0	0.5 mL
2	3	3	3	3	MenPF-1	25	50	0.5 mL
3	3	3	3	3	MenPF-1	50	50	2 x 0.5 mL

All animals were submitted for necropsy on Day 66 (Scheduled Euthanasia (Day 66) / Main Study) or Day 92 (Scheduled Euthanasia (Day 92) / Recovery Study). Necropsies were performed and organ weights were collected by Charles River, Edinburgh personnel. Except as noted in Text Table 2 tissues were collected in 10% neutral buffered formalin.

Text Table 2
Tissue Collection and Examination

Tissue	Weigh	Collect	Microscopic Evaluation	Comment
Administration site	-	X	X	Injection Site 1 and/or 2 (as appropriate) with additional muscle.
Animal identification	-	X	-	-
Artery. aorta	-	X	X	From thoracic segment.
Bone marrow smear	-	X	-	One bone marrow smear was collected from the femur (for possible examination). Bone marrow smears were allowed to air dry and were not fixed in formalin.
Bone marrow. femur	-	X	X	Collected with bone. femur
Bone marrow. sternum	-	X	X	Collected with bone. sternum
Bone. femur with articulating surface	-	X	X	Distal end to include femoral tibial joint.
Bone. sternum	-	X	X	-
Brain	X	X	X	Forebrain. midbrain. cerebellum. and medulla oblongata.
Cervix	-	X	X	Collected with uterus.
Epididymis	X	X	X	Separate weights and examination.
Eye	-	X	X	Separate examination; Preserved in Davidson's fixative.
Gallbladder	-	X	X	-
Gland. adrenal	X	X	X	Separate weights and examination.
Gland. lacrimal	-	X	X	Only 1 required for examination.
Gland, mammary	-	X	X	Collected with thoracic skin and included nipple; mammary gland was examined in females only
Gland. parathyroid	-	X	X	Collected with thyroid; Examined only if present in the routine section of thyroid.
Gland. pituitary	X	X	X	-
Gland. prostate	X	X	X	-

Appendix 26 (continued)
Pathology Report

Tissue	Weigh	Collect	Microscopic Evaluation	Comment
Gland, salivary	-	X	X	Submandibular: Only 1 required for examination.
Gland, seminal vesicle	-	X	X	
Gland, thyroid	X	X	X	Separate weights and examination: weight included parathyroid
Gross lesions/masses	-	X	X	-
Gut-associated lymphoid tissue	-	X	X	Collected with small intestine.
Heart	X	X	X	-
Kidney	X	X	X	Separate weights and examination.
Large intestine, appendix	-	X	X	
Large intestine, caecum	-	X	X	-
Large intestine, colon	-	X	X	-
Large intestine, rectum	-	X	X	-
Large intestine, sacculus rotundus	-	X	X	-
Liver	X	X	X	Gallbladder drained before weighing
Lung	X	X	X	Infused with 10% neutral buffered formalin after weighing.
Lymph node, mandibular	-	X	X	Only 1 required for examination.
Lymph node, mesenteric	-	X	X	-
Lymph node, lumbar	-	X	X	Left and right identified.
Lymph node, inguinal	-	X	X	Left and right identified.
Muscle, skeletal	-	X	X	From thigh
Nerve, optic	-	X	X	Preserved in Davidson's fixative: Examined only if present in the routine section of the eye.
Nerve, sciatic	-	X	X	Only 1 required for examination.
Oesophagus	-	X	X	-
Ovary	X	X	X	Separate weights and examination.
Oviduct	-	X	X	Only 1 required for examination. Collected with uterus.
Pancreas	-	X	X	-
Skin	-	X	X	Collected with mammary gland.
Small intestine, duodenum	-	X	X	-
Small intestine, ileum	-	X	X	-
Small intestine, jejunum	-	X	X	-
Spinal cord	-	X	X	Cervical, thoracic, lumbar.
Spleen	X	X	X	-
Stomach	-	X	X	Fundus and pylorus
Testis	X	X	X	Separate weights and examination: Preserved in Modified Davidson's fixative.
Thymus	X	X	X	-
Tongue	-	X	X	-
Trachea	-	X	X	-
Ureter	-	X	X	Only 1 required for examination.
Urinary bladder	-	X	X	-
Uterus	X	X	X	-
Vagina	-	X	X	-

X = procedure conducted: - = not applicable.

Tissues required for microscopic evaluation were trimmed, processed routinely, embedded in paraffin, cut 4-6 µm thick, mounted on glass slides, and stained with hematoxylin and eosin (H&E) by Charles River, Edinburgh personnel. Microscopic evaluation was conducted by

Appendix 26 (continued)
Pathology Report

the undersigned Board-certified Veterinary Pathologist on all protocol-specified tissues from Main Study animals in Groups 1 and 3; and on injection site and lumbar and inguinal lymph nodes from Recovery animals in Groups 1 and 3.

Tissues were evaluated by light microscopy, and the results were entered directly into a validated pathology computer program (PLACES 2000, Instem) for preparation of data tables.

4.1. Peer Review

All tissues from Animals 7, 9, 17 and 18; and all injection sites from Animals 19-21, 25-30, and 34-36 were examined by a second pathologist. Any differences in recording, grading or description of the findings were discussed by the Study Pathologist and Peer Reviewing Pathologist. The data in this report reflect the consensus view of the Study Pathologist and Reviewing Pathologist.

4.2. Computerized Systems

The data described in this report were generated by direct computer entry using PLACES 2000 Software version 1 supplied by Instem.

The files referred to in this report are listed below:

PLAFOR_520419_MACMAIN_LL_KEEP2
PLAFOR_520419_MACREC_LL_KEEP1
PLAFOR_520419_MICMAIN_LBE_KEEP1
PLAFOR_520419_MICREC_LBE_KEEP1

5. RESULTS AND DISCUSSIONS

5.1. Gross Pathology

5.1.1. Scheduled Euthanasia (Day 66)

Test article-related gross pathology findings are summarised in Text Table 3.

Text Table 3
Summary Gross Pathology Findings - Scheduled Euthanasia (Day 66)

	Males			Females		
Group	1	2	3	1	2	3
Dose (µg/dose)	0	25	50	0	25	50
No. animals examined	3	3	3	3	3	3
Lumbar lymph node (No. examined)	3	3	3	3	3	3
Enlargement, left	0	0	2	0	0	1

Other gross findings observed were considered incidental, of the nature commonly observed in this strain and age of rabbit, and/or were of similar incidence in control and treated animals and, therefore, were considered unrelated to administration of MenPF-1 vaccine.

Appendix 26 (continued)
Pathology Report

5.1.2. Scheduled Euthanasia (Day 92)

Test article-related gross findings noted at the terminal euthanasia were not observed at the end of the recovery period. Other gross findings observed were considered incidental, of the nature commonly observed in this strain and age of rabbit, and/or were of similar incidence in control and treated animals and, therefore, were considered unrelated to administration of MenPF-1 vaccine.

5.2. Organ Weights

No test article-related organ weight changes were noted at the end of the treatment and recovery periods. There were isolated organ weight values that were statistically different from their respective controls. There were, however, no patterns, trends, or correlating data to suggest these values were toxicologically relevant. Thus, the organ weight differences observed were considered incidental and unrelated to administration of Men-PF1 vaccine.

5.3. Histopathology

5.3.1. Scheduled Euthanasia (Day 66)

Test article-related microscopic findings are summarised in Text Table 4.

Appendix 26 (continued)
Pathology Report

Text Table 4
Summary Microscopic Findings – Scheduled Euthanasia (Day 66)

	Males		Females	
**Group**	1	3	1	3
**Dose (µg/dose)**	0	50	0	50
**No. animals examined**	3	3	3	3
**Injection site 1 (No. Examined)**	3	3	3	3
Macrophage accumulation, intramuscular	(3)[a]	(3)	(3)	(3)
Minimal	0	0	0	2
Mild	0	1	2	0
Moderate	3	2	1	1
Inflammation, polymorphonuclear leukocytic	(0)	(2)	(0)	(2)
Mild	0	0	0	1
Moderate	0	1	0	1
Marked	0	1	0	0
Inflammation, mononuclear cell	(2)	(1)	(2)	(1)
Minimal	0	0	0	1
Mild	1	1	2	0
Moderate	1	0	0	0
Myofibre necrosis	(1)	(2)	(1)	(1)
Minimal	0	0	1	0
Mild	1	1	0	1
Moderate	0	1	0	0
Myofibre regeneration	(0)	(1)	(1)	(2)
Minimal	0	0	1	2
Mild	0	1	0	0
Fibrosis, interstitial	(0)	(2)	(0)	(2)
Minimal	0	0	0	1
Mild	0	1	0	1
Moderate	0	0	0	0
Marked	0	1	0	0
Mineralisation	(0)	(0)	(0)	(1)
Minimal	0	0	0	1
**Lumbar lymph node (No. Examined)**	3	3	3	2
Macrophage accumulation	(1)	(2)	(1)	(2)
Minimal	1	2	0	2
Mild	0	0	1	0
Lymphoid hyperplasia	(0)	(2)	(0)	(2)
Minimal	0	0	0	0
Mild	0	1	0	1
Moderate	0	1	0	1

[a] Numbers in parentheses represent the number of animals with the finding.

The accumulation of macrophages, observed both in the injection sites and lumbar lymph nodes, was characterised by aggregates of macrophages containing an abundant, pale basophilic, amorphous cytoplasmic material considered to be aluminium hydroxide. These macrophages were admixed with variable numbers of multinucleated giant cells.

The lymphoid hyperplasia correlated with the enlarged lumbar lymph nodes observed at necropsy.

Other microscopic findings observed were considered incidental, of the nature commonly observed in this strain and age of rabbit, and/or were of similar incidence and severity in control and treated animals and, therefore, were considered unrelated to administration of MenPF-1.

Appendix 26 (continued)
Pathology Report

A number of changes were observed in the clinical chemistry, haematology and coagulation group mean values, when compared to their respective controls: there were increased total proteins and globulins, and decreased albumin/globulin ratio in all treated males and in females given 50 µg/dose; increased neutrophil counts in males given 50 µg/dose; increased monocyte counts in all treated male groups; and increased fibrinogen in treated groups from both sexes. These differences correlated with the inflammatory reaction observed in the injection sites.

5.3.2. Scheduled Euthanasia (Day 92)

Some of the microscopic findings noted at the terminal euthanasia were observed at the end of the period off dose (Day 92) and are summarised in Text Table 5.

Text Table 5
Summary Microscopic Findings – Scheduled Euthanasia (Day 92)

	Males		Females	
**Group**	1	3	1	3
**Dose (µg/dose)**	0	50	0	50
**No. animals examined**	3	3	3	3
**Injection site 1 (No. Examined)**	3	3	3	3
Macrophage accumulation, intramuscular	(3)[a]	(2)	(1)	(2)
Minimal	0	0	0	1
Mild	1	0	0	0
Moderate	2	2	1	1
Inflammation, with necrosis	(0)	(2)	(0)	(1)
Minimal	0	1	0	0
Mild	0	1	0	1
Inflammation, mononuclear cell	(0)	(0)	(0)	(1)
Minimal	0	0	0	1
Myofibre necrosis	(0)	(1)	(0)	(1)
Minimal	0	1	0	0
Myofibre regeneration	(0)	(2)	(0)	(1)
Minimal	0	2	0	1
Fibrosis, interstitial	(0)	(2)	(0)	(1)
Minimal	0	0	0	1
Mild	0	2	0	0
**Inguinal lymph node (No. Examined)**	3	3	3	3
Macrophage accumulation	(0)	(0)	(0)	(1)
Minimal	0	0	0	1
**Lumbar lymph node (No. Examined)**	3	3	2	1
Macrophage accumulation	(3)	(1)	(1)	(0)
Minimal	1	1	1	0
Mild	2	0	0	0

[a] Numbers in parentheses represent the number of animals with the finding.

No treatment related findings were noted in Injection Site 2 (Animal 27).

Other microscopic findings observed were considered incidental, of the nature commonly observed in this strain and age of rabbit, and/or were of similar incidence and severity in control and treated animals and, therefore, were considered unrelated to administration of MenPF-1.

Appendix 26 (continued)
Pathology Report

6. CONCLUSIONS

Intramuscular administration of MenPF-1 vaccine to rabbits on 4 occasions at 50 µg/dose resulted in the accumulation of foreign material-laden macrophages and giant cells at the injection site for all animals. Polymorphonuclear inflammation was noted in 2/3 males and 2/3 females, and mononuclear inflammation was noted in 1/3 males and 1/3 females. Myofibre necrosis and/or regeneration, interstitial fibrosis and/or mineralisation were also observed in treated injection sites.

Lumbar lymph node enlargement was observed at necropsy in 2/3 males and 1/3 females, with corresponding lymphoid hyperplasia. Accumulation of foreign material-laden macrophages and giant cells was noted in the lumbar lymph nodes of 2/3 males and 2/2 females.

After a 4 week recovery period, a number of findings persisted in treated injection sites (inflammation with or without necrosis, myofibre necrosis and/or regeneration, interstitial fibrosis).

Accumulation of foreign material-laden macrophages and giant cells was noted in the lumbar and inguinal lymph nodes of 1/3 males and 1/3 females, respectively.

Appendix 26 (continued)
Pathology Report

7. REPORT APPROVAL

______________________________ Date: 09 FEB 2012

Lise Bertrand, DVM, MSc, DESV, Dipl ECVP
Study Pathologist
Charles River, Edinburgh, UK

Appendix 26 (continued)
Pathology Report

charles river

PEER-REVIEW CERTIFICATE

CHARLES RIVER STUDY NO. 520419

A 9 Week Study of MenPF-1 Vaccine by Intramuscular Injection in Rabbits with a 4 Week Recovery Period

EXPERIMENTAL DESIGN: MenPF-1 Vaccine is a prophylactic vaccine for the prevention of infection from bacterial meningitis. The objective of this study was to determine the potential toxicity of MenPF-1 Vaccine when given by intramuscular injection for 4 occasions over a 9 week period to rabbits to evaluate the potential reversibility of any findings.

PURPOSE: The purpose of this peer review was to assess the overall quality and consistency of the microscopic data and determine the validity of the study pathologist's conclusions. The peer review for this study was conducted in accordance with the OECD Principles of Good Laboratory Practice as incorporated into the United Kingdom Statutory Instrument for GLP and as accepted by Regulatory Authorities throughout the European Community, United States of America (FDA and EPA) and Japan (MHLW, MAFF and METI).

METHODS:
1. Review of all tissues from animal numbers: 7 and 9 (Group 3 males), and 17 and 18 (Group 3 females).
2. Review of injection site 1 from all recovery animals.
3. Following review of the histologic sections and corresponding histopathology-related study data, findings were discussed with the study pathologist.

RESULTS:
Slides examined were of good quality with minimal artefacts (e.g. occasional minor folding, chattering, cracking during processing: occasional bone fragments from necropsy).

Any differences of opinion were resolved and mutual agreement on terminology and diagnoses were achieved. The histopathology tables and corresponding narrative contained in the pathology report reflect diagnoses and conclusions agreed to by the peer reviewer and study pathologist. No further action is recommended.

[signature] — 16 Dec 2011
PRP Signature — Date
PRP Petrina Rogerson, BMVS MRCVS

[signature] — 16 DEC 2011
SP Signature — Date
SP Lise Bertrand, DVM MSc DESV Dipl ECVP

cc J-Drive
Study File (Original)
